# Divergent copper-catalyzed syntheses of 3-carboxylpyrroles and 3-cyanofurans from *O*-acetyl oximes and β-ketoesters/nitriles†

**DOI:** 10.1039/d2ra04938d

**Published:** 2022-09-21

**Authors:** Wilfrido E. Almaraz-Ortiz, Aldahir Ramos Orea, Oscar Casadiego-Díaz, Agustín Reyes-Salgado, Arturo Mejía-Galindo, Rubén O. Torres-Ochoa

**Affiliations:** Instituto de Química, Universidad Nacional Autónoma de México, Circuito Exterior, Ciudad Universitaria, Coyoacán Ciudad de México 04510 Mexico romar.torres@iquimica.unam.mx https://www.iquimica.unam.mx

## Abstract

The reaction between *O*-acetyl oximes and β-ketoesters/nitriles catalyzed by copper generated polysubstituted pyrroles and furans, respectively, under redox–neutral reaction conditions. Using this protocol, pyrroles or furans could be obtained simply by choosing an appropriate active methylene compound. Although both transformations occur essentially under the same reaction conditions, control experiments indicated that they follow different mechanistic pathways.

## Abstract 2

A copper-catalyzed heteroannulation of *O*-acetyl oximes and β-ketoesters/nitriles to afford polysubstituted pyrroles and furans was successfully achieved.

## Graphical abstract

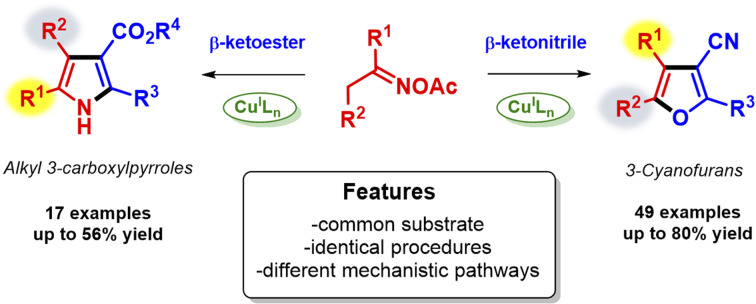

## Introduction

**Scheme 1:**
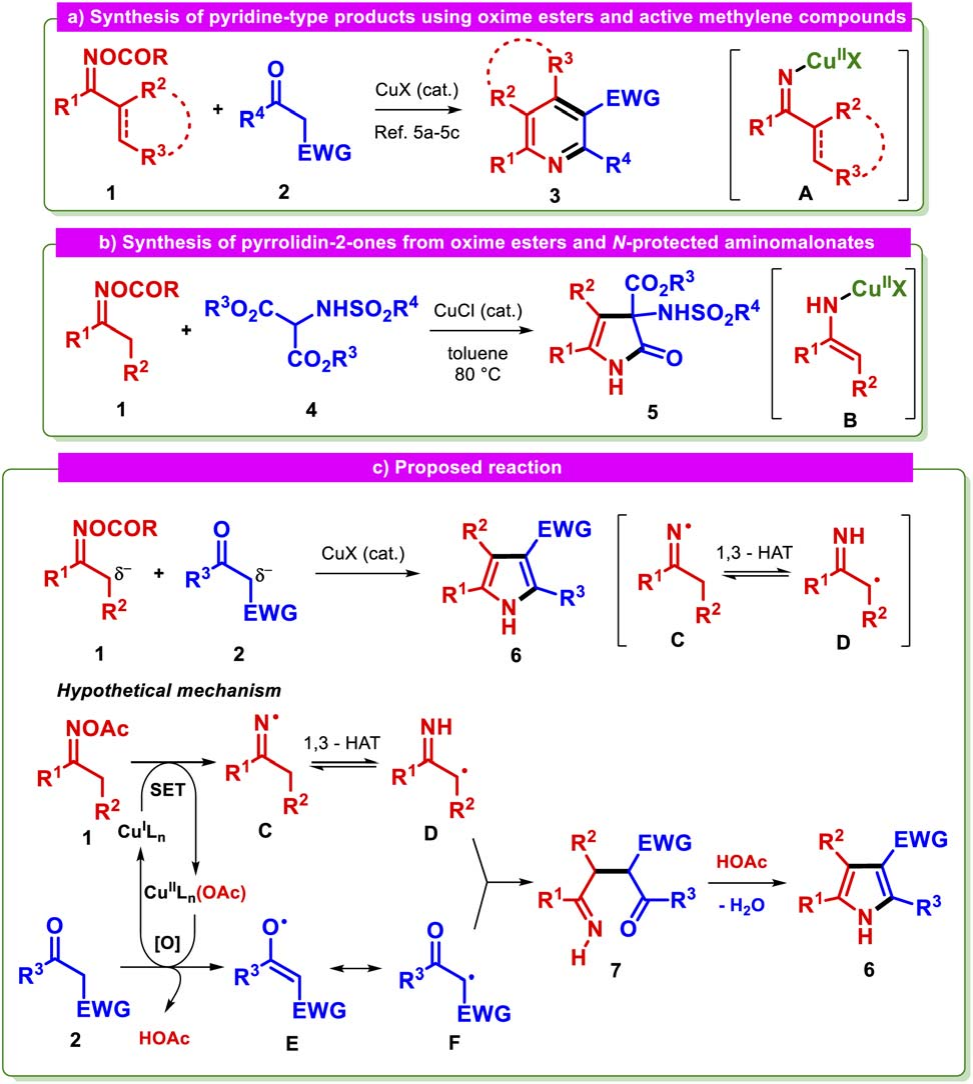
(a and b) Previously reported reactions between oxime esters and active methylene compounds. (c) Designed reaction and its proposed mechanism.

Redox-active oxime esters 1 are versatile building blocks used in a myriad of synthetic transformations beyond the classical reactions.^1^ This group of substrates can be effectively activated by transition metals under mild conditions with chemo- and regioselectivity.^2^ Copper represents a gentle, abundant, and inexpensive reaction mediator between oxime esters and a variety of components, forming the basis of a noteworthy arsenal of synthetic procedures,^3^ including annulations.^2*c*,4^ N–C–C and N–C–C–C synthons are commonly provided by oxime esters, facilitating the formation of medicinally important N-containing heterocyclic compounds. For instance, the reaction of oxime esters 1 with active methylene compounds 2 in the presence of a copper catalyst affords structurally diverse pyridines or fused-pyridines 3 through a [4 + 2] cyclization, with the organocopper species A as a central intermediate (Scheme 1a).^5^ In these reactions, the nucleophilic character of either 2 or A typically triggers the desired transformation.^6^ To the best of our knowledge, only one synthetic method is known to give a different type of product employing oxime esters as substrates. This process utilizes the readily oxidized *N*-protected α-aminomalonate 4 to generate 3-sulfonamido-4-pyrrolin-2-ones 5 and has as a key step the nucleophilic attack of species B on the *in situ* formed *N*-protected imine (Scheme 1b).^7,8^ Despite these previous protocols, there has been a notable lack of investigations into other common reaction intermediates derived from oxime esters with active methylene compounds. It is possible that such intermediates may modify the reaction course, allowing the synthesis of distinct products.

Iminyl radicals derived from the reduction of the N–O bond in oxime esters *via* single-electron transfer (SET) have recently attracted renewed interest because of the development of alternative syntheses with milder conditions,^9^ as well as their proclivity to form carbon-centered radicals,^10^*e.g.*, α-iminyl radicals form *via* a 1,3-hydrogen atom transfer process (1,3-HAT).^10*b*,11^ Based on our interest in metal-mediated reactions using oxime esters,^12^ we initially explored the possibility of preparing remarkable heterocycles such as pyrroles 6 starting from oxime esters 1 and active methylene compounds 2 through reaction pathways involving the iminyl radicals C and D (Scheme 1c).

We hypothesized that this transformation could be accomplished if the copper catalyst were to play a dual role in the reaction, acting as both an oxime ester reductant and as an oxidant for the active methylene compound. This would allow us to simultaneously access crucial radicals D and F, which would be expected to undergo selective radical/radical cross-coupling^13^ if species F behaves as a persistent radical. The planned strategy aimed to alter the nucleophilic character of the carbon atoms which are predicted to be bound to each other in the key step. Beyond the critical C–C bond formation stage, the success of the tandem reaction will depend on fulfilling the following requirements: (a) selective formation of the iminyl radical C, (b) transformation of such an intermediate to the α-iminyl species D, and (c) oxidation of the active methylene compound 2. The synthetic value of the proposed methodology derives from the importance of pyrroles as the cores of many natural products, including drugs and agrochemicals.^14^

## Results and discussion

To test our hypothesis, we began by investigating the reaction between *O*-acetyl oxime 1a and methyl acetoacetate (2a). The highest yield was obtained with the conditions shown in the reaction scheme embedded in Table 1, which afforded the pyrrole 6a^15^ in 56% isolated yield.^16^

**Table 1:** Optimization of reaction conditions^a^

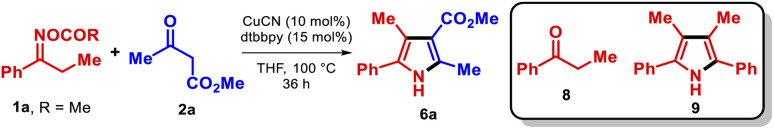		
Entry	Deviation from the standard conditions	Yield^b^ (%)
1	None	65% (56%)^c^^,^^d^
2	Other Cu^I^ and Cu^II^ salts	<36%
3	0.037 M/0.05 M	38%/47%
4	Li_2_CO_3_, Na_2_CO_3_, K_2_CO_3_, Et_3_N	<45%
5	3 Å, 4 Å, 5 Å MS	<48%
6	Na_2_S_2_O_3_/Na_2_SO_3_/NaHSO_3_	30%/49%/0%
7	Mn(OAc)_3_, CAN, DCP, TBHP	<48%
8	R = *t*Bu, Ph, C_6_H_4_-*p*CF_3_, C_6_F_5_	<44%
9	Without Cu	NR

a Reaction conditions: 1a (75 μmol), 2a (2 equiv.), CuCN (10 mol%), dtbbpy (15 mol%) in 1 mL THF (0.075 M) at 100 °C for 36 h.

b NMR yield.

c 1a (0.15 mmol scale).

d Isolated yield. NR = no reaction.

During the optimization phase, the importance of using CuCN as the copper source became clear since other salts did not furnish comparable results (entry 2). Modification of the reaction concentration, and the addition of Brønsted bases or molecular sieves did not boost the yield either (entries 3–5). The major product observed in most of the experiments was propiophenone (8), which can form from reactive intermediates A or B. To avoid this undesired product, several reductants^11*b*,17^ and oxidants were added to the reaction (entries 6, 7). Unfortunately, none of these additives hindered the formation of propiophenone, demonstrating that the inhibition of parasitic pathways is not straightforward. Reactions using *O*-acyl oximes were ineffective at producing the pyrrole 6a in higher yields (entry 8). We also corroborated that Cu is essential for the reaction (entry 9). As a final remark, during the optimization we often observed the presence of pyrrole 9, which may indicate the participation of the α-iminyl radical D in this transformation.^11*c*^

We proceeded to test the reaction with a set of aryl alkyl *O*-acetyl oximes to determine its scope (Table 2). Electron-donating groups at *p*- and *m*-positions gave the expected pyrroles 6a–6c and 6h in good yields; a slightly diminished yield was observed when either halogens or electron-withdrawing substituents were present at the *p*- and *m*-positions (6d–6f). Other β-ketoesters were also tolerated although ethyl isobutyrylacetate and ethyl benzoylacetate reacted to form pyrroles but less efficiently (6k, 6l). Notably, 4,5-diaryl pyrroles were also synthesized in moderate yields (6m, 6n); such compounds have shown important bioactivities,^14*a*^ for example atorvastatin acts as a lipid-lowering agent.^18^ Other substrates with a larger alkyl side chain that might undergo a 1,5-HAT process^10*a*,*b*^ gave the products 6o and 6p. Lastly, a cyclooctanone oxime derivate afforded the fused pyrrole 6q in 34% yield. Unfortunately, the *O*-acetyl oximes 1 in which R^1^ was an alkyl chain only formed the parent ketones. This class of substrates likely followed the pathway towards undesired species A and B.

**Table 2:** Substrate scope with some oxime esters (synthesis of pyrroles 6a–6q)

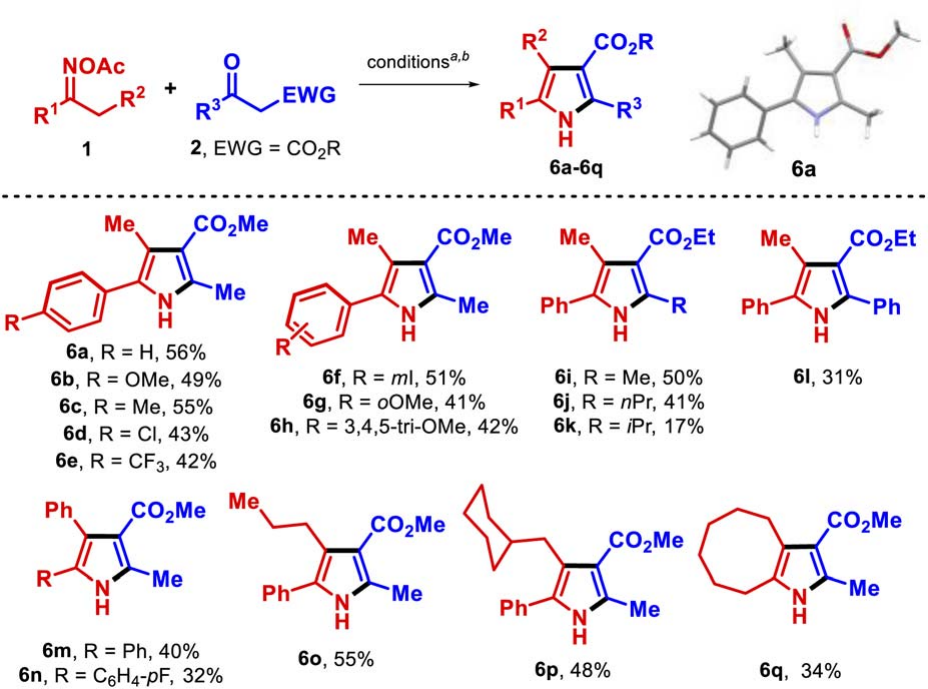

a 1 (0.15 mmol), 2 (0.3 mmol), CuCN (10 mol%), dtbbpy (15 mol%) in 2 mL THF (0.075 M) at 100 °C for 36 h.

b Isolated yield.

Next, we focused on the effect of using another active methylene compound, such as a β-ketonitrile, instead of a β-ketoester. When *O*-acetyl oxime 1a was reacted with benzoylacetonitrile (2b), 3-cyanofuran 10a (45% isolated yield) was obtained instead of the expected 3-cyanopyrrole (Table 3). The product was preliminarily identified using spectroscopic data, and was unambiguously confirmed by single-crystal X-ray diffraction analysis (CCDC 2155771†).^19^ Surprisingly, the regioselectivity of the reaction also changed, in that the substituents at the 4- and 5-positions were transposed compared to pyrroles 6. Remarkably, 10a was obtained in higher yield using only 1.1 equiv. of 2b (70% isolated yield), although propiophenone 8 was still observed as the main byproduct. To our knowledge, this represents the first synthesis of furans from oxime esters.^20^ 3-Cyanofurans are useful blocks for synthesizing complex molecules^21^ and have shown interesting UV-absorbing properties;^22^ thus, the development of novel protocols to access those heterocycles is an attractive goal.^23^ When the transformation was applied to the formerly synthesized oximes 1 and others oxime esters, these molecules reacted with 2b to provide a wide range of furans in a more efficient fashion than was observed in the corresponding syntheses of pyrroles described above (Table 3).

**Table 3:** Substrate scope with aryl alkyl oxime esters (synthesis of furans 10a–10ab)

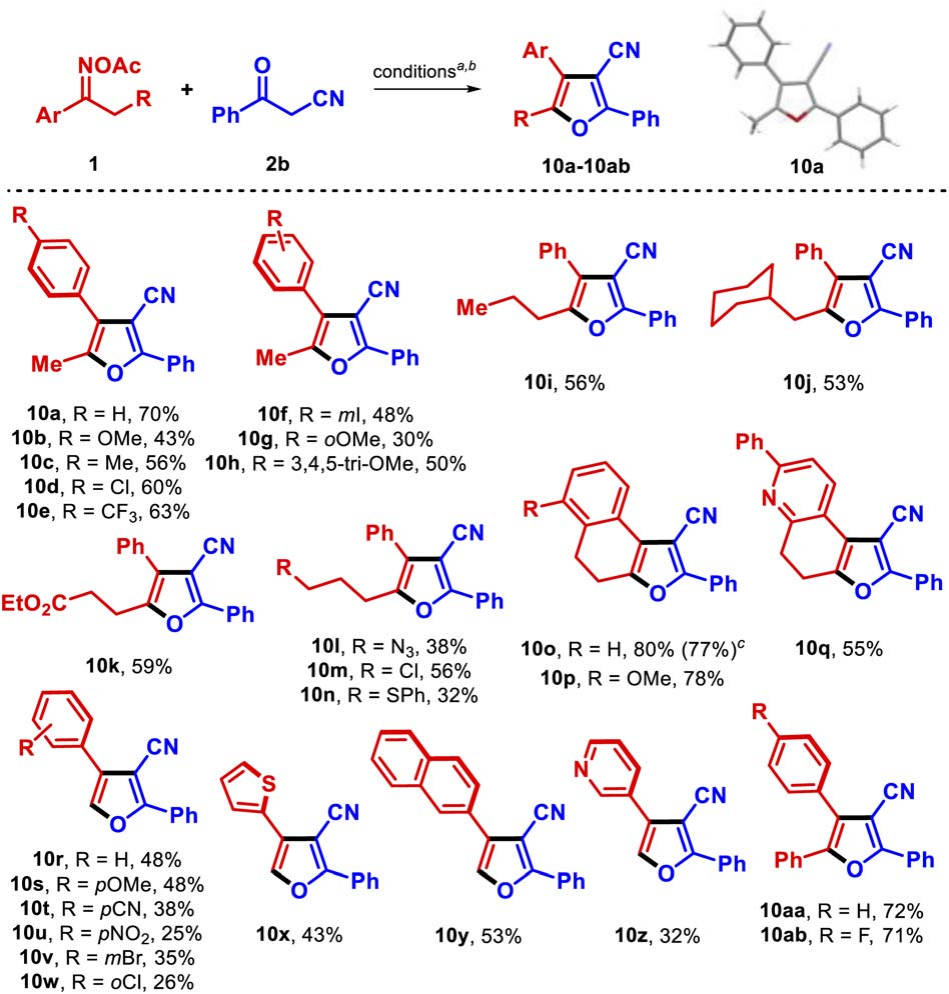

a 1 (0.15 mmol), 2b (1.1 equiv.), CuCN (10 mol%), dtbbpy (15 mol%) in 2 mL THF (0.075 M) at 100 °C for 36 h.

b Isolated yield.

All but one of the substituted propiophenone oxime derivatives reacted as intended to generate furans in good yields (10b–10f, 10h); the exception was the *O*-methoxy substituted substrate, which gave poorer results due to steric constraints (10g). Substrates with an extended aliphatic side chain reacted well (10i, 10j), even those bearing more sensitive functionalities such as ester, azido, chloro, and thioether groups (10k–10n). Those functional groups have the potential to be exploited for the further derivatization of the products. α-Tetralone oximes produced the tricyclic products 10o and 10p in high yield, with the nucleus of these compounds resembling the natural product laevigatin.^24^ The tetrahydroquinolinone oxime derivative efficiently led to dihydrofuroquinoline 10q in 55% yield. The robustness of the method was demonstrated by isolating 10o in 77% yield from a 1.5 mmol scale reaction. In addition, acetophenone *O*-acetyl oximes and related compounds were transformed into the respective 2,3,4-trisubstituted furans 10r–10z, although in somewhat inferior yields. These low yields can be attributed to a slower radical isomerization rate in these substrates arising from the lack of an electron-rich alkyl substituent in the α-iminyl radical intermediate. Furthermore, in the reactions with substrates bearing either electronegative or electron-withdrawing substituents, hydrolysis of the *O*-acetyl moiety was observed. Pleasingly, the use of α-phenylacetophenones as substrates allowed the smooth synthesis of the significant triaryl-3-cyanofurans 10aa and 10ab.

The methodology was next applied to the group of dialkyl oximes 1aa (Table 4). Furans 10ac–10ag were successfully obtained under the optimized conditions in good yields. Interestingly, the reaction of 2,6-dimethylheptan-4-one oxime ester with benzoylacetonitrile also afforded the alkene 12, likely through condensation between the transient imine 11 and benzoylacetonitrile (2b). In principle, such a side product might be involved in the reaction pathway as one of the transient intermediates. Particularly, oxime ester 1ab yielded a 10 : 1 mixture of furans 10ah and 10ai owing to the existence of two distinct α-methylenes. In the cases of the phenoxyacetone and 3-phenoxybutan-2-one oxime derivatives 1ac and 1ad, the furan products 10aj–10ak were found not to contain the phenoxy moiety, while the reaction of the 1-(phenylthio)propan-2-one oxime ester 1ae with benzoylacetonitrile afforded an inseparable mixture of products 10aj and 10al.

**Table 4:** Substrate scope with dialkyl oxime esters (synthesis of furans 10ac–10al)

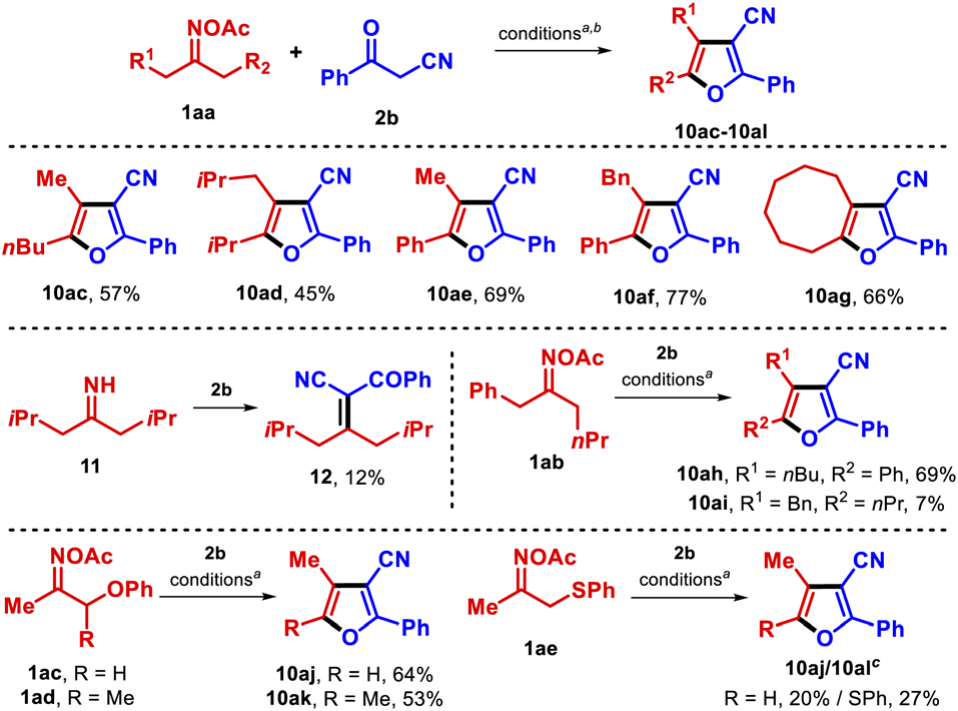

a 1aa (0.15 mmol), 2b (1.1 equiv.), CuCN (10 mol%), dtbbpy (15 mol%) in 2 mL THF (0.075 M) at 100 °C for 36 h.

b Isolated yield.

c Inseparable mixture.

We also explored the products obtained when different acylacetonitriles were used as reactants (Table 5). Furans 10am–10as were obtained in good yields, with the presence of electron-withdrawing substituents on the acylacetonitrile having a positive influence on the transformation (10ao–10aq). Notably, reaction of aliphatic propionylacetonitrile with 1a afforded the furan 10at in 66% yield. 2-Pyrenyl-3-cyanofuran 10au was also synthesized and exhibited fluorescent features under UV-light. Lastly, beyond the model oxime ester 1a, other substrates successfully reacted with substituted benzoylacetonitriles to produce furans 10av and 10aw.

**Table 5:** Substrate scope with diverse acylacetonitriles (synthesis of furans 10am–10aw)

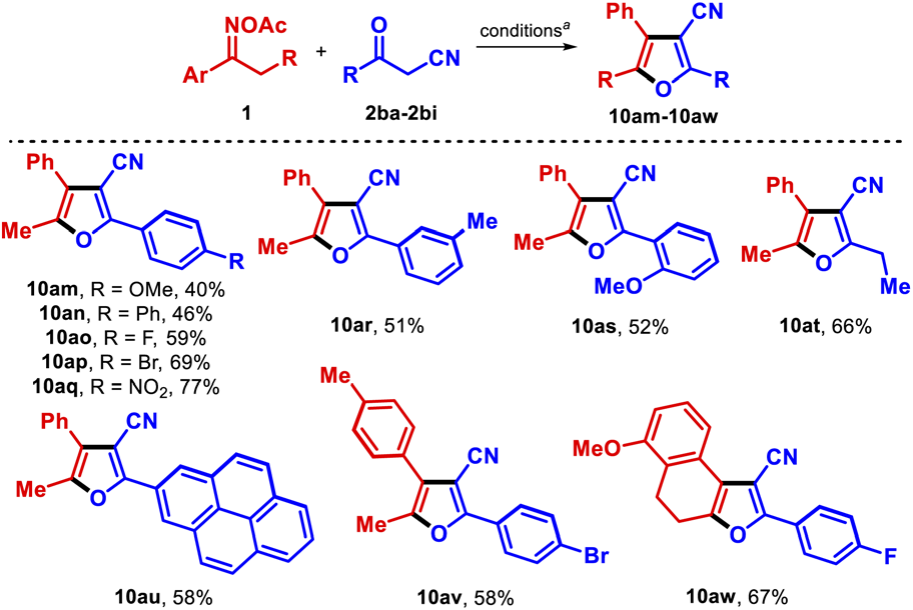

a 1 (0.15 mmol), 2ba–2bi (1.1 equiv.), CuCN (10 mol%), dtbbpy (15 mol%) in 2 mL THF (0.075 M) at 100 °C for 36 h.

b Isolated yield.

**Scheme 2:**
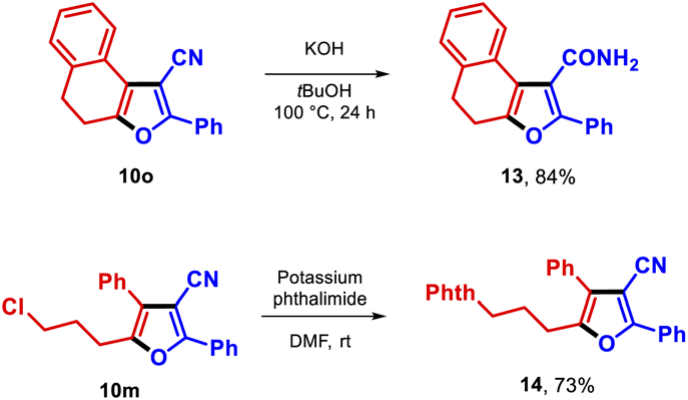
Derivatization of furans 10o and 10m.

Finally, derivatization of the furan-3-carbonitrile 10o was achieved by transforming this furan into the carboxamide 13 in high yield. Additionally, the chloro functionality in compound 10m was successfully substituted by potassium phthalimide, yielding compound 14 in 73% yield (Scheme 2).

**Scheme 3:**
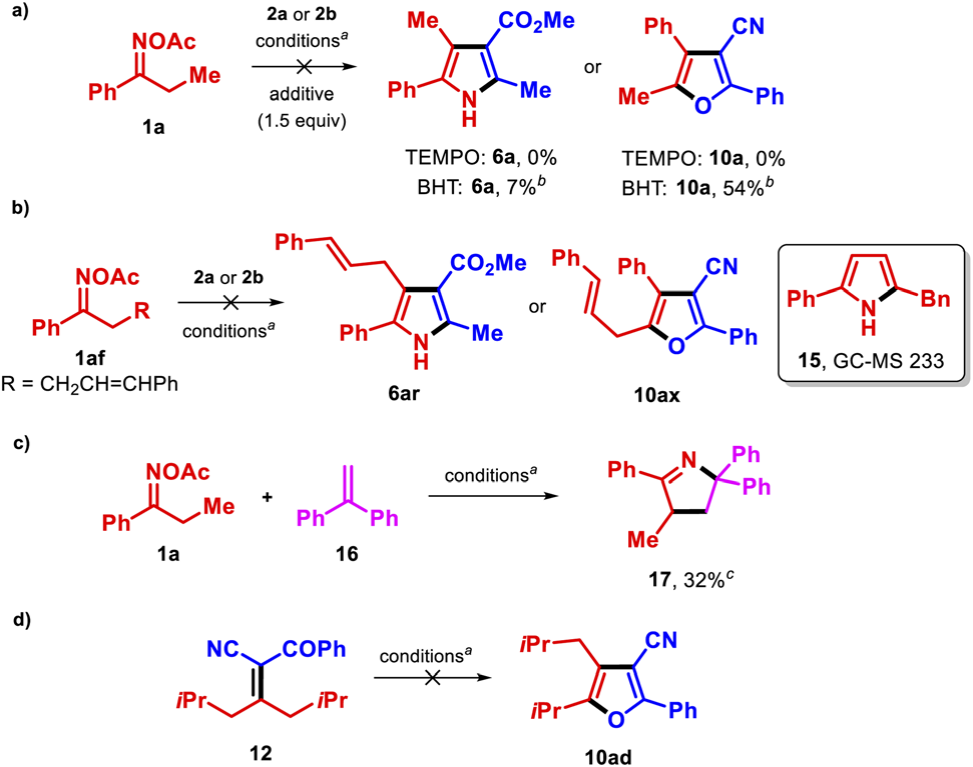
Control experiments. ^*a*^CuCN (10 mol%), dtbbpy (15 mol%) in THF (0.075 M) at 100 °C for 36 h. ^*b*^NMR yield. ^*c*^Isolated yield.

We next performed experiments to gain insights into the reaction mechanism (Scheme 3). We found that the addition of an external oxidant such as TEMPO to the reaction of 1a with either methyl acetoacetate (2a) or benzoylacetonitrile (2b) completely suppressed the reaction, and that the presence of the non-oxidizing radical scavenger *tert*-butylhydroxytoluene (BHT) affected the reactions of 2b and 2a with 1a differently, with the former reaction being less impacted (Scheme 3a). These results were consistent with the proposed radical mechanism *via* SET to the copper catalyst in the synthesis of pyrroles (Scheme 1c), although it is probable that a somewhat different pathway is operative in the formation of furans 10. Additionally, reaction of the *O*-acetyl oxime bearing an alkene-tethered motif 1af with 2a or 2b did not yield the expected products 6r and 10ax (Scheme 3b). GC-MS analysis of those reactions suggested the presence of pyrrole 15, which could form *via* an iminyl radical γ,δ-cyclization.^25^ Thus, when 1af is used as a reactant, the latter process is apparently more rapid than the desirable 1,3-HAT process. The reaction between 1a and the radical acceptor 16 in the absence of the active methylene compound furnished the pyrroline 17^26^ in moderate yield (Scheme 3c), thus confirming the formation of an α-iminyl radical during the process. Finally, submission of nitrile 12 to the optimal conditions did not afford 10ad (Scheme 3d), ruling out its participation as an intermediate in the transformation.

**Scheme 4:**
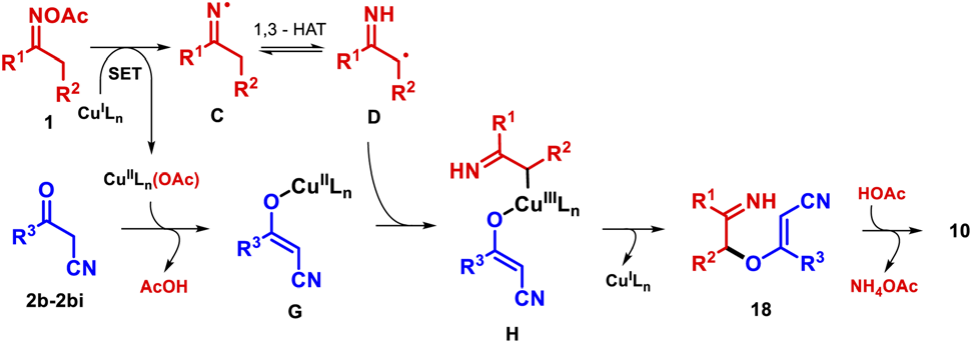
Alternative mechanism for the synthesis of furans 10.

On the basis of these results and literature reports,^27^ we suggest an alternative mechanistic pathway in the case of furans 10 (Scheme 4). We tentatively propose that a rapid trapping of radical D by the copper species G,^27*a*^ formed by ligand exchange, gives alkyl-Cu^III^ intermediate H.^25^ The latter undergoes reductive elimination to create the C–O bond and regenerates the active Cu^I^ catalyst.^28^ Subsequently, the intramolecular nucleophilic addition of C-3 to the imine in intermediate 18 takes place under AcOH catalysis, generating the product.

Although β-ketoesters could follow the same mechanism, it seems not to be operational; apparently, the chelation of Cu^II^ species by 2a followed by its oxidation is more favorable.

## Conclusions

In summary, we have developed two external-oxidant-free synthetic procedures to access alkyl 3-carboxylpyrroles and 3-cyanofurans *via* copper-mediated cyclization of oxime esters with β-ketoesters and β-ketonitriles, respectively. These protocols are technically identical but mechanistically different. Unlike previously reported synthetic pathways, which have nucleophilic organocopper species as intermediates, the proposed syntheses proceed *via* radical intermediates. In the procedures described here, the copper catalyst acts as both an oxime ester reductant and as an activator of the active methylene compounds. The preparation of furans was not only more effective but also displayed a broader scope due to the direct participation of the catalyst in the C–O bond formation step. The synthesis of pyrroles was less efficient than that of furans, possibly due to oxidation of the esters and radical–radical cross-coupling being outcompeted by other pathways. Despite the limitations observed in the synthesis of pyrroles, this reaction represents a proof of concept of a strategy that can potentially be used to synthesize other heterocycles. Currently, further studies are underway focused on understanding the divergence in the reaction pathway depending on the active methylene compound used.

## Author contributions

W. E. A.-O., A. R. O., O. C.-D., A. R.-S. and A. M.-G. prepared the substrates. W. E. A.-O. and A. R. O. performed and analyzed the experiments. R. O. T.-O. conceived and supervised the project. R. O. T.-O., W. E. A.-O. and A. R. O. wrote the manuscript.

## Conflicts of interest

There are no conflicts of interest to declare.

## Supplementary Material

RA-012-D2RA04938D-s001

RA-012-D2RA04938D-s002
