# Supplementary material for: Divergent copper-catalyzed syntheses of 3-carboxylpyrroles and 3-cyanofurans from O-acetyl oximes and β-ketoesters/nitriles
Source: RSC Adv. 2022 Sep 21;12(41):26673–9. doi: 10.1039/d2ra04938d (PMC9490516; doi:10.1039/d2ra04938d)
Supplement: RA-012-D2RA04938D-s001 [file RA-012-D2RA04938D-s001.pdf]

Electronic Supplementary Material (ESI) for RSC Advances.
This journal is © The Royal Society of Chemistry 2022

Supporting information

Divergent copper-catalyzed syntheses of 3-carboxylpyrroles and 3-cyanofurans from *O*-acetyl oximes and β-ketoesters/nitriles

Wilfrido E. Almaraz-Ortiz,[+,a] Aldahir Ramos Orea,[+,a] Oscar Casadiego-Díaz,[a] Agustín Reyes-Salgado,[a] Arturo Mejía-Galindo,[a] Rubén O. Torres-Ochoa*[a]

[a]Instituto de Química, Universidad Nacional Autónoma de México, Circuito Exterior, Ciudad Universitaria, Coyoacán, Ciudad de México, 04510, México. E-mail: romar.torres@iquimica.unam.mx, www.iquimica.unam.mx

Table of contents

1. General information **S2**
2. Synthesized oxime esters **S2**
3. General procedure for the synthesis of the *O*-acetyl oximes **S3**
4. General procedure for the synthesis of the β-ketonitriles **S4**
5. Reaction optimization **S5**
6. General procedure for the synthesis of pyrroles 6 **S12**
7. General procedure for the synthesis of furans 10 **S12**
8. Synthesis of furan 10o in large scale **S13**
9. Derivatization of the products **S13**
10. Mechanistic investigations **S14**
11. Spectroscopic data of pyrroles 6a-6q **S19**
12. Spectroscopic data of furans 10a-10aw **S22**
13. Crystallographic data of pyrrole 6a and furan 10a **S32**
14. Copies of ^{1}H NMR and ^{13}C NMR spectra **S34**

1. General information

Commercially available chemicals were purchased from Merck and Sigma Aldrich and were used without further purification. ^{1}H NMR and ^{13}C NMR (300 and 75 MHz / 400 and 100 MHz, respectively) spectra were acquired in $CDCl_3$ at 25 °C on a 300 MHz Jeol Eclipse, 300 MHz Fourier Brucker, 300 MHz Avance Bruker, and Bruker Avance III 400 MHz spectrometers. The chemical shifts are reported on the δ scale in parts per million (ppm) and calibrated to residual solvents (7.26 ppm in $CDCl_3$) for ^{1}H NMR and (77.16 ppm in $CDCl_3$) ^{13}C NMR. Some ^{1}H NMR spectra were calibrated using TMS as an internal reference (0 ppm). The peak shapes are indicated as follows: s, singlet; br s, broad singlet; d, doublet; t, triplet; q, quartet; quint, quintet; sext, sextet; sept, septet; m, multiplet. The coupling constant values (*J*) are reported in Hertz (Hz). Melting points were measured in open capillaries using a Mel-Temp apparatus and were not corrected. IR spectra were obtained using an FTIR Tensor 27 Bruker spectrometer. Mass spectra were recorded with a JEOL SX 102 A spectrometer by electronic impact (EI) and a Jeol AccuTOF DART instrument for high-resolution measurements. Reactions were monitored by TLC and visualized using a dual short-wavelength/long-wavelength UV lamp. Flash column chromatography was carried out on silica gel 60 (230-400 mesh ASTM) from Macherey-Nagel GmbH & Co. All solvents were distilled under a nitrogen atmosphere. Dichloromethane and acetonitrile were distilled from calcium hydride, and tetrahydrofuran was distilled from sodium benzophenone ketyl. X-ray diffraction data were obtained from Bruker Smart APEX II CCD diffractometer equipped with graphite monochromatic MoKα radiation.

2. Synthesized oxime esters

Aryl(Heteroaryl) alkyl oxime derivatives

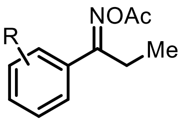

R = H, **1a**
R = *p*OMe, **S1a**
R = *p*Me, **S1b**
R = *p*Cl, **S1c**
R = pCF_3, **S1d**
R = *m*I, **S1e**
R = *o*OMe, **S1f**
R = 3,4,5-tri-OMe, **S1g**

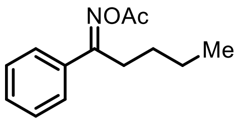

S1h

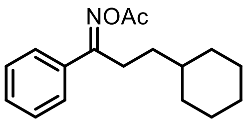

S1i

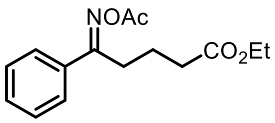

S1j

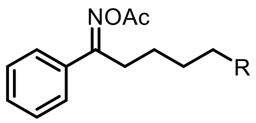

R = N_3, **S1k**
R = Cl, **S1l**
R = SPh, **S1m**

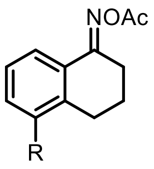

R = H, **S1n**
R = OMe, **S1o**

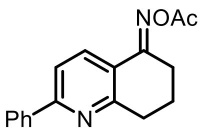

S1p

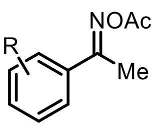

R = H, **S1q**
R = *p*OMe, **S1r**
R = *p*CN, **S1s**
R = pNO_2, **S1t**
R = *m*Br, **S1u**
R = *o*Cl, **S1v**

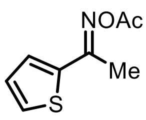

S1w

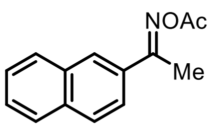

S1x

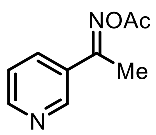

S1y

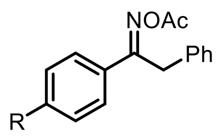

R = H, **S1z**
R = F, **S1a'**

Dialkyl oxime derivatives

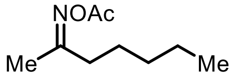

S1aa

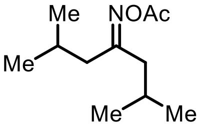

S1ab

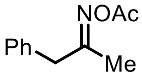

S1ac

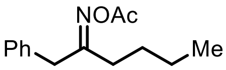

1ab

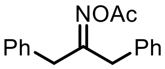

S1ad

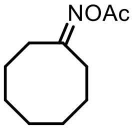

S1ae

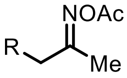

R = OPh, **1ac**
R = SPh, **1ae**

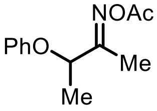

1ad

Acylacetonitriles

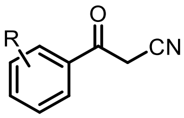

R = H, **2b**
R = *p*OMe, **2ba**
R = *p*Ph, **2bb**
R = *p*F, **2bc**
R = *p*Br, **2bd**
R = *p*NO_2, **2be**
R = *m*Me, **2bf**
R = *o*OMe, **2bg**

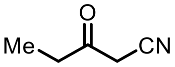

2bh

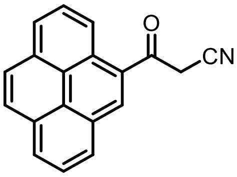

2bi

3. General procedure for the synthesis of the *O*-acetyl oximes

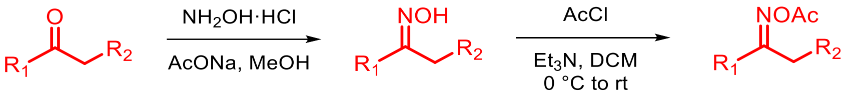

Ketoxime synthesis: A mixture of ketone (1 equiv), $NH_2OH{\cdot}HCl$ (1.2 equiv), and NaOAc (1.5 equiv) in methanol (3 mL/mmol) was stirred at room temperature until complete consumption of the starting material as determined by TLC. The reaction mixture was concentrated *in vacuo* until most of the solvent was removed. The reaction mixture was diluted with EtOAc and washed with water, 1 M HCl solution, saturated $NaHCO_3$ solution, and brine. The organic layer was dried over Na_2SO_4, the solvent was removed by evaporation under reduced pressure, and the residue was used directly in the next step.

***O*-Acetyl oxime synthesis:** The crude ketoximes were dissolved in anhydrous DCM (4 mL/mmol). After cooling the solution to 0 °C, acetyl chloride (1.2 equiv) was added, then Et_3N (1.5 equiv) dropwise. The mixture was stirred at room temperature until the completion of the reaction, as determined by TLC. The solvent was concentrated *in vacuo*. The reaction residue was redissolved with EtOAc and washed with water, 1 M HCl solution, saturated $NaHCO_3$ solution, and brine. The organic layer was dried over Na_2SO_4, and the solvent was removed *in vacuo*. The corresponding *O*-acetyl oximes were purified by column chromatography on silica gel.

4. General procedure for the synthesis of the β-ketonitriles

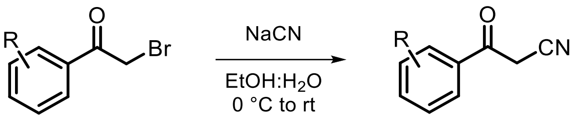

Benzoylacetonitriles 2b-2bg, 2bi: NaCN (3 equiv) was added in one single portion to a solution of the α-bromoketone (1 equiv) in a mixture EtOH:H_2O (4:1, 0.4 M) at 0 °C. The reaction was stirred for 1 h at the same temperature and then overnight at room temperature. The mixture was diluted with water and carefully acidified with concentrated HCl in a well-ventilated hood. The suspension was stirred for 15 min, then transferred to a separatory funnel. The crude product was extracted with EtOAc, the combined organic layer was dried over Na_2SO_4, and the solvent was removed *in vacuo*. The corresponding β-ketonitriles were purified by column chromatography on silica gel.

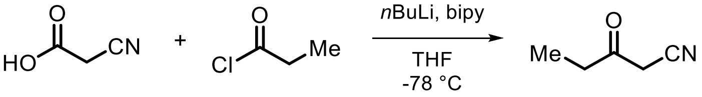

2bh

3-Oxopentanenitrile 2bh:[1] To a solution of cyanoacetic acid (0.49 g, 5.74 mmol) and 2-2-bipyridyl (ca. 3 mg) in THF (24 mL) was added *n*BuLi (a 2.0 M solution in hexanes) at -78 °C until a pink color persisted, indicating that a slight excess was present. The temperature was allowed to rise to -10 °C, additional *n*BuLi was added as needed to maintain the pink color, and the solution was cooled to -78 °C. The acid chloride (0.25 mL, 2.86 mmol) was added and stirred for 0.5 h. The mixture was poured into a separatory funnel containing DCM (30 mL) and 1 N HCl (10 mL). The organic phase was washed with sat. $NaHCO_3$ and dried over Na_2SO_4. The solvent was removed *in vacuo*, and nitrile **2bh** was purified by column chromatography on silica gel affording 68 mg of a light-yellow oil (24%).

[1] Y. Chen, S. M. Sieburth, *Synthesis*, **2002**, 2191.

5. Reaction optimization

5.1 Copper source

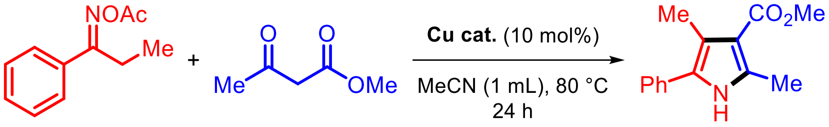

1a, 75 μmol **2a**, 2 equiv **6a**

Entry	Cu cat. (10 mol%)	NMR Yield (%)
1	CuBr	9%
2	CuCN	10%
3	$CuBr \cdot SMe_2$	NR
4	$(CF_3SO_3Cu)_2 \cdot C_6H_6$	NR
5	$(CF_3SO_3Cu)_2 \cdot C_6H_5CH_3$	NR
6	$Cu(OAc)_2$	NR
7	$Cu(OTf)_2$	NR
8	$CuBr_2$	NR
9	$CuSO_4 \cdot 5H_2O$	NR
10	Cu_2O	NR
11	$[CH_3(CH_2)_3CH(C_2H_5)CO_2]_2Cu$	<9%
12	$[Cu(MeCN)_4]PF_6$	<9%
13	$Cu(NCCH_3)_4 \cdot CF_3SO_3$	<9%
14	$Cu(acac)_2$	<9%
15	$Cu(C_5H_4F_3O_2)_2$	<9%

5.2 Ligand and temperature effect screening

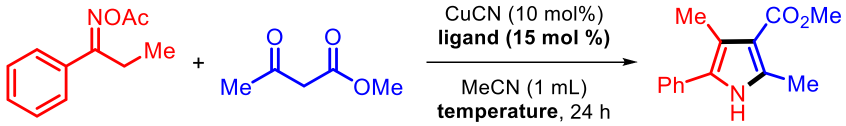

1a, 75 μmol **2a**, 2 equiv **6a**

Entry	Ligand (15 mol%)	Temperature	NMR Yield (%)
-	-	80 °C	10%
1	**L1**	80 °C	<9%
2	**L2**	80 °C	<9%
3	**L3**	80 °C	<9%
4	**L4**	80 °C	<9%
5	**L5**	80 °C	<9%
6	**L6**	80 °C	<9%
7	**L7**	80 °C	Traces
8	**L8**	80 °C	Traces
12	**L1**	100 °C	<25%
10	**L2**	100 °C	<20%
11	**L3**	100 °C	<20%
12	**L4**	100 °C	33%
13	**L5**	100 °C	<20%
14	**L6**	100 °C	<25%
15	**L9**	100 °C	<20%
16	**L10**	100 °C	<20%
17	**L4**	110 °C	25%

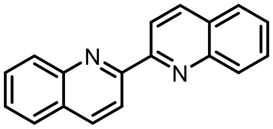

L1

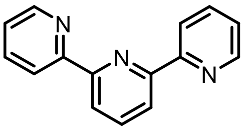

L2

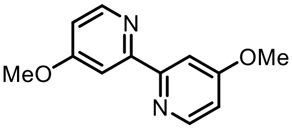

L3

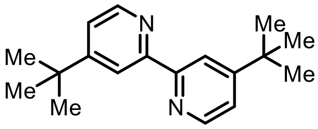

L4

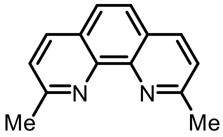

L5

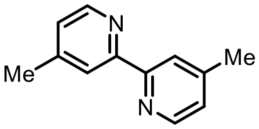

L6

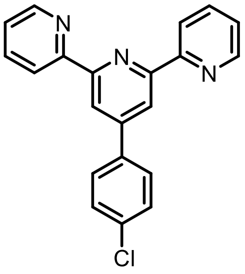

L7

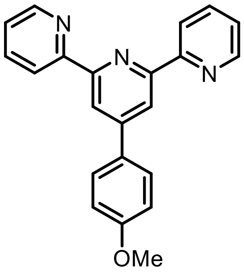

L8

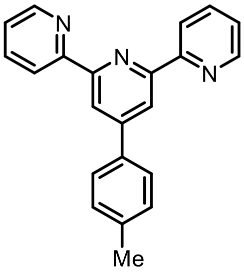

L9

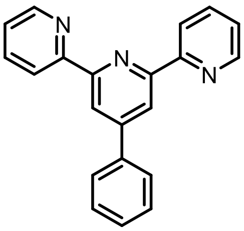

L10

5.3 Solvent and time screening

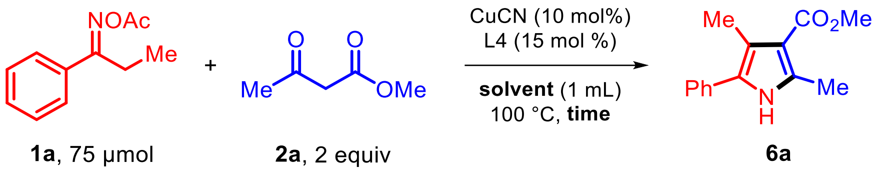

Entry	Solvent	Time	NMR Yield (%)
-	MeCN	24 h	33%
1	MeCN	48 h	<30%
2	MeCN	72 h	<30%
3	DCE	24 h	<30%
4	$PhCF_3$	24 h	<30%
5	AcOEt	24 h	<30%
6	THF	24 h	45%[a]
7	THF	48 h	47%
8	THF	48 h	41%[b]
9	$CHCl_3$	48 h	<33%
10	DMF	48 h	<33%
11	*t*BuOH	24 h	NR
12	Dioxane	24 h	34%
**13**	**THF**	**36 h**	**65%**

[a]23% of oxime **1a** was left.
[b]CuCN (7.5 mol%) and **L4** (15 mol%).

The reactions between oxime ester **1a** and active methylene compound **2a** at 24, 36, and 48 h in THF were carried out. At 24 h (entry 6), the expected pyrrole was formed in only 45%, whereas some starting material **1a** was left (23%). The optimal reaction time was 36 h since the limiting reagent was entirely consumed, and such an experiment afforded the highest NMR yield (65%, entry 13). Finally, after 48 h of heating, although the full conversion was reached, pyrrole **6a** was formed in only 47% (entry 7). It was noticed that the lower yield in the last experiment is due to partial decomposition of pyrrole occurring by heating for an extended time. In most Table 5.3 experiments, including entries 6, 7, and 13, the major side product corresponded to propiophenone **8**, and a small amount of the symmetric pyrrole **9** was equally observed. The formation of propiophenone might be a consequence of parasitic pathways which conduct organometallic intermediates **A** or **B**,[2] whereas the presence of **9** may indicate the participation of the α-iminyl radical **D** in this transformation.[3]

[2] a) Z.-H. Ren, Z.-Y. Zhang, B.-Q. Yang, Y.-Y. Wang, Z.-H. Guan, *Org.Lett.*, **2011**, *13*, 5394; b) Z.-H. Guan, Z.-Y. Zhang, Z.-H. Ren, Y.-Y. Wang, X. Zhang, *J. Org.Chem.*, **2011**, *76*, 339; c) Y. Wei, N. Yoshikai, *J. Am. Chem.Soc.*, **2013**, *135*, 3756.
[3] L. Ran, Z.-H. Ren, Y.-Y. Wang, Z.-H. Guan, *Green Chem.*, **2014**, *16*, 112.

5.4 Oxime activating group

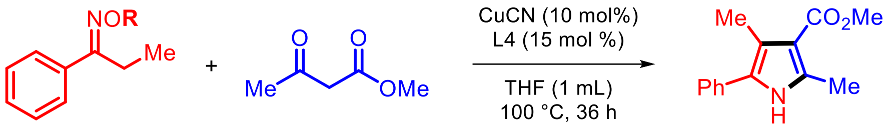

1a, 75 μmol **2a**, 2 equiv **6a**

Entry	R	NMR Yield (%)
**-**	**-Ac**	**65%**
1	-Piv	36%
2	-Bz	32%
3	-COC_6H_4-*p*CF_3	29%
4	-COC_6F_5	44%

5.5 Catalyst/Ligand effect screening

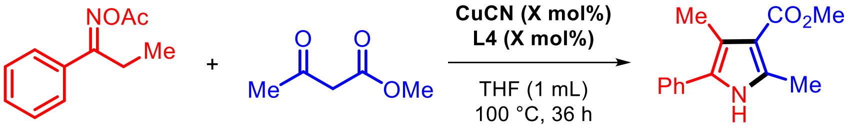

1a, 75 μmol **2a**, 2 equiv **6a**

Entry	CuCN (mol%)	L4 (mol%)	NMR Yield (%)
1	5	10	32%
2	7.5	15	41%
3	12.5	25	52%
4	15	30	52%
**-**	**10**	**15**	**65%**
5	12.5	17.5	49%

5.6 Re-screening of the copper source

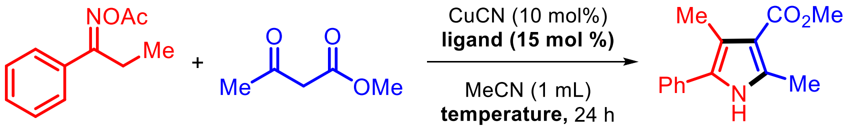

1a, 75 μmol **2a**, 2 equiv **6a**

Entry	Copper catalyst (10 mol%)	NMR Yield (%)
**-**	**CuCN**	**65%**
1	CuI	35%
2	CuBr	33%
3	CuCl	36%
4	$CuSO_4$	31%
5	$CuBr_2$	28%

5.7 Concentration effect screening

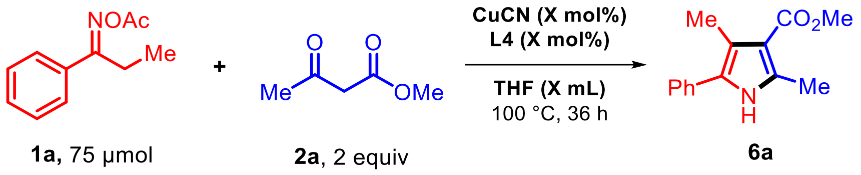

Entry	Volume THF (mL)	Concentration (mmol/mL)	[CuCN] mol%	L4 mol%	NMR Yield (%)
**-**	**1**	**0.075**	**10**	**15**	**65%**
1	1.5	0.05	10	15	47%
2	2	0.037	10	15	38%
3	1	0.075	12.5	17.5	49%
4	1.5	0.05	12.5	17.5	43%
5	2	0.037	12.5	17.5	34%

5.8 Use of a reducing agent as an additive

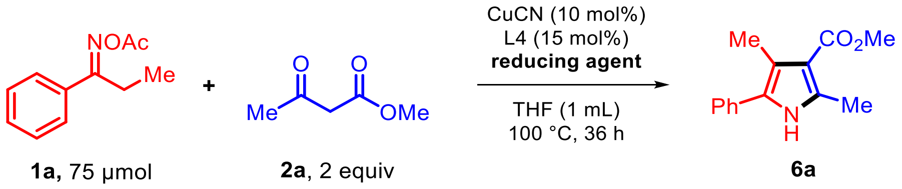

Entry	Reducing agent	Equivalents	NMR Yield (%)
-	-	-	**65%**
1	$NaHSO_3$	0.2-2	0%
2	Na_2SO_3	1	49%
3	HCOONa	1	37%
4	$Na_2S_2O_3$	1	30%

5.9 Use of an oxidizing agent as an additive

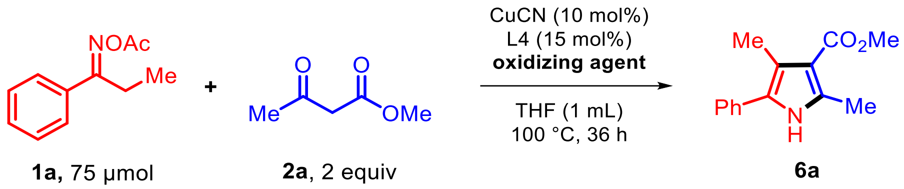

Entry	Oxidizing agent	Equivalents	NMR Yield (%)
-	-	-	**65%**
1	$Mn(OAc)_3·2(H_2O)$	0.05	43%
2	$Mn(OAc)_3·2(H_2O)$	0.1	48%
3	$Mn(OAc)_3·2(H_2O)$	0.25	47%
4	Cu(OAc)	0.1	25%
5	$Cu(OAc)_2$	0.1	34%
6	Potassium persulfate	1	0%
7	CAN	1	31%
8	Dicumyl peroxide	1	48%
9	*tert*-Butylperoxy benzoate	1	<10%
10	*tert*-Butyl hydroperoxide	1	<10%

5.10 Addition of molecular sieves

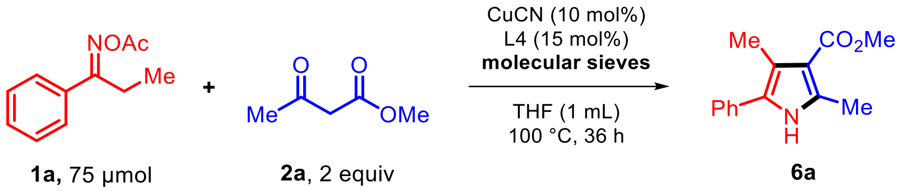

Entry	Sieve Size	Mass	NMR Yield (%)
-	-	-	**65%**
1	3 Å (beads)	20 mg	43%
2	3 Å (powder)	20 mg	41%
3	4 Å (beads)	20 mg	48%
4	4 Å (powder)	20 mg	48%
5	5 Å (beads)	20 mg	35%
6	5 Å (powder)	20 mg	42%

5.11 Addition of a base

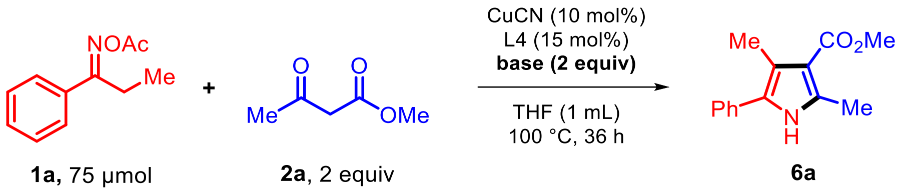

Entry	Base	NMR Yield (%)
-	-	**65%**
1	Na_2CO_3	39%
2	Cs_2CO_3	0%
3	K_2CO_3	45%
4	Li_2CO_3	44%
5	Et_3N	40%

5.12 Equiv of β-ketoester

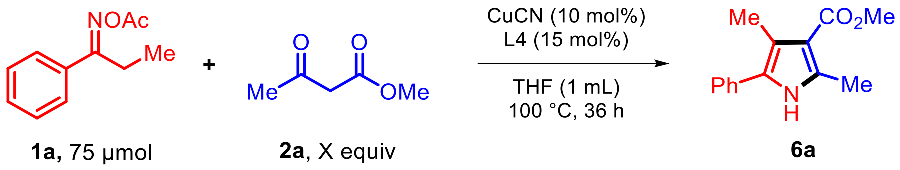

Entry	Equiv of 2a	NMR Yield (%)
-	**2**	**65%**
1	1.5	50%
2	1.1	27%

5.13 Isolated yield

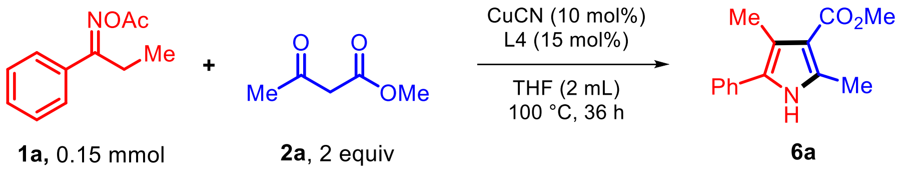

Entry	Modification	Isolated Yield (%)
**1**	-	**56%**
2	without Cu	NR

5.14 Equiv of β-ketonitrile

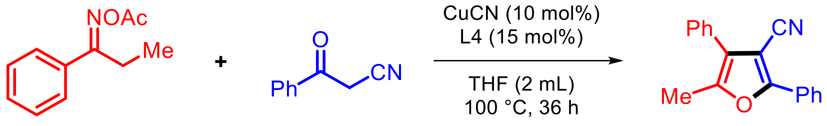

1a, 0.15 mmol **2b**, X equiv **10a**

Entry	Equiv of 2b	Isolated Yield (%)
1	2	45%
2	1.5	48%
**3**	**1.1**	**70%**

6. General procedure for the synthesis of pyrroles 6

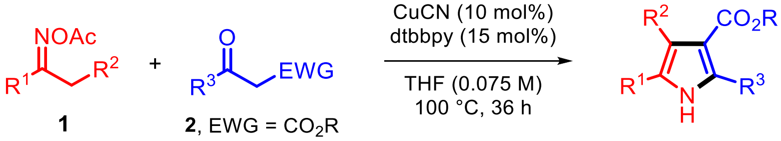

1 **2**, EWG = CO_2R **6**

To an oven-dried screw-capped tube with a Teflon-coated magnetic stir bar were added CuCN (10 mol%), 4,4′-di-*tert*-butyl-2,2′-dipyridyl (dtbbpy, **L4**) (15 mol%) and anhydrous THF (13.3 mL/mmol). The resulting mixture was stirred at room temperature for 30 minutes. Afterward, the corresponding *O*-acetyl oxime **1** (0.15 mmol) was added, and the reaction was purged under N_2. The β-ketoester **2** (2 equiv) was injected *via* syringe, and the reaction mixture was stirred at 100 °C for 36 h. The reaction was diluted with EtOAc and washed with 1 M HCl solution. The organic layer was dried over Na_2SO_4, concentrated *in vacuo*, and purified by column chromatography on silica gel to afford the desired pyrrole **6**.

Note: In the case of liquid *O*-acetyloximes, these substrates were weighed together with CuCN and dtbbpy.

7. General procedure for the synthesis of furans 10

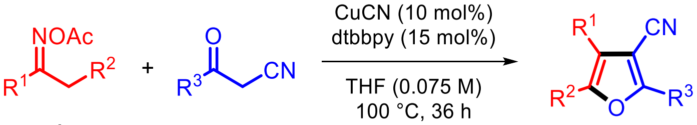

1 **2b-2bi** **10**

To an oven-dried screw-capped tube with a Teflon-coated magnetic stir bar were added CuCN (10 mol%), 4,4′-di-*tert*-butyl-2,2′-dipyridyl (dtbbpy, **L4**) (15 mol%), and anhydrous THF (13.3 mL/mmol). The resulting mixture was stirred at room temperature for 30 minutes. Afterward, the corresponding *O*-acetyl oxime **1** (0.15 mmol) and β-ketonitrile **2b-2bi** (1.1 equiv) were added, and the reaction was purged under N_2. The mixture was stirred at 100 °C for 36 h. The reaction was filtered through a silica plug, concentrated *in vacuo*, and purified by column chromatography on silica gel to afford the desired furan.

Note: In the case of liquid *O*-acetyloximes, these substrates were weighed together with CuCN and dtbbpy.

8. Synthesis of furan 10o on a larger scale

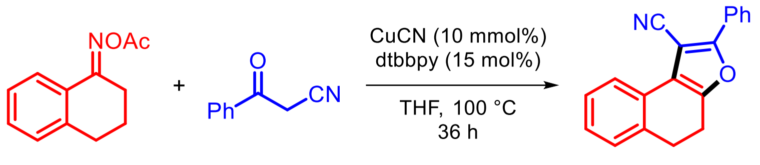

To an oven-dried screw-capped tube with a Teflon-coated magnetic stir bar were added CuCN (13.4 mg, 10 mol%), 4,4′-di-*tert*-butyl-2,2′-dipyridyl (dtbbpy, 60.4 mg, 15 mol%), and anhydrous THF (20 mL). The resulting mixture was stirred at room temperature for 30 minutes. Afterward, α-tetralone *O*-acetyl oxime **S1n** (305 mg, 1.5 mmol) and benzoylacetonitrile **2b** (240 mg, 1.65 mmol) were added, and the reaction was purged under N_2. The mixture was stirred at 100 °C in an oil bath for 36 h. The reaction was filtered through a silica plug, concentrated *in vacuo*, and purified by column chromatography on silica gel using as solvent mixture hexane:EtOAc (99:1 → 98:2) to afford the furan **10o** (311.6 mg, 77%) as a white solid.

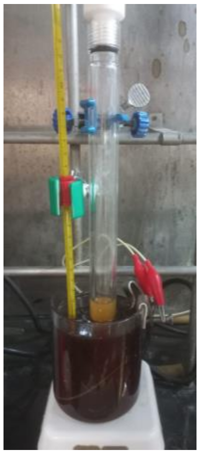

Supplementary Figure 1. Photography of the large-scale experiment

9. Derivatization of the products

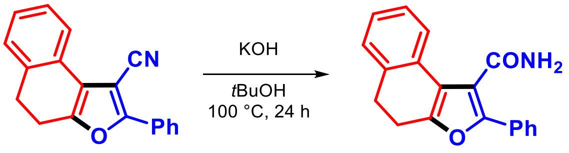

To an oven-dried screw-capped tube with a Teflon-coated magnetic stir bar were added KOH (0.66 mmol, 7.2 equiv) and *t*BuOH (1 mL). The mixture was stirred at 100 °C for 10 min and then cooled to room temperature. 2-Phenyl-4,5-dihydronaphtho[2,1-*b*]furan-1-carbonitrile **10o** (92 µmol, 1 equiv) was added. The mixture was stirred at 100 °C for 24 h. The reaction mixture was poured into water (10 mL) and extracted with EtOAc (3 x 10 mL). The organic phase was dried over anhydrous Na_2SO_4, filtered, and concentrated in vacuo. The reaction crude was purified by silica gel column chromatography using hexane:EtOAc (7:3) as solvent mixture to afford amide **13** as a white solid.

2-Phenyl-4,5-dihydronaphtho[2,1-*b*]furan-1-carboxamide (13). White solid (22.4 mg, 84% yield); mp 203-205 °C. **^{1}H NMR (300 MHz, $CDCl_3$) δ ppm** 7.80-7.76 (m, 2H), 7.65-7.62 (m, 1H), 7.44-7.38 (m, 2H),

7.36-7.30 (m, 1H), 7.27-7.10 (m, 3H), 6.06 (brs, 1H), 5.89 (brs, 1H), 3.12-3.07 (m, 2H), 2.96-2.90 (m, 2H); **^{13}C NMR (75 MHz, $CDCl_3$) δ ppm** 168.11, 153.36, 150.90, 133.80, 130.02, 129.83, 128.84, 128.55, 128.25, 127.21, 126.48, 126.26, 123.36, 118.44, 114.70, 29.66, 22.09. **HRMS (ESI+):** Calculated for $C_{19}H_{16}NO_2^+$: 290.11810; Found: 290.11858. **IR (ATR)** (υ_{max}, cm^{-1}) 3380 (w), 3187 (w), 1633 (m), 1599 (m), 1446 (w).

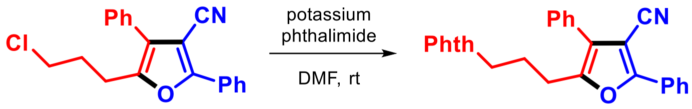

10m **14**, 73%

To an oven-dried screw-capped tube with a Teflon-coated magnetic stir bar were added 2-phenyl-4,5-dihydronaphthol[2,1-*b*]furan-1-carbonitrile **10m** (52.8 µmol, 1 equiv) and potassium phthalimide (89.8 µmol, 1.7 equiv) in anhydrous DMF (1 mL). The resulting mixture was stirred at 80 °C for 5 h. After cooling to room temperature, the reaction mixture was poured into water (10 mL) and extracted with EtOAc (3 x 10 mL). The organic phase was dried over anhydrous Na_2SO_4, filtered, and concentrated in vacuo. The resulting crude was purified by silica gel column chromatography using hexane:EtOAc (8:2) as solvent mixture to afford phthalimide **14** as a white solid.

5-(3-(1,3-Dioxoisoindolin-2-yl)propyl)-2,4-diphenylfuran-3-carbonitrile (14). Light yellow oil (16.7 mg, 73% yield). **^{1}H NMR (300 MHz, $CDCl_3$) δ ppm** 7.99-7.94 (m, 2H), 7.80-7.73 (m, 2H), 7.70-7.64 (m, 2H), 7.50-7.35(m, 8H), 3.78 (t, *J* = 7.0 Hz, 2H), 2.88 (t, *J* = 7.6 Hz, 2H), 2.18 (quint, *J* = 7.3 Hz, 2H); **^{13}C NMR (75 MHz, $CDCl_3$) δ ppm** 168.35, 158.56, 150.85, 134.13, 132.03, 130.04, 129.92, 129.13, 129.06, 128.82, 128.37, 128.17, 125.40, 124.02, 123.35, 115.12, 93.25, 37.64, 26.71, 24.24. **HRMS (ESI+):** Calculated for $C_{28}H_{21}N_2O_3^+$: 433.15522; Found: 433.15499. **IR (ATR)** (υ_{max}, cm^{-1}) 3195 (m), 2223 (w), 1773 (m), 1706 (s), 1306 (s).

10. Mechanistic investigations

10.1 Inhibition experiments with TEMPO and BHT

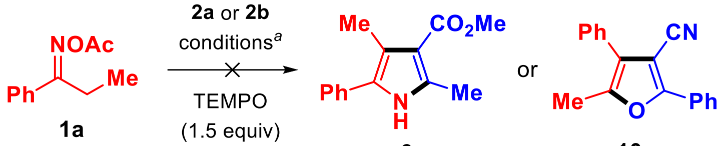

1a **6a** **10a**

Entry	Modification	Product
1	**2a** (2 equiv)	**6a** (0%)
2	**2b** (1.1 equiv)	**10a** (0%)

[a] CuCN (10 mol%), dtbbpy (15 mol%) in THF (0.075 M) at 100 °C for 36 h.

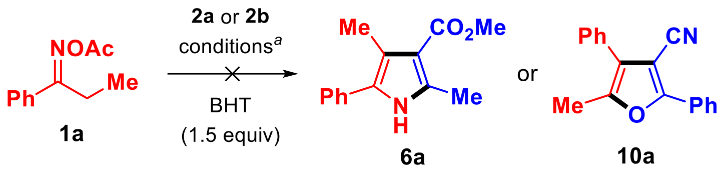

Entry	Modification	Product[b]
1	**2a** (2 equiv)	**6a** (7%)
2	**2b** (1.1 equiv)	**10a** (54%)

[a] CuCN (10 mol%), dtbbpy (15 mol%) in THF (0.075 M) at 100 °C for 36 h.
[b] NMR yield.

10.2 Experiment with oxime 1af

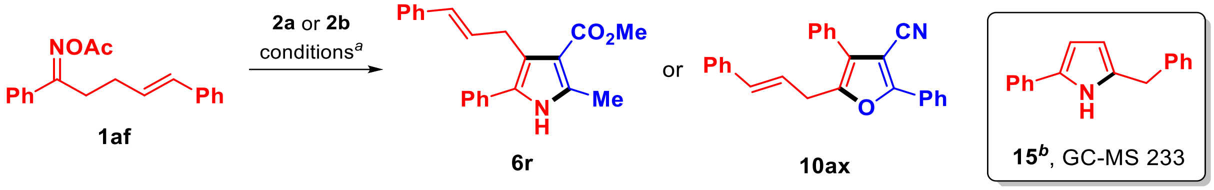

Entry	Modification	Product
1	**2a** (2 equiv)	**6r** (0%)
2	**2b** (1.1 equiv)	**10ax** (0%)

[a] CuCN (10 mol%), dtbbpy (15 mol%) in THF (0.075 M) at 100 °C for 36 h.
[b] Observed by GC-MS in both entries.

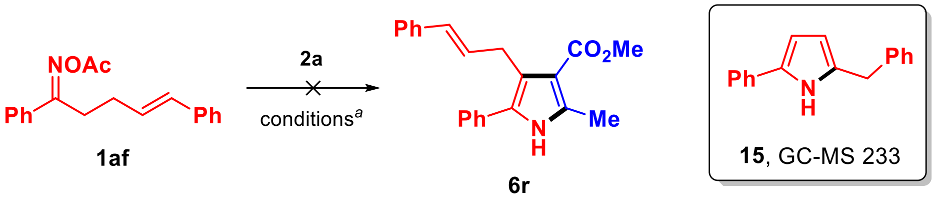

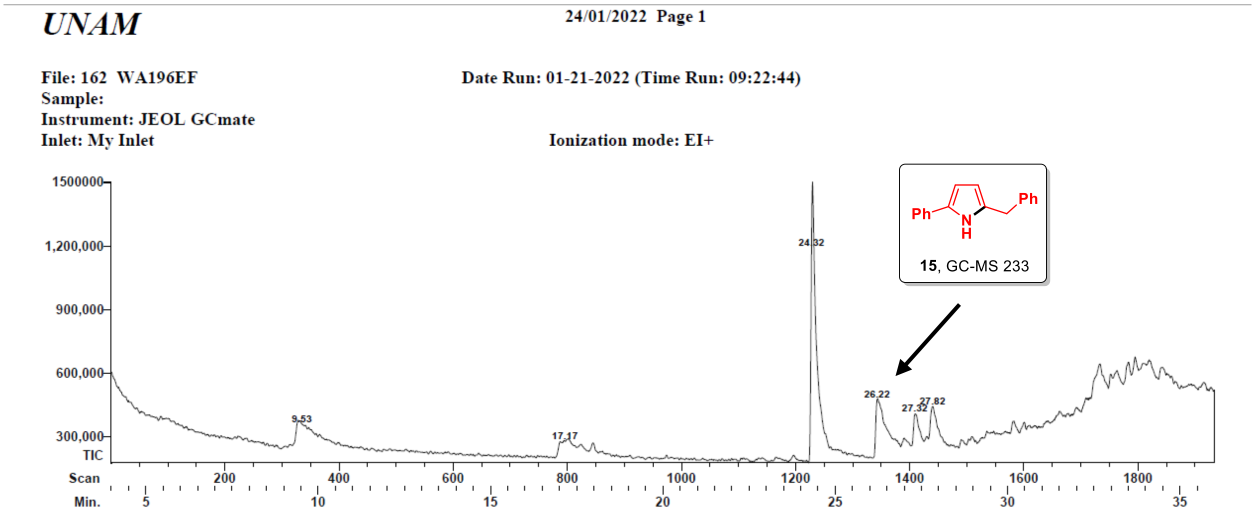

UNAM

24/01/2022 Page 6

File: 162 WA196EF Date Run: 01-21-2022 (Time Run: 09:22:44)
Sample:
Instrument: JEOL GCmate
Inlet: My Inlet Ionization mode: EI+

Scan: 1343 R.T.: 26.22
Base: m/z 40; 3.2%FS TIC: 476848 #Ions: 44

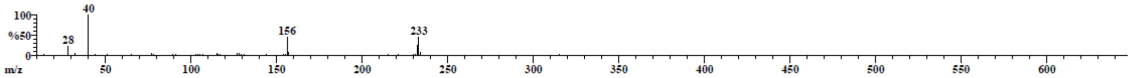

Threshold: 2% of Base Displayed TIC: 476848

Mass	%Base	Mass	%Base	Mass	%Base	Mass	%Base	Mass	%Base	Mass	%Base	Mass	%Base
14.3177	2.1	50.9938	3.3	89.0316	2.2	104.9885	2.1	127.9955	5.6	154.9730	2.8	220.9742	2.8
27.9898	22.0	65.0340	2.5	91.0293	4.1	115.0021	5.0	129.0259	2.8	156.0072	44.3	229.9938	4.1
31.9772	4.9	77.0358	6.3	102.9858	2.2	116.0146	2.7	144.0249	2.8	157.0251	7.3	230.9861	2.3
39.9363	100.0	78.0052	2.1	104.0088	3.7	126.9871	4.6	154.0005	3.1	214.9644	2.5	232.0280	26.1

Mass	%Base	Mass	%Base	Mass	%Base	Mass	%Base	Mass	%Base	Mass	%Base	Mass	%Base
233.0247	44.5	233.9760	8.4	314.9234	2.1								

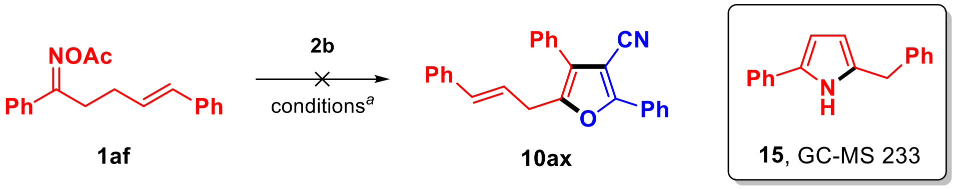

UNAM

24/01/2022 Page 1

File: 163 WA195EF Date Run: 01-21-2022 (Time Run: 10:09:16)
Sample:
Instrument: JEOL GCmate
Inlet: My Inlet Ionization mode: EI+

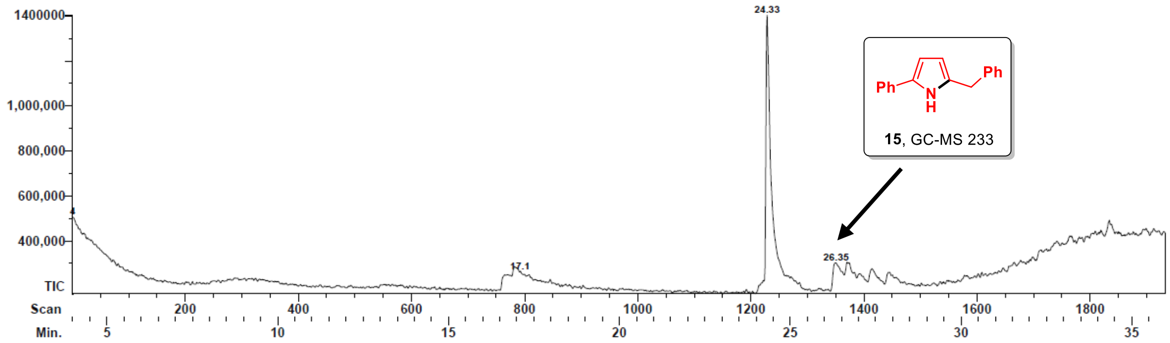

UNAM 24/01/2022 Page 6

File: 163 WA195EF Date Run: 01-21-2022 (Time Run: 10:09:16)
Sample:
Instrument: JEOL GCmate
Inlet: My Inlet Ionization mode: EI+

Scan: 1351 R.T.: 26.35
Base: m/z 40; 11.9%FS TIC: 308060 #Ions: 27

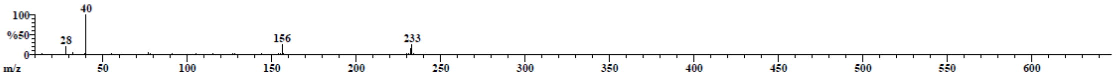

Threshold: 2% of Base Displayed TIC: 308060

Mass	%Base	Mass	%Base	Mass	%Base	Mass	%Base	Mass	%Base	Mass	%Base	Mass	%Base
14.3177	2.1	39.9363	100.0	91.0293	2.3	128.0132	3.4	154.9925	2.4	229.9702	2.9	233.0009	24.5
27.9898	21.6	77.0220	5.7	104.9724	4.4	144.0249	2.2	155.9876	24.1	231.0097	2.3	233.9998	4.4
31.9683	6.1	78.0052	2.1	115.0021	3.6	154.0199	2.3	156.9663	3.2	232.0043	14.9		

10.3 Competitive experiments with alkene 16

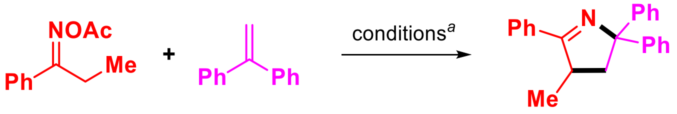

1a **16**, 1.5 equiv **17**

Entry	Modification	Products
1	-	**17** (32%)[b]
2	with **2a** (2 equiv)	**17** (0%), **6a** (17%)[c]
3	with **2b** (1.1 equiv)	**17** (0%), **10a** (67%)[c]

[a] CuCN (10 mol%), dtbbpy (15 mol%) in THF (0.075 M) at 100 °C for 36 h.
[b] Isolated yield.
[c] NMR yield.

The reaction between the radical species **F** and those derived from either **2a** or **2b** is faster than the radical addition to the alkene **16**. Although the yield of pyrrole **6a** is negatively impacted by the presence of **16**, it does not have any remarkable effect in the case of furan **10a**.

The isolation of compound **17** demonstrated the participation of the α-iminyl radical species **F** in the mechanism. The formation of the pyrroline might derive from either Cu-mediated radical oxidation of the adduct **A'** followed by cyclization or intramolecular radical addition to the iminyl moiety and oxidation of the intermediate **C'**.

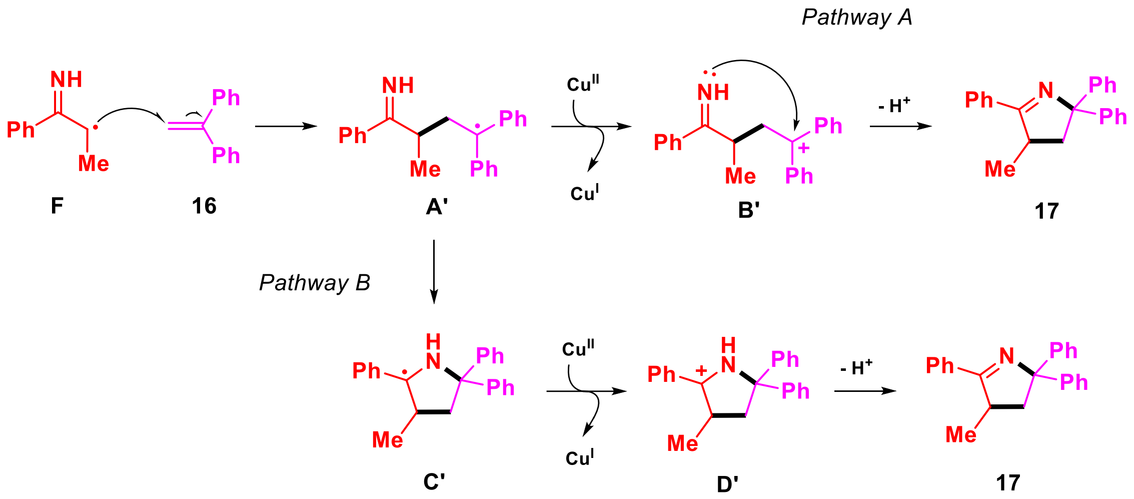

Supplementary Scheme 1. Proposed mechanistic pathways for the synthesis of pyrroline **17**

4-Methyl-2,2,5-triphenyl-3,4-dihydro-2*H*-pyrrole (17). Colorless oil (14.8 mg, 32%). **^{1}H NMR (300 MHz, $CDCl_3$) δ ppm** 7.89-7.86 (m, 2H), 7.55-7.50 (m, 2H), 7.40-7.37 (m, 5H), 7.29-7.07 (m, 6H), 3.54 (dqd, *J* = 8.7, 7.2, 5.1 Hz, 1H), 3.02 (dd, *J* = 12.9, 8.7 Hz, 1H), 2.46 (dd, *J* = 12.9, 5.1 Hz, 1H), 1.07 (d, *J* = 7.2 Hz, 3H); **^{13}C NMR (75 MHz, $CDCl_3$) δ ppm** 175.63, 149.16, 148.44, 134.32, 130.32, 128.57, 128.44, 128.29, 128.26, 126.75, 126.70, 126.38, 82.13, 46.56, 42.95, 18.64. **HRMS (ESI+):** Calculated for $C_{23}H_{22}N^{+}$: 312.17522; Found: 312.17501. **IR (ATR)** (υ_{max}, cm^{-1}) 2923 (w), 1614 (w), 1446 (m), 1328 (w).

10.4 Cyclization using alkene 12

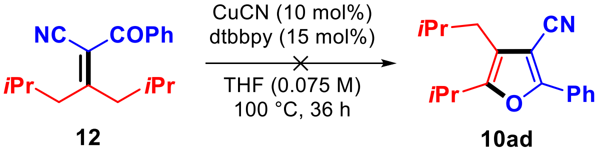

Alkene **12** was submitted to the standard conditions to prove its involvement as a reaction intermediate; however, the expected furan **10ad** was not observed. This ruled out its participation in the transformation.

10.5 Effect of $NaHSO_3$ in the reaction

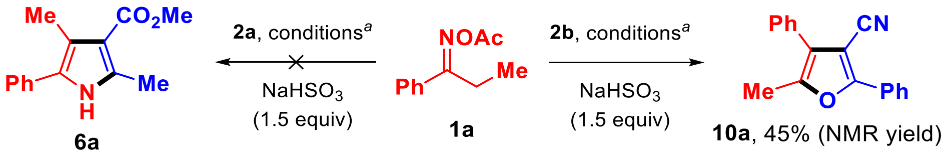

[a] CuCN (10 mol%), dtbbpy (15 mol%) in THF (0.075 M) at 100 °C for 36 h.

The use of $NaHSO_3$ inhibited the reaction of oxime **1a** with methyl acetoacetate (**2a**), whereas, in the transformation with benzoylacetonitrile (**2b**), the yield decreased slightly. It indicates that reactive species derived from **2a** are more sensitive than those formed from **2b,** which might explain the lower yields in the synthesis of pyrroles **6**.

11. Spectroscopic data of pyrroles 6a-6q

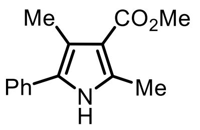

6a

Methyl 2,4-dimethyl-5-phenyl-1*H*-pyrrole-3-carboxylate. White solid (19.3 mg, 56% yield); mp 113-115 °C. **^{1}H NMR (300 MHz, $CDCl_3$) δ ppm** 8.11 (br s, 1H), 7.44-7.36 (m, 4H), 7.30-7.26 (m, 1H), 3.83 (s, 3H), 2.55 (s, 3H), 2.38 (s, 3H); **^{13}C NMR (75 MHz, $CDCl_3$) δ ppm** 166.85, 135.68, 132.90, 128.86, 127.46, 127.42, 126.79, 118.06, 112.39, 50.66, 14.24, 11.97. **HRMS (ESI+):** Calculated for $C_{14}H_{16}NO_2^+$: 230.11810; Found: 230.11765. **IR (ATR)** (υ_{max}, cm^{-1}) 3251 (w), 1661 (s), 1100 (s).

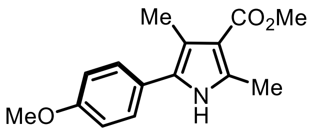

6b

Methyl 5-(4-methoxyphenyl)-2,4-dimethyl-1*H*-pyrrole-3-carboxylate. White solid (19.1 mg, 49% yield); mp 123-125 °C. **^{1}H NMR (300 MHz, $CDCl_3$) δ ppm** 7.97 (br s, 1H), 7.29 (d, *J* = 8.6 Hz, 2H), 6.95 (d, *J* = 8.6 Hz, 2H), 3.84 (s, 3H), 3.82 (s, 3H), 2.53 (s, 3H), 2.33 (s, 3H); **^{13}C NMR (75 MHz, $CDCl_3$) δ ppm** 166.89, 158.68, 135.15, 128.86, 127.29, 125.54, 117.17, 114.33, 112.14, 55.49, 50.63, 14.22, 11.89. **HRMS (ESI+):** Calculated for $C_{15}H_{18}NO_3^+$: 260.12867; Found: 260.12851. **IR (ATR)** (υ_{max}, cm^{-1}) 3361 (w), 1666 (m), 1177 (m), 1091 (m).

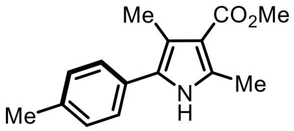

6c

Methyl 2,4-dimethyl-5-(*p*-tolyl)-1*H*-pyrrole-3-carboxylate. White solid (20.0 mg, 55% yield); mp 121-123 °C. **^{1}H NMR (300 MHz, $CDCl_3$) δ ppm** 7.98 (br s, 1H), 7.29-7.20 (m, 4H), 3.83 (s, 3H), 2.54 (s, 3H), 2.38 (s, 3H), 2.35 (s, 3H); **^{13}C NMR (75 MHz, $CDCl_3$) δ ppm** 166.85, 136.63, 135.36, 130.03, 129.58, 127.45, 127.36, 117.63, 112.31, 50.65, 21.32, 14.26, 11.95. **HRMS (ESI+):** Calculated for $C_{15}H_{18}NO_2^+$: 244.13375; Found: 244.13324. **IR (ATR)** (υ_{max}, cm^{-1}) 3303 (w), 1667 (m), 1161 (m).

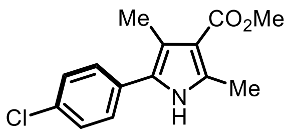

6d

Methyl 5-(4-chlorophenyl)-2,4-dimethyl-1*H*-pyrrole-3-carboxylate. White solid (17.0 mg, 43% yield); mp 139-142 °C. **^{1}H NMR (300 MHz, $CDCl_3$) δ ppm** 8.15 (br s, 1H), 7.41-7.27 (m, 4H), 3.82 (s, 3H), 2.53 (s, 3H), 2.34 (s, 3H); **^{13}C NMR (75 MHz, $CDCl_3$) δ ppm** 166.76, 136.02, 132.56, 131.34, 129.04, 128.59, 126.32, 118.56, 112.53, 50.72, 14.22, 11.93. **HRMS (ESI+):** Calculated for $C_{14}H_{14}ClNO_2$: 263.0713; Found: 263.0726. **IR (ATR)** (υ_{max}, cm^{-1}) 3291 (w), 2918 (w), 1653 (m), 1270 (m).

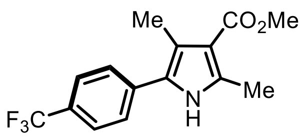

6e

Methyl 2,4-dimethyl-5-(4-(trifluoromethyl)phenyl)-1*H*-pyrrole-3-carboxylate. White solid (19.0 mg, 42% yield); mp 135-138 °C. **^{1}H NMR (300 MHz, $CDCl_3$) δ ppm** 8.12 (br s, 1H), 7.65 (d, *J* = 8.2 Hz, 2H), 7.48 (d, *J* = 8.1 Hz, 2H), 3.84 (s, 3H), 2.56 (s, 3H), 2.39 (s, 3H); **^{13}C NMR (75 MHz, $CDCl_3$) δ ppm**166.57, 136.62, 136.33, 128.52 (q, *J* = 32.6 Hz), 127.19, 126.03, 125.87 (q, *J* = 3.6 Hz), 124.33 (q, *J* = 270.2 Hz), 119.81, 112.95, 50.79, 14.31, 12.03. **HRMS (ESI+):** Calculated for $C_{15}H_{15}F_3NO_2^+$: 298.10549; Found: 298.10549. **IR (ATR)** (υ_{max}, cm^{-1}) 3326 (w), 1664 (m), 1106 (m).

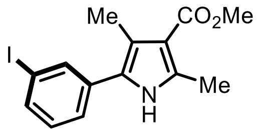

6f

Methyl 5-(3-iodophenyl)-2,4-dimethyl-1*H*-pyrrole-3-carboxylate. White solid (27.2 mg, 51% yield); mp 144-146 °C. **^{1}H NMR (300 MHz, CDCl$_3$) δ ppm** 8.16 (br s, 1H), 7.71 (t, *J* = 1.7 Hz, 1H), 7.59 (ddd, *J* = 7.8, 1.7, 1.0 Hz, 1H), 7.34 (ddd, *J* = 7.8, 1.7, 1.0 Hz, 1H), 7.12 (t, *J* = 7.8 Hz, 1H), 3.82 (s, 3H), 2.53 (s, 3H), 2.35 (s, 3H); **^{13}C NMR (75 MHz, CDCl$_3$) δ ppm** 166.68, 136.24, 135.99, 135.58, 135.01, 130.44, 126.62, 125.76, 119.03, 112.59, 94.78, 50.74, 14.25, 11.94. **HRMS (ESI+):** Calculated for $C_{14}H_{15}INO_2^+$: 356.01475; Found: 356.01370. **IR (ATR)** (υ_{max}, cm^{-1}) 3283 (w), 1657 (s), 1439 (m), 1251 (m).

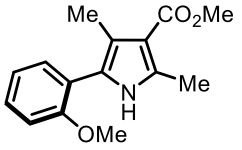

6g

Methyl 5-(2-methoxyphenyl)-2,4-dimethyl-1*H*-pyrrole-3-carboxylate. White solid (16.0 mg, 41% yield); mp 104-106 °C. **^{1}H NMR (300 MHz, CDCl$_3$) δ ppm** 8.62 (br s, 1H), 7.36 (dd, *J* = 7.6, 1.6 Hz, 1H), 7.29-7.23 (m, 1H), 7.04-6.96 (m, 2H), 3.86 (s, 3H), 3.82 (s, 3H), 2.54 (s, 3H), 2.35 (s, 3H); **^{13}C NMR (75 MHz, CDCl$_3$) δ ppm** 166.91, 156.46, 135.01, 130.72, 128.19, 123.93, 121.11, 121.03, 119.29, 111.53, 111.37, 55.78, 50.57, 14.44, 12.50. **HRMS (ESI+):** Calculated for $C_{15}H_{18}NO_3^+$: 260.12867; Found: 260.12869. **IR (ATR)** (υ_{max}, cm^{-1}) 3299 (w), 1664 (s), 1260 (s).

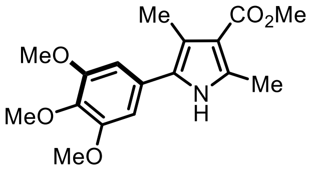

6h

Methyl 2,4-dimethyl-5-(3,4,5-trimethoxyphenyl)-1*H*-pyrrole-3-carboxylate. Colorless oil (20.1 mg, 42% yield). **^{1}H NMR (400 MHz, CDCl$_3$) δ ppm** 8.11 (br s, 1H), 6.55 (s, 2H), 3.87 (s, 9H), 3.83 (s, 3H), 2.55 (s, 3H), 2.37 (s, 3H); **^{13}C NMR (100 MHz, CDCl$_3$) δ ppm** 166.78, 153.55, 137.19, 135.45, 128.60, 127.51, 117.79, 107.73, 104.97, 61.10, 56.33, 50.70, 14.22, 11.97. **HRMS (ESI+):** Calculated for $C_{17}H_{22}NO_5^+$: 320.14980; Found: 320.14855. **IR (ATR)** (υ_{max}, cm^{-1}) 3321 (w), 1669 (m), 1238 (s).

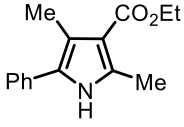

6i

Ethyl 2,4-dimethyl-5-phenyl-1*H*-pyrrole-3-carboxylate.[4] Yellow oil (18.2 mg, 50% yield). **^{1}H NMR (300 MHz, CDCl$_3$) δ ppm** 8.11 (br s, 1H), 7.44-7.36 (m, 4H), 7.31-7.24 (m, 1H), 4.30 (q, *J* = 7.1 Hz, 2H), 2.55 (s, 3H), 2.38 (s, 3H), 1.37 (t, *J* = 7.1 Hz, 3H).

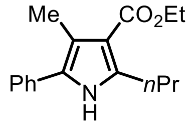

6j

Ethyl 4-methyl-5-phenyl-2-propyl-1*H*-pyrrole-3-carboxylate. White solid (16.7 mg, 41% yield); mp 94-95 °C. **^{1}H NMR (300 MHz, CDCl$_3$) δ ppm** 8.08 (br s, 1H), 7.44-7.36 (m, 4H), 7.30-7.27 (m, 1H), 4.30 (q, *J* = 7.1 Hz, 2H), 3.04-2.86 (m, 2H), 2.38 (s, 3H), 1.70 (sext, *J* = 7.4 Hz, 2H), 1.37 (t, *J* = 7.1 Hz, 3H), 0.99 (t, *J* = 7.4 Hz, 3H); **^{13}C NMR (75 MHz, CDCl$_3$) δ ppm** 166.24, 140.03, 133.17, 128.88, 127.56, 127.51, 126.79, 118.20, 112.28, 59.28, 30.24, 23.02, 14.64, 14.12, 11.99. **HRMS**

[4] Y.-F. Wang, K. K. Toh, S. Chiba, K. Narasaka, *Org. Lett.* **2008**, *10*, 5019.

(ESI+): Calculated for $C_{17}H_{22}NO_2^+$: 272.16505; Found: 272.16495. **IR (ATR)** (υ_{max}, cm^{-1}) 3307 (w), 1658 (s), 1254 (s).

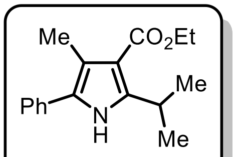

6k

Ethyl 2-isopropyl-4-methyl-5-phenyl-1*H*-pyrrole-3-carboxylate. White solid (7.0 mg, 17% yield); mp 117-119 °C. **^{1}H NMR (300 MHz, $CDCl_3$) δ ppm** 8.08 (br s, 1H), 7.45-7.37 (m, 4H), 7.32-7.27 (m, 1H), 4.31 (q, *J* = 7.1 Hz, 2H), 3.83 (sept, *J* = 7.0 Hz, 1H), 2.37 (s, 3H), 1.37 (t, *J* = 7.1 Hz, 3H), 1.31 (d, *J* = 7.0 Hz, 6H); **^{13}C NMR (75 MHz, $CDCl_3$) δ ppm** 166.20, 145.24, 133.12, 128.89, 127.61, 127.31, 126.86, 117.98, 111.24, 59.35, 26.43, 22.22, 14.62, 12.04. **HRMS (ESI+):** Calculated for $C_{17}H_{22}NO_2^+$: 272.16505; Found: 272.16437. **IR (ATR)** (υ_{max}, cm^{-1}) 3273 (w), 1657 (s), 1249 (s).

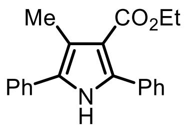

6l

Ethyl 4-methyl-2,5-diphenyl-1*H*-pyrrole-3-carboxylate.[5] Yellow oil (14.2 mg, 31% yield). **^{1}H NMR (300 MHz, $CDCl_3$) δ ppm** 8.27 (br s, 1H), 7.55-7.52 (m, 2H), 7.45-7.29 (m, 8H), 4.21 (q, *J* = 7.1 Hz, 2H), 2.44 (s, 3H), 1.20 (t, *J* = 7.2 Hz, 3H).

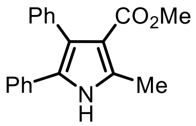

6m

Methyl 2-methyl-4,5-diphenyl-1*H*-pyrrole-3-carboxylate.[6] White solid (17.5 mg, 40% yield); mp 130-131 °C. **^{1}H NMR (300 MHz, $CDCl_3$) δ ppm** 8.23(br s, 1H), 7.33-7.01 (m, 10H), 3.62 (s, 3H), 2.62 (s, 3H).

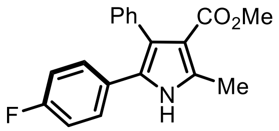

6n

Methyl 5-(4-fluorophenyl)-2,4-dimethyl-1*H*-pyrrole-3-carboxylate. Yellow oil (14.8 mg, 32% yield). **^{1}H NMR (300 MHz, $CDCl_3$) δ ppm** 8.20 (br s, 1H), 7.32-7.25 (m, 2H), 7.20-7.08 (m, 3H), 7.03-6.93 (m, 2H), 6.93-6.87 (m, 2H), 3.62 (s, 3H), 2.61 (s, 3H); **^{13}C NMR (75 MHz, $CDCl_3$) δ ppm** 166.04, 161.75 (d, *J* = 243.9 Hz), 135.75, 135.54, 130.84, 128.84, 128.73, 128.49, 128.45, 127.84, 126.99, 126.74, 126.64, 123.53, 115.68 (d, *J* = 21.6 Hz), 112.46, 50.69, 14.11. **HRMS (ESI+):** Calculated for $C_{19}H_{17}FNO_2^+$: 310.12433; Found: 310.12438. **IR (ATR)** (υ_{max}, cm^{-1}) 3301 (w), 1676 (s), 1448 (m).

---

[5] N. N. K. Reddy, D. Rawat, S. Adimurthy, *J. Org. Chem.* **2018**, *83*, 9412.
[6] F. Tamaddon, M. Farahi, *Synlett* **2012**, *23*, 1379.

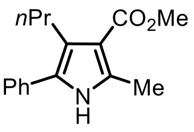

6o

Methyl 2-methyl-5-phenyl-4-propyl-1*H*-pyrrole-3-carboxylate. White solid (21.2 mg, 55% yield); mp 111-113 °C. **^{1}H NMR (300 MHz, CDCl$_3$) δ ppm** 8.06 (br s, 1H), 7.44-7.34 (m, 4H), 7.32-7.26 (m, 1H), 3.82 (s, 3H), 2.76-2.71 (m, 2H), 2.54 (s, 3H), 1.68-1.56 (m, 2H), 0.94 (t, *J* = 7.2 Hz, 3H); **^{13}C NMR (75 MHz, CDCl$_3$) δ ppm** 166.59, 135.81, 133.11, 128.89, 127.53, 127.43, 126.94, 123.54, 111.63, 50.63, 27.81, 25.19, 14.54, 14.37. **HRMS (ESI+):** Calculated for $C_{16}H_{20}NO_2^+$: 258.14940; Found: 258.14880. **IR (ATR)** (υ_{max}, cm^{-1}) 3295 (w), 1660 (m), 1166 (m).

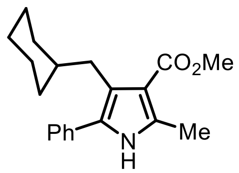

6p

Methyl 4-(cyclohexylmethyl)-2-methyl-5-phenyl-1*H*-pyrrole-3-carboxylate. White solid (22.4 mg, 48% yield); mp 129-131 °C. **^{1}H NMR (300 MHz, CDCl$_3$) δ ppm** 7.99 (br s, 1H), 7.43-7.36 (m, 4H), 7.33-7.26 (m, 1H), 3.82 (s, 3H), 2.72 (d, *J* = 6.9 Hz, 2H), 2.53 (s, 3H), 1.60-1.39 (m, 6H), 1.16-1.01 (m, 3H), 0.83-0.70 (m, 2H); **^{13}C NMR (75 MHz, CDCl$_3$) δ ppm** 166.78, 135.62, 133.54, 128.80, 128.22, 128.11, 126.97, 121.83, 111.86, 50.58, 40.03, 33.42, 32.68, 26.76, 26.60, 14.36. **HRMS (ESI+):** Calculated for $C_{20}H_{26}NO_2^+$: 312.19635; Found: 312.19714. **IR (ATR)** (υ_{max}, cm^{-1}) 3312 (w), 2916 (w), 1655 (m), 1094 (m).

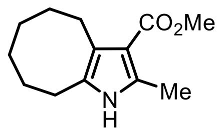

6q

Methyl 2-methyl-4,5,6,7,8,9-hexahydro-1*H*-cycloocta[*b*]pyrrole-3-carboxylate. White solid (11.3 mg, 34% yield); mp 146-149 °C. **^{1}H NMR (300 MHz, CDCl$_3$) δ ppm** 7.67 (br s, 1H), 3.78 (s, 3H), 2.82-2.78 (m, 2H), 2.61-2.57 (m, 2H), 2.46 (s, 3H), 1.68-1.54 (m, 4H), 1.48-1.41 (m, 2H), 1.36-1.27 (m, 2H); **^{13}C NMR (75 MHz, CDCl$_3$) δ ppm** 166.94, 133.23, 127.73, 120.62, 110.11, 50.43, 30.90, 29.90, 26.14, 25.96, 25.84, 23.08, 14.11. **HRMS (ESI+):** Calculated for $C_{13}H_{20}NO_2^+$: 222.14940; Found: 222.14910. **IR (ATR)** (υ_{max}, cm^{-1}) 3280 (w), 2924 (m), 1660 (m), 1268 (m).

12. Spectroscopic data of furans 10a-10aw

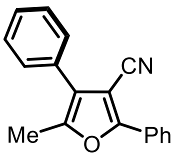

10a

5-Methyl-2,4-diphenylfuran-3-carbonitrile. White solid (27.1 mg, 70%); mp 83-85 °C. **^{1}H NMR (300 MHz, CDCl$_3$) δ ppm** 8.05-8.01 (m, 2H), 7.52-7.36 (m, 8H), 2.47 (s, 3H); **^{13}C NMR (75 MHz, CDCl$_3$) δ ppm** 158.39, 148.66, 130.35, 129.93, 129.16, 129.03, 128.69, 128.41, 128.14, 125.35, 123.54, 115.37, 93.19, 12.60. **HRMS (ESI+):** Calculated for $C_{18}H_{14}NO^+$: 260.10754; Found: 260.10739. **IR (ATR)** (υ_{max}, cm^{-1}) 3055 (w), 2917 (w), 2221 (m), 1556 (m), 1496 (w), 1447 (m), 1146 (w).

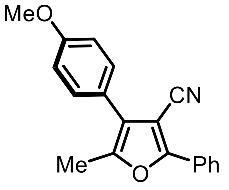

10b

4-(4-Methoxyphenyl)-5-methyl-2-phenylfuran-3-carbonitrile. White solid (18.7 mg, 43%); mp 162-164 °C. **^{1}H NMR (300 MHz, CDCl$_3$) δ ppm** 8.03-7.99 (m, 2H), 7.51-7.38 (m, 5H), 7.01 (d, *J* = 8.7 Hz, 2H), 3.86 (s, 3H), 2.45 (s, 3H); **^{13}C NMR (75 MHz, CDCl$_3$) δ ppm** 159.54, 158.17, 148.19, 129.91, 129.86, 129.15, 128.49, 125.31, 123.25, 122.63, 115.54, 114.52, 93.33, 55.49, 12.57. **HRMS (ESI+):** Calculated for $C_{19}H_{16}NO_2^+$: 290.11810; Found: 290.11803. **IR (ATR)** (υ_{max}, cm^{-1}) 3054 (w), 2917 (w), 2223 (w), 1514 (m), 1146 (w).

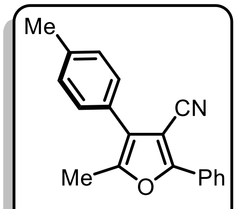

10c

5-Methyl-2-phenyl-4-(*p*-tolyl)furan-3-carbonitrile. White solid (22.9 mg, 56%); mp 133-135 °C. **^{1}H NMR (300 MHz, $CDCl_3$) δ ppm** 8.03-7.99 (m, 2H), 7.51-7.35 (m, 5H), 7.30-7.27 (m, 2H), 2.45 (s, 3H), 2.41 (s, 3H); **^{13}C NMR (75 MHz, $CDCl_3$) δ ppm** 158.26, 148.42, 138.03, 129.87, 129.74, 129.15, 128.56, 128.48, 127.38, 125.34, 123.52, 115.47, 93.31, 21.41, 12.61. **HRMS (ESI+):** Calculated for $C_{19}H_{16}NO^+$: 274.12319; Found: 274.12205. **IR (ATR)** (υ_{max}, cm^{-1}) 3034 (w), 2954 (w), 2222 (w), 1513 (w), 1145 (w).

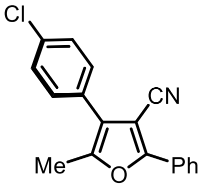

10d

4-(4-Chlorophenyl)-5-methyl-2-phenylfuran-3-carbonitrile. White solid (26.5 mg, 60%); mp 129-131 °C. **^{1}H NMR (300 MHz, $CDCl_3$) δ ppm** 8.03-7.99 (m, 2H), 7.52-7.38 (m, 7H), 2.45 (s, 3H); **^{13}C NMR (75 MHz, $CDCl_3$) δ ppm** 158.57, 148.79, 134.24, 130.10, 129.96, 129.31, 129.19, 128.80, 128.21, 125.36, 122.51, 115.14, 92.95, 12.59. **HRMS (ESI+):** Calculated for $C_{18}H_{13}ClNO^+$: 294.06857; Found: 294.06810. **IR (ATR)** (υ_{max}, cm^{-1}) 3070 (w), 2917 (w), 2224 (w), 1557 (m), 1092 (m).

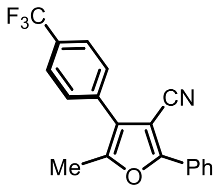

10e

5-Methyl-2-phenyl-4-(4-(trifluoromethyl)phenyl)furan-3-carbonitrile. White solid (31.0 mg, 63%); mp 102-103 °C. **^{1}H NMR (300 MHz, $CDCl_3$) δ ppm** 8.04-8.00 (m, 2H), 7.75 (d, J = 8.1 Hz, 2H), 7.60 (d, J = 8.1 Hz, 2H), 7.53-7.42 (m, 3H), 2.49 (s, 3H); **^{13}C NMR (75 MHz, $CDCl_3$) δ ppm** 158.88, 149.35, 134.07, 130.27, 130.25 (q, J = 32.3 Hz), 129.24, 128.99, 128.08, 126.05 (q, J = 3.8 Hz), 125.43, 124.16 (q, J = 270.0 Hz), 122.38, 115.01, 92.81, 12.65. **HRMS (ESI+):** Calculated for $C_{19}H_{13}F_3NO^+$: 328.09492; Found: 328.09414. **IR (ATR)** (υ_{max}, cm^{-1}) 3078 (w), 2925 (w), 2223 (w), 1619 (w), 1323 (m), 1163 (m).

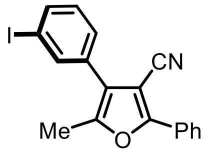

10f

4-(3-Iodophenyl)-5-methyl-2-phenylfuran-3-carbonitrile. White solid (27.7 mg, 48%); mp 105-107 °C. **^{1}H NMR (300 MHz, $CDCl_3$) δ ppm** 8.03-7.99 (m, 2H), 7.79 (t, J = 1.8 Hz, 1H), 7.73 (ddd, J = 8.1, 1.8, 1.2 Hz, 1H), 7.52-7.40 (m, 4H), 7.22 (t, J = 7.8 Hz, 1H), 2.46 (s, 3H); **^{13}C NMR (75 MHz, $CDCl_3$) δ ppm** 158.61, 149.05, 137.43, 137.19, 132.49, 130.69, 130.14, 129.20, 128.16, 127.99, 125.38, 122.07, 114.99, 94.70, 92.89, 12.60. **HRMS (ESI+):** Calculated for $C_{18}H_{12}NOI^+$: 384.9982; Found: 384.9964. **IR (ATR)** (υ_{max}, cm^{-1}) 2920 (w), 2218 (m), 1554 (m), 1491 (w), 1203 (w).

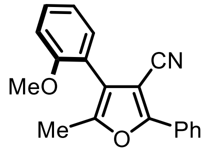

10g

4-(2-Methoxyphenyl)-5-methyl-2-phenylfuran-3-carbonitrile. Colorless oil (13.0 mg, 30%). **^{1}H NMR (300 MHz, $CDCl_3$) δ ppm** 8.04-8.00 (m, 2H), 7.50-7.36 (m, 4H), 7.27 (dd, J = 7.5, 1.8 Hz, 1H), 7.07-7.01 (m, 2H), 3.90 (s, 3H), 2.36 (s, 3H); **^{13}C NMR (75 MHz, $CDCl_3$) δ ppm** 157.83, 157.19, 149.42, 131.20, 130.07, 129.66, 129.08, 128.69, 125.30, 120.90, 120.30, 119.08, 115.46, 111.43, 94.79, 55.59, 12.63. **HRMS (ESI+):** Calculated for $C_{19}H_{16}NO_2^+$: 290.11810; Found: 290.11731. **IR (ATR)** (υ_{max}, cm^{-1}) 3062 (w), 2916 (w), 2224 (w), 1557 (w), 1250 (w).

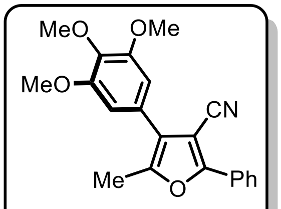

10h

5-Methyl-2-phenyl-4-(3,4,5-trimethoxyphenyl)furan-3-carbonitrile. White solid (26.1 mg, 50%); mp 154-155 °C. **^{1}H NMR (300 MHz, CDCl$_3$) δ ppm** 8.03-8.00 (m, 2H), 7.52-7.40 (m, 3H), 6.68 (s, 2H), 3.92 (s, 6H), 3.91 (s, 3H), 2.49 (s, 3H); **^{13}C NMR (75 MHz, CDCl$_3$) δ ppm** 158.36, 153.63, 148.44, 138.08, 130.00, 129.17, 128.33, 125.76, 125.34, 123.67, 115.43, 106.02, 93.19, 61.07, 56.41, 12.71. **HRMS (ESI+):** Calculated for $C_{21}H_{20}NO_4^+$: 350.13923; Found: 350.13806. **IR (ATR)** (υ_{max}, cm^{-1}) 2998 (w), 2924 (w), 2220 (w), 1578 (w), 1124 (m).

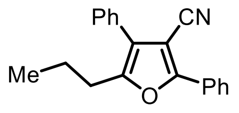

10i

2,4-Diphenyl-5-propylfuran-3-carbonitrile. White solid (24.4 mg, 56%); mp < 30 °C. **^{1}H NMR (300 MHz, CDCl$_3$) δ ppm** 8.05-8.01 (m, 2H), 7.52-7.37 (m, 8H), 2.78-2.73 (m, 2H), 1.78 (sext, J = 7.5 Hz, 2H), 0.99 (t, J = 7.2 Hz, 3H); **^{13}C NMR (75 MHz, CDCl$_3$) δ ppm** 158.33, 152.63, 130.40, 129.92, 129.16, 129.01, 128.87, 128.51, 128.19, 125.37, 123.65, 115.39, 93.27, 28.43, 21.86, 13.91. **HRMS (ESI+):** Calculated for $C_{20}H_{18}NO^+$: 288.13884; Found: 288.13787. **IR (ATR)** (υ_{max}, cm^{-1}) 2961 (w), 2222 (w), (w), 1556 (w), 1145 (w).

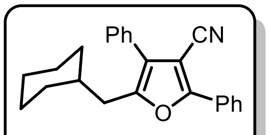

10j

5-(Cyclohexylmethyl)-2,4-diphenylfuran-3-carbonitrile. White solid (27.0 mg, 53%); mp 94-95 °C. **^{1}H NMR (300 MHz, CDCl$_3$) δ ppm** 8.05-8.02 (m, 2H), 7.53-7.36 (m, 8H), 2.65 (d, J = 6.6 Hz, 2H), 1.84-1.63 (m, 6H), 1.31-1.11 (m, 3H), 1.02-0.89 (m, 2H); **^{13}C NMR (75 MHz, CDCl$_3$) δ ppm** 158.27, 151.92, 130.45, 129.89, 129.16, 128.97, 128.54, 128.16, 125.38, 124.40, 115.38, 93.35, 37.69, 34.02, 33.23, 26.34, 26.25. **HRMS (ESI+):** Calculated for $C_{24}H_{24}NO^+$: 342.18579; Found: 342.18549. **IR (ATR)** (υ_{max}, cm^{-1}) 2916 (w), 2220 (w), 1555 (w), 1144 (w).

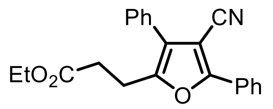

10k

Ethyl 3-(4-cyano-3,5-diphenylfuran-2-yl)propanoate. Colorless oil (30.6 mg, 59%). **^{1}H NMR (300 MHz, CDCl$_3$) δ ppm** 8.03-7.99 (m, 2H), 7.52-7.38 (m, 8H), 4.14 (q, J = 7.2 Hz, 2H), 3.14 (dd, J = 8.1, 7.2 Hz, 2H), 2.75 (dd, J = 8.1, 7.2 Hz, 2H), 1.23 (t, J = 7.2 Hz, 3H); **^{13}C NMR (75 MHz, CDCl$_3$) δ ppm** 172.04, 158.68, 150.14, 130.13, 129.85, 129.19, 129.12, 128.81, 128.43, 128.26, 125.41, 124.22, 115.10, 93.47, 60.94, 32.55, 22.13, 14.29. **HRMS (ESI+):** Calculated for $C_{22}H_{20}NO_3^+$: 346.14432; Found: 346.14395. **IR (ATR)** (υ_{max}, cm^{-1}) 3058 (w), 2962 (w), 2223 (w), 1493 (w), 1181 (w).

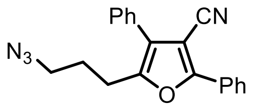

10l

5-(3-Azidopropyl)-2,4-diphenylfuran-3-carbonitrile. Colorless oil (18.7 mg, 38%). **^{1}H NMR (300 MHz, CDCl$_3$) δ ppm** 8.04-8.00 (m, 2H), 7.53-7.38 (m, 8H), 3.36 (t, J = 6.9 Hz, 2H), 2.89 (t, J = 7.2 Hz, 2H), 2.01 (quint, J = 6.6 Hz, 2H); **^{13}C NMR (75 MHz, CDCl$_3$) δ ppm** 158.70, 150.82, 130.16, 129.90, 129.23, 129.16, 128.79, 128.47, 128.24, 125.42, 124.33, 115.12, 93.44, 50.65, 27.65, 23.71. **HRMS (ESI+):** Calculated for $C_{20}H_{17}N_4O^+$: 329.14024; Found: 329.13946. **IR (ATR)** (υ_{max}, cm^{-1}) 3057 (w), 2923 (w), 2223 (w), 1556 (w), 1145 (w).

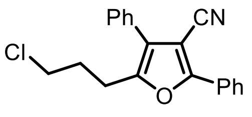

10m

5-(3-Chloropropyl)-2,4-diphenylfuran-3-carbonitrile. White solid (27.0 mg, 56%); mp 72-74 °C. **^{1}H NMR (300 MHz, $CDCl_3$) δ ppm** 8.04-8.00 (m, 2H), 7.52-7.37 (m, 8H), 3.59 (t, *J* = 6.3 Hz, 2H), 2.97 (t, *J* = 8.1, 6.6 Hz, 2H), 2.25-2.16 (m, 2H); **^{13}C NMR (75 MHz, $CDCl_3$) δ ppm** 158.69, 150.73, 130.15, 129.90, 129.22, 129.13, 128.81, 128.44, 128.25, 125.40, 124.40, 115.13, 93.43, 44.00, 31.11, 23.89. **HRMS (ESI+):** Calculated for $C_{20}H_{17}NOCl^+$: 322.09987; Found: 322.10034. **IR (ATR)** (υ_{max}, cm^{-1}) 3057 (w), 2959 (w), 2223 (w), 1954 (w), 1556 (w), 1146 (w).

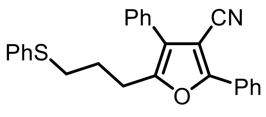

10n

2,4-Diphenyl-5-(3-(phenylthio)propyl)furan-3-carbonitrile. Yellow oil (19.3 mg, 32%). **^{1}H NMR (300 MHz, $CDCl_3$) δ ppm** 8.01-7.97 (m, 2H), 7.52-7.37 (m, 8H), 7.32-7.15 (m, 5H), 2.98-2.92 (m, 4H), 2.06 (quint, *J* = 7.2 Hz, 2H); **^{13}C NMR (75 MHz, $CDCl_3$) δ ppm** 158.56, 151.34, 135.92, 130.07, 130.03, 129.71, 129.18, 129.09, 128.82, 128.35, 128.29, 126.37, 125.39, 124.14, 115.20, 93.34, 33.23, 27.69, 25.37. **HRMS (ESI+):** Calculated for $C_{26}H_{22}NOS^+$: 396.14221; Found: 396.14031. **IR (ATR)** (υ_{max}, cm^{-1}) 2931 (w), 2223 (w), 1493 (w), 1447 (w).

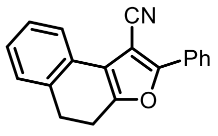

10o

2-Phenyl-4,5-dihydronaphtho[2,1-*b*]furan-1-carbonitrile. Light yellow solid (32.6 mg, 80%); mp 118-119 °C. **^{1}H NMR (300 MHz, $CDCl_3$) δ ppm** 8.03 (d, *J* = 6.9 Hz, 2H), 7.91 (d, *J* = 7.5 Hz, 1H), 7.52-7.40 (m, 3H), 7.33-7.18 (m, 3H), 3.15 (t, *J* = 8.4 Hz, 2H), 3.02-2.97 (m, 2H); **^{13}C NMR (75 MHz, $CDCl_3$) δ ppm** 159.61, 153.03, 133.32, 130.00, 129.19, 128.80, 128.48, 128.36, 127.46, 125.36, 122.41, 119.65, 115.89, 88.97, 29.22, 21.86. **HRMS (ESI+):** Calculated for $C_{19}H_{14}NO^+$: 272.10754; Found: 272.10699. **IR (ATR)** (υ_{max}, cm^{-1}) 3047 (w), 2902 (w), 2218 (w), 1500 (w), 1072 (w).

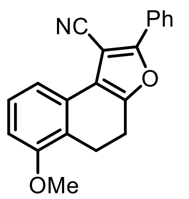

10p

6-Methoxy-2-phenyl-4,5-dihydronaphtho[2,1-*b*]furan-1-carbonitrile. Light yellow solid (35.4 mg, 78%); mp 166-168 °C. **^{1}H NMR (300 MHz, $CDCl_3$) δ ppm** 8.05-8.00 (m, 2H), 7.56 (d, *J* = 7.5 Hz, 1H), 7.51-7.38 (m, 3H), 7.26 (t, *J* = 8.1 Hz, 1H), 6.82 (dd, *J* = 8.4, 1.2 Hz, 1H), 3.86 (s, 3H), 3.15 (t, *J* = 8.4 Hz, 2H), 2.98-2.92 (m, 2H); **^{13}C NMR (75 MHz, $CDCl_3$) δ ppm** 159.43, 156.82, 153.21, 129.88, 129.13, 128.48, 127.98, 125.29, 121.05, 119.33, 115.93, 115.12, 110.09, 89.14, 55.63, 21.28, 21.12. **HRMS (ESI+):** Calculated for $C_{20}H_{16}NO_2^+$: 302.11810; Found: 302.11730. **IR (ATR)** (υ_{max}, cm^{-1}) 3054 (w), 2926 (w), 2214 (m), 1569 (w), 1240 (m).

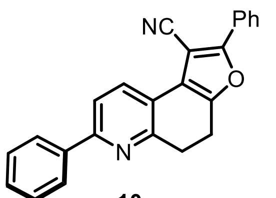

10q

2,7-Diphenyl-4,5-dihydrofuro[3,2-*f*]quinoline-1-carbonitrile. White solid (29.0 mg, 55%); mp 209-211 °C. **^{1}H NMR (300 MHz, $CDCl_3$) δ ppm** 8.20 (d, *J* = 8.1 Hz, 1H), 8.05-8.00 (m, 4H), 7.65 (d, *J* = 8.1 Hz, 1H), 7.54-7.38 (m, 6H), 3.44 (t, *J* = 8.4 Hz, 2H), 3.21-3.15 (m, 2H); **^{13}C NMR (75 MHz, $CDCl_3$) δ ppm** 160.34, 155.27, 154.45, 152.80, 139.22, 130.32, 129.62, 129.29, 129.06, 128.95, 128.21, 126.86, 125.42, 122.90, 118.92, 118.56, 115.54, 88.90, 32.00, 21.74. **HRMS (ESI+):** Calculated for

$C_{24}H_{17}N_2O^+$: 349.13409; Found: 349.13369. **IR (ATR)** (υ_{max}, cm^{-1}) 3060 (w), 2896 (w), 2220 (w), 1552 (w), 1029 (w).

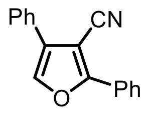

10r

2,4-Diphenylfuran-3-carbonitrile. White solid (17.6 mg, 48%); mp 85-87 °C. **^{1}H NMR (300 MHz, $CDCl_3$) δ ppm** 8.08-8.04 (m, 2H), 7.67-7.64 (m, 3H), 7.55-7.38 (m, 6H); **^{13}C NMR (75 MHz, $CDCl_3$) δ ppm** 161.28, 138.54, 130.45, 129.37, 129.25, 129.24, 128.98, 128.73, 128.23, 127.16, 125.71, 115.22, 91.78. **HRMS (ESI+):** Calculated for $C_{17}H_{12}NO^+$: 246.09189; Found: 246.09149. **IR (ATR)** (υ_{max}, cm^{-1}) 3144 (w), 2920 (w), 2230 (w), 1545 (w), 1126 (w).

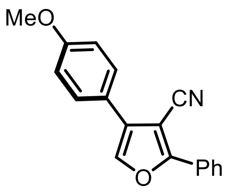

10s

4-(4-Methoxyphenyl)-2-phenylfuran-3-carbonitrile. White solid (19.7 mg, 48%); mp 147-149 °C. **^{1}H NMR (300 MHz, $CDCl_3$) δ ppm** 8.06-8.02 (m, 2H), 7.59 (s, 1H), 7.58 (d, *J* = 9.0 Hz, 2H), 7.54-7.42 (m, 3H), 7.00 (d, *J* = 8.7 Hz, 2H), 3.86 (s, 3H); **^{13}C NMR (75 MHz, $CDCl_3$) δ ppm** 161.06, 160.04, 137.90, 130.36, 129.22, 128.70, 128.43, 128.32, 125.66, 121.78, 115.41, 114.70, 91.83, 55.51. **HRMS (ESI+):** Calculated for $C_{18}H_{14}NO_2^+$: 276.10245; Found: 276.10172. **IR (ATR)** (υ_{max}, cm^{-1}) 3121 (w), 2921 (w), 2222 (w), 1548 (m), 1113 (w).

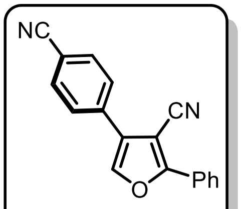

10t

4-(4-Cyanophenyl)-2-phenylfuran-3-carbonitrile. White solid (15.3 mg, 38%); mp 175-177 °C. **^{1}H NMR (300 MHz, $CDCl_3$) δ ppm** 8.07-8.03 (m, 2H), 7.80-7.73 (m, 5H), 7.57-7.47 (m, 3H); **^{13}C NMR (75 MHz, $CDCl_3$) δ ppm** 162.08, 139.55, 134.02, 133.09, 130.96, 129.38, 127.71, 127.61, 127.32, 125.83, 118.57, 114.72, 112.41, 91.19. **HRMS (ESI+):** Calculated for $C_{18}H_{11}N_2O^+$: 271.08714; Found: 271.08763. **IR (ATR)** (υ_{max}, cm^{-1}) 3128 (w), 2924 (w), 2226 (w), 1550 (w), 1198 (w).

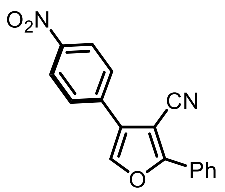

10u

4-(4-Nitrophenyl)-2-phenylfuran-3-carbonitrile. White solid (11.0 mg, 25%); mp 185-187 °C. **^{1}H NMR (300 MHz, $CDCl_3$) δ ppm** 8.34 (d, *J* = 9.0 Hz, 2H), 8.08-8.03 (m, 2H), 7.84 (d, *J* = 9.0 Hz, 2H), 7.80 (s, 1H), 7.57-7.47 (m, 3H); **^{13}C NMR (75 MHz, $CDCl_3$) δ ppm** 162.19, 147.82, 139.80, 135.90, 131.02, 129.39, 127.75, 127.64, 126.97, 125.85, 124.62, 114.67, 91.21. **HRMS (ESI+):** Calculated for $C_{17}H_{11}N_2O_3^+$: 291.07697; Found: 291.07655. **IR (ATR)** (υ_{max}, cm^{-1}) 2923 (w), 2229 (w), 1600 (s), 1511 (s), 1335 (s).

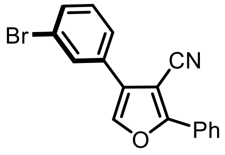

10v

4-(3-Bromophenyl)-2-phenylfuran-3-carbonitrile. White solid (17.2 mg, 35%); mp 102-104 °C. **^{1}H NMR (300 MHz, $CDCl_3$) δ ppm** 8.06-8.02 (m, 2H), 7.75 (t, *J* = 1.8 Hz, 1H), 7.66 (s, 1H), 7.62 (ddd, *J* = 7.8, 1.8, 1.2 Hz, 1H), 7.55-7.44 (m, 4H), 7.35 (t, *J* = 7.8 Hz, 1H); **^{13}C NMR (75 MHz, $CDCl_3$) δ ppm** 161.55, 138.92, 131.77, 131.41, 130.81, 130.67, 130.10, 129.30, 127.99, 127.62, 125.76, 123.24, 114.83, 91.54. **HRMS (ESI+):** Calculated for $C_{17}H_{11}BrNO^+$: 324.00240; Found:

324.00377. **IR (ATR)** (υ_{max}, cm^{-1}) 3075 (w), 2921 (w), 2223 (m), 1555 (m), 1203 (m), 1065 (m).

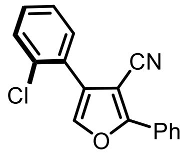

10w

4-(2-Chlorophenyl)-2-phenylfuran-3-carbonitrile. White solid (11.0 mg, 26%); mp 87-89 °C. **^{1}H NMR (300 MHz, $CDCl_3$) δ ppm** 8.08-8.05 (m, 2H), 7.70 (s, 1H), 7.55-7.44 (m, 5H), 7.41-7.32 (m, 2H); **^{13}C NMR (75 MHz, $CDCl_3$) δ ppm** 160.38, 140.89, 133.48, 131.31, 130.51, 130.46, 130.12, 129.26, 128.15, 128.10, 127.30, 125.82, 125.70, 114.68, 93.57. **HRMS (ESI+):** Calculated for $C_{17}H_{11}ClNO^+$: 280.05292; Found: 280.05212. **IR (ATR)** (υ_{max}, cm^{-1}) 3162 (w), 2921 (w), 2225 (w), 1548 (w), 1199 (w).

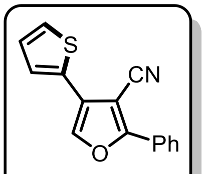

10x

2-Phenyl-4-(thiophen-2-yl)furan-3-carbonitrile. White solid (16.3 mg, 43%); mp 75-77 °C. **^{1}H NMR (300 MHz, $CDCl_3$) δ ppm** 8.05-8.01 (m, 2H), 7.67 (s, 1H), 7.54-7.43 (m, 4H), 7.33 (dd, *J* = 5.1, 1.2 Hz, 1H), 7.12 (dd, *J* = 5.1, 3.6 Hz, 1H); **^{13}C NMR (75 MHz, $CDCl_3$) δ ppm** 161.20, 138.10, 130.63, 130.33, 129.27, 128.21, 127.95, 125.88, 125.72, 125.51, 122.99, 114.94, 91.55. **HRMS (ESI+):** Calculated for $C_{15}H_{10}NOS^+$: 252.04831; Found: 252.04803. **IR (ATR)** (υ_{max}, cm^{-1}) 3070 (w), 2920 (w), 2219 (w), 1501 (s), 1226 (w).

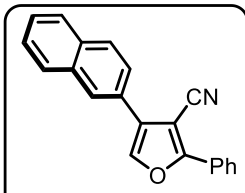

10y

4-(Naphthalen-2-yl)-2-phenylfuran-3-carbonitrile. Light yellow solid (23.4 mg, 53%); mp 109-111 °C. **^{1}H NMR (300 MHz, $CDCl_3$) δ ppm** 8.18 (d, *J* = 1.5 Hz, 1H), 8.09-8.05 (m, 2H), 7.93-7.84 (m, 3H), 7.75 (s, 1H), 7.69 (dd, *J* = 8.4, 1.8 Hz, 1H), 7.55-7.44 (m, 5H); **^{13}C NMR (75 MHz, $CDCl_3$) δ ppm** 161.44, 138.88, 133.59, 133.22, 130.50, 129.26, 129.04, 128.88, 128.42, 128.21, 127.88, 126.83, 126.73, 126.07, 125.75, 124.83, 115.36, 91.77. **HRMS (ESI+):** Calculated for $C_{21}H_{14}NO^+$: 296.10754; Found: 296.10706. **IR (ATR)** (υ_{max}, cm^{-1}) 3045 (w), 2921 (w), 2228 (w), 1547 (w), 1184 (w).

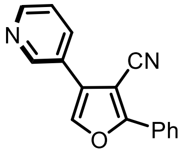

10z

2-Phenyl-4-(pyridin-3-yl)furan-3-carbonitrile. White solid (12.0 mg, 32%); mp 138-140 °C.**^{1}H NMR (300 MHz, $CDCl_3$) δ ppm** 8.85 (d, *J* = 1.8 Hz, 1H), 8.65 (dd, *J* = 4.8, 1.5 Hz, 1H), 8.07-8.01 (m, 3H), 7.73 (s, 1H), 7.56-7.46 (m, 3H), 7.42 (ddd, *J* = 7.8, 4.8, 0.6 Hz, 1H); **^{13}C NMR (75 MHz, $CDCl_3$) δ ppm** 161.76, 149.81, 148.08, 138.80, 134.39, 130.80, 129.33, 127.85, 125.77, 125.73, 125.70, 124.01, 114.68, 91.58. **HRMS (ESI+):** Calculated for $C_{16}H_{11}N_2O^+$: 247.08714; Found: 247.08657. **IR (ATR)** (υ_{max}, cm^{-1}) 3090 (w), 2225 (w), 1544 (m), 1126 (w).

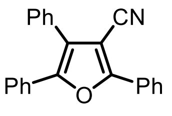

10aa

2,4,5-Triphenylfuran-3-carbonitrile. White solid (34.7 mg, 72%); mp 178-180 °C. **^{1}H NMR (300 MHz, $CDCl_3$) δ ppm** 8.14-8.11 (m, 2H), 7.56-7.42 (m, 10H), 7.36-7.31 (m, 3H); **^{13}C NMR (75 MHz, $CDCl_3$) δ ppm** 158.52, 148.74, 130.35, 129.47, 129.27, 128.86, 128.80, 128.18, 126.40, 125.64, 124.26, 114.85, 95.80. **HRMS (ESI+):** Calculated for $C_{23}H_{16}NO^+$: 322.12319; Found: 322.12192. **IR (ATR)** (υ_{max}, cm^{-1}) 3051 (w), 2952 (w), 2228 (w), 1070 (w).

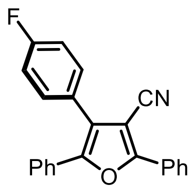

10ab

4-(4-Fluorophenyl)-2,5-diphenylfuran-3-carbonitrile. White solid (36.0 mg, 71%); mp 129-130 °C. **^{1}H NMR (300 MHz, CDCl$_3$) δ ppm** 8.13-8.09 (m, 2H), 7.56-7.44 (m, 7H), 7.37-7.31 (m, 3H), 7.20-7.13 (m, 2H); **^{13}C NMR (75 MHz, CDCl$_3$) δ ppm** 163.09 (d, J = 248.7 Hz), 158.57, 148.85, 131.34 (d, J = 8.3 Hz), 130.45, 129.30, 129.07, 129.00, 128.88, 128.06, 126.37, 125.63, 123.19, 116.45 (d, J = 21.8 Hz), 114.71, 95.69. **HRMS (ESI+):** Calculated for $C_{23}H_{15}FNO^+$: 340.11377; Found: 340.11256. **IR (ATR)** (υ_{max}, cm^{-1}) 3069 (w), 2224 (w), 1512 (w), 1224 (w).

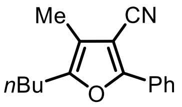

10ac

5-Butyl-4-methyl-2-phenylfuran-3-carbonitrile. Light yellow oil (20.5 mg, 57%). **^{1}H NMR (300 MHz, CDCl$_3$) δ ppm** 7.95-7.92 (m, 2H), 7.48-7.34 (m, 3H), 2.63 (t, J = 7.4 Hz, 2H), 2.09 (s, 3H), 1.65 (quint, J = 7.4 Hz, 2H), 1.37 (sext, J = 7.3 Hz, 2H), 0.95 (t, J = 7.3 Hz, 3H); **^{13}C NMR (75 MHz, CDCl$_3$) δ ppm** 157.24, 152.21, 129.54, 129.08, 128.75, 125.06, 117.20, 115.39, 94.55, 30.42, 25.75, 22.30, 13.89, 8.71. **HRMS (ESI+):** Calculated for $C_{16}H_{18}NO^+$: 240.13884; Found: 240.13855. **IR (ATR)** (υ_{max}, cm^{-1}) 2924 (w), 2222 (w), 1558 (w), 1152 (w).

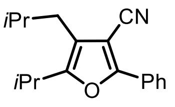

10ad

4-Isobutyl-5-isopropyl-2-phenylfuran-3-carbonitrile. White solid (18.2 mg, 45%); mp 64 °C. **^{1}H NMR (300 MHz, CDCl$_3$) δ ppm** 7.97-7.93 (m, 2H), 7.48-7.42 (m, 2H), 7.40-7.35 (m, 1H), 3.02 (sept, J = 6.9 Hz, 1H), 2.35 (d, J = 7.3 Hz, 2H), 1.95 (sept, 6.7 Hz, 1H), 1.29 (d, J = 6.9 Hz, 6H), 0.96 (d, J = 6.7 Hz, 6H); **^{13}C NMR (75 MHz, CDCl$_3$) δ ppm** 157.31, 156.77, 129.55, 129.07, 128.85, 125.09, 119.48, 115.67, 93.97, 32.93, 29.13, 26.31, 22.48, 21.58. **HRMS (ESI+):** Calculated for $C_{18}H_{22}NO^+$: 268.17014; Found: 268.16894. **IR (ATR)** (υ_{max}, cm^{-1}) 2967 (w), 2220 (m), 1557 (w), 1117 (w).

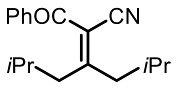

12

2-Benzoyl-3-isopropyl-4-methylpent-2-enenitrile. Light yellow oil (4.8 mg, 12%). **^{1}H NMR (300 MHz, CDCl$_3$) δ ppm** 7.94-7.91 (m, 2H) 7.65-7.62 (m, 1H), 7.53-7.48 (m, 2H), 2.52 (d, J = 7.4 Hz, 2H), 2.27 (d, J = 7.5 Hz, 2H), 2.03 (sept, J = 6.8 Hz, 1H), 1.88 (sept, J = 6.9 Hz, 1H), 1.06 (d, J = 6.6 Hz, 6H), 0.85 (d, J = 6.6 Hz, 6H); **^{13}C NMR (75 MHz, CDCl$_3$) δ ppm** 189.41, 172.55, 136.01, 134.30, 129.65, 129.02, 116.74, 113.07, 45.18, 41.48, 28.21, 28.15, 22.71, 22.68. **HRMS (ESI+):** Calculated for $C_{18}H_{24}NO^+$: 270.18579 Found: 20.18677. **IR (ATR)** (υ_{max}, cm^{-1}) 2958 (w), 2207 (w), 1673 (m), 1597 (w).

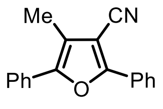

10ae

4-Methyl-2,5-diphenylfuran-3-carbonitrile. White solid (26.8 mg, 69%); mp 88-90 °C. **^{1}H NMR (300 MHz, CDCl$_3$) δ ppm** 8.06-8.02 (m, 2H), 7.71-7.67 (m, 2H), 7.53-7.34 (m, 6H), 2.42 (s, 3H); **^{13}C NMR (75 MHz, CDCl$_3$) δ ppm** 157.53, 148.96, 130.08, 129.96, 129.21, 128.99, 128.34, 125.90, 125.39, 118.93, 114.90, 96.71, 10.52. **HRMS (ESI+):** Calculated for $C_{18}H_{14}NO^+$: 260.10754; Found: 260.10723. **IR (ATR)** (υ_{max}, cm^{-1}) 3053 (w), 2921 (w), 2226 (w), 1593 (w), 1069 (w).

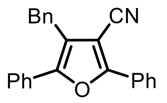

10af

4-Benzyl-2,5-diphenylfuran-3-carbonitrile. White solid (38.9 mg, 77%); mp 164-166 °C. **^{1}H NMR (300 MHz, $CDCl_3$) δ ppm** 8.09-8.06 (m, 2H), 7.68-7.65 (m, 2H), 7.53-7.24 (m, 11H), 4.18 (s, 2H); **^{13}C NMR (75 MHz, $CDCl_3$) δ ppm** 158.27, 150.05, 138.04, 130.24, 129.48, 129.23, 129.08, 128.95, 128.82, 128.31, 128.24, 126.91, 126.25, 125.50, 121.64, 114.64, 96.24, 30.49. **HRMS (ESI+):** Calculated for $C_{24}H_{18}NO^+$: 336.13884; Found: 336.13807. **IR (ATR)** (υ_{max}, cm^{-1}) 3063 (w), 2918 (w), 2222 (w), 1595 (w), 1157 (w).

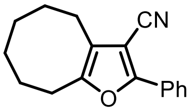

10ag

2-Phenyl-4,5,6,7,8,9-hexahydrocycloocta[*b*]furan-3-carbonitrile. Light yellow oil (25.0 mg, 66%). **^{1}H NMR (300 MHz, $CDCl_3$) δ ppm** 7.96-7.92 (m, 2H), 7.47-7.33 (m, 3H), 2.86-2.81 (m, 2H), 2.70-2.66 (m, 2H). 1.84-1.73 (m, 4H), 1.59-1.49 (m, 4H); **^{13}C NMR (75 MHz, $CDCl_3$) δ ppm** 156.83, 152.46, 129.43, 129.06, 128.76, 124.97, 121.48, 115.25, 94.16, 27.95, 26.91, 26.01, 25.99, 25.27, 22.31. **HRMS (ESI+):** Calculated for $C_{17}H_{18}NO^+$: 252.13884; Found: 252.13971. **IR (ATR)** (υ_{max}, cm^{-1}) 2927 (w), 2221 (w), 1555 (w), 1073 (w).

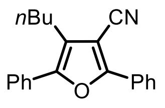

10ah

4-Butyl-2,5-diphenylfuran-3-carbonitrile. White solid (31.0 mg, 69%); mp 70-72 °C. **^{1}H NMR (300 MHz, $CDCl_3$) δ ppm** 8.07-8.04 (m, 2H), 7.69-7.66 (m, 2H), 7.53-7.35 (m, 6H), 2.82-2.77 (m, 2H), 1.74 (quint, *J* = 7.6 Hz, 2H), 1.48 (sext, *J* = 7.3 Hz, 2H), 0.98 (t, *J* = 7.3 Hz, 3H); **^{13}C NMR (75 MHz, $CDCl_3$) δ ppm** 157.83, 148.79, 130.06, 129.96, 129.19, 129.02, 128.45, 128.38, 126.08, 125.43, 124.23, 115.03, 95.90, 31.91, 24.62, 22.75, 13.96. **HRMS (ESI+):** Calculated for $C_{21}H_{20}NO^+$: 302.15449; Found: 302.15484. **IR (ATR)** (υ_{max}, cm^{-1}) 3057 (w), 2931 (w), 2220 (w), 1593 (w), 1065 (w).

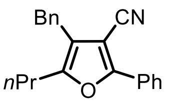

10ai

4-Benzyl-2-phenyl-5-propylfuran-3-carbonitrile. Light yellow oil (3.0 mg, 7%). **^{1}H NMR (300 MHz, $CDCl_3$) δ ppm** 7.95-7.91 (m, 2H), 7.47-7.19 (m, 8H), 3.84 (s, 2H), 2.63 (t, *J* = 7.4 Hz, 2H), 1.68 (quint, *J* = 7.4 Hz, 2H), 0.95 (t, *J* = 7.4 Hz, 3H); **^{13}C NMR (75 MHz, $CDCl_3$) δ ppm** 157.85, 152.81, 138.91, 129.72, 129.10, 128.81, 128.60, 128.56, 126.75, 125.14, 120.96, 115.23, 93.95, 29.97, 28.17, 21.76, 13.89. **HRMS (ESI+):** Calculated for $C_{21}H_{20}NO^+$: 302.15449; Found: 302.15469. **IR (ATR)** (υ_{max}, cm^{-1}) 329 (w), 2926 (w), 2222 (w), 1491 (w).

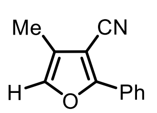

10aj

4-Methyl-2-phenylfuran-3-carbonitrile. White solid (17.6 mg, 64%); mp 48-50 °C. **^{1}H NMR (300 MHz, $CDCl_3$) δ ppm** 7.98-7.95 (m, 2H), 7.50-7.39 (m, 3H), 7.26 (q, *J* = 1.5 Hz, 1H), 2.18 (d, *J* = 1.3 Hz, 3H); **^{13}C NMR (75 MHz, $CDCl_3$) δ ppm** 159.75, 138.97, 130.07, 129.14, 128.44, 125.35, 123.54, 114.79, 94.37, 8.65. **HRMS (ESI+):** Calculated for $C_{12}H_{10}NO^+$: 184.07624; Found: 184.07604. **IR (ATR)** (υ_{max}, cm^{-1}) 3126 (w), 2923 (w), 2222 (w), 1548 (w), 1066 (w).

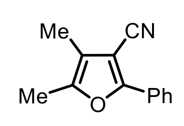

10ak

4,5-Dimethyl-2-phenylfuran-3-carbonitrile. White solid (15.7 mg, 53%); mp 90-92 °C. **^{1}H NMR (300 MHz, $CDCl_3$) δ ppm** 7.94-7.90 (m, 2H), 7.47-7.34 (m, 3H), 2.29 (d, *J* = 0.9 Hz, 3H), 2.08 (d, *J* = 0.9 Hz, 3H); **^{13}C NMR (75 MHz, $CDCl_3$) δ ppm** 157.28, 148.09, 129.56, 129.08, 128.65, 125.02, 117.41, 115.33, 94.60, 11.58, 8.73. **HRMS (ESI+):** Calculated for $C_{13}H_{12}NO^+$: 198.09189; Found: 198.09125. **IR (ATR)** ($υ_{max}$, cm^{-1}) 3063 (w), 2918 (w), 2219 (w), 1556 (w), 1158 (w).

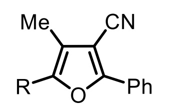

10aj/10al
R = H/SPh

4-Methyl-2-phenylfuran-3-carbonitrile (10aj) / 4-methyl-2-phenyl-5-(phenylthio)furan-3-carbonitrile (10al) *(inseparable mixture)*. Yellow solid (17.1 mg, **10aj** 20%, **10al** 27%). **^{1}H NMR (300 MHz, $CDCl_3$) δ ppm** 8.02-7.95 (m, 3.46H), 7.51-7.41 (m, 5.2H), 7.32-7.19 (m, 5.74H), 2.28 (s, 3H), 2.18 (d, *J* = 1.5 Hz, 2.2H); **^{13}C NMR (75 MHz, $CDCl_3$) δ ppm** *(compound **10aj**)* 159.78, 138.98, 130.09, 129.16, 128.46, 125.38, 123.57, 114.80, 94.40, 8.67; *(compound **10al**)* 161.65, 134.89, 131.91, 130.75, 129.53, 129.24, 129.19, 128.05, 127.91, 127.12, 125.77, 114.29, 95.55, 10.25. **HRMS (ESI+):** Calculated for $C_{18}H_{14}NOS^+$ (**10al**): 292.07961; Found: 292.07847. **IR (ATR)** ($υ_{max}$, cm^{-1}) 3068 (w), 2920 (w), 2220 (w), 1580 (w), 1440 (w), 1068 (w).

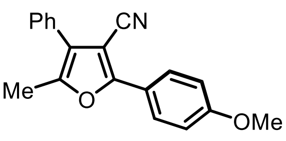

10am

2-(4-Methoxyphenyl)-5-methyl-4-phenylfuran-3-carbonitrile. White solid (17.5 mg, 40%); mp 134-136 °C. **^{1}H NMR (300 MHz, $CDCl_3$) δ ppm** 7.98-7.95 (m, 2H), 7.47-7.34 (m, 5H), 7.00-6.97 (m, 2H), 3.86 (s, 3H), 2.44 (s, 3H); **^{13}C NMR (75 MHz, $CDCl_3$) δ ppm** 160.93, 158.76, 147.80, 130.56, 128.99, 128.66, 128.03, 127.07, 123.17, 121.31, 115.78, 114.57, 91.47, 55.55, 12.56. **HRMS (ESI+):** Calculated for $C_{19}H_{16}NO_2^+$: 290.11810; Found: 290.11819. **IR (ATR)** ($υ_{max}$, cm^{-1}) 3054 (w), 2964 (w), 2220 (w), 1608 (w), 1033 (w).

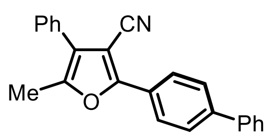

10an

2-([1,1'-Biphenyl]-4-yl)-5-methyl-4-phenylfuran-3-carbonitrile. White solid (23.3 mg, 46%); mp 168-170 °C. **^{1}H NMR (300 MHz, $CDCl_3$) δ ppm** 8.10 (d, *J* = 8.5 Hz, 2H), 7.71 (d, *J* = 8.5 Hz, 2H), 7.67-7.60 (m, 2H), 7.53-7.35 (m, 8H), 2.48 (s, 3H); **^{13}C NMR (75 MHz, $CDCl_3$) δ ppm** 158.19, 148.73, 142.57, 140.17, 130.35, 129.09, 129.05, 128.70, 128.17, 128.05, 127.75, 127.26, 127.20, 125.77, 123.64, 115.45, 93.17, 12.66. **HRMS (ESI+):** Calculated for $C_{24}H_{18}NO^+$: 336.13884; Found: 336.13818. **IR (ATR)** ($υ_{max}$, cm^{-1}) 3037 (w), 2917 (w), 2220 (w), 1570 (w), 1127 (w).

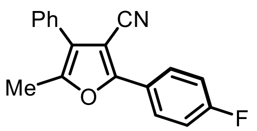

10ao

2-(4-Fluorophenyl)-5-methyl-4-phenylfuran-3-carbonitrile. White solid (24.6 mg, 59%); mp 98-99 °C. **^{1}H NMR (300 MHz, $CDCl_3$) δ ppm** 8.05-7.98 (m, 2H), 7.51-7.36 (m, 5H), 7.26-7.13 (m, 2H), 2.46 (s, 3H); **^{13}C NMR (75 MHz, $CDCl_3$) δ ppm** 163.53 (*J* = 251.42 Hz), 157.53, 148.65, 130.23, 129.06, 128.65, 128.21, 127.46 (*J* = 8.5 Hz), 124.78 (*J* = 3.2 Hz), 123.50, 116.38 (*J* = 22.2 Hz), 115.31, 92.96, 12.58. **HRMS (ESI+):** Calculated for $C_{18}H_{13}FNO^+$: 278.09812; Found: 278.09716. **IR (ATR)** ($υ_{max}$, cm^{-1}) 3070 (w), 2920 (w), 2219 (w), 1501 (s), 1226 (w).

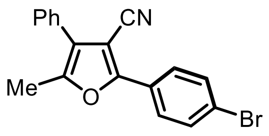

10ap

2-(4-Bromophenyl)-5-methyl-4-phenylfuran-3-carbonitrile. White solid (35.0 mg, 69%); mp 130-132 °C. **^{1}H NMR (300 MHz, $CDCl_3$) δ ppm** 7.90 (d, *J* = 8.6 Hz, 2H), 7.61 (d, *J* = 8.6 Hz, 2H), 7.51-7.36 (m, 5H), 2.46 (s, 3H); **^{13}C NMR (75 MHz, $CDCl_3$) δ ppm** 157.22, 149.06, 132.42, 130.09, 129.08, 128.67, 128.28, 127.27, 126.71, 124.18, 123.77, 115.13, 93.70, 12.65. **HRMS (ESI+):** Calculated for $C_{18}H_{13}NOBr^+$: 338.01805; Found: 338.01853. **IR (ATR)** (υ_{max}, cm^{-1}) 3052 (w), 2917 (w), 2223 (m), 1482 (m), 826 (m).

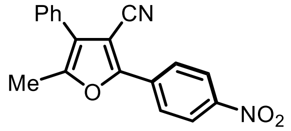

10aq

5-Methyl-2-(4-nitrophenyl)-4-phenylfuran-3-carbonitrile. White solid (35.0 mg, 77%); mp 185-187 °C. **^{1}H NMR (300 MHz, $CDCl_3$) δ ppm** 8.35-8.32 (m, 2H), 8.21-8.18 (m, 2H), 7.53-7.39 (m, 5H), 2.51 (s, 3H); **^{13}C NMR (75 MHz, $CDCl_3$) δ ppm** 155.25, 150.96, 147.90, 133.73, 129.54, 129.20, 128.67, 128.61, 125.72, 124.70, 124.61, 114.54, 96.50, 12.78. **HRMS (ESI+):** Calculated for $C_{18}H_{13}N_2O_3^+$: 305.09262; Found: 305.09176. **IR (ATR)** (υ_{max}, cm^{-1}) 3104 (w), 2924 (w), 2230 (w), 1561 (w), 1124 (m).

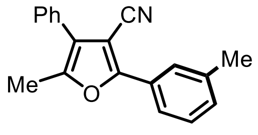

10ar

5-Methyl-4-phenyl-2-(*m*-tolyl)furan-3-carbonitrile. White solid (20.8 mg, 51%); mp 56-58 °C. **^{1}H NMR (300 MHz, $CDCl_3$) δ ppm** 7.85-7.82 (m, 2H), 7.49-7.43 (m, 4H), 7.41-7.34 (m, 2H), 7.25-7.21 (m, 1H), 2.47 (s, 3H), 2.44 (s, 3H); **^{13}C NMR (75 MHz, $CDCl_3$) δ ppm** 158.60, 148.51, 138.93, 130.80, 130.41, 129.08, 129.02, 128.70, 128.35, 128.11, 125.86, 123.49, 122.62, 115.42, 93.03, 21.65, 12.60. **HRMS (ESI+):** Calculated for $C_{19}H_{16}NO^+$: 274.12319; Found: 274.12227. **IR (ATR)** (υ_{max}, cm^{-1}) 3059 (w), 2959 (w), 2217 (w), 1553 (w), 1192 (w).

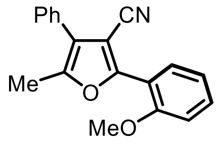

10as

2-(2-Methoxyphenyl)-5-methyl-4-phenylfuran-3-carbonitrile. White solid (22.4 mg, 52%); mp 98-100 °C. **^{1}H NMR (300 MHz, $CDCl_3$) δ ppm** 7.67 (dd, *J* = 7.7, 1.8 Hz, 1H), 7.51-7.34 (m, 6H), 7.08-7.00 (m, 2H), 3.98 (s, 3H), 2.45 (s, 3H); **^{13}C NMR (75 MHz, $CDCl_3$) δ ppm** 156.90, 155.63, 148.51, 131.48, 130.84, 129.28, 128.96, 128.90, 127.96, 123.70, 120.80, 118.03, 114.78, 111.69, 97.07, 55.10, 12.56. **HRMS (ESI+):** Calculated for $C_{19}H_{16}NO_2^+$: 290.11810; Found: 290.11681. **IR (ATR)** (υ_{max}, cm^{-1}) 3053 (w), 2921 (w), 2230 (w), 2033 (w), 1602 (w), 1248 (m), 1105 (m).

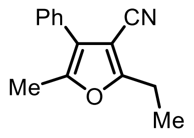

10at

2-Ethyl-5-methyl-4-phenylfuran-3-carbonitrile. White solid (21.0 mg, 66%); mp 66-68 °C. **^{1}H NMR (300 MHz, $CDCl_3$) δ ppm** 7.47-7.31 (m, 5H), 2.84 (q, *J* = 7.6 Hz, 2H), 2.37 (s, 3H), 1.34 (t, *J* = 7.6 Hz, 3H); **^{13}C NMR (75 MHz, $CDCl_3$) δ ppm** 164.99, 147.71, 130.90, 128.96, 128.49, 127.82, 121.37, 114.47, 94.46, 21.43, 12.47, 12.15. **HRMS (ESI+):** Calculated for $C_{14}H_{14}NO^+$: 212.10754; Found: 212.10808. **IR (ATR)** (υ_{max}, cm^{-1}) 3058 (w), 2978 (w), 2222 (m), 1575 (w), 1187 (w).

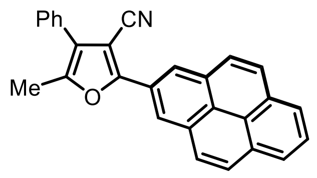

10au

5-Methyl-4-phenyl-2-(pyren-2-yl)furan-3-carbonitrile. White solid (32.3 mg, 58%); mp 220-222 °C. **^{1}H NMR (300 MHz, $CDCl_3$) δ ppm** 8.50 (d, *J* = 9.3 Hz, 1H), 8.38 (d, *J* = 8.0 Hz, 1H), 8.27-8.03 (m, 7H), 7.61-7.50 (m, 4H), 7.45-7.39 (m, 1H), 2.62 (s, 3H); **^{13}C NMR (75 MHz, $CDCl_3$) δ ppm** 160.31, 149.78, 132.87, 131.42, 130.88, 130.50, 129.13, 129.10, 128.78, 128.21, 127.42, 127.30, 126.58, 126.24, 125.99, 125.19, 125.00, 124.65, 124.62, 123.42, 122.26, 115.03, 97.42, 12.94. **HRMS (ESI+):** Calculated for $C_{28}H_{18}NO^+$: 384.13884; Found: 384.13791. **IR (ATR)** (υ_{max}, cm^{-1}) 3038 (w), 2921 (w), 2225 (w), 1564 (w), 1133 (w).

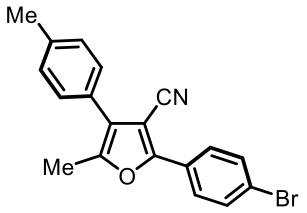

10av

2-(4-Bromophenyl)-5-methyl-4-(*p*-tolyl)furan-3-carbonitrile. White solid (29.3 mg, 58%); mp 118-120 °C. **^{1}H NMR (300 MHz, $CDCl_3$) δ ppm** 7.90-7.87 (m, 2H), 7.62-7.59 (m, 2H), 7.37-7.34 (m, 2H), 7.30-7.27 (m, 2H), 2.45 (s, 3H), 2.41 (s, 3H); **^{13}C NMR (75 MHz, $CDCl_3$) δ ppm** 157.05, 148.82, 138.17, 132.39, 129.77, 128.51, 127.33, 127.11, 126.67, 124.07, 123.73, 115.20, 93.80, 21.40, 12.62. **HRMS (ESI+):** Calculated for $C_{19}H_{15}NOBr^+$:352.03370; Found: 352.03321. **IR (ATR)** (υ_{max}, cm^{-1}) 3038 (w), 2919 (w), 2225 (m), 1074 (m).

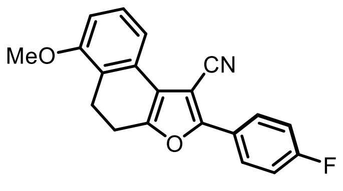

10aw

2-(4-Fluorophenyl)-6-methoxy-4,5-dihydronaphtho[2,1-*b*]furan-1-carbonitrile. White solid (32.0 mg, 67%); mp 178-180°C. **^{1}H NMR (300 MHz, $CDCl_3$) δ ppm** 8.04-7.99 (m, 2H), 7.56 (d, *J* = 7.6 Hz, 1H), 7.27 (t, *J* = 8.0 Hz, 1H), 7.18 (t, *J* = 8.7 Hz, 2H), 6.84 (d, *J* = 8.3 Hz, 1H), 3.87 (s, 3H), 3.16 (t, *J* = 8.1 Hz, 2H), 2.96 (t, *J* = 8.1 Hz, 2H); **^{13}C NMR (75 MHz, $CDCl_3$) δ ppm** 163.62 (d, *J* = 251.5 Hz), 158.71, 157.00, 153.28, 129.88, 128.10, 127.55 (d, *J* = 8.5 Hz), 125.01 (*J* = 3.4 Hz), 121.21, 119.46, 116.42 (*J* = 22.1 Hz), 115.81, 115.27, 110.38, 109.80, 55.75, 21.39, 21.21. **HRMS (ESI+):** Calculated for 320.10868; Found: 320.10870. **IR (ATR)** (υ_{max}, cm^{-1}) 3077 (w), 2933 (w), 2223 (w), 1561 (w), 1260 (m), 1236 (m), 1169 (w).

13. Crystallographic data of pyrrole 6a and furan 10a

13.1 Pyrrole 6a

The single crystal was prepared by diffusing heptanes into an ethyl acetate solution of **6a**.

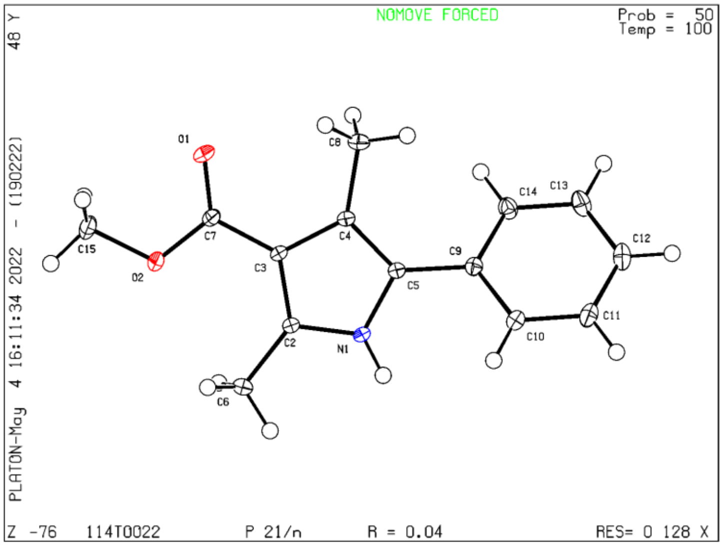

Supplementary Figure 2. ORTEP drawing of **6a** showing thermal ellipsoids at the 50% probability level. CCDC deposition number 2177946 contains the supplementary crystallographic data for this paper which can be obtained free of charge at https://www.ccdc.cam.ac.uk/structures/

```
Bond precision:   C-C = 0.0010 A          Wavelength=0.71073

Cell:             a=7.5726(1)     b=12.6658(2)       c=12.7853(2)
                  alpha=90        beta=92.774(1)     gamma=90
Temperature:      100 K

                 Calculated                  Reported
Volume           1224.84(3)                  1224.84(3)
Space group      P 21/n                      P 21/n
Hall group       -P 2yn                      -P 2yn
Moiety formula   C14 H15 N O2                C14 H15 N O2
Sum formula      C14 H15 N O2                C14 H15 N O2
Mr               229.27                      229.27
Dx,g cm-3        1.243                       1.243
Z                4                           4
Mu (mm-1)        0.083                       0.083
F000             488.0                       488.0
F000'            488.22
h,k,lmax         12,20,20                    12,20,20
Nref             5374                        5361
Tmin,Tmax        0.971,0.983                 0.710,0.747
Tmin'            0.966

Correction method= # Reported T Limits: Tmin=0.710 Tmax=0.747
AbsCorr = MULTI-SCAN

Data completeness= 0.998            Theta(max)= 34.972

                                                      wR2(reflection
R(reflections)= 0.0425( 4572)                         0.1209( 5361)

S = 1.051                   Npar= 160
```

13.2 Furan 10a

The single crystal was prepared by diffusing heptanes into an ethyl acetate solution of **10a**.

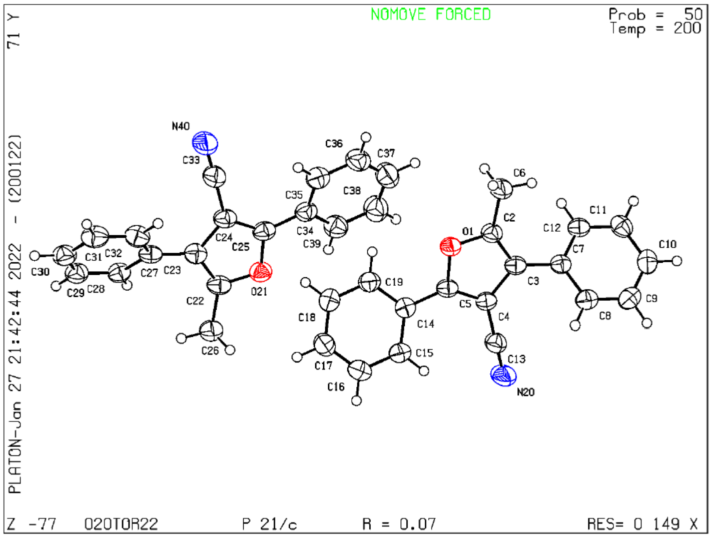

Supplementary Figure 3. ORTEP drawing of **10a** showing thermal ellipsoids at the 50% probability level. CCDC deposition number 2155771 contains the supplementary crystallographic data for this paper which can be obtained free of charge at https://www.ccdc.cam.ac.uk/structures/

Bond precision: C-C = 0.0036 A Wavelength=1.54178

Cell:	a=9.7123(2)	b=15.2241(4)	c=18.7948(5)
	alpha=90	beta=101.086(1)	gamma=90
Temperature:	200 K		

	Calculated	Reported
Volume	2727.16(12)	2727.16(12)
Space group	P 21/c	P 21/c
Hall group	-P 2ybc	-P 2ybc
Moiety formula	C18 H13 N O	C18 H13 N O
Sum formula	C18 H13 N O	C18 H13 N O
Mr	259.29	259.29
Dx,g cm-3	1.263	1.263
Z	8	8
Mu (mm-1)	0.617	0.617
F000	1088.0	1088.0
F000’	1091.08	
h,k,lmax	11,18,22	11,18,22
Nref	5014	4942
Tmin,Tmax	0.859,0.900	0.653,0.753
Tmin’	0.761	

Correction method= # Reported T Limits: Tmin=0.653 Tmax=0.753
AbsCorr = MULTI-SCAN

Data completeness= 0.986 Theta(max)= 68.328

R(reflections)= 0.0695(3828) wR2(reflections)= 0.1637(4942)

S = 1.158 Npar= 363

14. Copies of ^{1}H NMR and ^{13}C NMR spectra

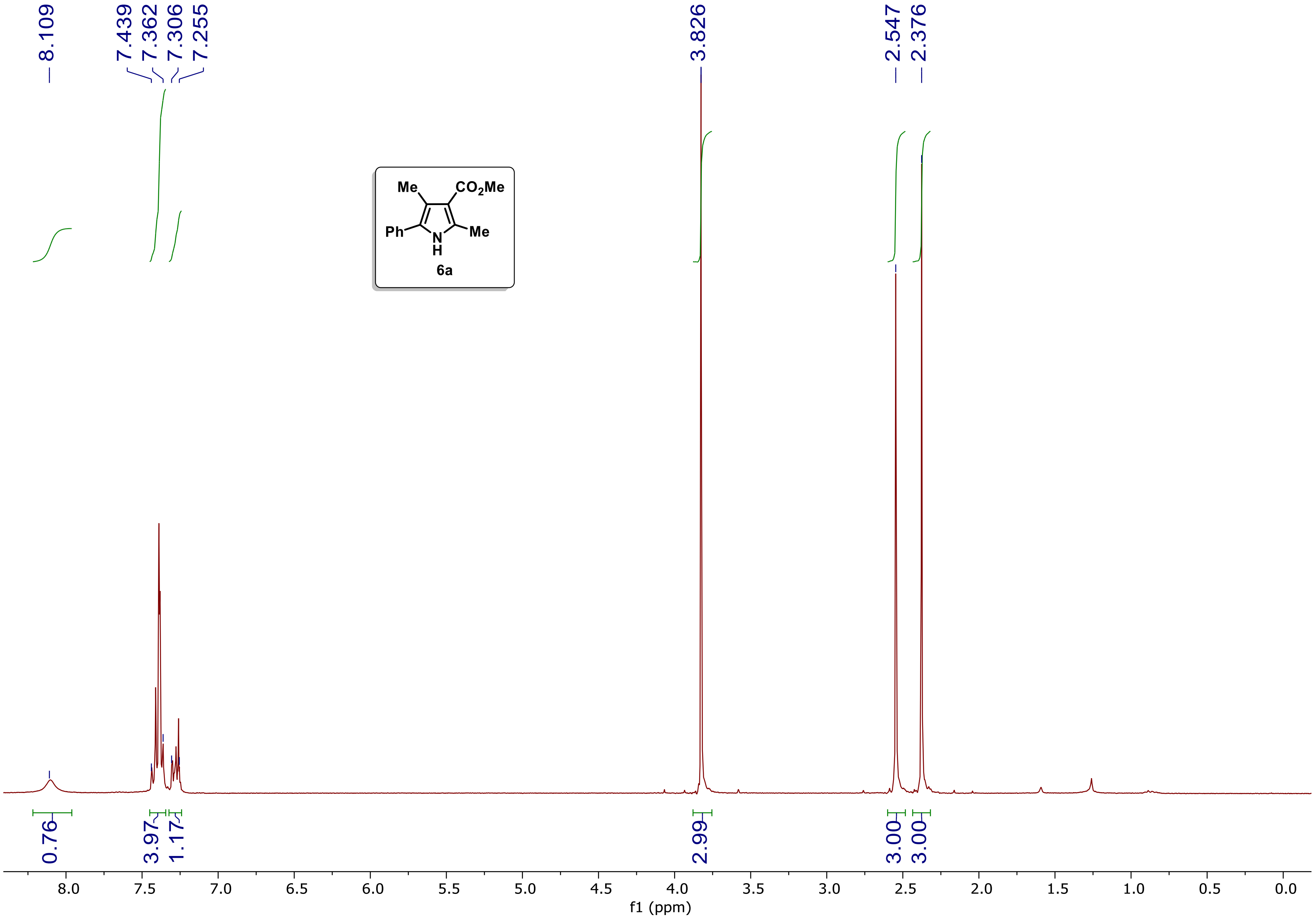

8.109
7.439
7.362
7.306
7.255
3.826
2.547
2.376
Me
CO2Me
Ph
N
H
Me
6a
0.76
3.97
1.17
2.99
3.00
3.00
8.0
7.5
7.0
6.5
6.0
5.5
5.0
4.5
4.0
3.5
3.0
2.5
2.0
1.5
1.0
0.5
0.0
f1 (ppm)

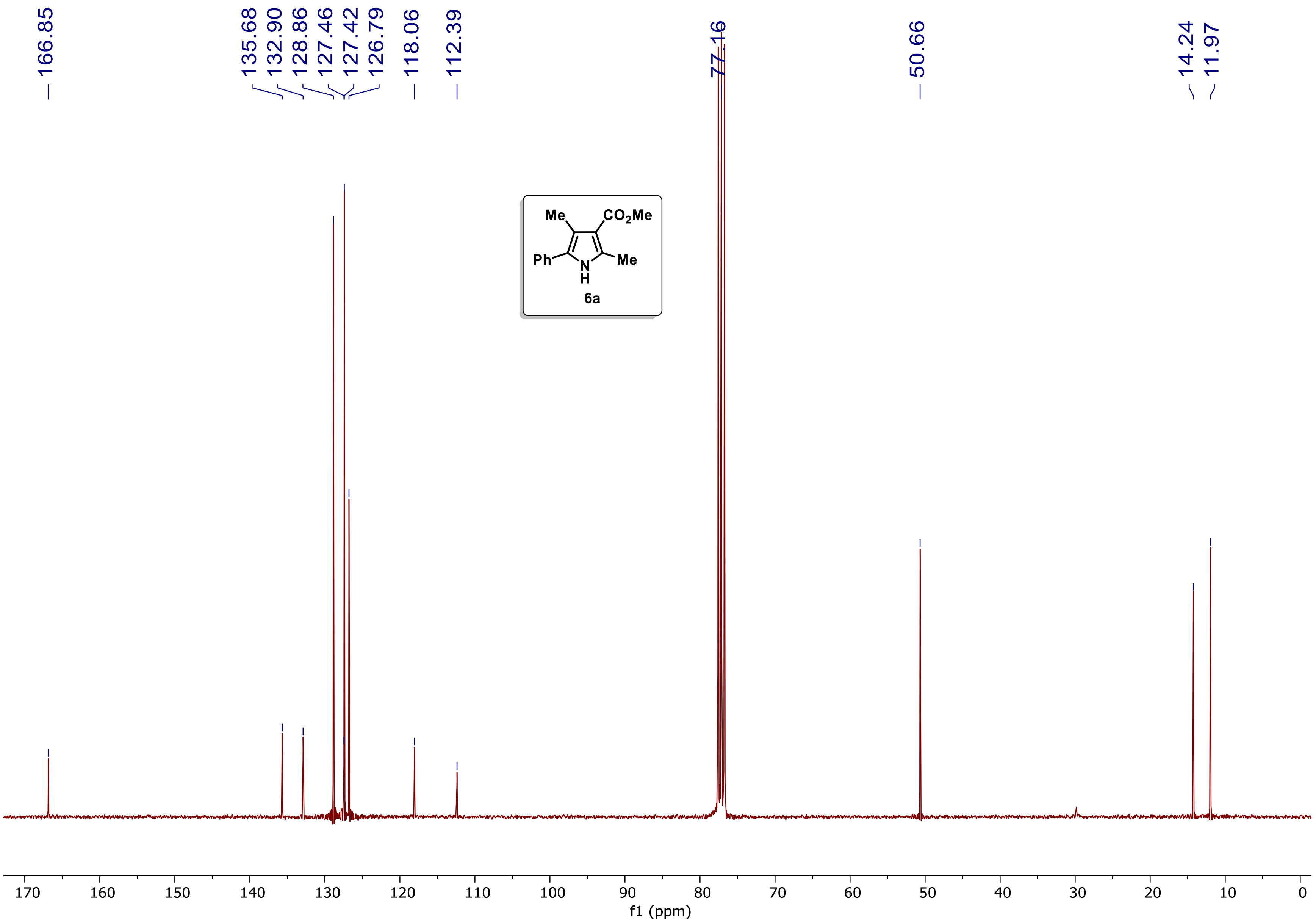

166.85
135.68
132.90
128.86
127.46
127.42
126.79
118.06
112.39
77.16
50.66
14.24
11.97
Me
CO2Me
Ph
N
H
Me
6a
170
160
150
140
130
120
110
100
90
80
70
60
50
40
30
20
10
0
f1 (ppm)

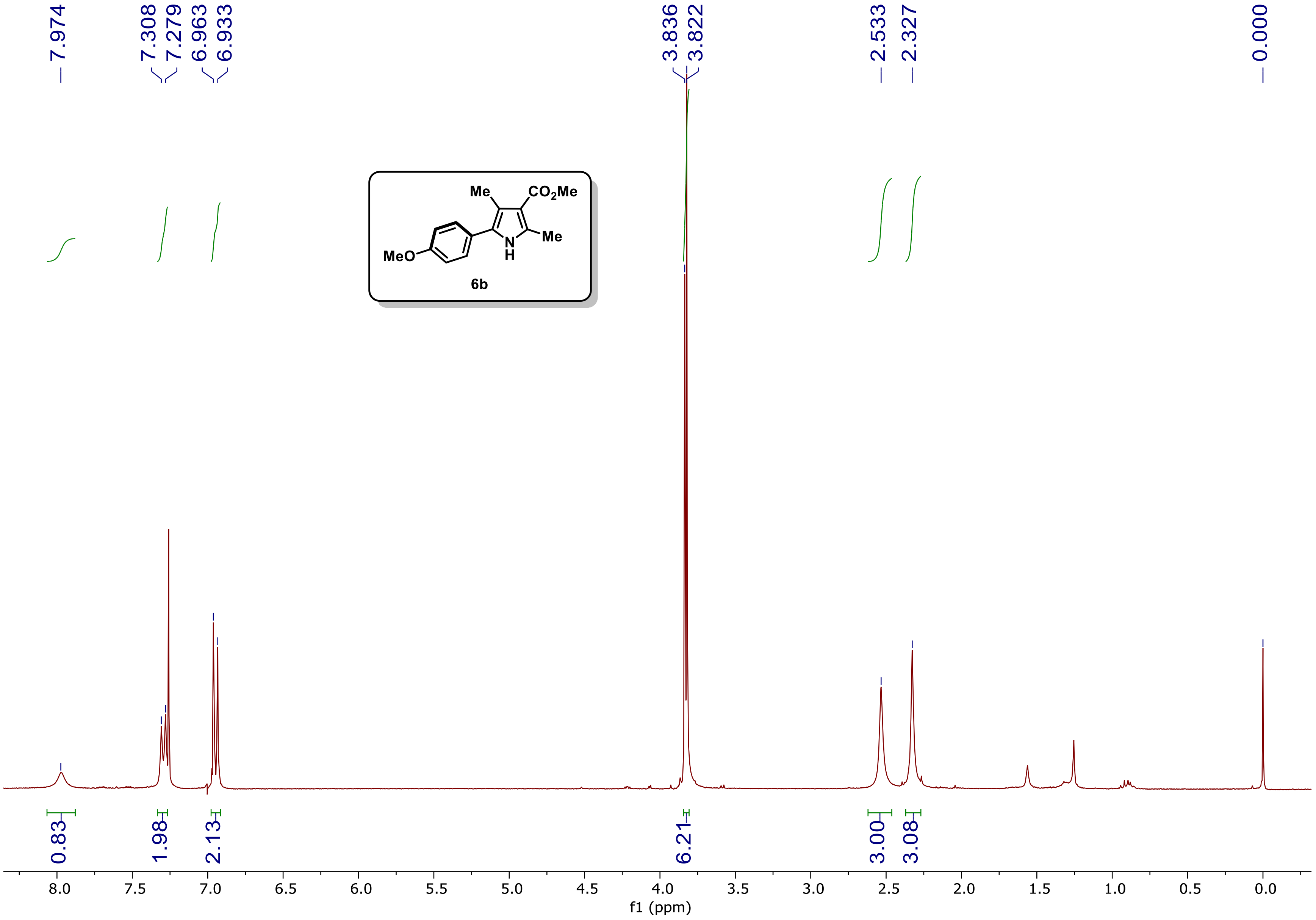
7.974
7.308
7.279
6.963
6.933
3.836
3.822
2.533
2.327
0.000
Me
CO2Me
Me
N
H
MeO
6b
0.83
1.98
2.13
6.21
3.00
3.08
8.0
7.5
7.0
6.5
6.0
5.5
5.0
4.5
4.0
3.5
3.0
2.5
2.0
1.5
1.0
0.5
0.0
f1 (ppm)

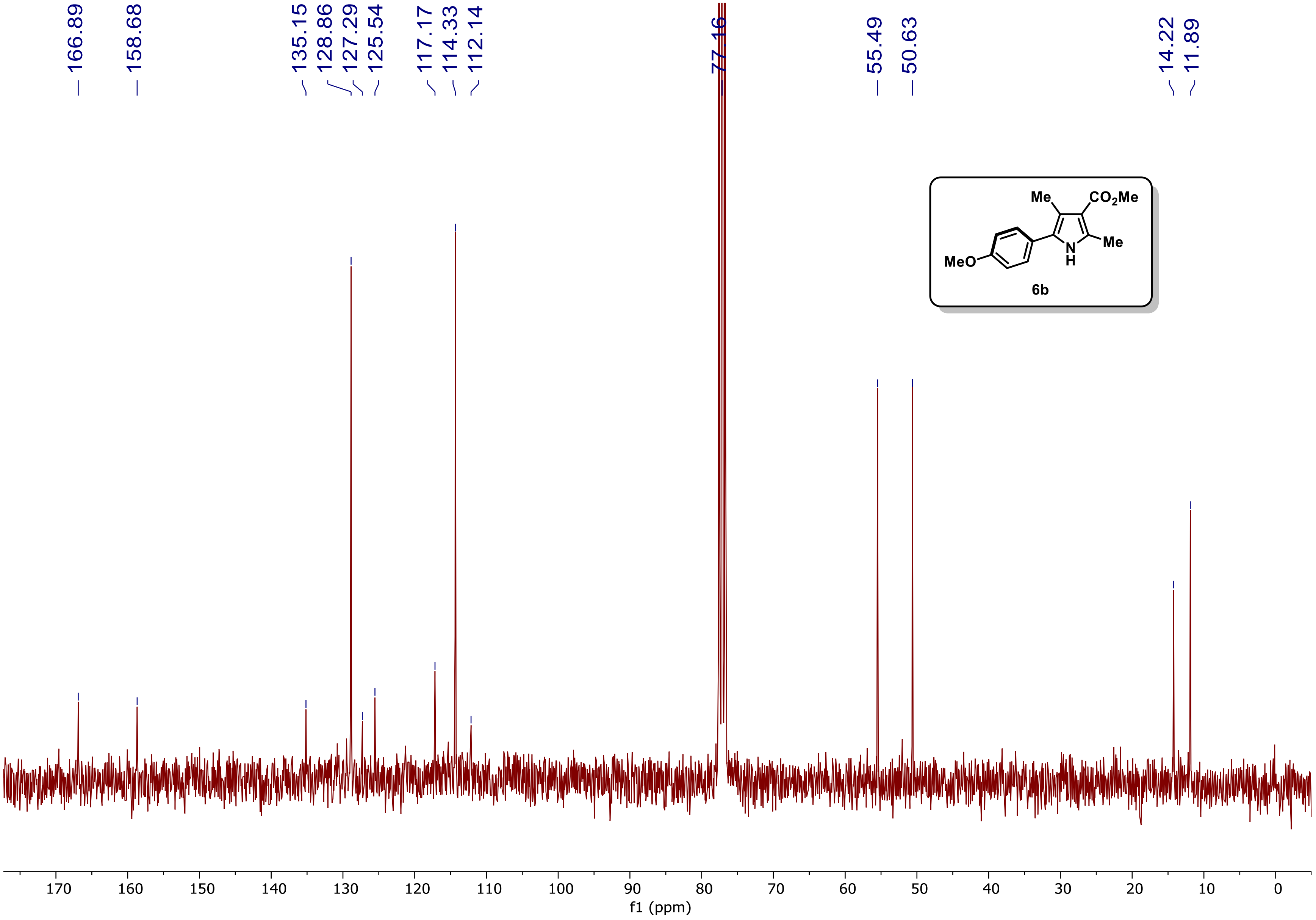
166.89
158.68
135.15
128.86
127.29
125.54
117.17
114.33
112.14
77.16
55.49
50.63
14.22
11.89
Me
CO2Me
Me
N
H
MeO
6b
170 160 150 140 130 120 110 100 90 80 70 60 50 40 30 20 10 0
f1 (ppm)

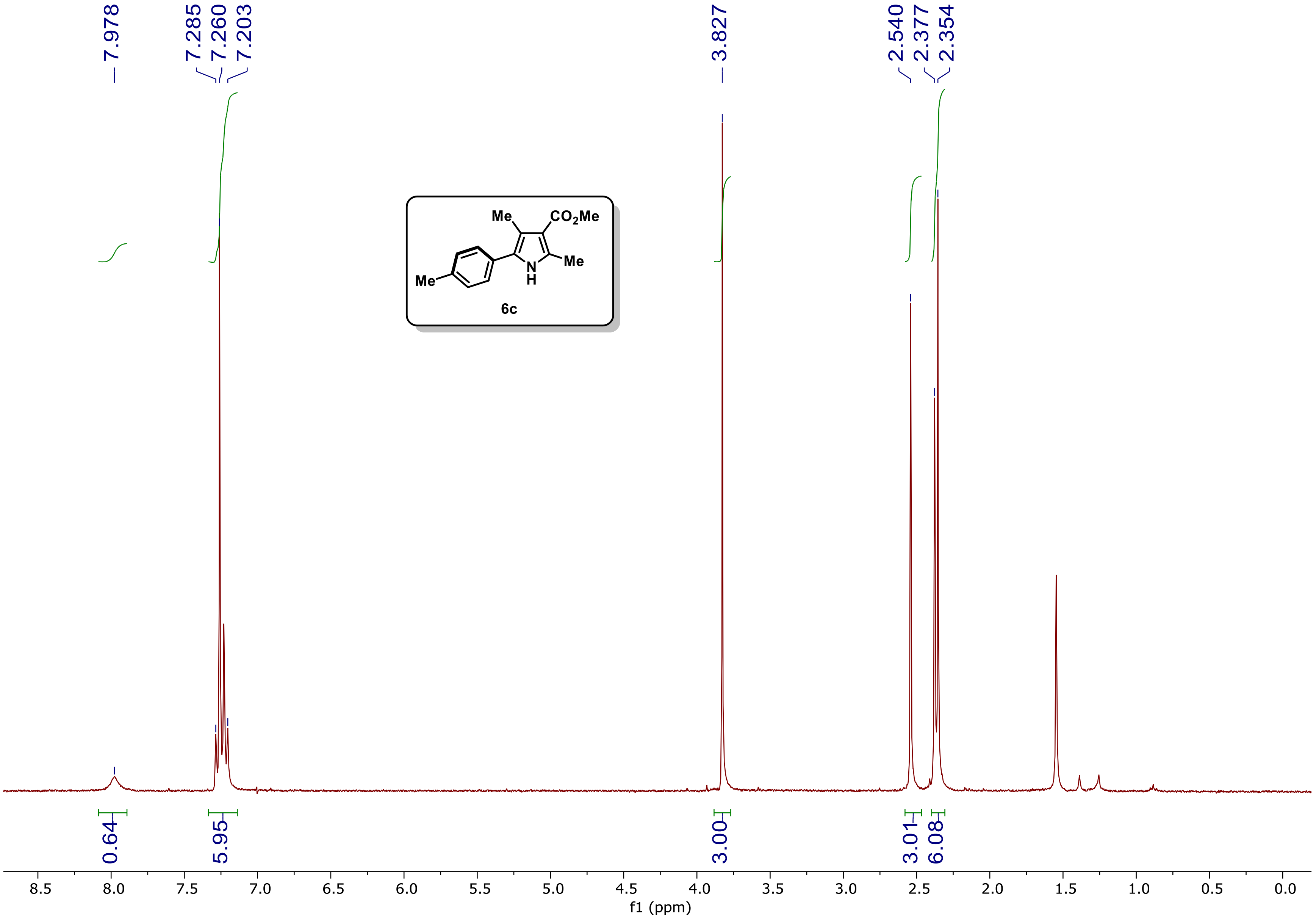

7.978
7.285
7.260
7.203
3.827
2.540
2.377
2.354
Me
CO2Me
Me
N
H
Me
6c
0.64
5.95
3.00
3.01
6.08
8.5
8.0
7.5
7.0
6.5
6.0
5.5
5.0
4.5
4.0
3.5
3.0
2.5
2.0
1.5
1.0
0.5
0.0
f1 (ppm)

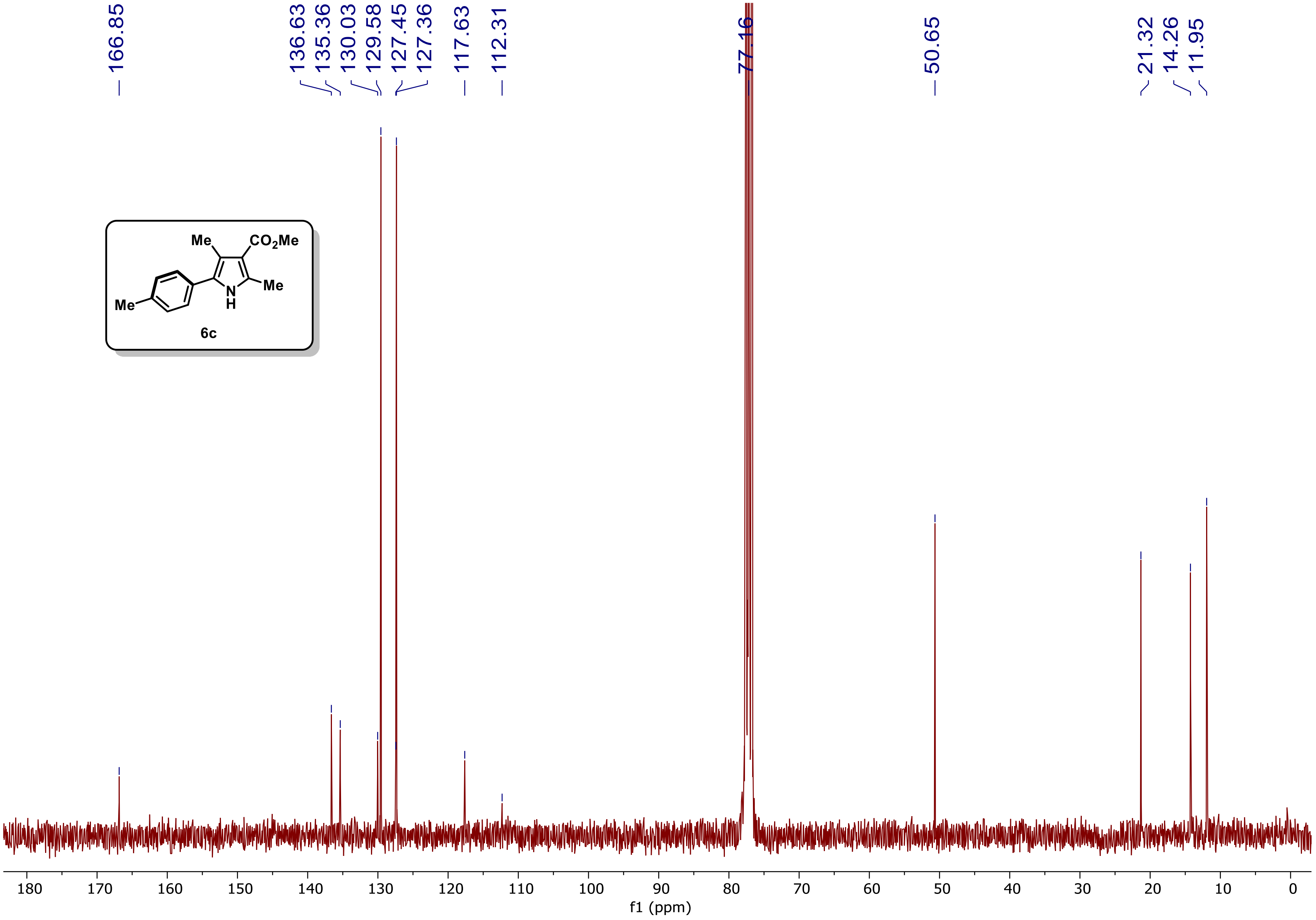

166.85
136.63
135.36
130.03
129.58
127.45
127.36
117.63
112.31
77.16
50.65
21.32
14.26
11.95
Me
CO2Me
Me
Me
N
H
6c
180 170 160 150 140 130 120 110 100 90 80 70 60 50 40 30 20 10 0
f1 (ppm)

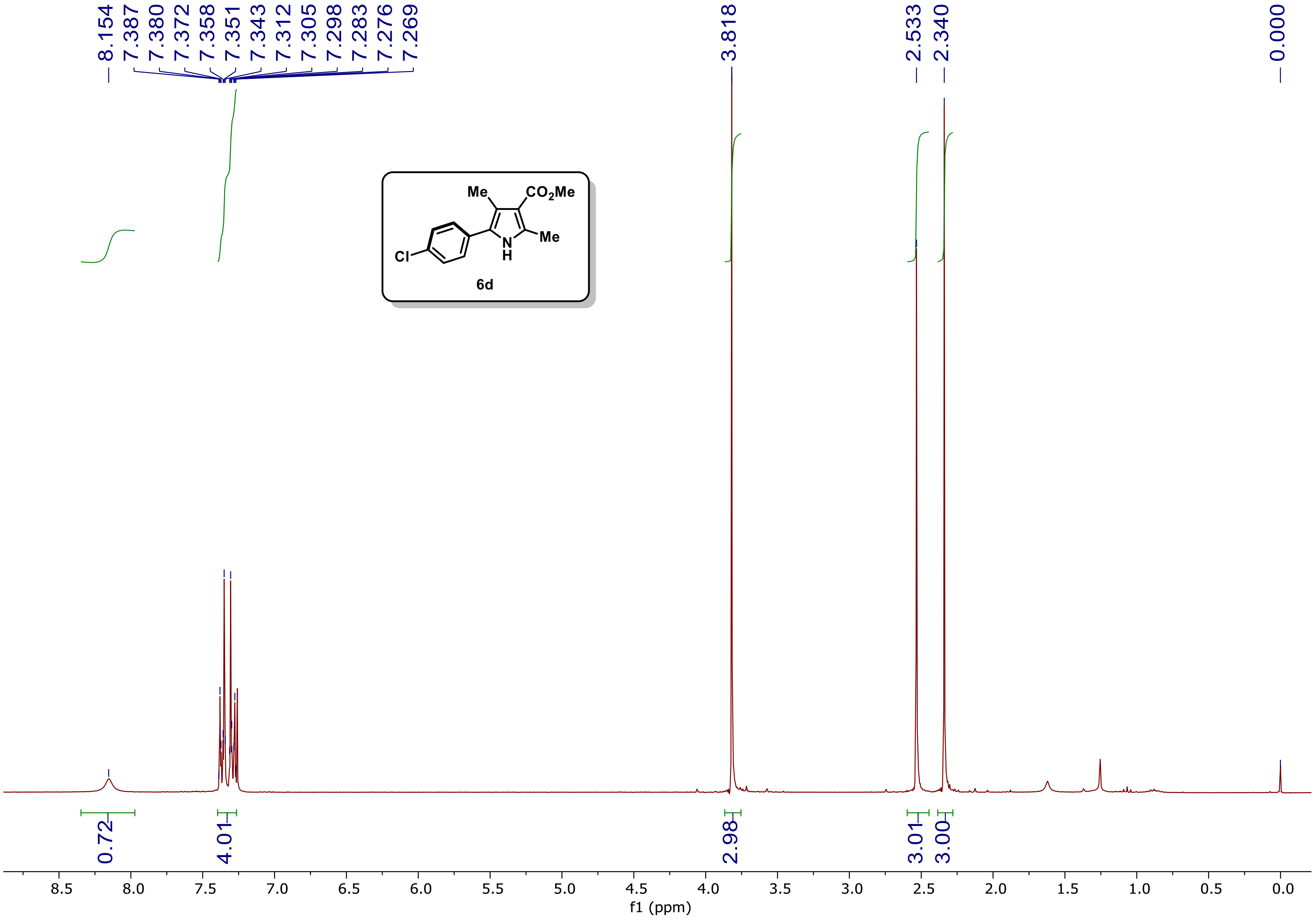
8.154
7.387
7.380
7.372
7.358
7.351
7.343
7.312
7.305
7.298
7.283
7.276
7.269
3.818
2.533
2.340
0.000
Me
CO2Me
Me
Cl
N
H
6d
0.72
4.01
2.98
3.01
3.00
8.5
8.0
7.5
7.0
6.5
6.0
5.5
5.0
4.5
4.0
3.5
3.0
2.5
2.0
1.5
1.0
0.5
0.0
f1 (ppm)

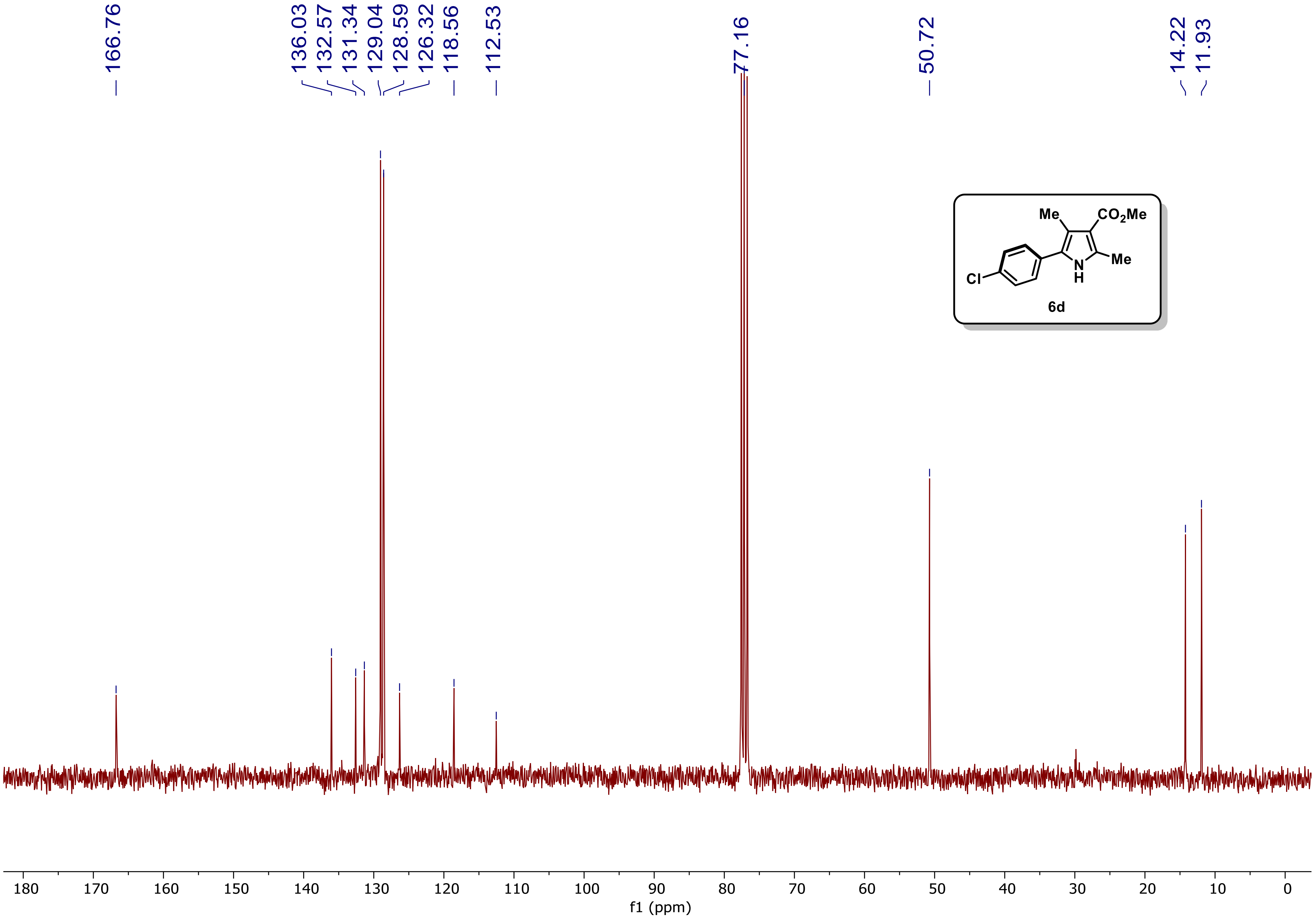
166.76
136.03
132.57
131.34
129.04
128.59
126.32
118.56
112.53
77.16
50.72
14.22
11.93
Me
CO2Me
Me
Cl
N
H
6d
180
170
160
150
140
130
120
110
100
90
80
70
60
50
40
30
20
10
0
f1 (ppm)

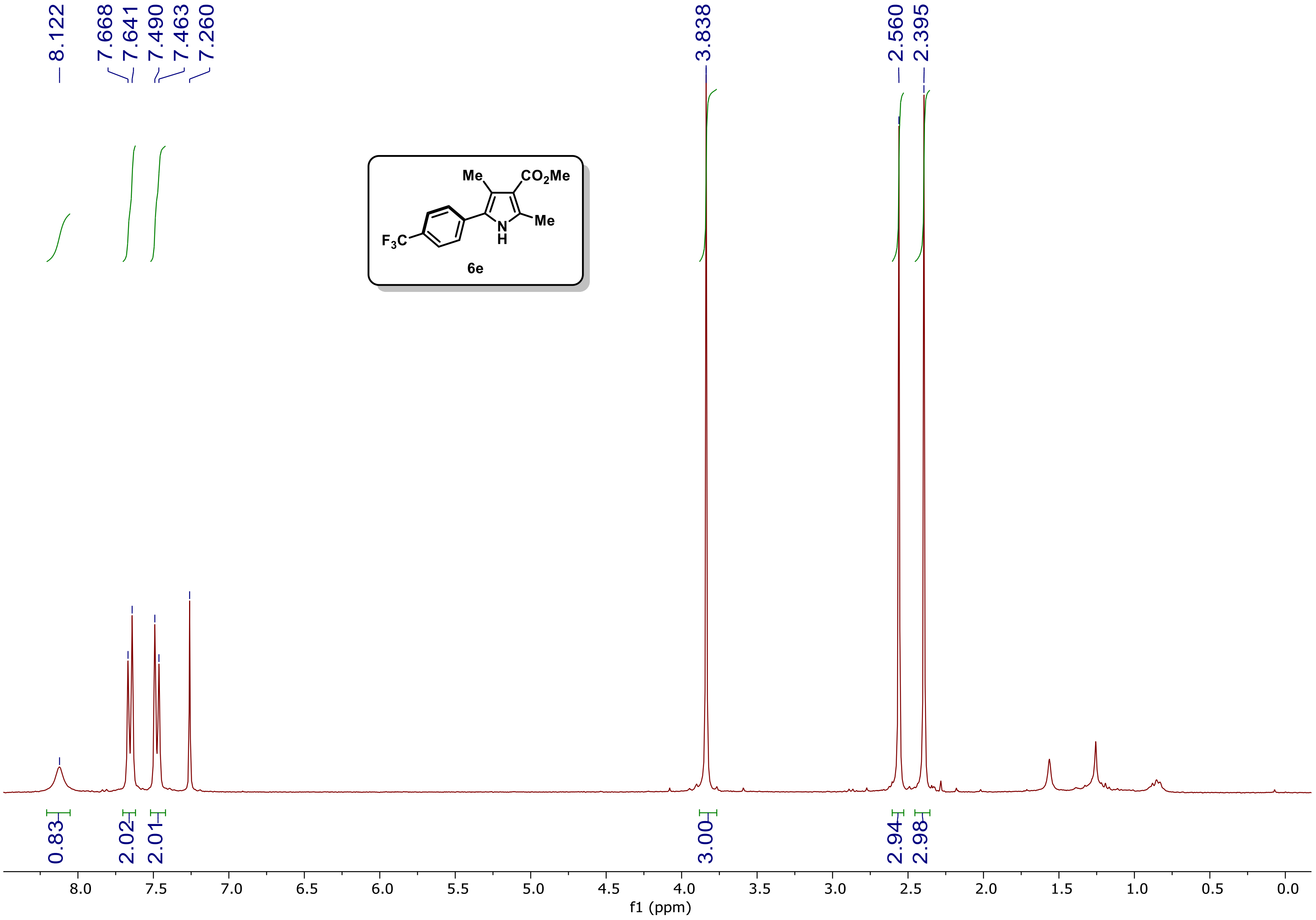

8.122
7.668
7.641
7.490
7.463
7.260
3.838
2.560
2.395
Me
CO2Me
Me
N
H
F3C
6e
0.83
2.02
2.01
3.00
2.94
2.98
8.0
7.5
7.0
6.5
6.0
5.5
5.0
4.5
4.0
3.5
3.0
2.5
2.0
1.5
1.0
0.5
0.0
f1 (ppm)

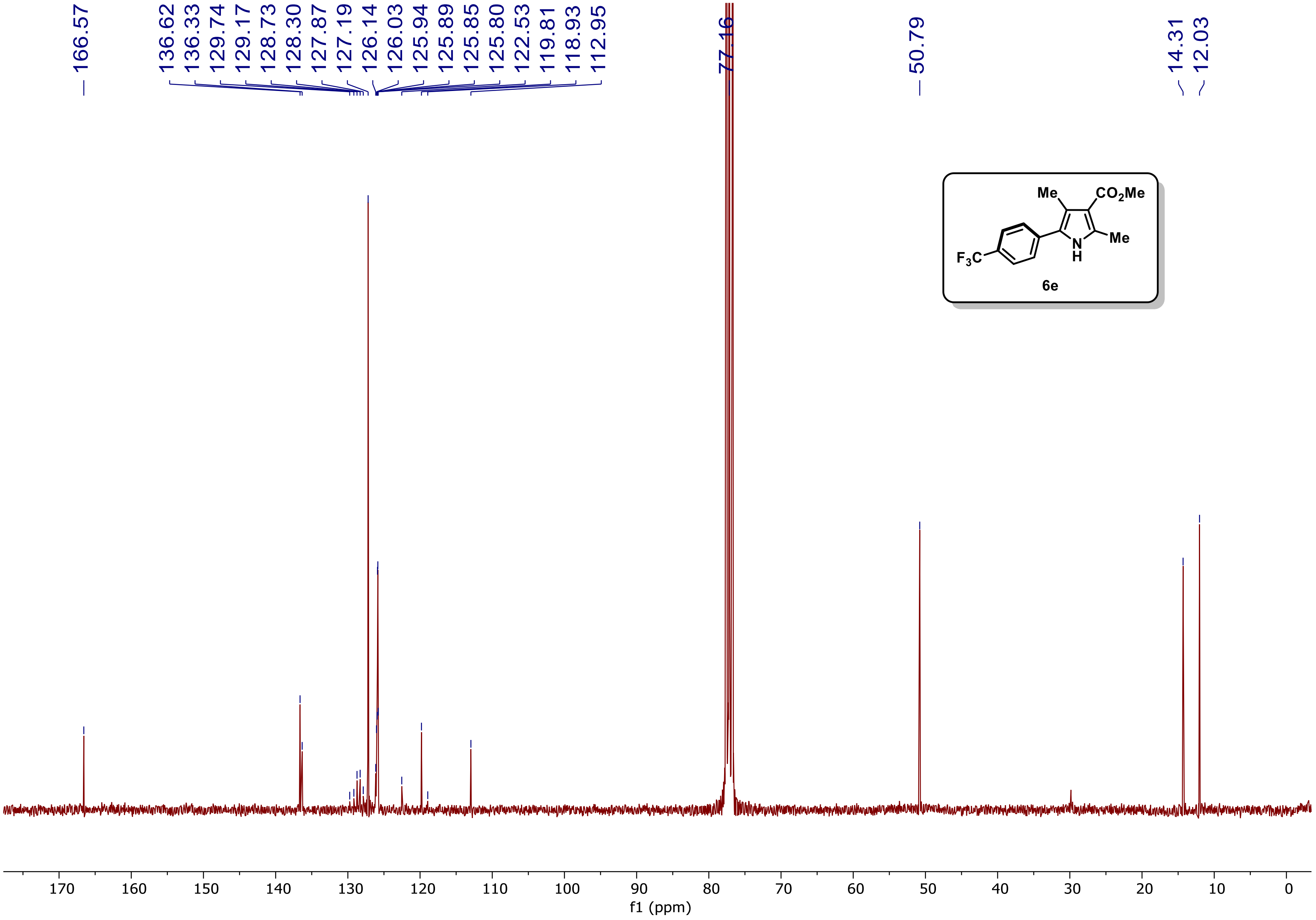
166.57
136.62
136.33
129.74
129.17
128.73
128.30
127.87
127.19
126.14
126.03
125.94
125.89
125.85
125.80
122.53
119.81
118.93
112.95
77.16
50.79
14.31
12.03
Me
CO_2Me
F_3C
N
H
Me
6e
170 160 150 140 130 120 110 100 90 80 70 60 50 40 30 20 10 0
f1 (ppm)

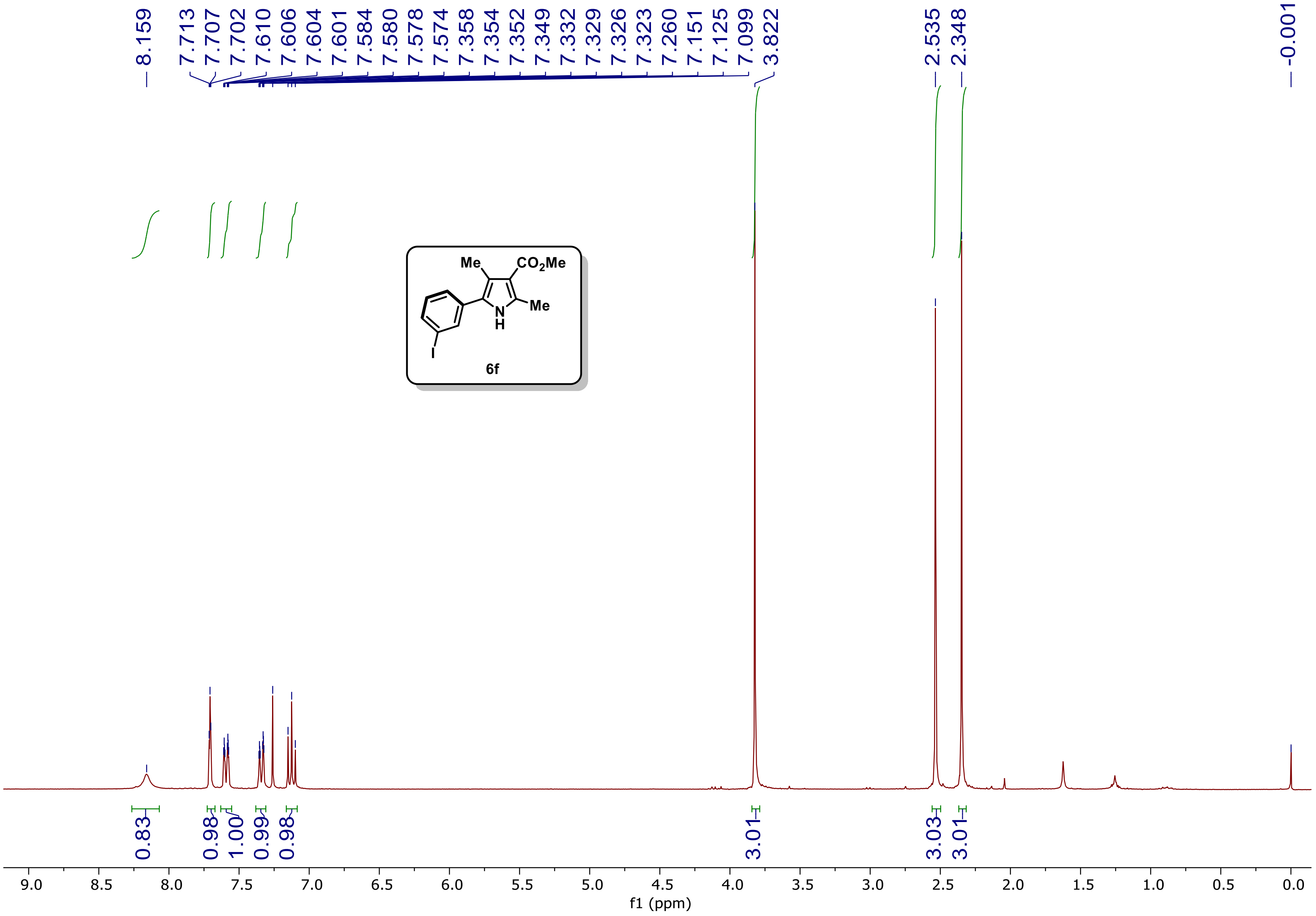
8.159
7.713
7.707
7.702
7.610
7.606
7.604
7.601
7.584
7.580
7.578
7.574
7.358
7.354
7.352
7.349
7.332
7.329
7.326
7.323
7.260
7.151
7.125
7.099
3.822
2.535
2.348
-0.001
Me
CO2Me
Me
N
H
I
6f
0.83
0.98
1.00
0.99
0.98
3.01
3.03
3.01
9.0
8.5
8.0
7.5
7.0
6.5
6.0
5.5
5.0
4.5
4.0
3.5
3.0
2.5
2.0
1.5
1.0
0.5
0.0
f1 (ppm)

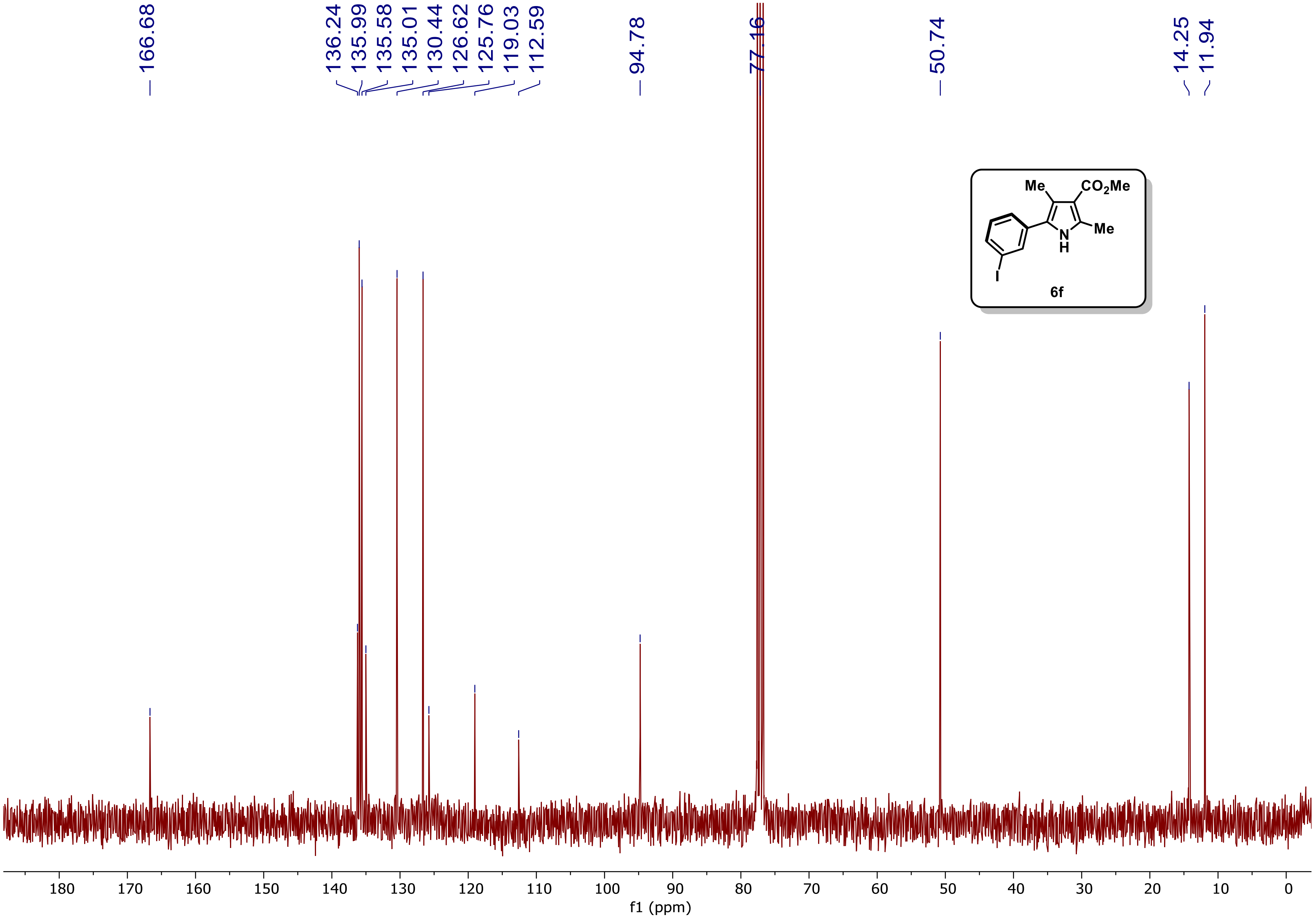
166.68
136.24
135.99
135.58
135.01
130.44
126.62
125.76
119.03
112.59
94.78
77.16
50.74
14.25
11.94
Me
CO_2Me
Me
N
H
I
6f
180
170
160
150
140
130
120
110
100
90
80
70
60
50
40
30
20
10
0
f1 (ppm)

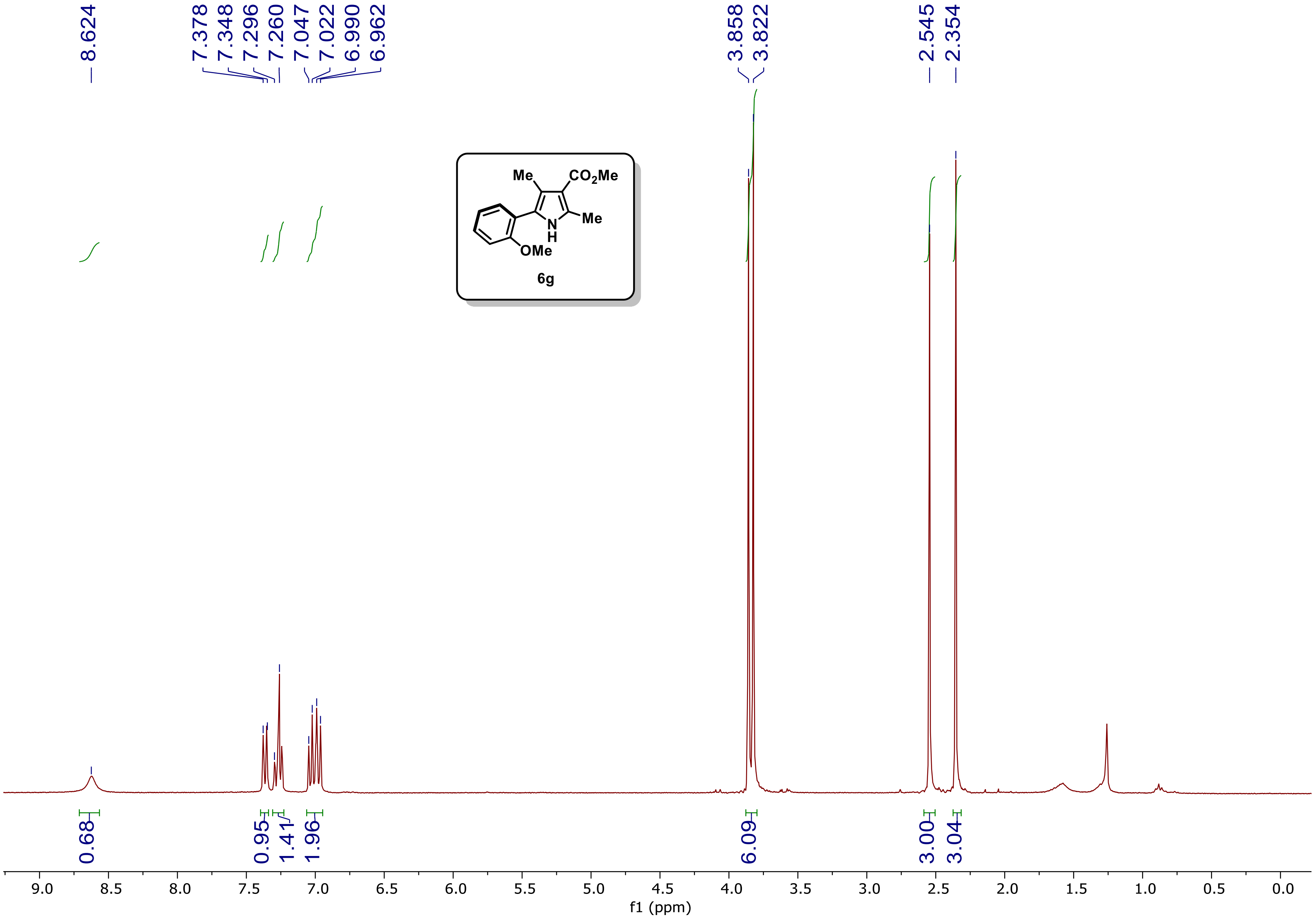
8.624
7.378
7.348
7.296
7.260
7.047
7.022
6.990
6.962
3.858
3.822
2.545
2.354
Me
CO2Me
Me
N
H
OMe
6g
0.68
0.95
1.41
1.96
6.09
3.00
3.04
9.0
8.5
8.0
7.5
7.0
6.5
6.0
5.5
5.0
4.5
4.0
3.5
3.0
2.5
2.0
1.5
1.0
0.5
0.0
f1 (ppm)

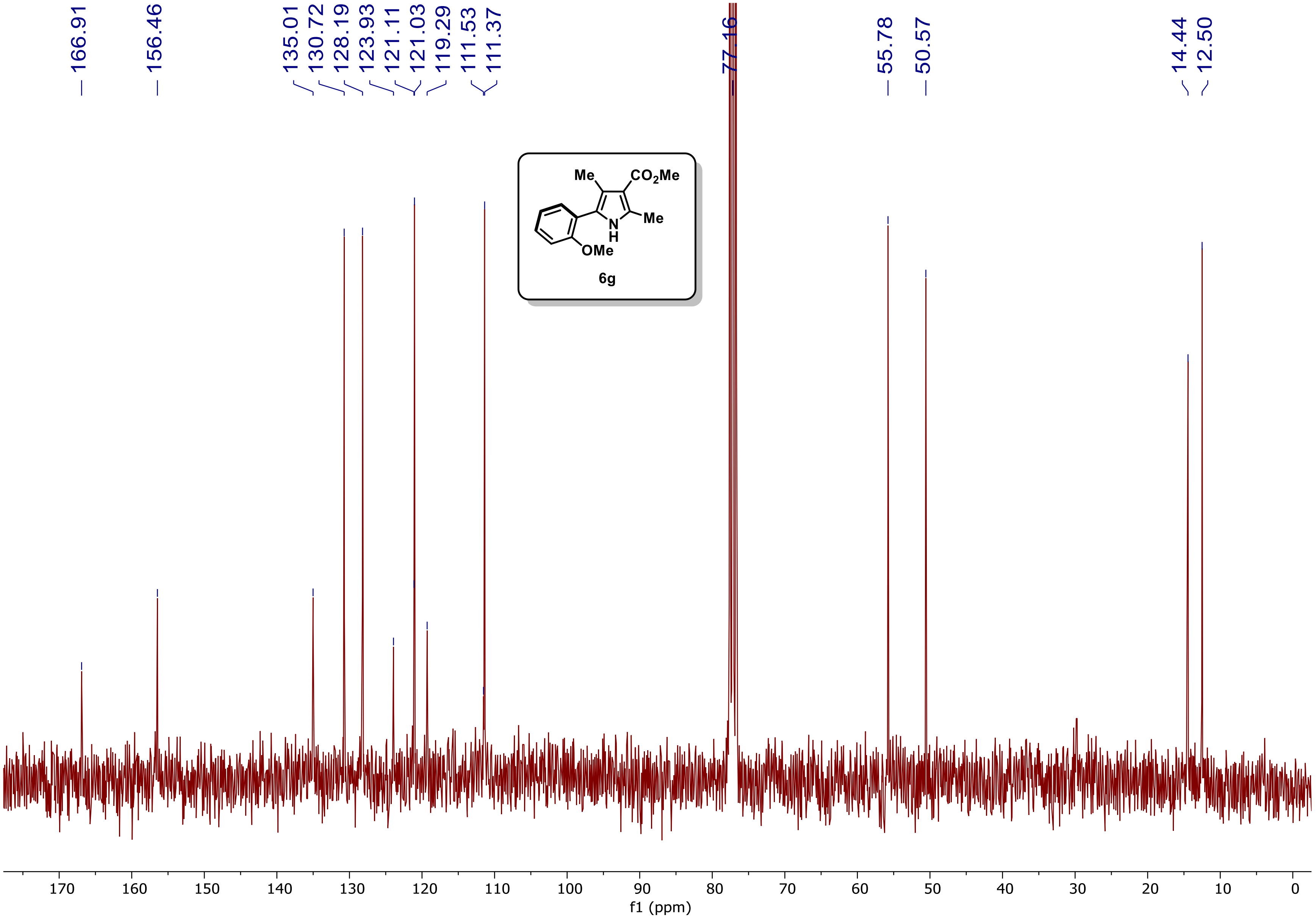

166.91
156.46
135.01
130.72
128.19
123.93
121.11
121.03
119.29
111.53
111.37
77.16
55.78
50.57
14.44
12.50
Me
CO2Me
Me
N
H
OMe
6g
170 160 150 140 130 120 110 100 90 80 70 60 50 40 30 20 10 0
f1 (ppm)

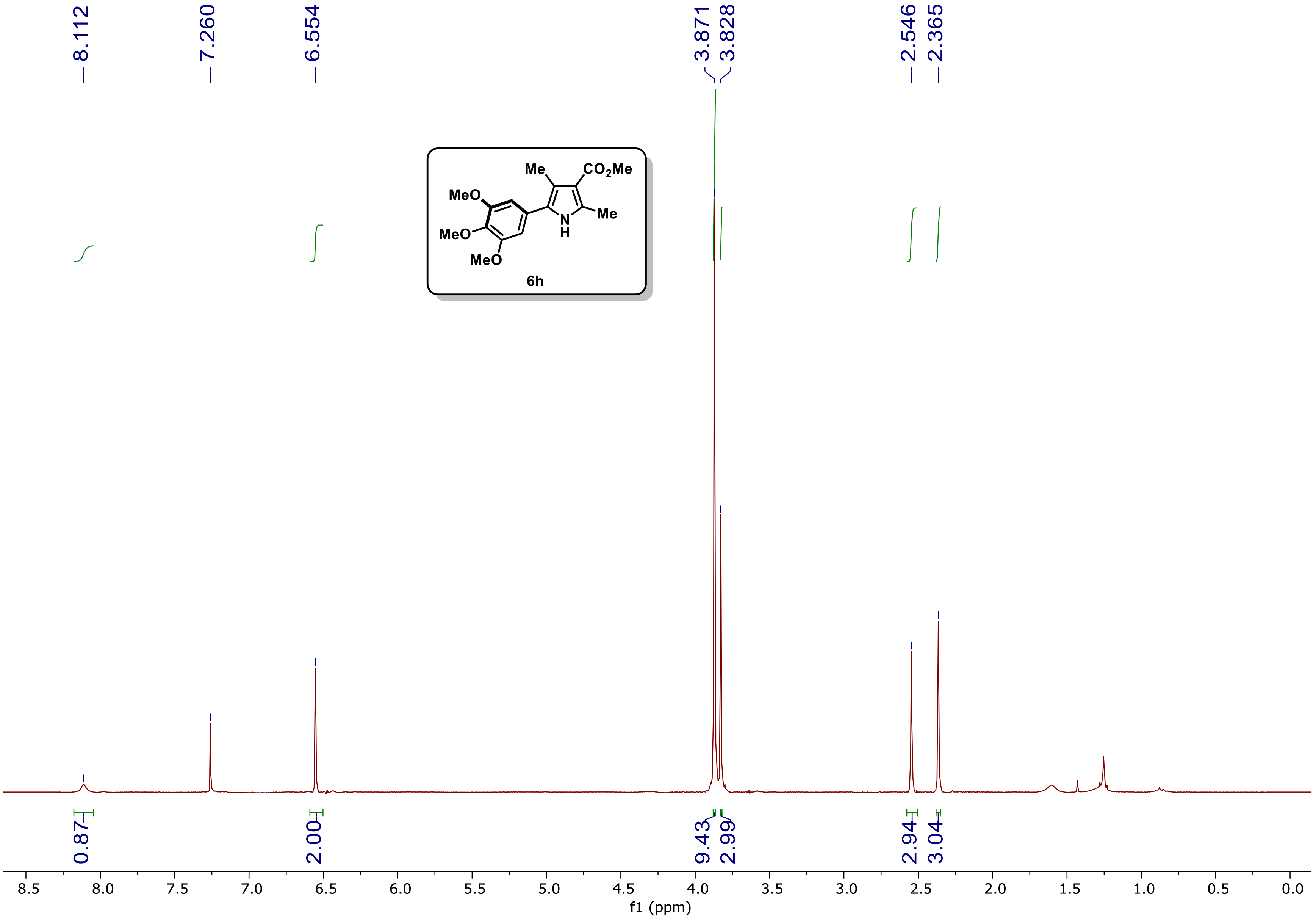

8.112
7.260
6.554
3.871
3.828
2.546
2.365
Me
CO2Me
MeO
MeO
MeO
N
H
Me
6h
0.87
2.00
9.43
2.99
2.94
3.04
8.5
8.0
7.5
7.0
6.5
6.0
5.5
5.0
4.5
4.0
3.5
3.0
2.5
2.0
1.5
1.0
0.5
0.0
f1 (ppm)

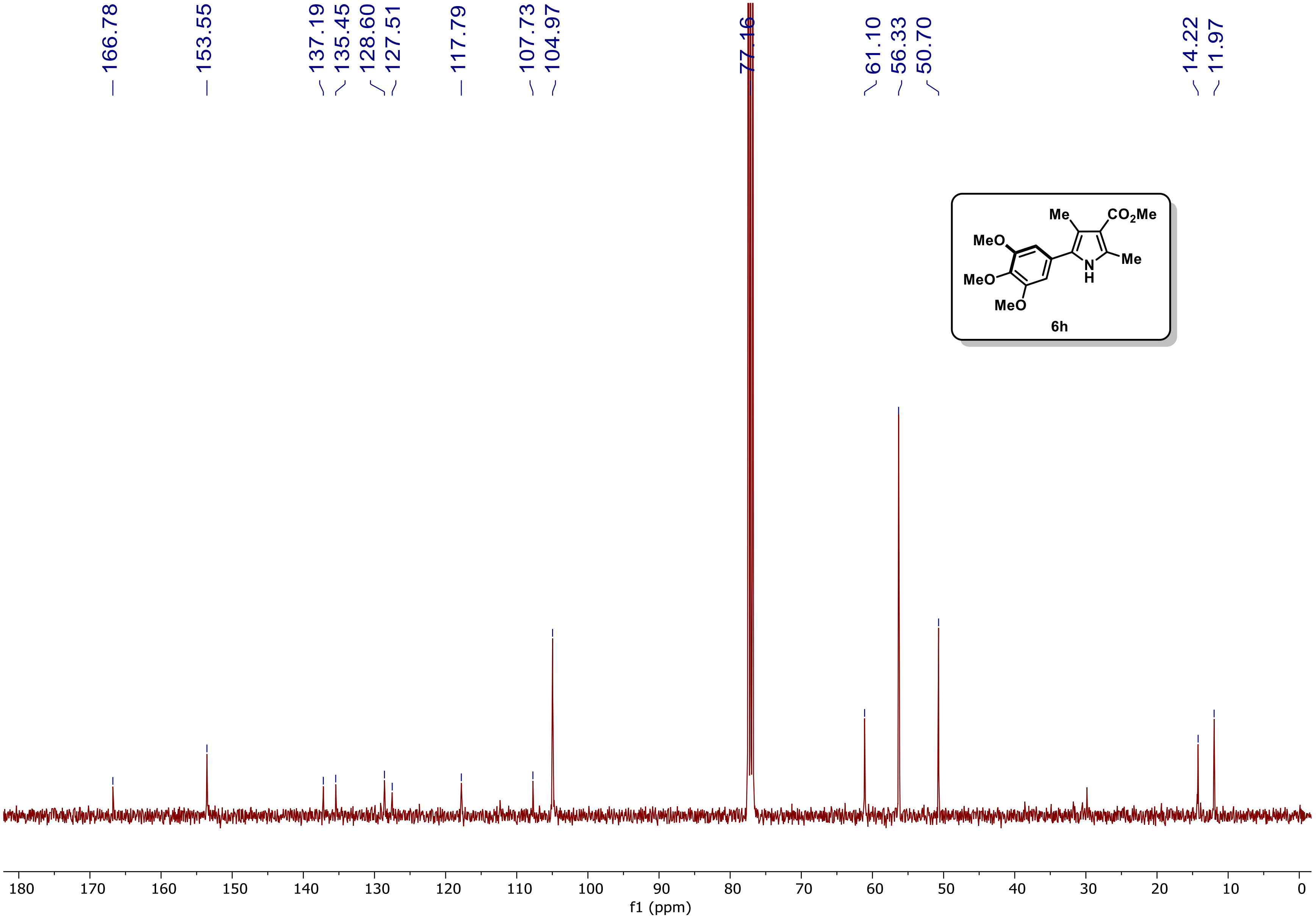
166.78
153.55
137.19
135.45
128.60
127.51
117.79
107.73
104.97
77.16
61.10
56.33
50.70
14.22
11.97
Me
CO2Me
MeO
MeO
MeO
N
H
Me
6h
180
170
160
150
140
130
120
110
100
90
80
70
60
50
40
30
20
10
0
f1 (ppm)

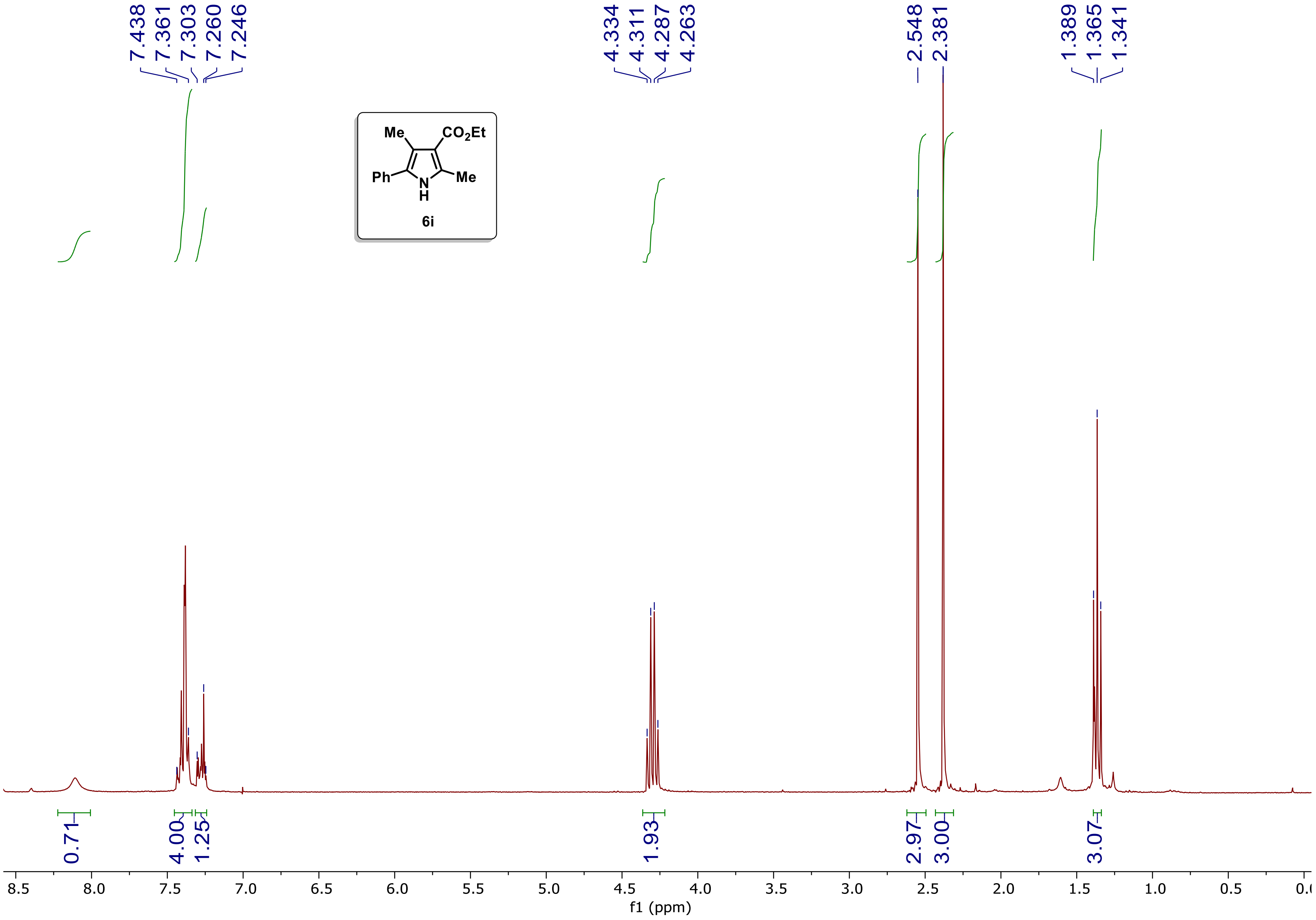

7.438
7.361
7.303
7.260
7.246
4.334
4.311
4.287
4.263
2.548
2.381
1.389
1.365
1.341
Me
CO2Et
Ph
N
H
Me
6i
0.71
4.00
1.25
1.93
2.97
3.00
3.07
8.5
8.0
7.5
7.0
6.5
6.0
5.5
5.0
4.5
4.0
3.5
3.0
2.5
2.0
1.5
1.0
0.5
f1 (ppm)

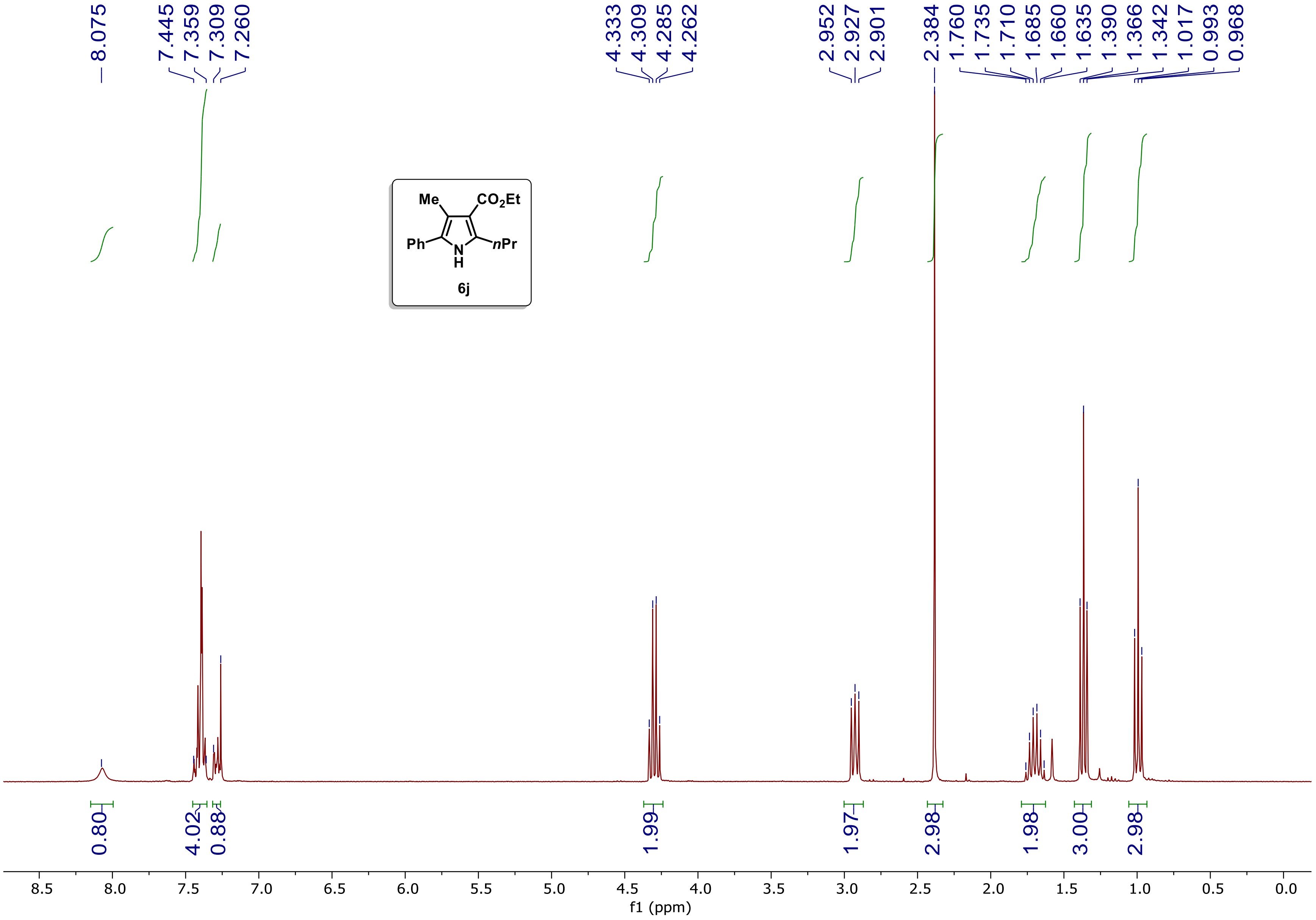
8.075
7.445
7.359
7.309
7.260
4.333
4.309
4.285
4.262
2.952
2.927
2.901
2.384
1.760
1.735
1.710
1.685
1.660
1.635
1.390
1.366
1.342
1.017
0.993
0.968
Me
CO2Et
Ph
N
H
nPr
6j
0.80
4.02
0.88
1.99
1.97
2.98
1.98
3.00
2.98
8.5
8.0
7.5
7.0
6.5
6.0
5.5
5.0
4.5
4.0
3.5
3.0
2.5
2.0
1.5
1.0
0.5
0.0
f1 (ppm)

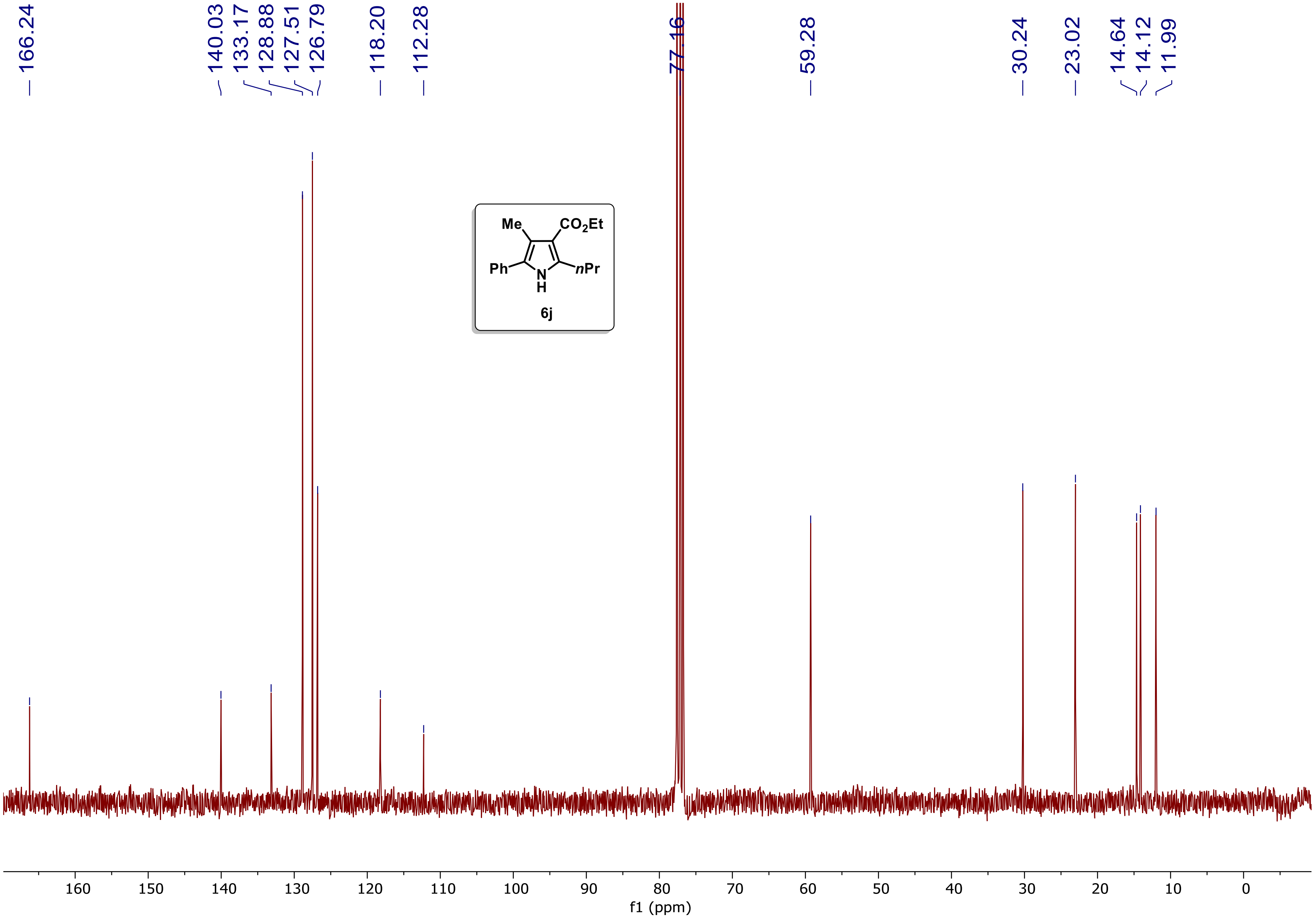
166.24
140.03
133.17
128.88
127.51
126.79
118.20
112.28
77.16
59.28
30.24
23.02
14.64
14.12
11.99
Me
CO2Et
Ph
N
H
nPr
6j
160
150
140
130
120
110
100
90
80
70
60
50
40
30
20
10
0
f1 (ppm)

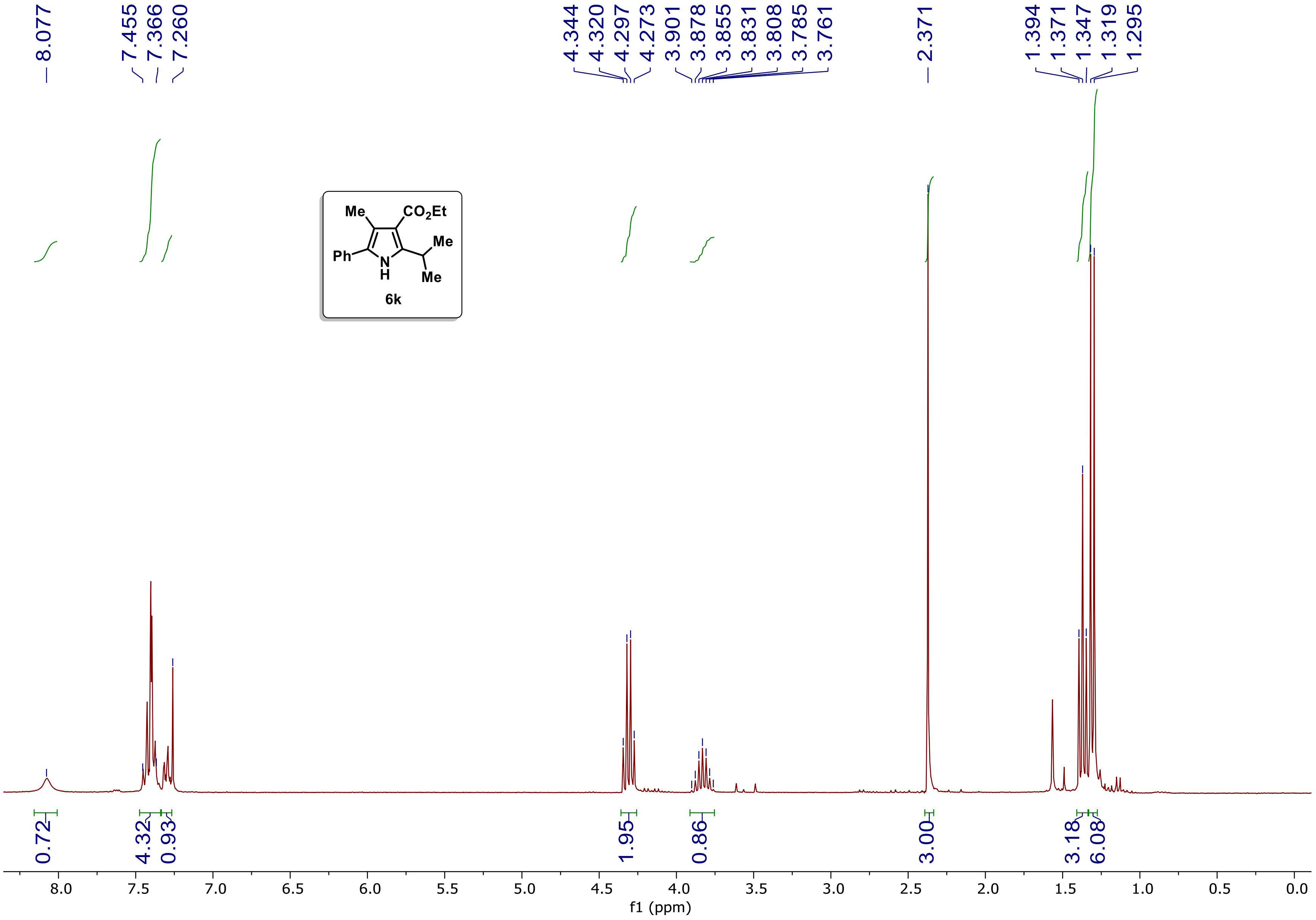
8.077
7.455
7.366
7.260
4.344
4.320
4.297
4.273
3.901
3.878
3.855
3.831
3.808
3.785
3.761
2.371
1.394
1.371
1.347
1.319
1.295
Me
CO2Et
Me
Ph
N
H
Me
6k
0.72
4.32
0.93
1.95
0.86
3.00
3.18
6.08
8.0
7.5
7.0
6.5
6.0
5.5
5.0
4.5
4.0
3.5
3.0
2.5
2.0
1.5
1.0
0.5
0.0
f1 (ppm)

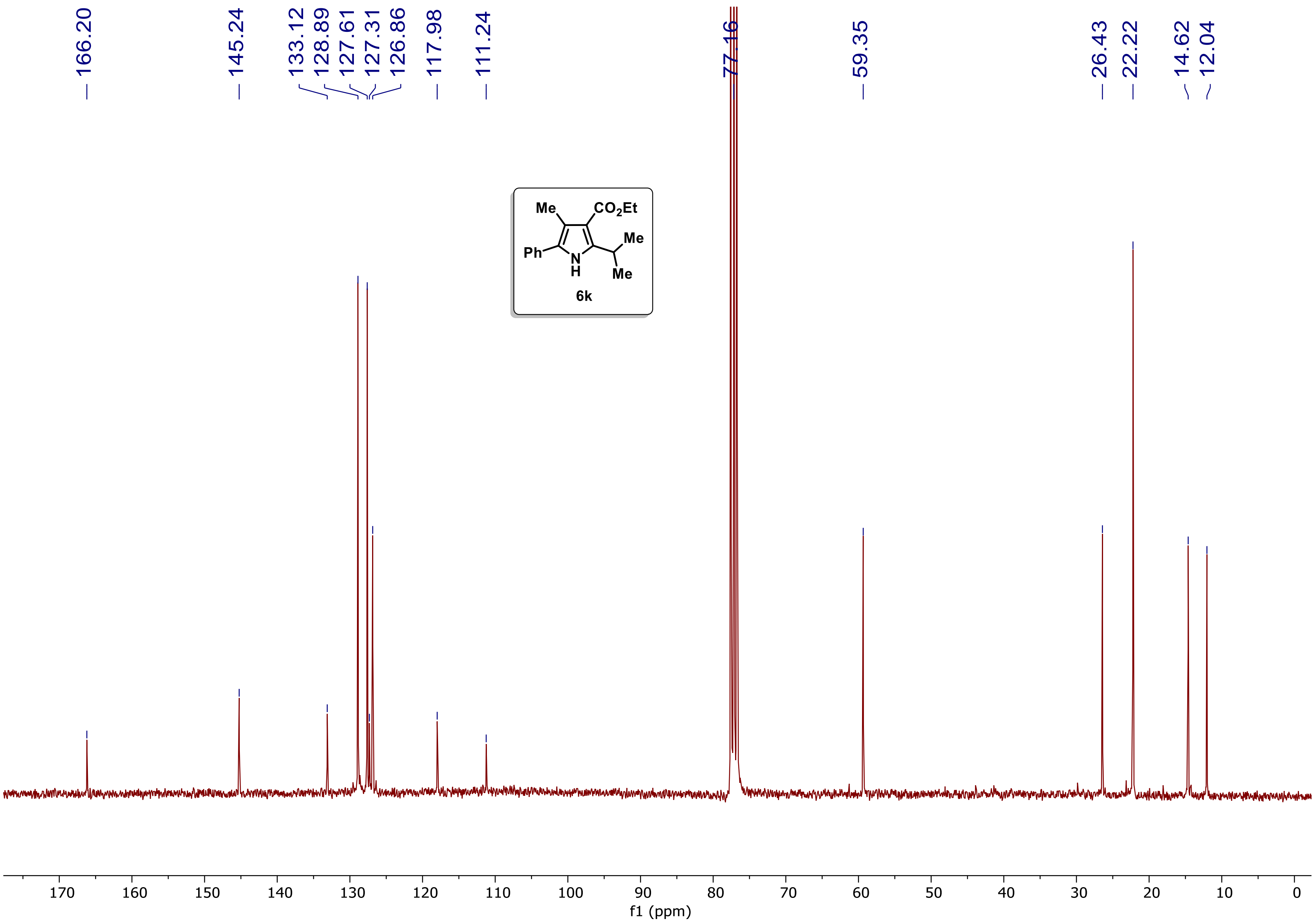
166.20
145.24
133.12
128.89
127.61
127.31
126.86
117.98
111.24
77.16
59.35
26.43
22.22
14.62
12.04
Me
CO2Et
Ph
N
H
Me
Me
6k
170
160
150
140
130
120
110
100
90
80
70
60
50
40
30
20
10
0
f1 (ppm)

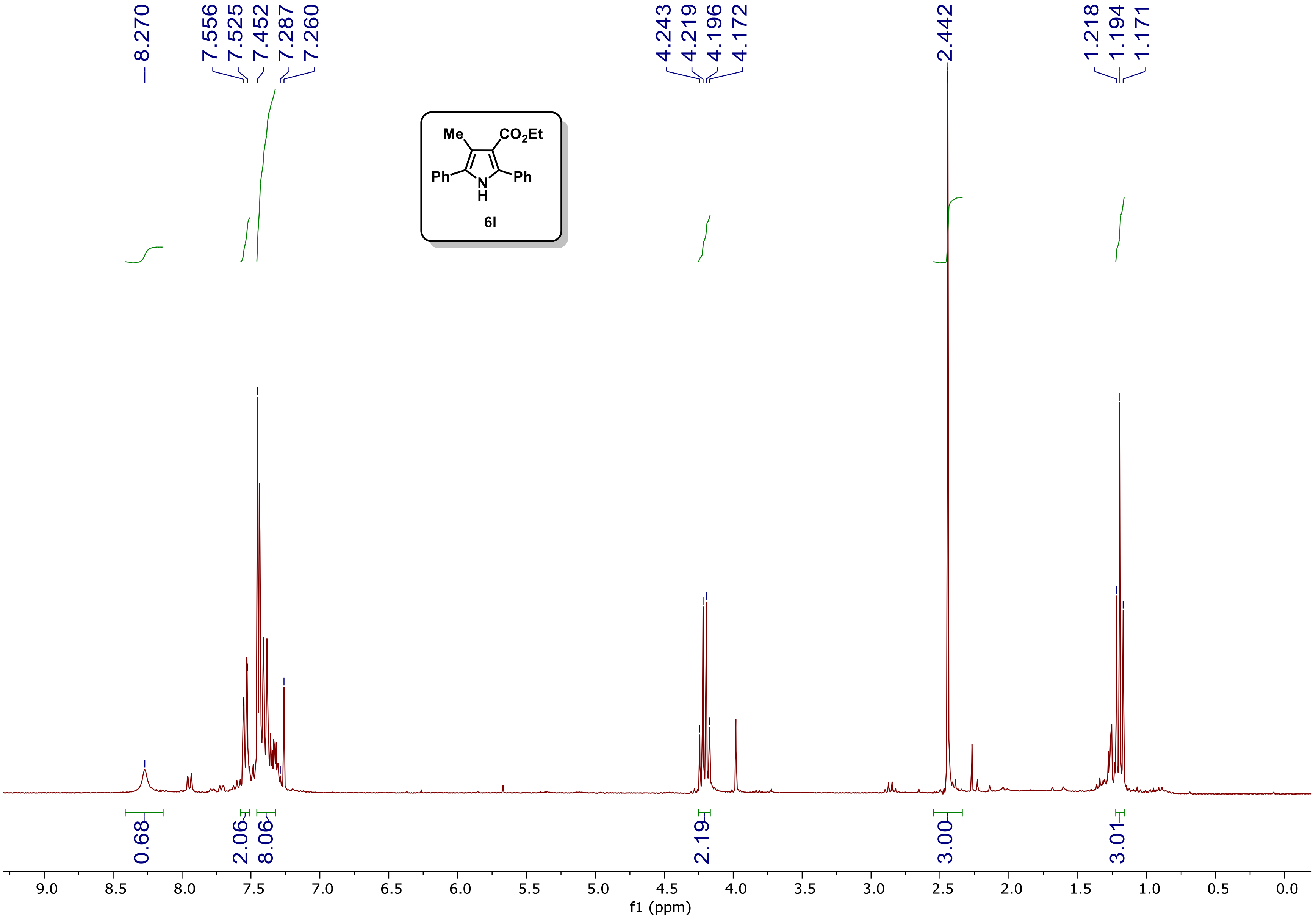
8.270
7.556
7.525
7.452
7.287
7.260
4.243
4.219
4.196
4.172
2.442
1.218
1.194
1.171
Me
CO2Et
Ph
N
H
Ph
6l
0.68
2.06
8.06
2.19
3.00
3.01
9.0 8.5 8.0 7.5 7.0 6.5 6.0 5.5 5.0 4.5 4.0 3.5 3.0 2.5 2.0 1.5 1.0 0.5 0.0
f1 (ppm)

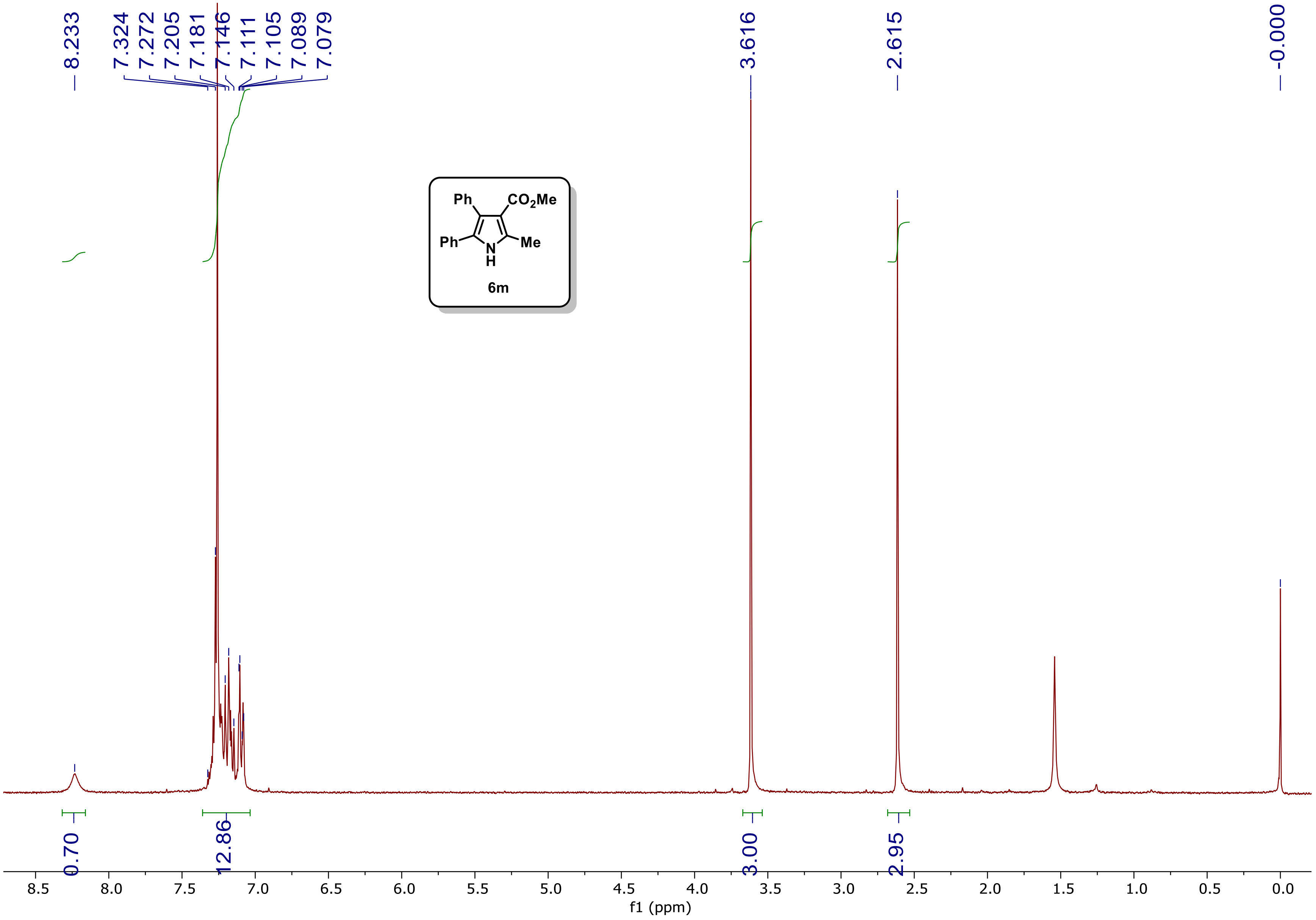
8.233
7.324
7.272
7.205
7.181
7.146
7.111
7.105
7.089
7.079
3.616
2.615
-0.000
Ph
CO_2Me
Ph
Me
N
H
6m
0.70
12.86
3.00
2.95
8.5 8.0 7.5 7.0 6.5 6.0 5.5 5.0 4.5 4.0 3.5 3.0 2.5 2.0 1.5 1.0 0.5 0.0
f1 (ppm)

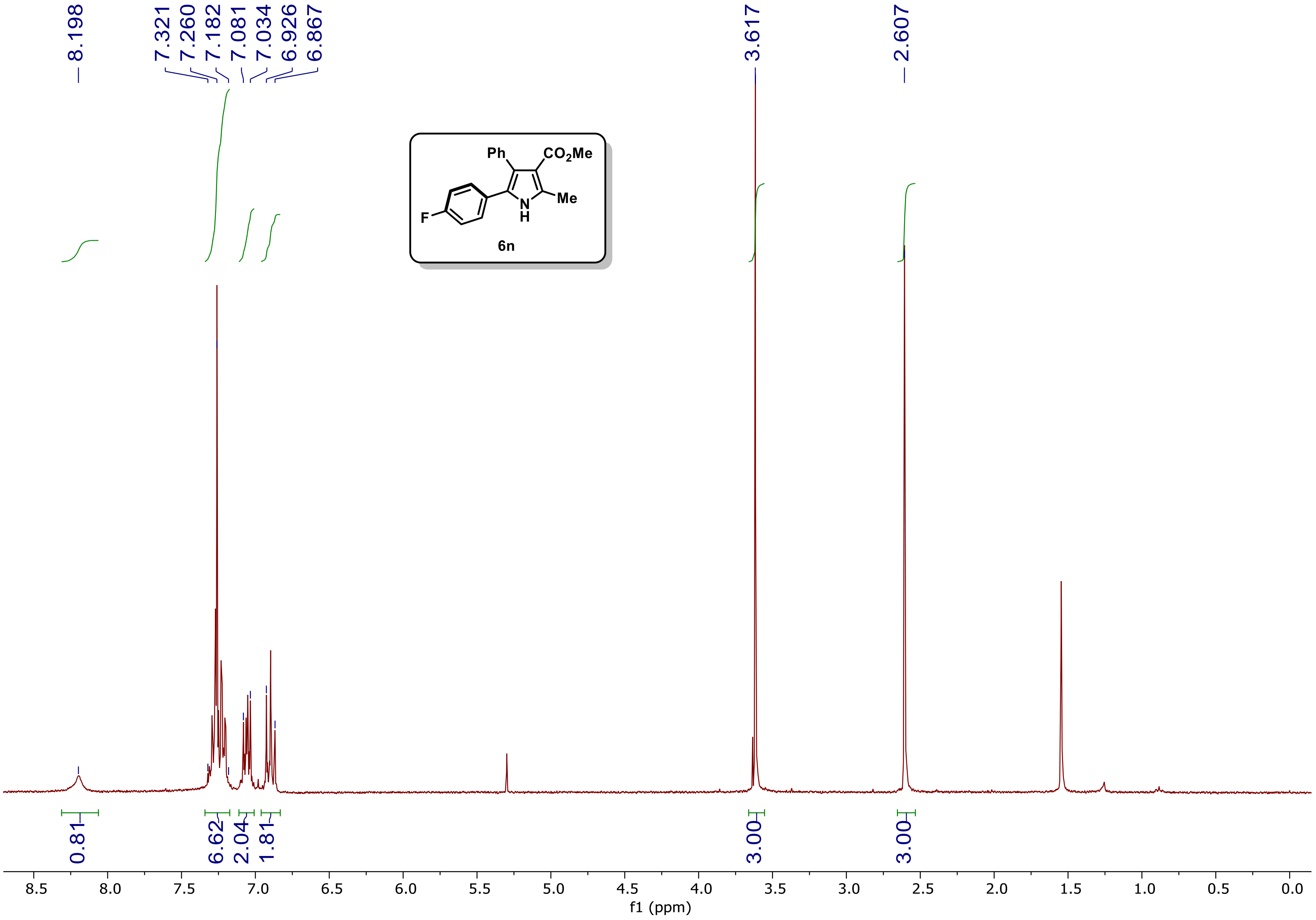
8.198
7.321
7.260
7.182
7.081
7.034
6.926
6.867
3.617
2.607
Ph
CO2Me
Me
F
N
H
6n
0.81
6.62
2.04
1.81
3.00
3.00
8.5 8.0 7.5 7.0 6.5 6.0 5.5 5.0 4.5 4.0 3.5 3.0 2.5 2.0 1.5 1.0 0.5 0.0
f1 (ppm)

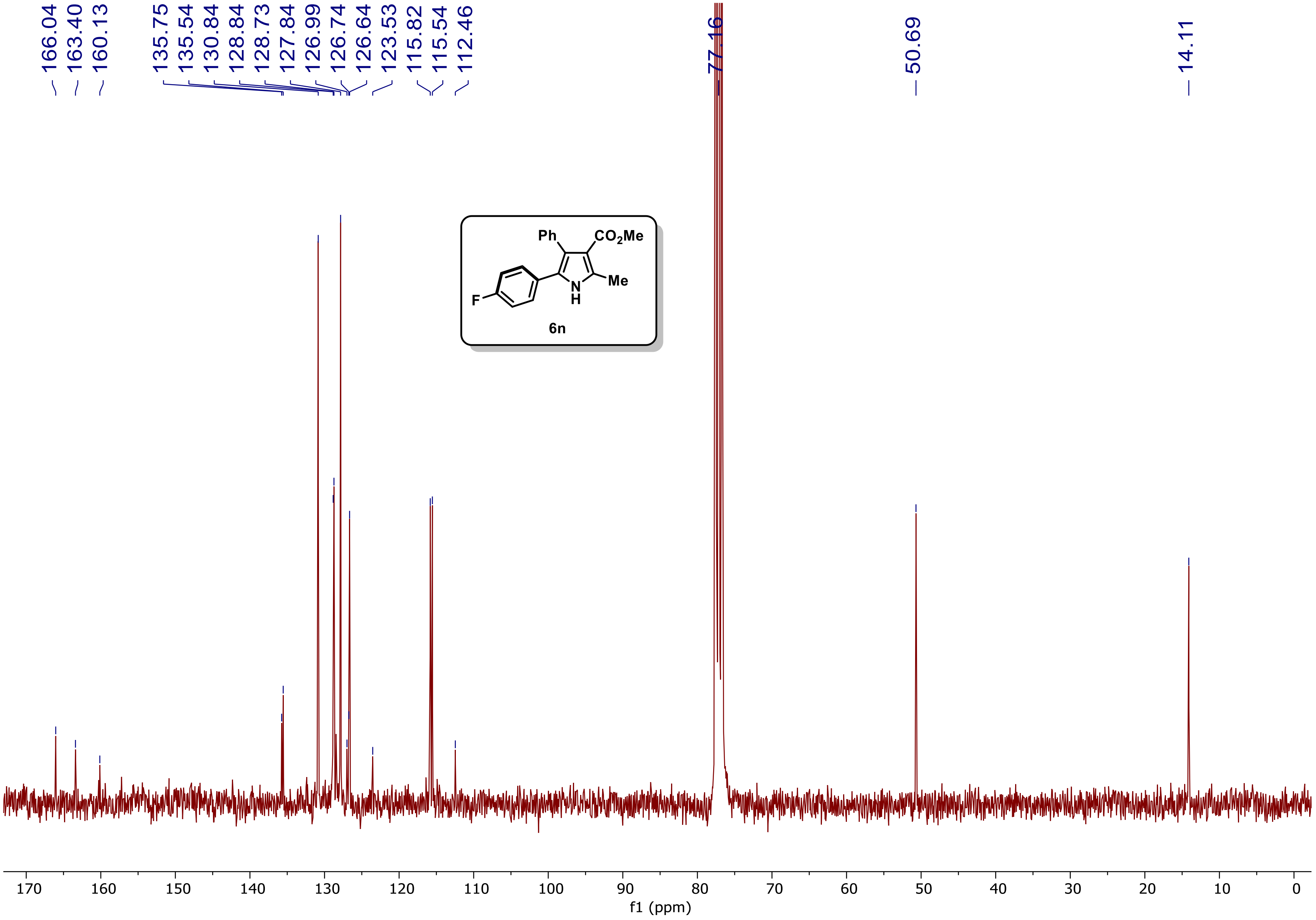
166.04
163.40
160.13
135.75
135.54
130.84
128.84
128.73
127.84
126.99
126.74
126.64
123.53
115.82
115.54
112.46
77.16
50.69
14.11
Ph
CO2Me
Me
F
N
H
6n
170 160 150 140 130 120 110 100 90 80 70 60 50 40 30 20 10 0
f1 (ppm)

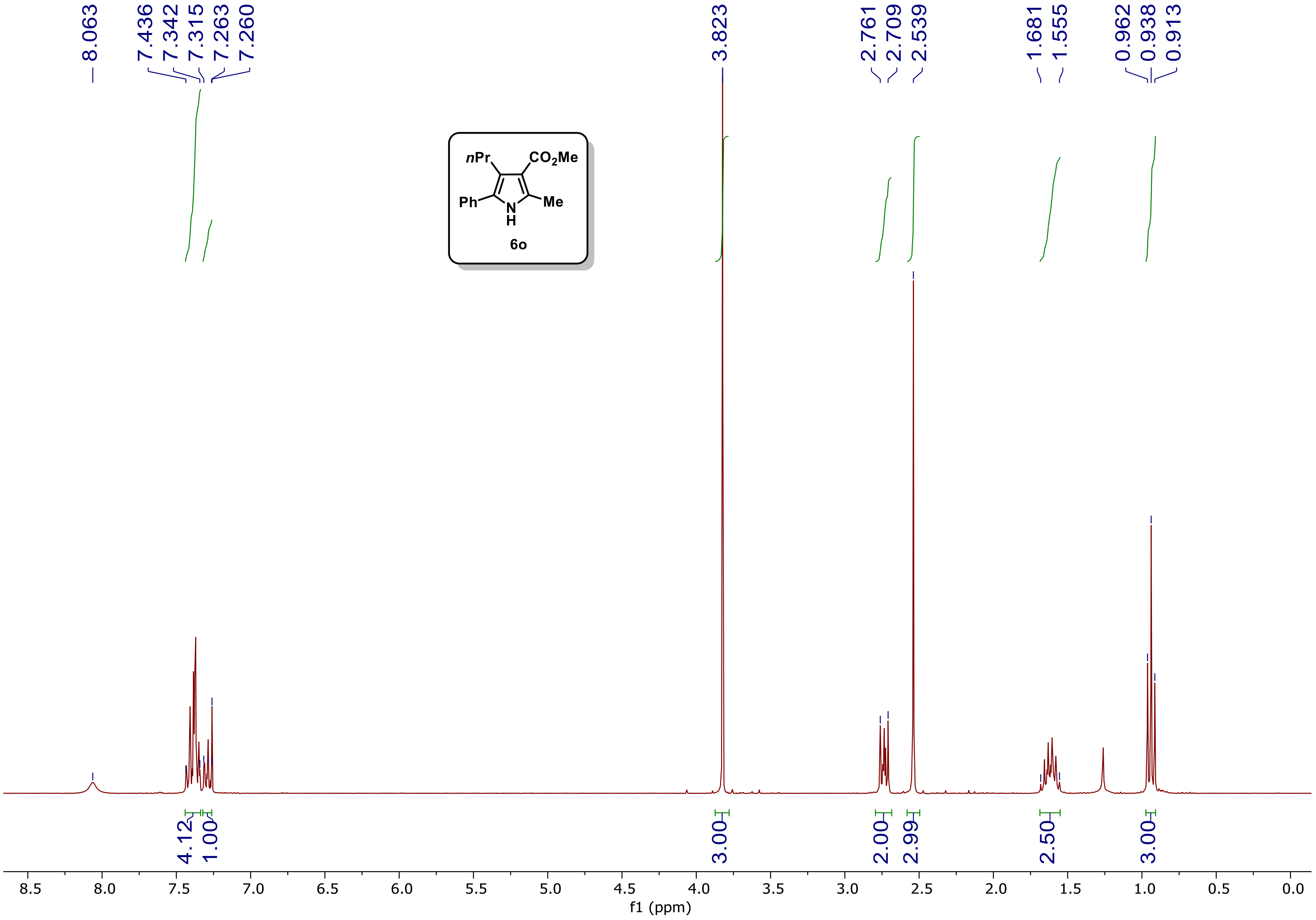
8.063
7.436
7.342
7.315
7.263
7.260
3.823
2.761
2.709
2.539
1.681
1.555
0.962
0.938
0.913
nPr
CO2Me
Ph
N
H
Me
6o
4.12
1.00
3.00
2.00
2.99
2.50
3.00
8.5
8.0
7.5
7.0
6.5
6.0
5.5
5.0
4.5
4.0
3.5
3.0
2.5
2.0
1.5
1.0
0.5
0.0
f1 (ppm)

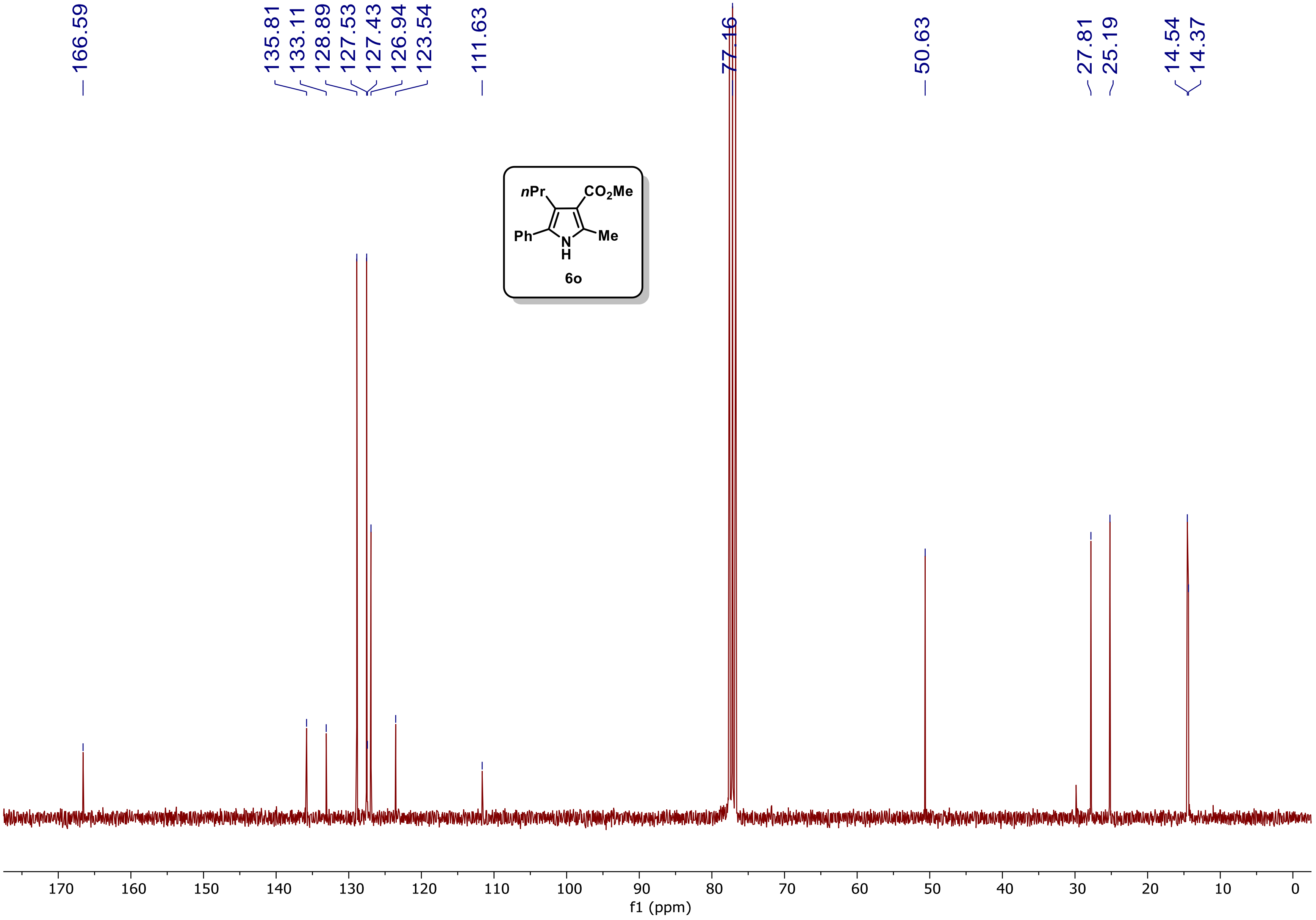

166.59
135.81
133.11
128.89
127.53
127.43
126.94
123.54
111.63
77.16
50.63
27.81
25.19
14.54
14.37
nPr
CO2Me
Ph
N
H
Me
6o
170
160
150
140
130
120
110
100
90
80
70
60
50
40
30
20
10
0
f1 (ppm)

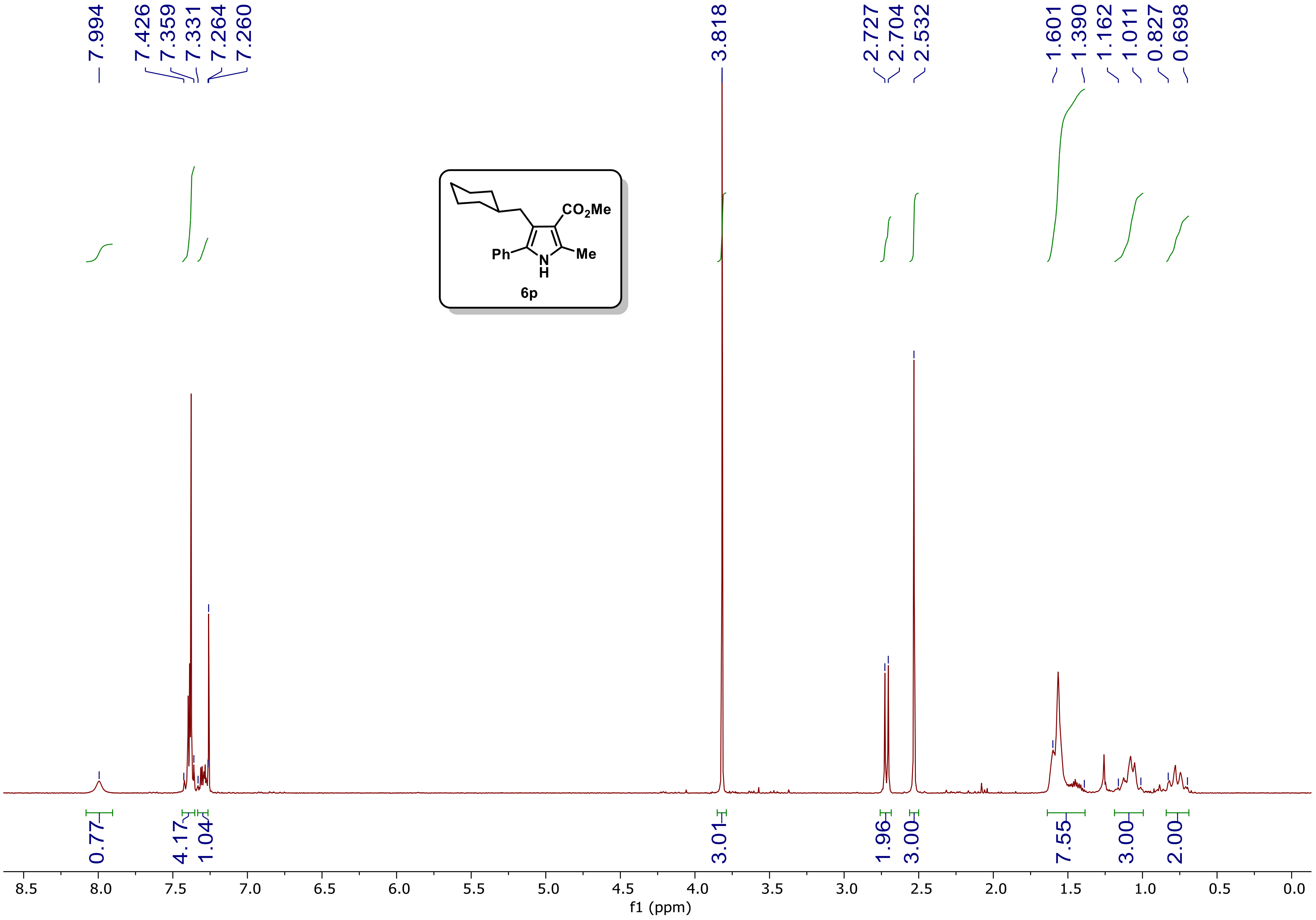
7.994
7.426
7.359
7.331
7.264
7.260
3.818
2.727
2.704
2.532
1.601
1.390
1.162
1.011
0.827
0.698
CO2Me
Ph
N
H
Me
6p
0.77
4.17
1.04
3.01
1.96
3.00
7.55
3.00
2.00
8.5
8.0
7.5
7.0
6.5
6.0
5.5
5.0
4.5
4.0
3.5
3.0
2.5
2.0
1.5
1.0
0.5
0.0
f1 (ppm)

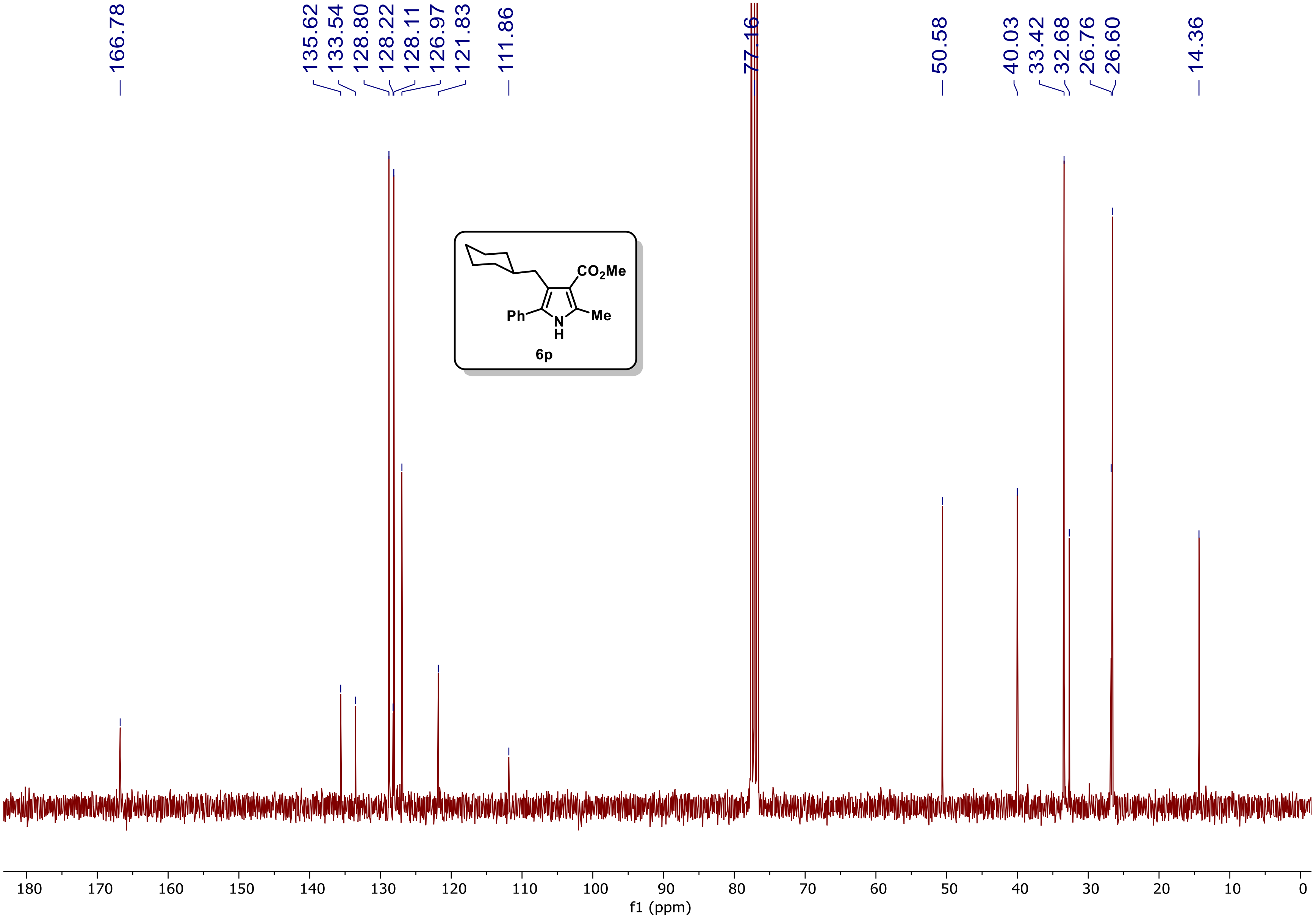
166.78
135.62
133.54
128.80
128.22
128.11
126.97
121.83
111.86
77.16
50.58
40.03
33.42
32.68
26.76
26.60
14.36
CO2Me
Ph
N
H
Me
6p
180 170 160 150 140 130 120 110 100 90 80 70 60 50 40 30 20 10 0
f1 (ppm)

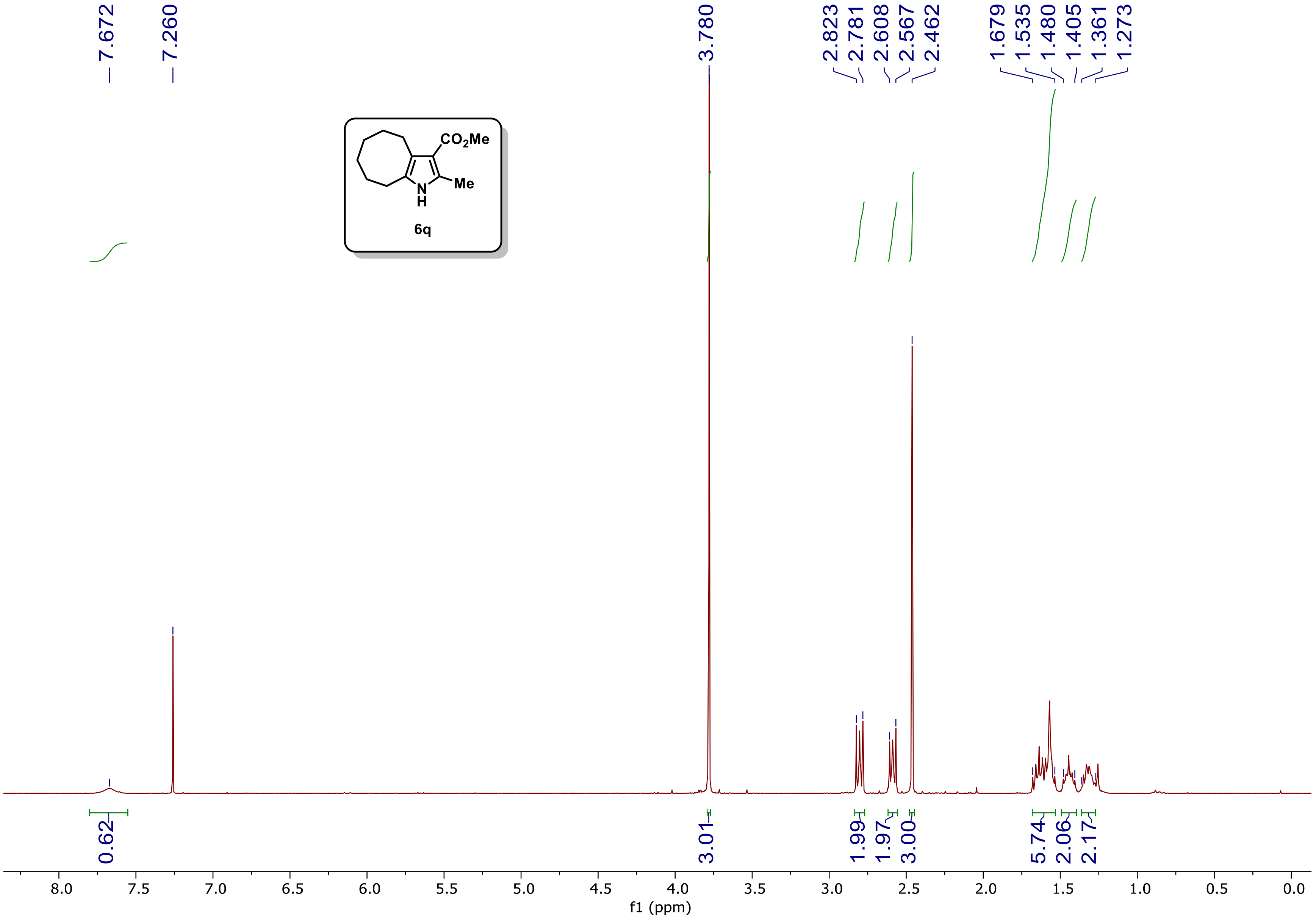
7.672
7.260
3.780
2.823
2.781
2.608
2.567
2.462
1.679
1.535
1.480
1.405
1.361
1.273
CO2Me
Me
N
H
6q
0.62
3.01
1.99
1.97
3.00
5.74
2.06
2.17
8.0 7.5 7.0 6.5 6.0 5.5 5.0 4.5 4.0 3.5 3.0 2.5 2.0 1.5 1.0 0.5 0.0
f1 (ppm)

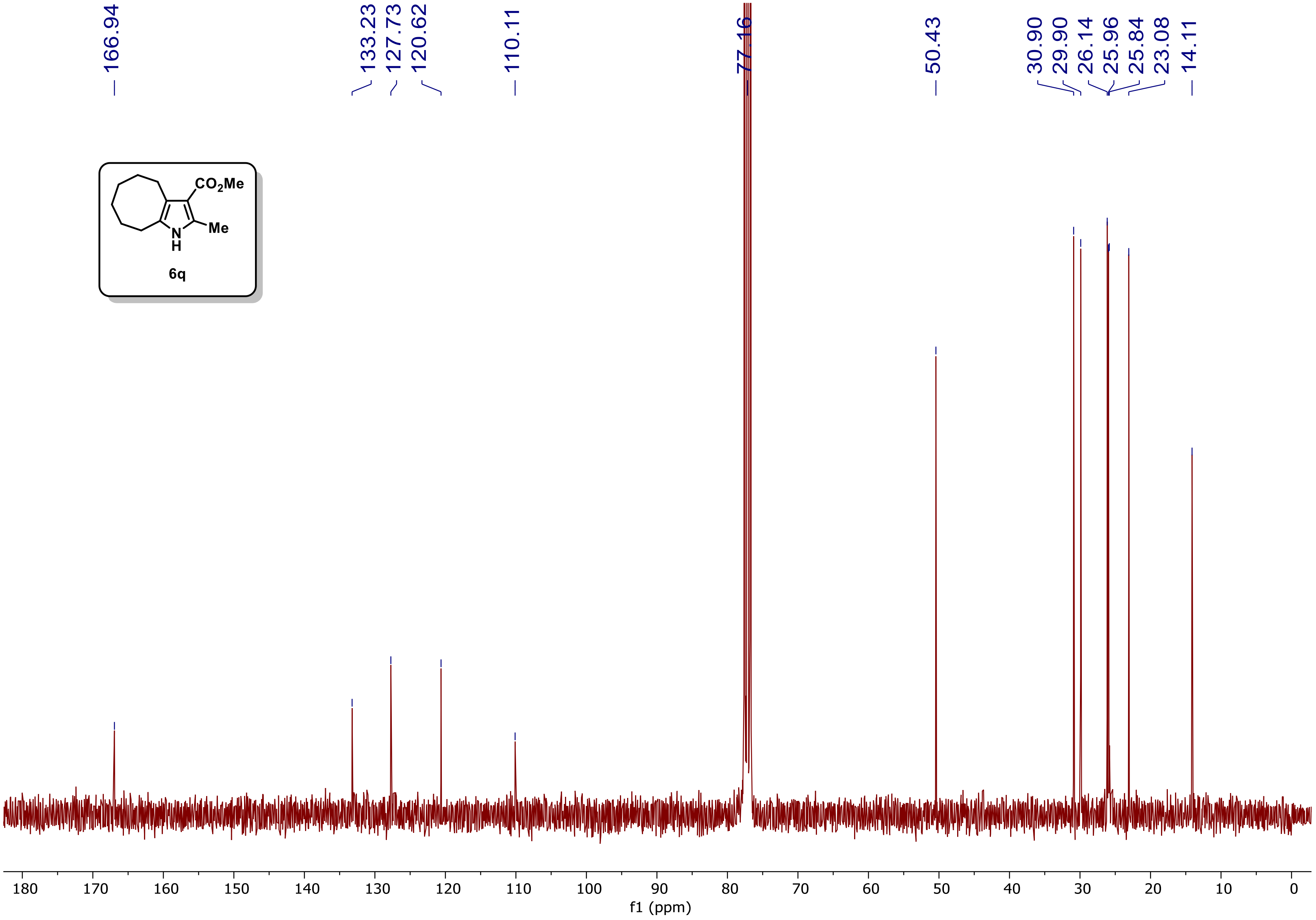

166.94
133.23
127.73
120.62
110.11
77.16
50.43
30.90
29.90
26.14
25.96
25.84
23.08
14.11
CO2Me
Me
N
H
6q
180
170
160
150
140
130
120
110
100
90
80
70
60
50
40
30
20
10
0
f1 (ppm)

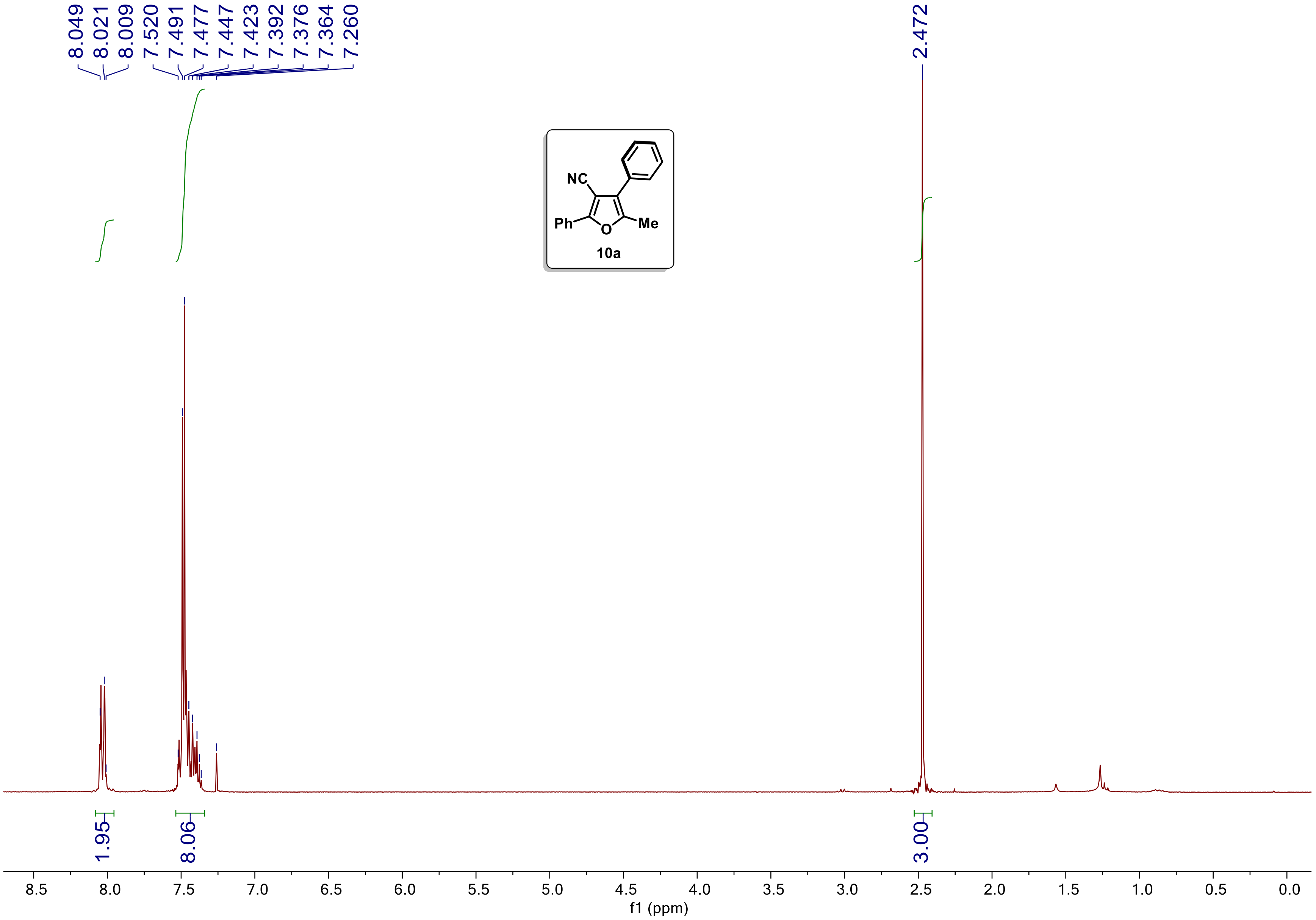
8.049
8.021
8.009
7.520
7.491
7.477
7.447
7.423
7.392
7.376
7.364
7.260
2.472
NC
Ph
O
Me
10a
1.95
8.06
3.00
8.5
8.0
7.5
7.0
6.5
6.0
5.5
5.0
4.5
4.0
3.5
3.0
2.5
2.0
1.5
1.0
0.5
0.0
f1 (ppm)

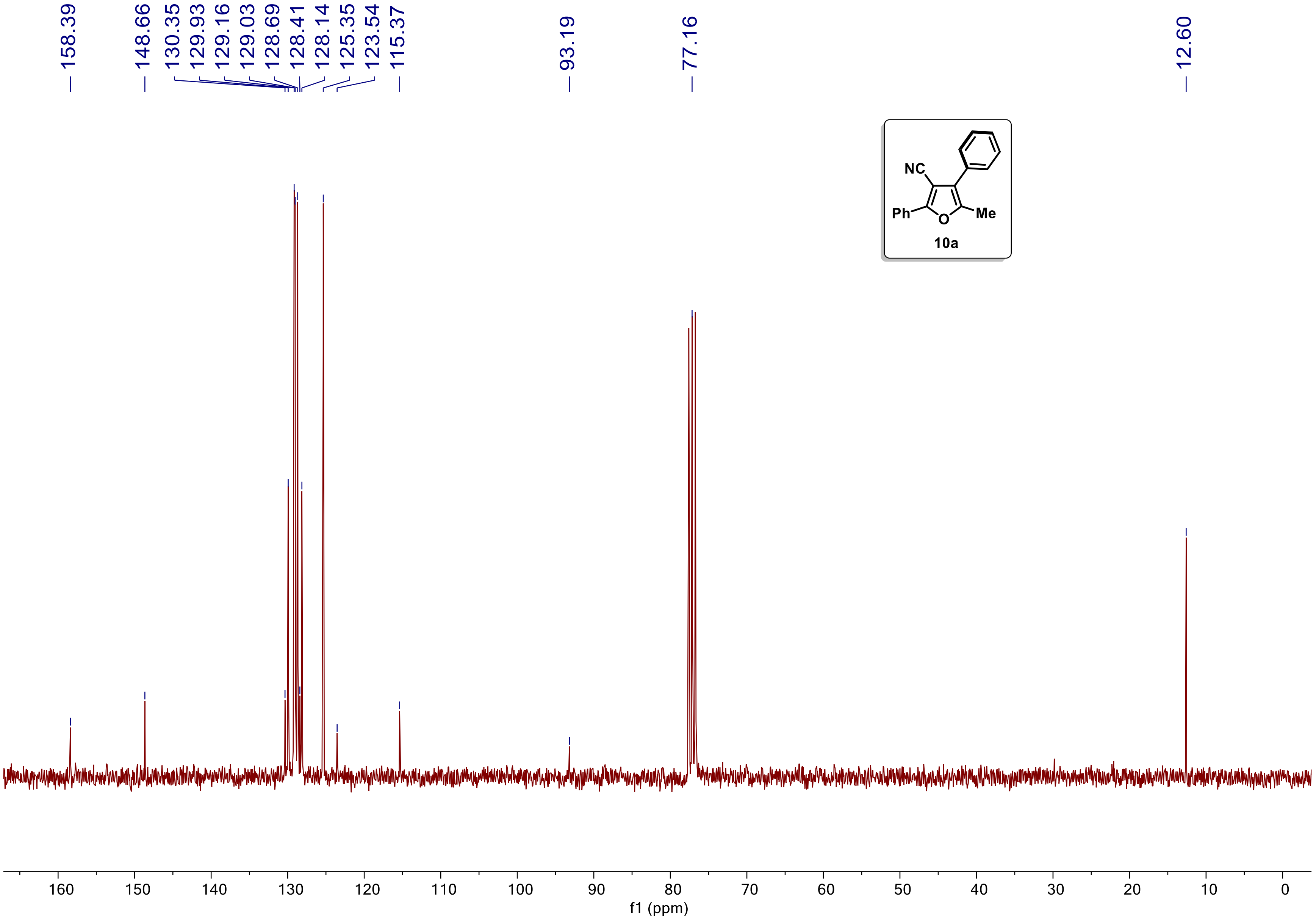

158.39
148.66
130.35
129.93
129.16
129.03
128.69
128.41
128.14
125.35
123.54
115.37
93.19
77.16
12.60
NC
Ph
O
Me
10a
160
150
140
130
120
110
100
90
80
70
60
50
40
30
20
10
0
f1 (ppm)

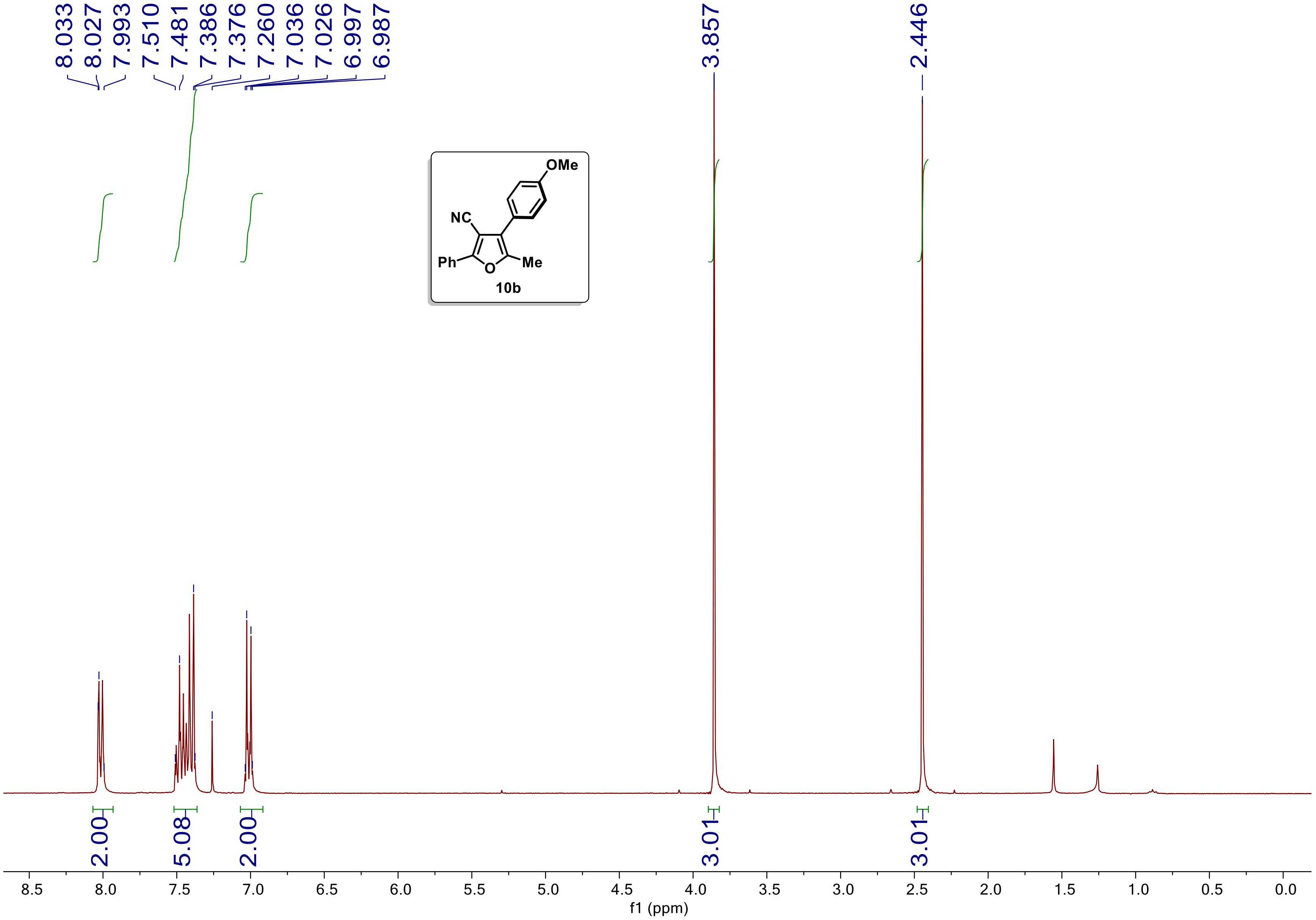
8.033
8.027
7.993
7.510
7.481
7.386
7.376
7.260
7.036
7.026
6.997
6.987
3.857
2.446
OMe
NC
Ph
O
Me
10b
2.00
5.08
2.00
3.01
3.01
8.5
8.0
7.5
7.0
6.5
6.0
5.5
5.0
4.5
4.0
3.5
3.0
2.5
2.0
1.5
1.0
0.5
0.0
f1 (ppm)

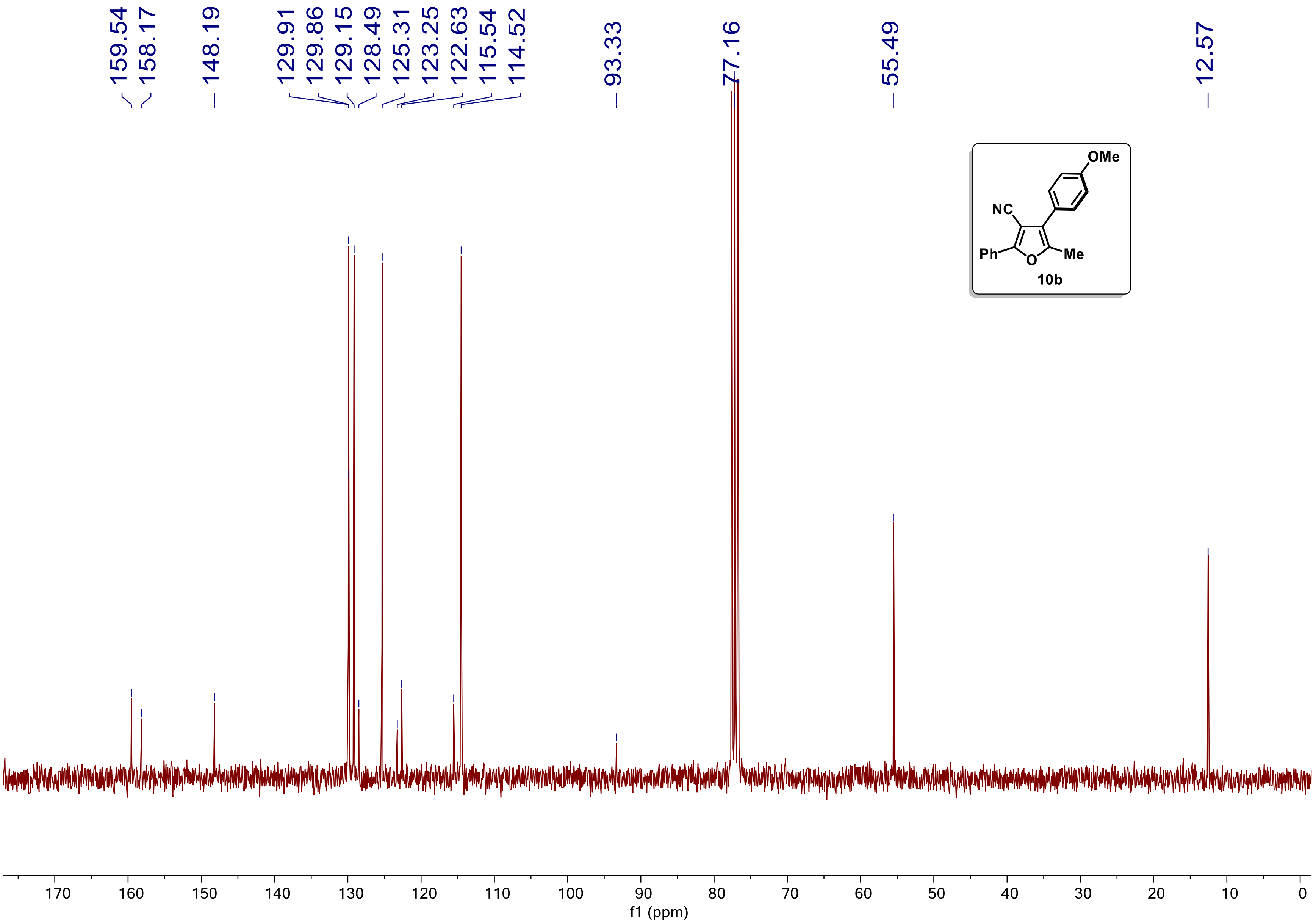
159.54
158.17
148.19
129.91
129.86
129.15
128.49
125.31
123.25
122.63
115.54
114.52
93.33
77.16
55.49
12.57
OMe
NC
Ph
O
Me
10b
170 160 150 140 130 120 110 100 90 80 70 60 50 40 30 20 10 0
f1 (ppm)

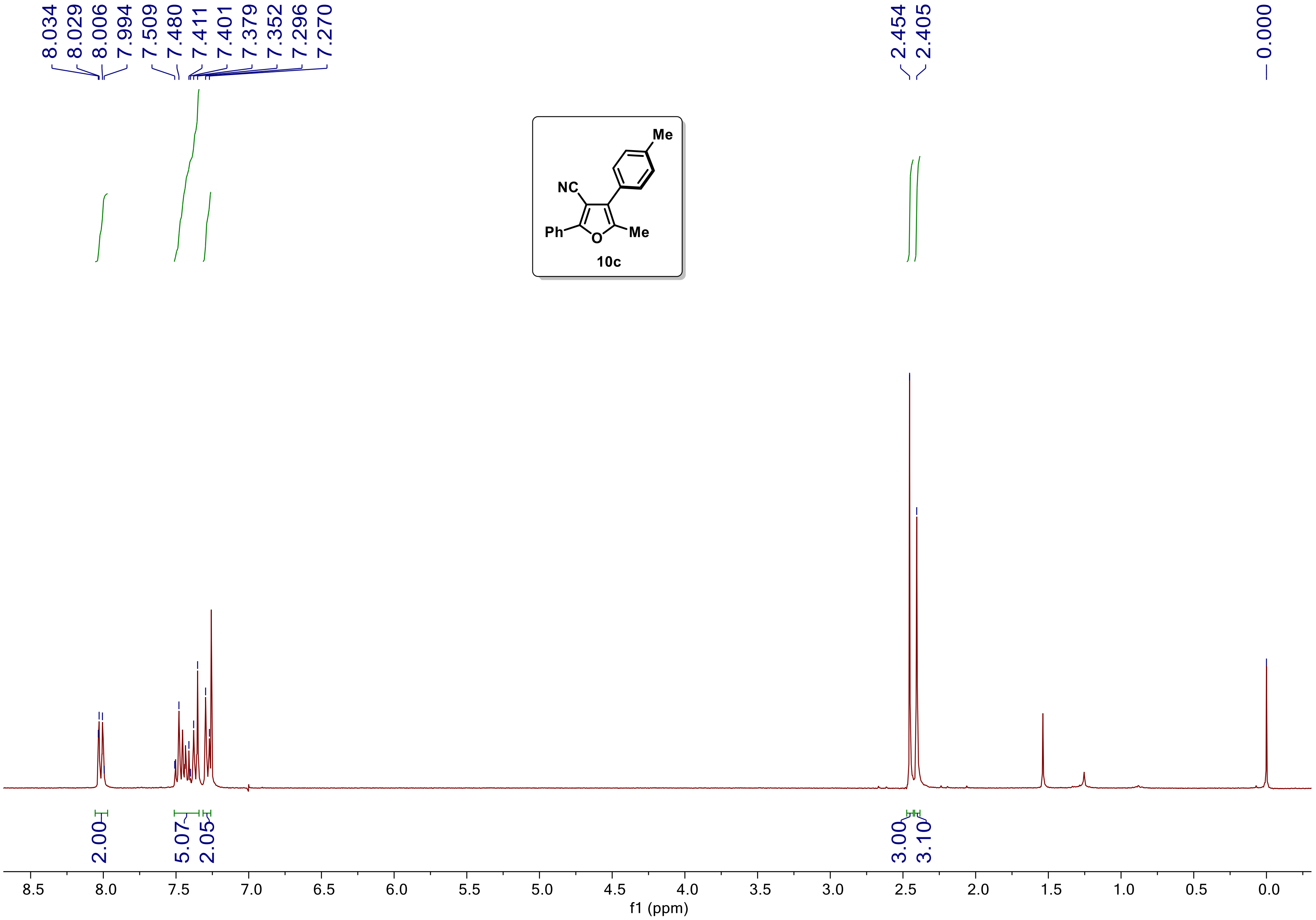
8.034
8.029
8.006
7.994
7.509
7.480
7.411
7.401
7.379
7.352
7.296
7.270
2.454
2.405
0.000
NC
Me
Ph
O
Me
10c
2.00
5.07
2.05
3.00
3.10
8.5
8.0
7.5
7.0
6.5
6.0
5.5
5.0
4.5
4.0
3.5
3.0
2.5
2.0
1.5
1.0
0.5
0.0
f1 (ppm)

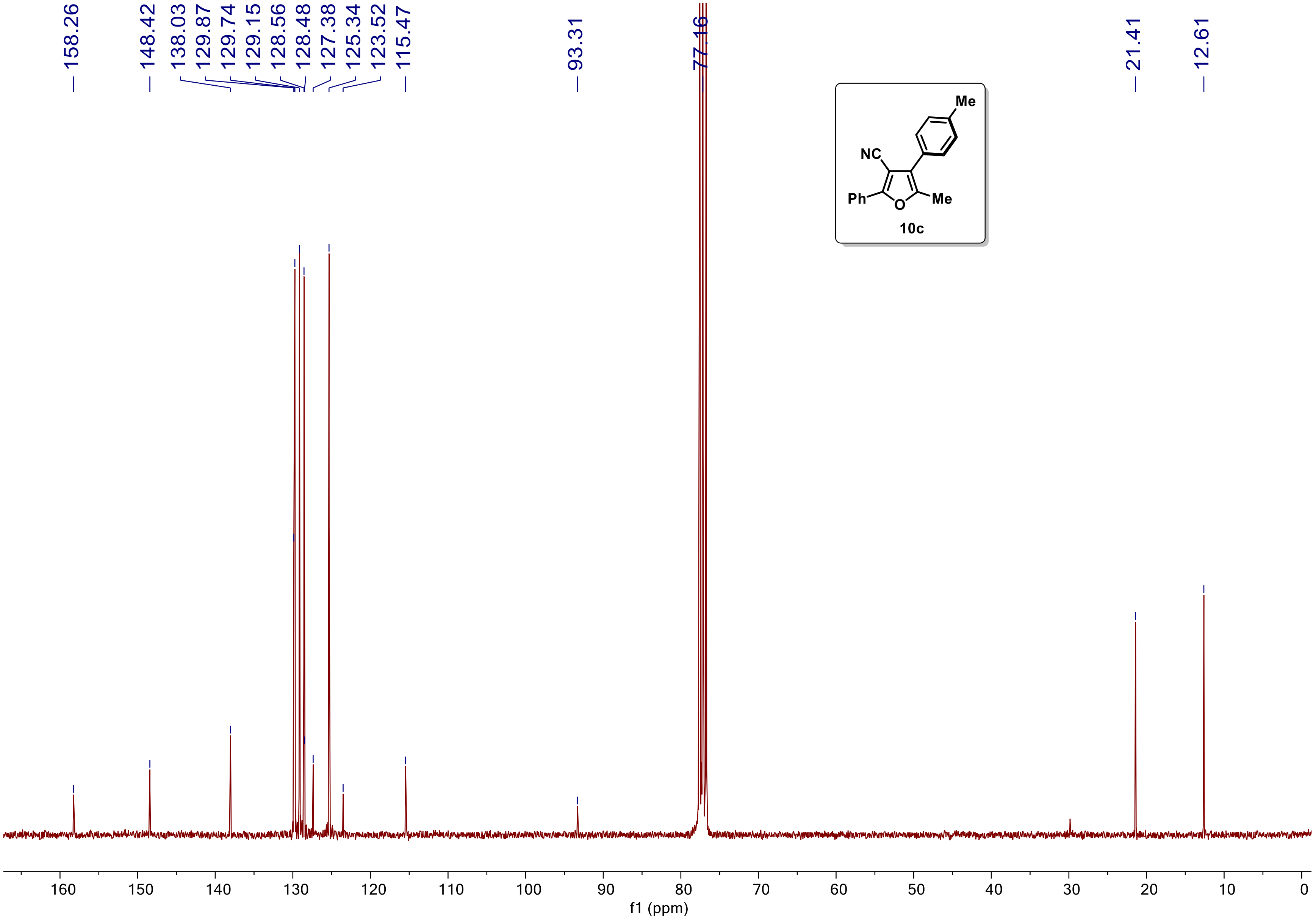
158.26
148.42
138.03
129.87
129.74
129.15
128.56
128.48
127.38
125.34
123.52
115.47
93.31
77.16
21.41
12.61
NC
Me
Ph
O
Me
10c
160
150
140
130
120
110
100
90
80
70
60
50
40
30
20
10
0
f1 (ppm)

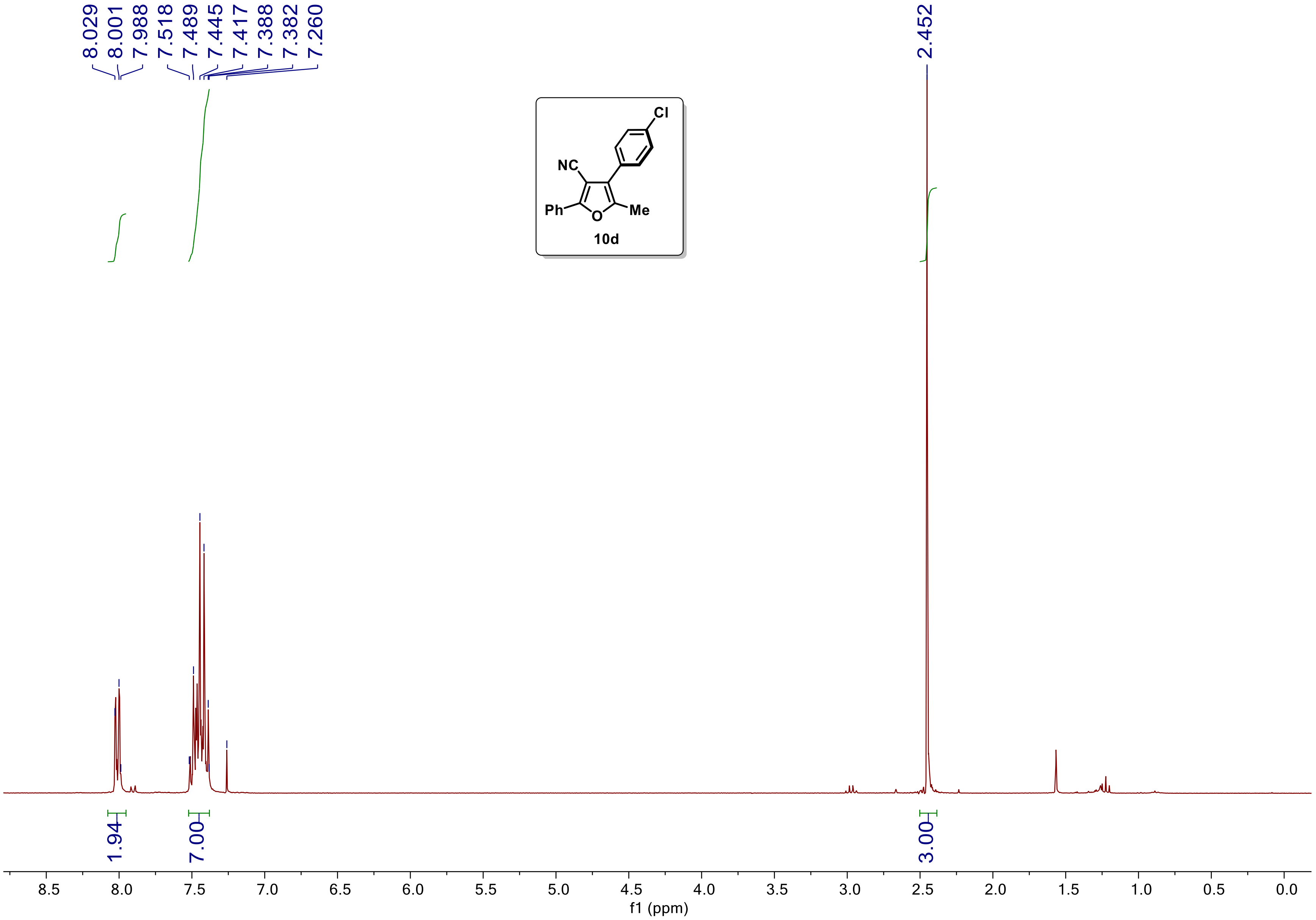
8.029
8.001
7.988
7.518
7.489
7.445
7.417
7.388
7.382
7.260
2.452
NC
Cl
Ph
O
Me
10d
1.94
7.00
3.00
8.5
8.0
7.5
7.0
6.5
6.0
5.5
5.0
4.5
4.0
3.5
3.0
2.5
2.0
1.5
1.0
0.5
0.0
f1 (ppm)

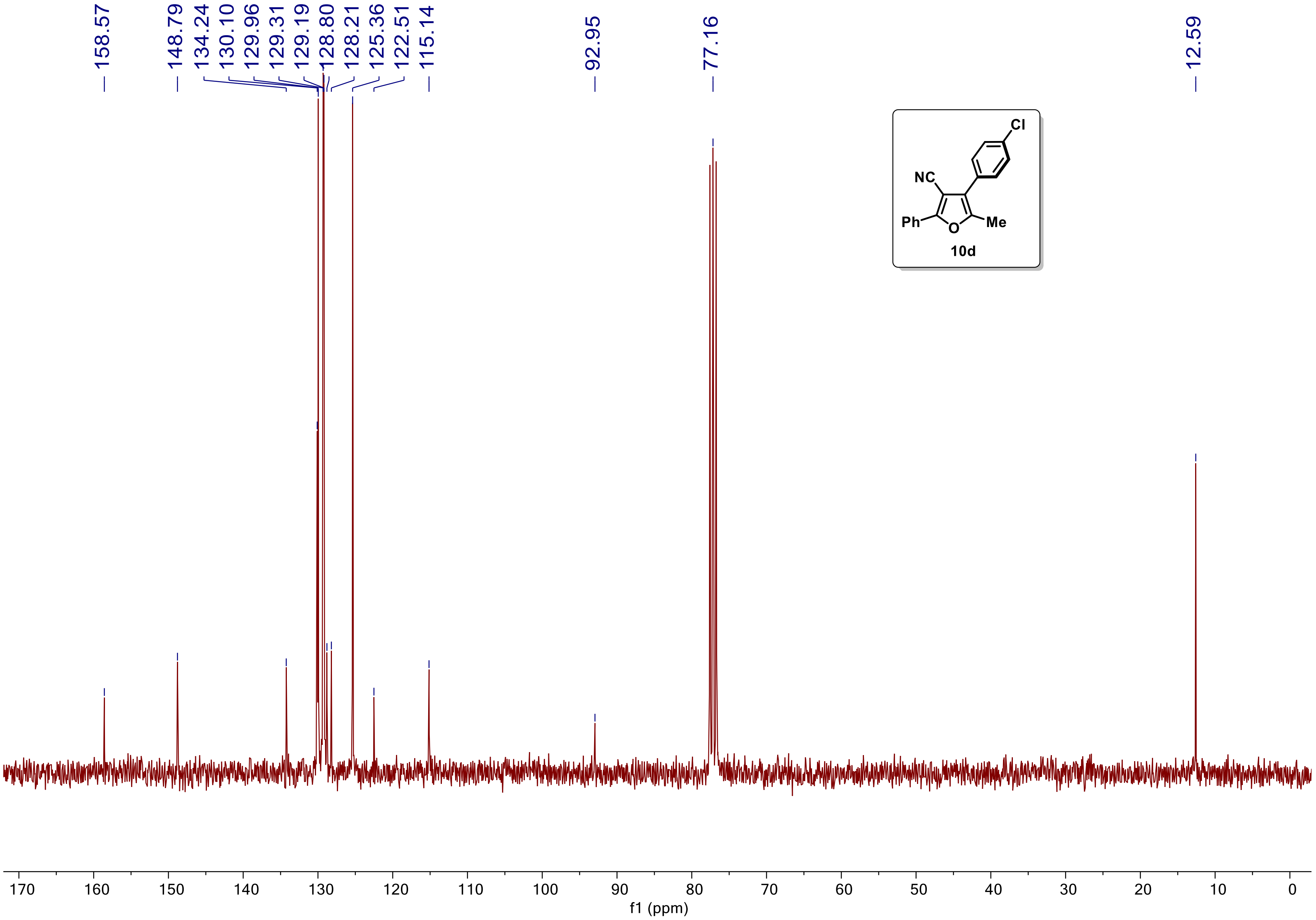
158.57
148.79
134.24
130.10
129.96
129.31
129.19
128.80
128.21
125.36
122.51
115.14
92.95
77.16
12.59
NC
Cl
Ph
O
Me
10d
170
160
150
140
130
120
110
100
90
80
70
60
50
40
30
20
10
0
f1 (ppm)

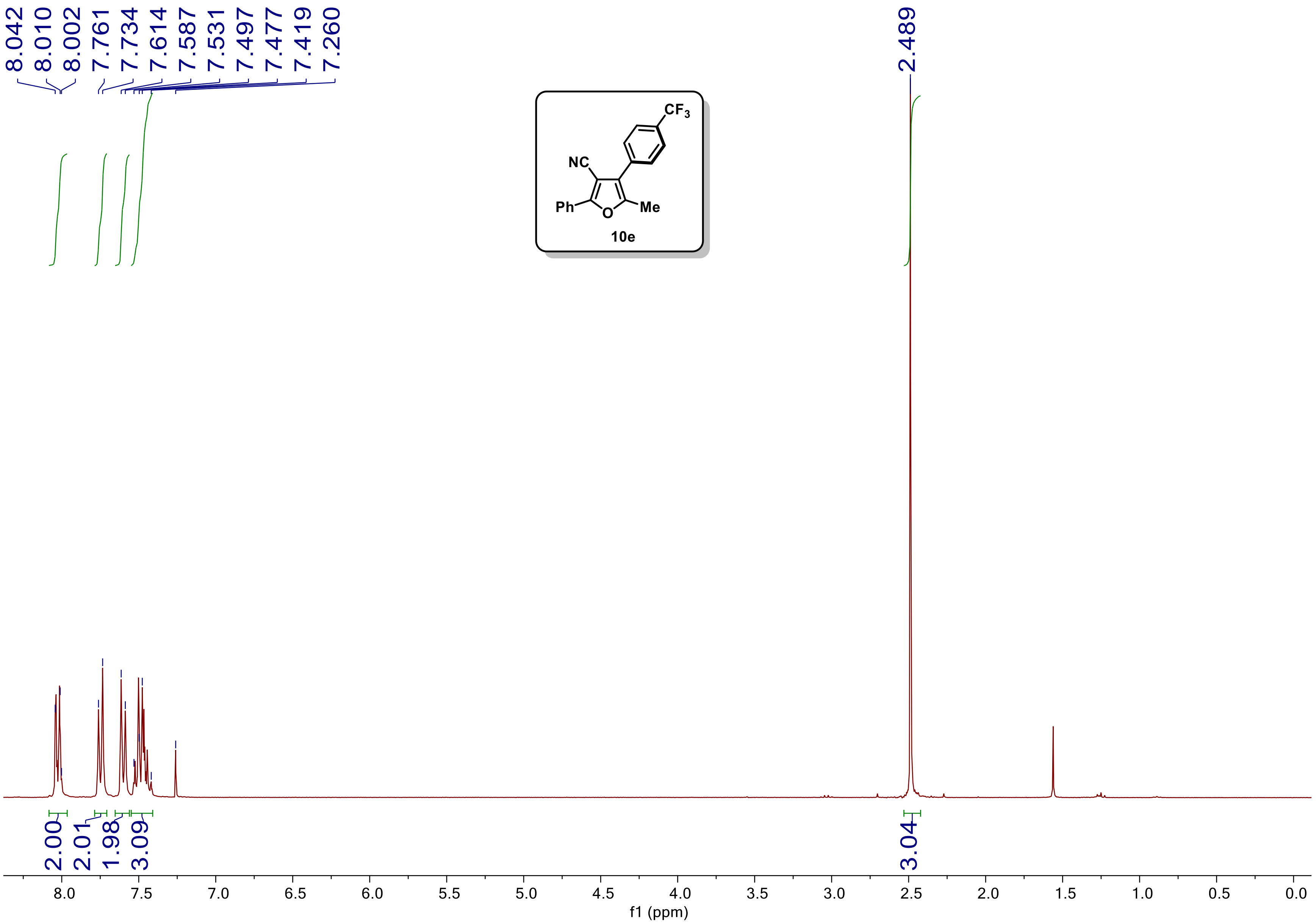
8.042
8.010
8.002
7.761
7.734
7.614
7.587
7.531
7.497
7.477
7.419
7.260
2.489
CF3
NC
Ph
O
Me
10e
2.00
2.01
1.98
3.09
3.04
8.0
7.5
7.0
6.5
6.0
5.5
5.0
4.5
4.0
3.5
3.0
2.5
2.0
1.5
1.0
0.5
0.0
f1 (ppm)

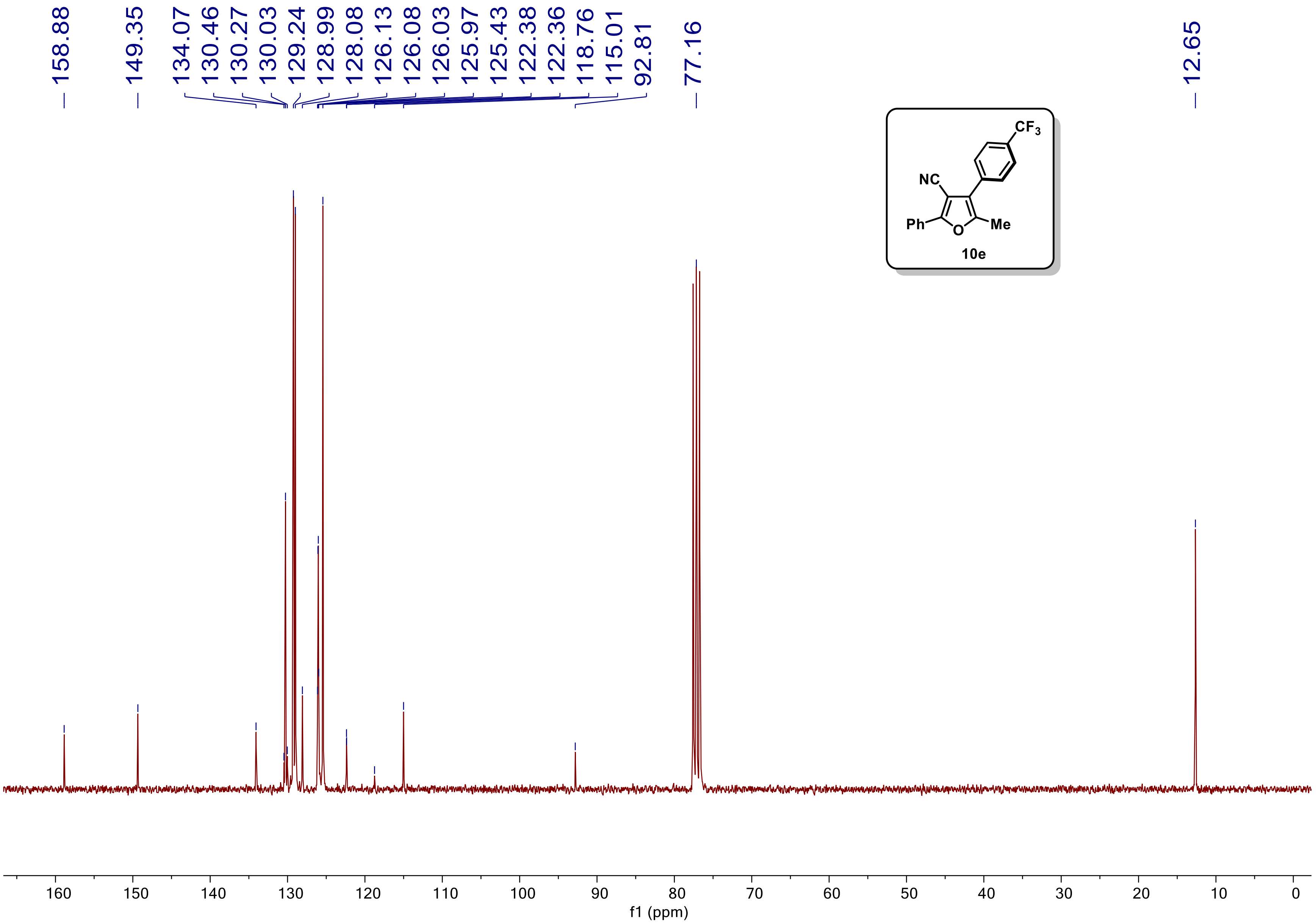
158.88
149.35
134.07
130.46
130.27
130.03
129.24
128.99
128.08
126.13
126.08
126.03
125.97
125.43
122.38
122.36
118.76
115.01
92.81
77.16
12.65
CF3
NC
Ph
O
Me
10e
160 150 140 130 120 110 100 90 80 70 60 50 40 30 20 10 0
f1 (ppm)

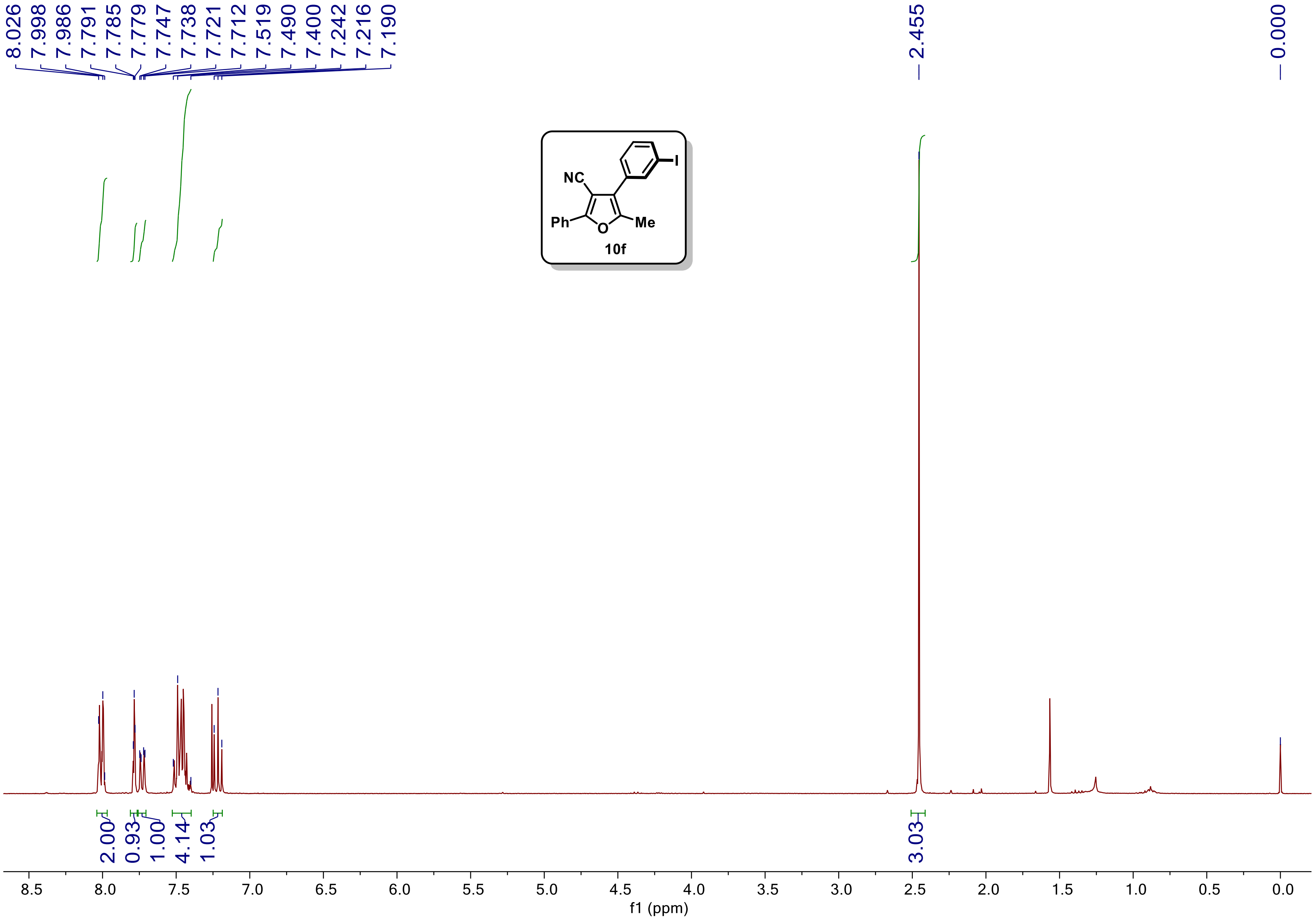
8.026
7.998
7.986
7.791
7.785
7.779
7.747
7.738
7.721
7.712
7.519
7.490
7.400
7.242
7.216
7.190
2.455
0.000
NC
I
Ph
O
Me
10f
2.00
0.93
1.00
4.14
1.03
3.03
8.5
8.0
7.5
7.0
6.5
6.0
5.5
5.0
4.5
4.0
3.5
3.0
2.5
2.0
1.5
1.0
0.5
0.0
f1 (ppm)

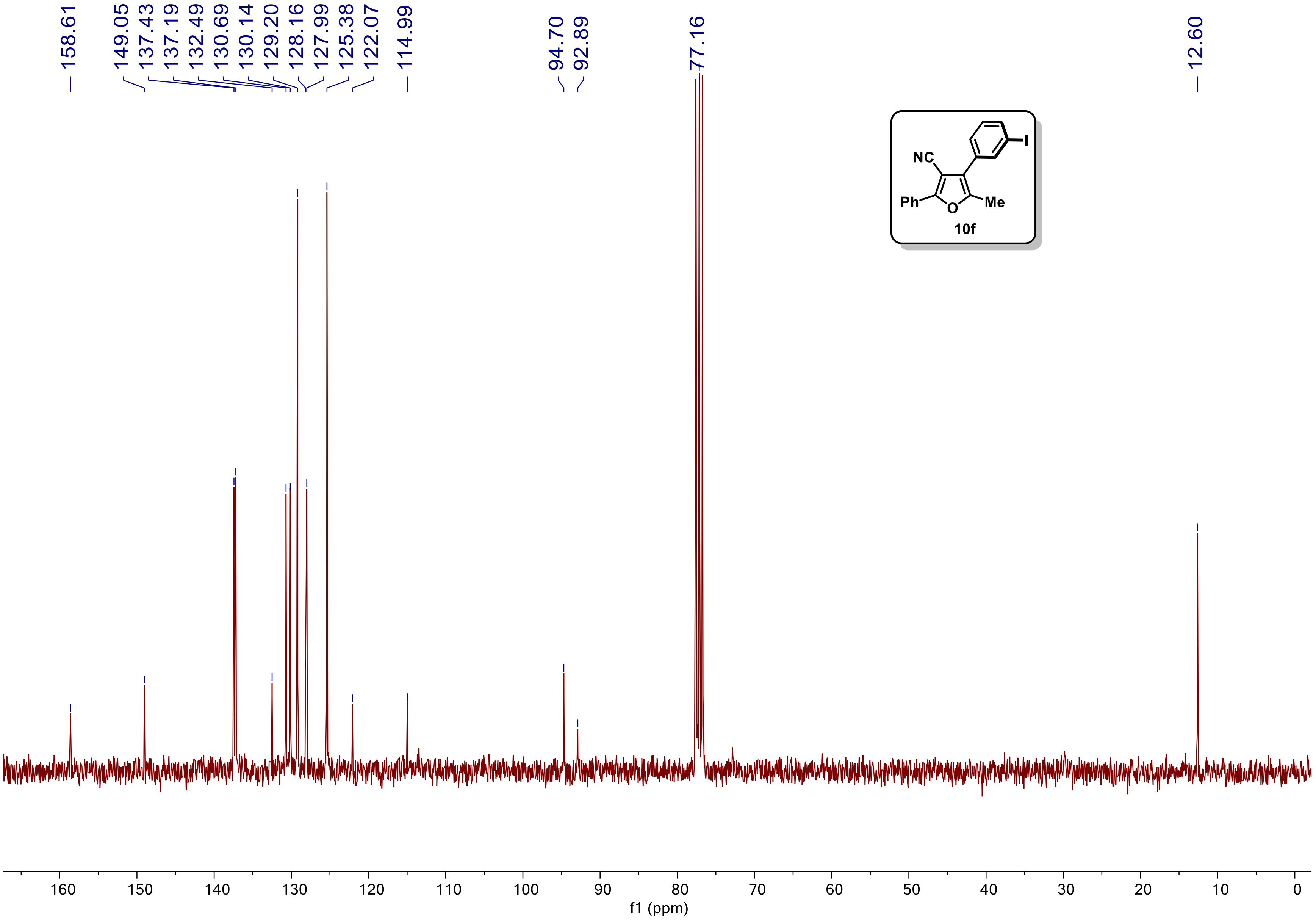
158.61
149.05
137.43
137.19
132.49
130.69
130.14
129.20
128.16
127.99
125.38
122.07
114.99
94.70
92.89
77.16
12.60
NC
Ph
O
Me
I
10f
160
150
140
130
120
110
100
90
80
70
60
50
40
30
20
10
0
f1 (ppm)

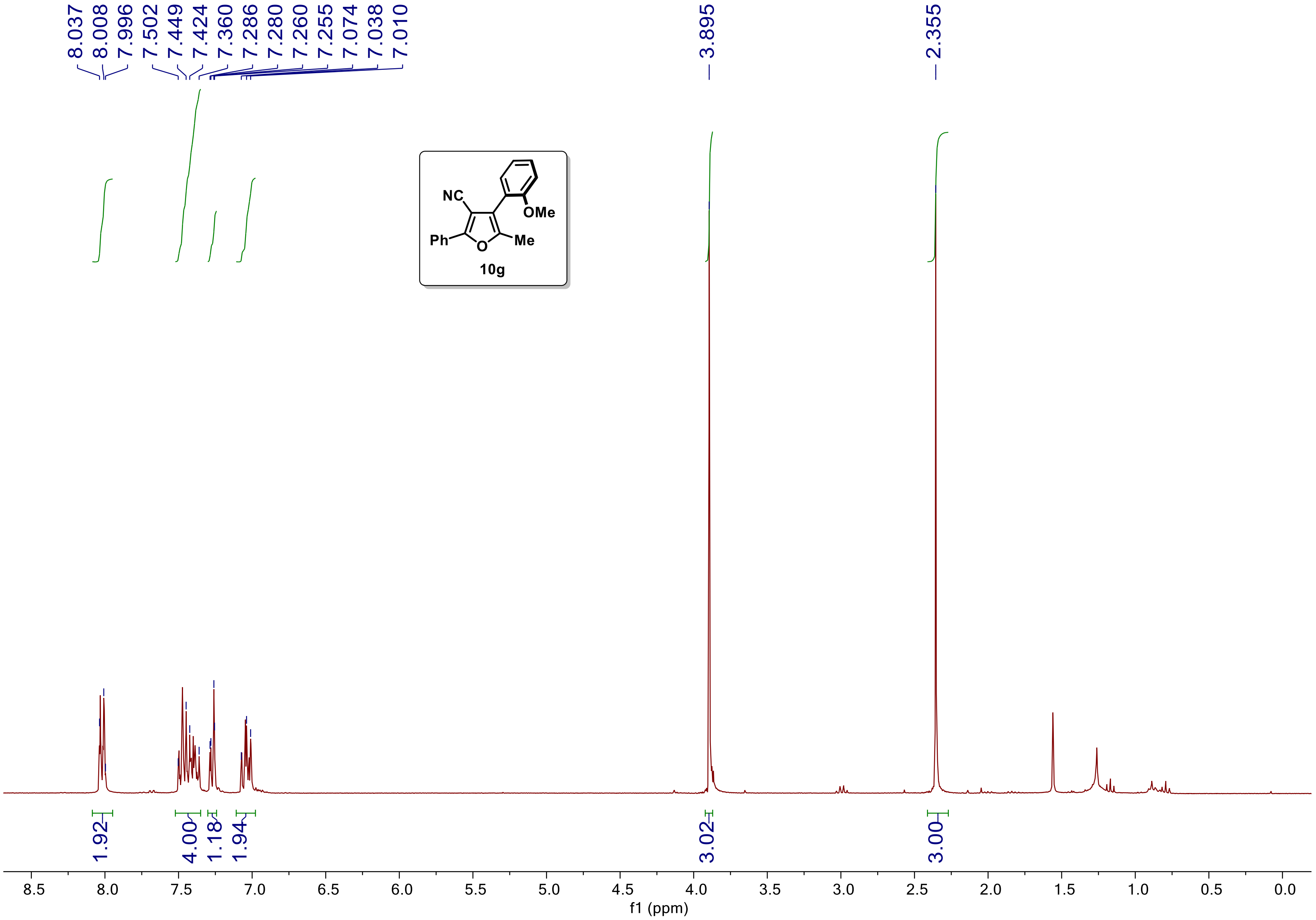

8.037
8.008
7.996
7.502
7.449
7.424
7.360
7.286
7.280
7.260
7.255
7.074
7.038
7.010
3.895
2.355
NC
OMe
Ph
O
Me
10g
1.92
4.00
1.18
1.94
3.02
3.00
8.5
8.0
7.5
7.0
6.5
6.0
5.5
5.0
4.5
4.0
3.5
3.0
2.5
2.0
1.5
1.0
0.5
0.0
f1 (ppm)

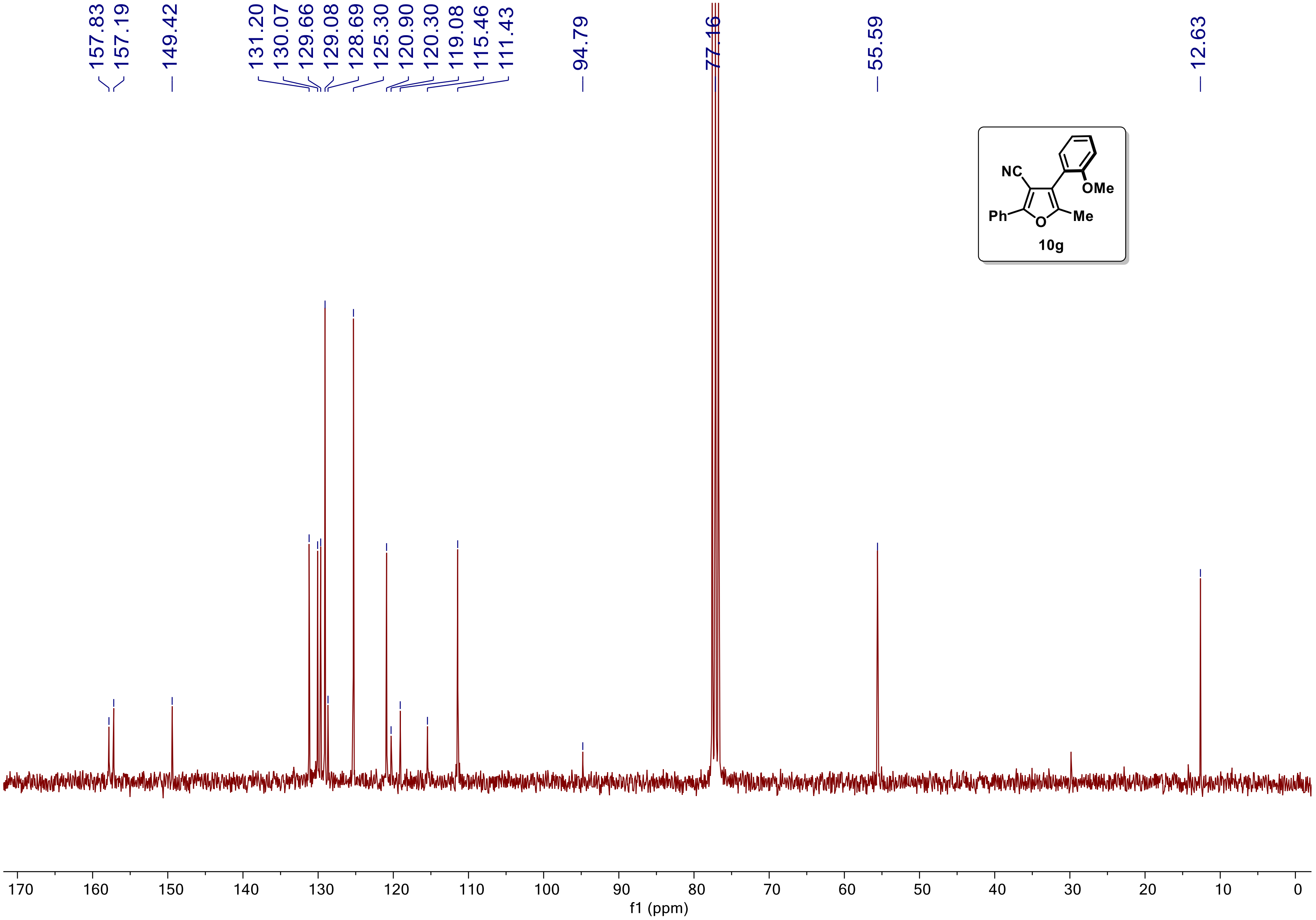
157.83
157.19
149.42
131.20
130.07
129.66
129.08
128.69
125.30
120.90
120.30
119.08
115.46
111.43
94.79
77.16
55.59
12.63
NC
OMe
Ph
O
Me
10g
170
160
150
140
130
120
110
100
90
80
70
60
50
40
30
20
10
0
f1 (ppm)

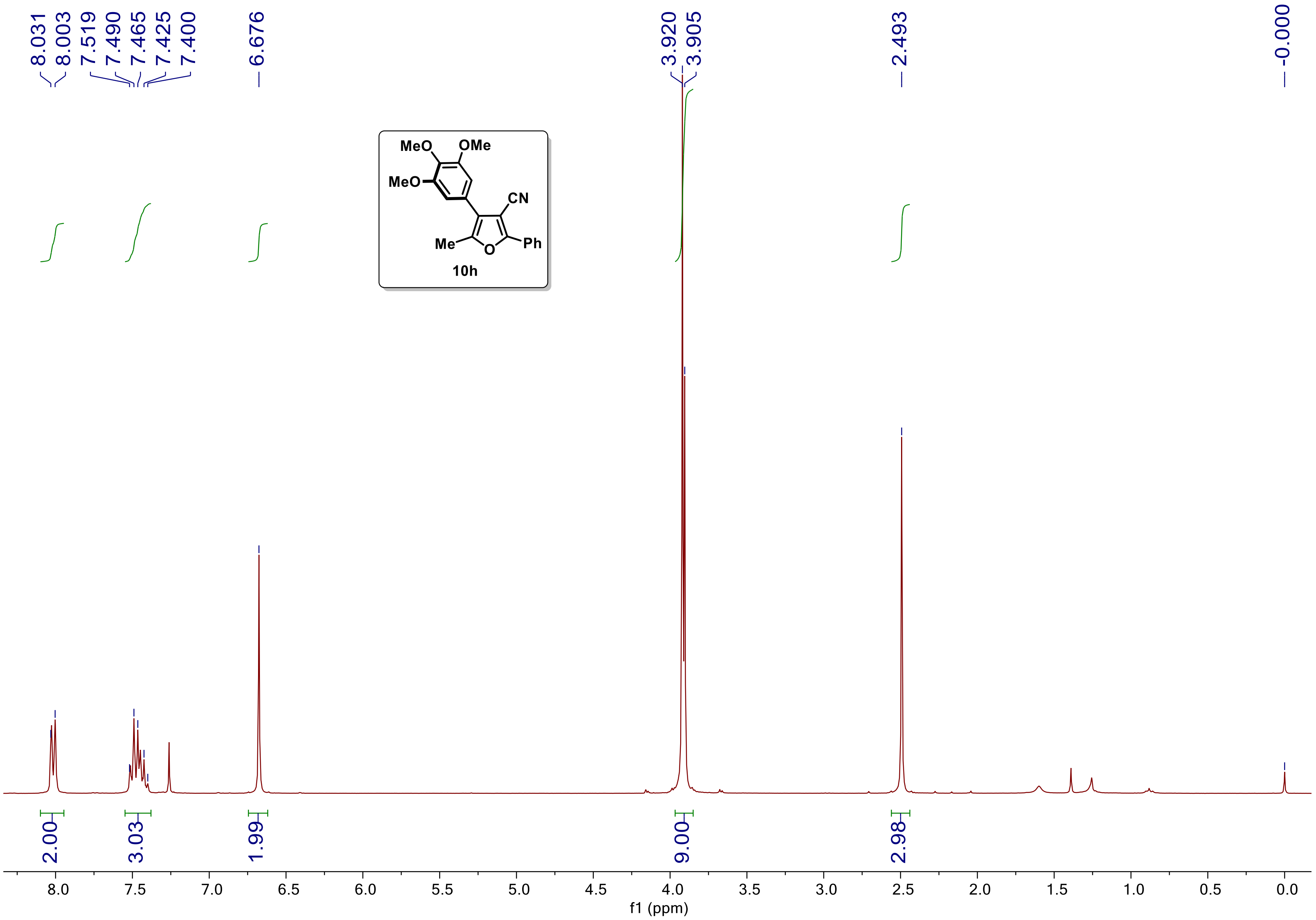
8.031
8.003
7.519
7.490
7.465
7.425
7.400
6.676
3.920
3.905
2.493
-0.000
MeO
OMe
MeO
CN
Me
O
Ph
10h
2.00
3.03
1.99
9.00
2.98
8.0
7.5
7.0
6.5
6.0
5.5
5.0
4.5
4.0
3.5
3.0
2.5
2.0
1.5
1.0
0.5
0.0
f1 (ppm)

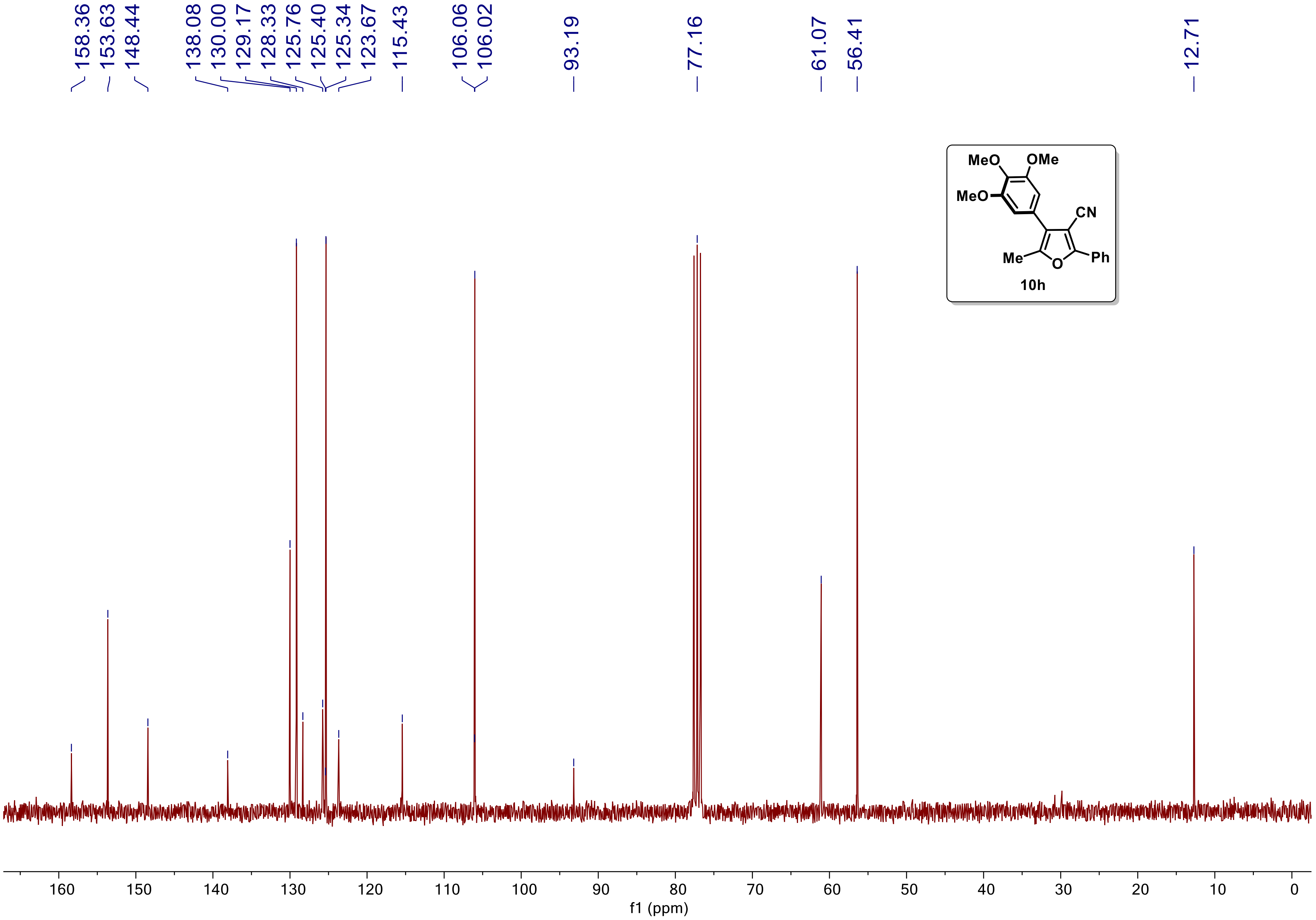
158.36
153.63
148.44
138.08
130.00
129.17
128.33
125.76
125.40
125.34
123.67
115.43
106.06
106.02
93.19
77.16
61.07
56.41
12.71
MeO
OMe
MeO
CN
Me
O
Ph
10h
160
150
140
130
120
110
100
90
80
70
60
50
40
30
20
10
0
f1 (ppm)

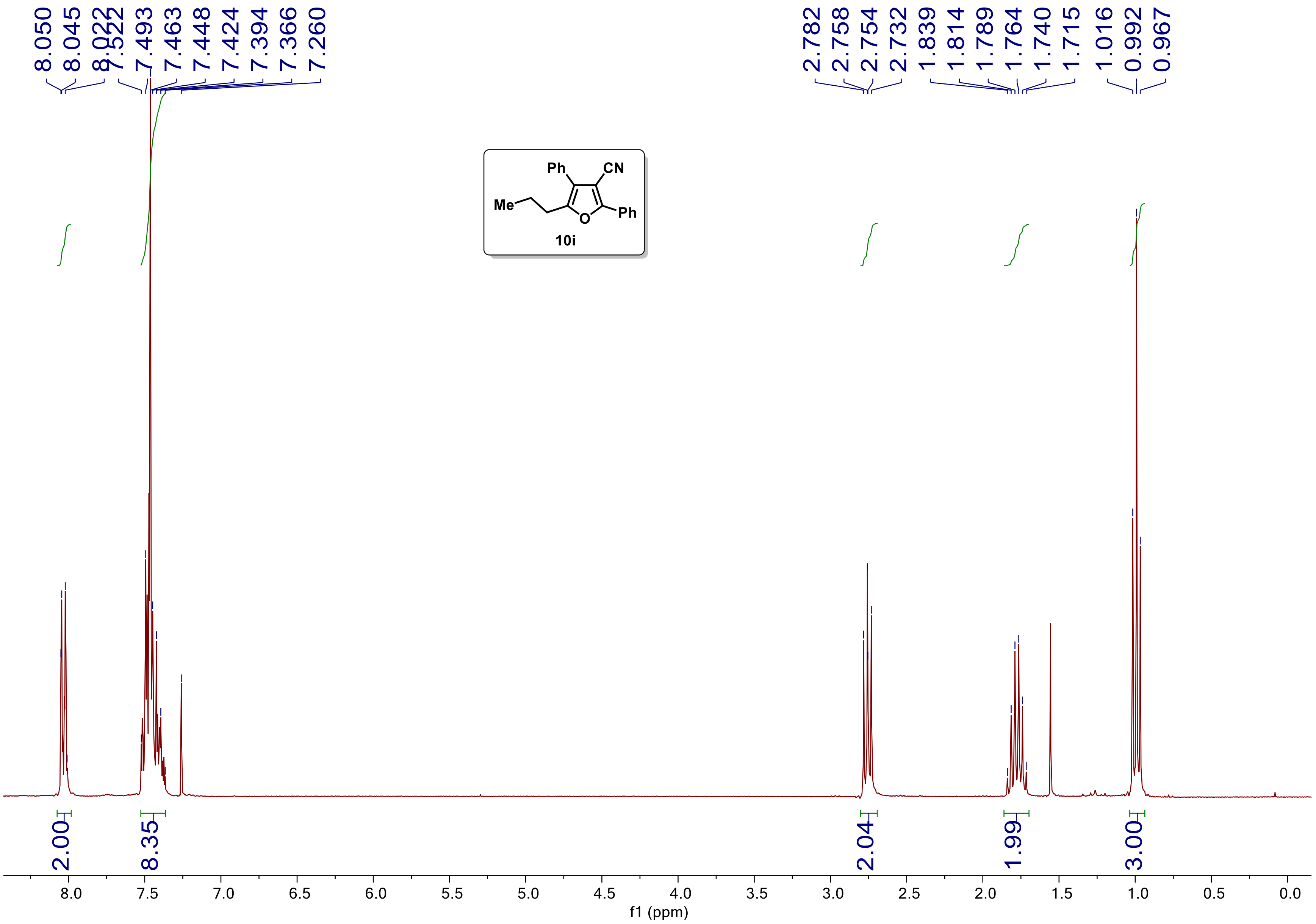
8.050
8.045
8.022
7.522
7.493
7.463
7.448
7.424
7.394
7.366
7.260
2.782
2.758
2.754
2.732
1.839
1.814
1.789
1.764
1.740
1.715
1.016
0.992
0.967
Ph
CN
Me
O
Ph
10i
2.00
8.35
2.04
1.99
3.00
f1 (ppm)

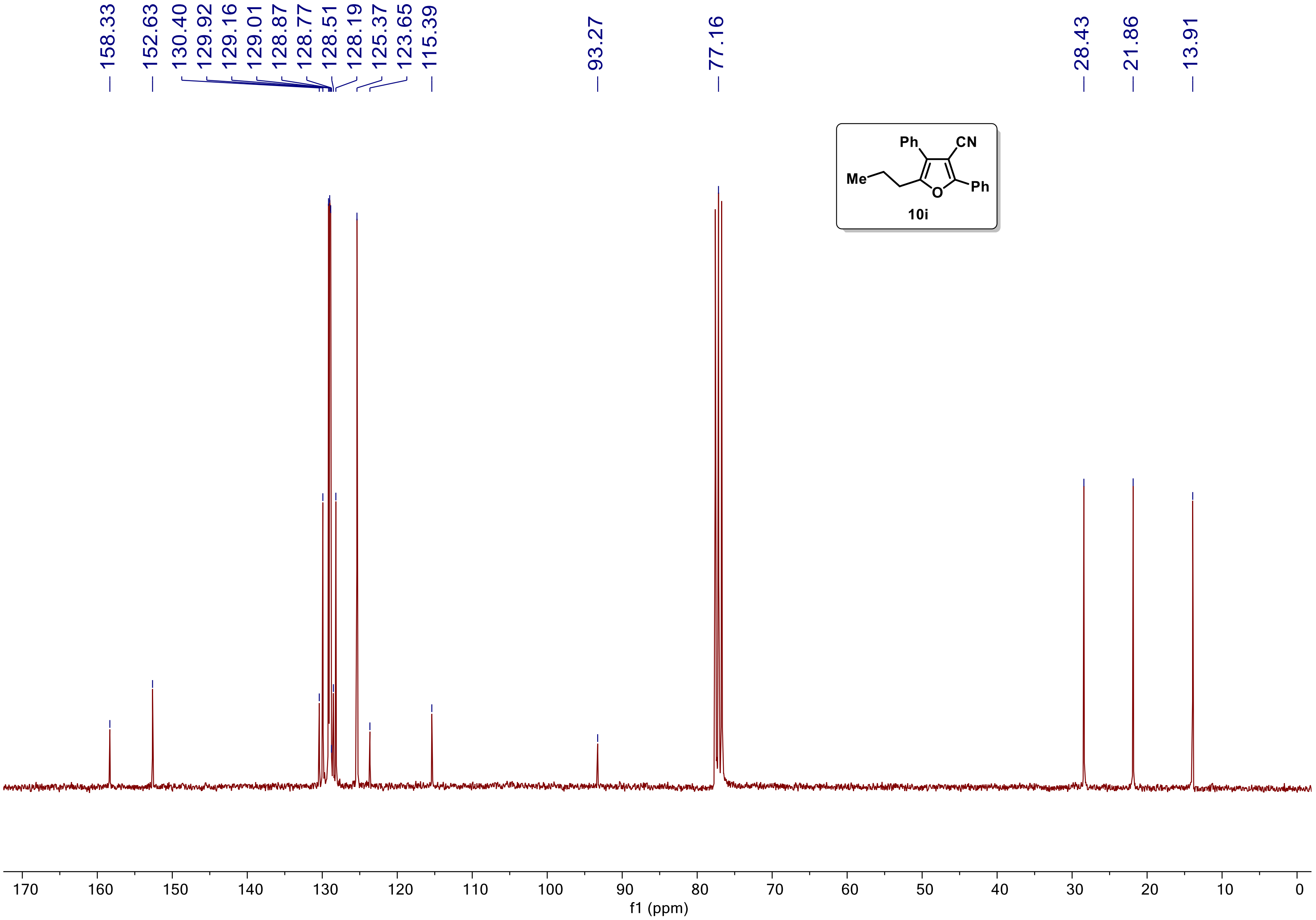
158.33
152.63
130.40
129.92
129.16
129.01
128.87
128.77
128.51
128.19
125.37
123.65
115.39
93.27
77.16
28.43
21.86
13.91
Ph
CN
Me
O
Ph
10i
170 160 150 140 130 120 110 100 90 80 70 60 50 40 30 20 10 0
f1 (ppm)

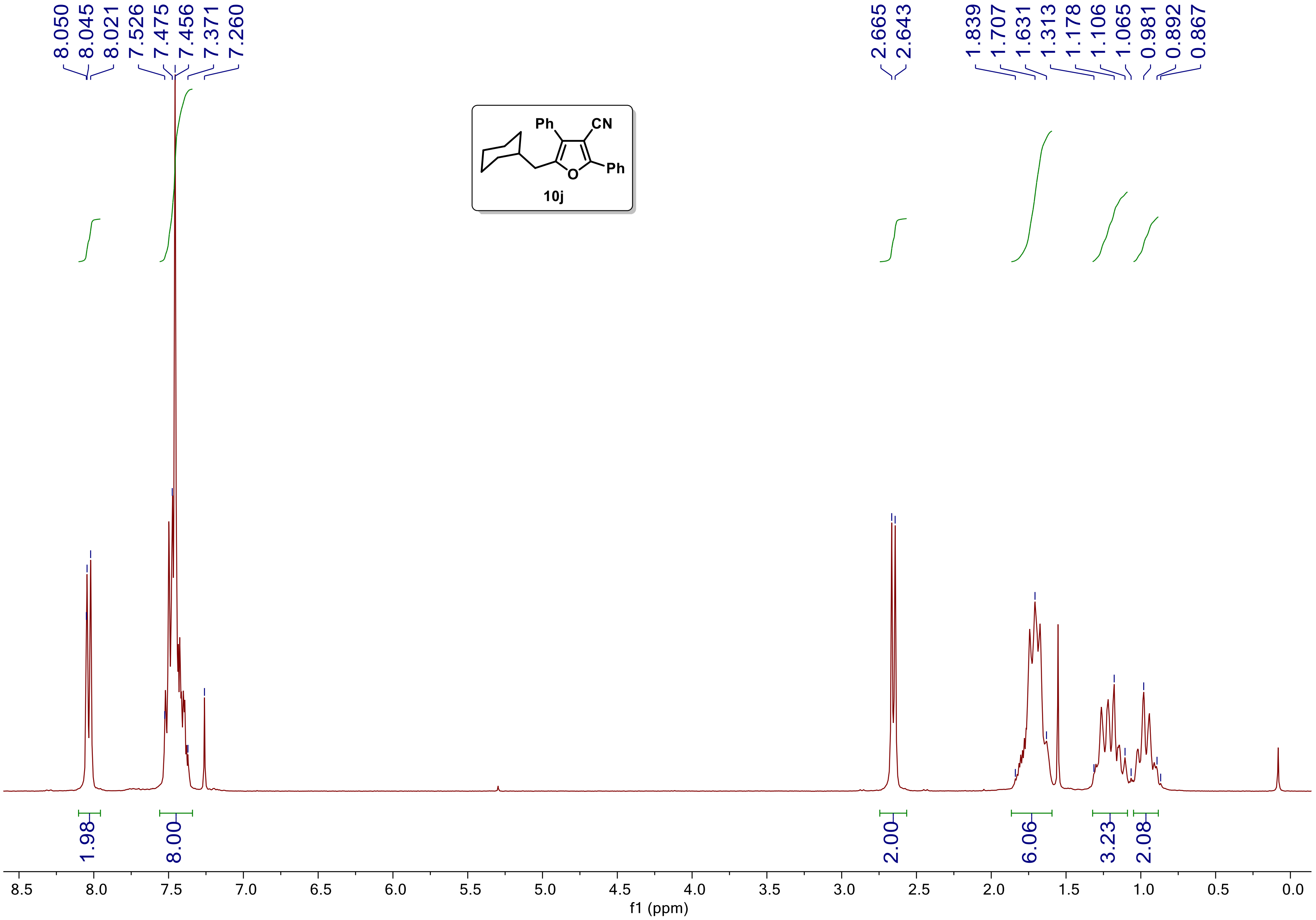
8.050
8.045
8.021
7.526
7.475
7.456
7.371
7.260
2.665
2.643
1.839
1.707
1.631
1.313
1.178
1.106
1.065
0.981
0.892
0.867
Ph
CN
Ph
O
10j
1.98
8.00
2.00
6.06
3.23
2.08
8.5
8.0
7.5
7.0
6.5
6.0
5.5
5.0
4.5
4.0
3.5
3.0
2.5
2.0
1.5
1.0
0.5
0.0
f1 (ppm)

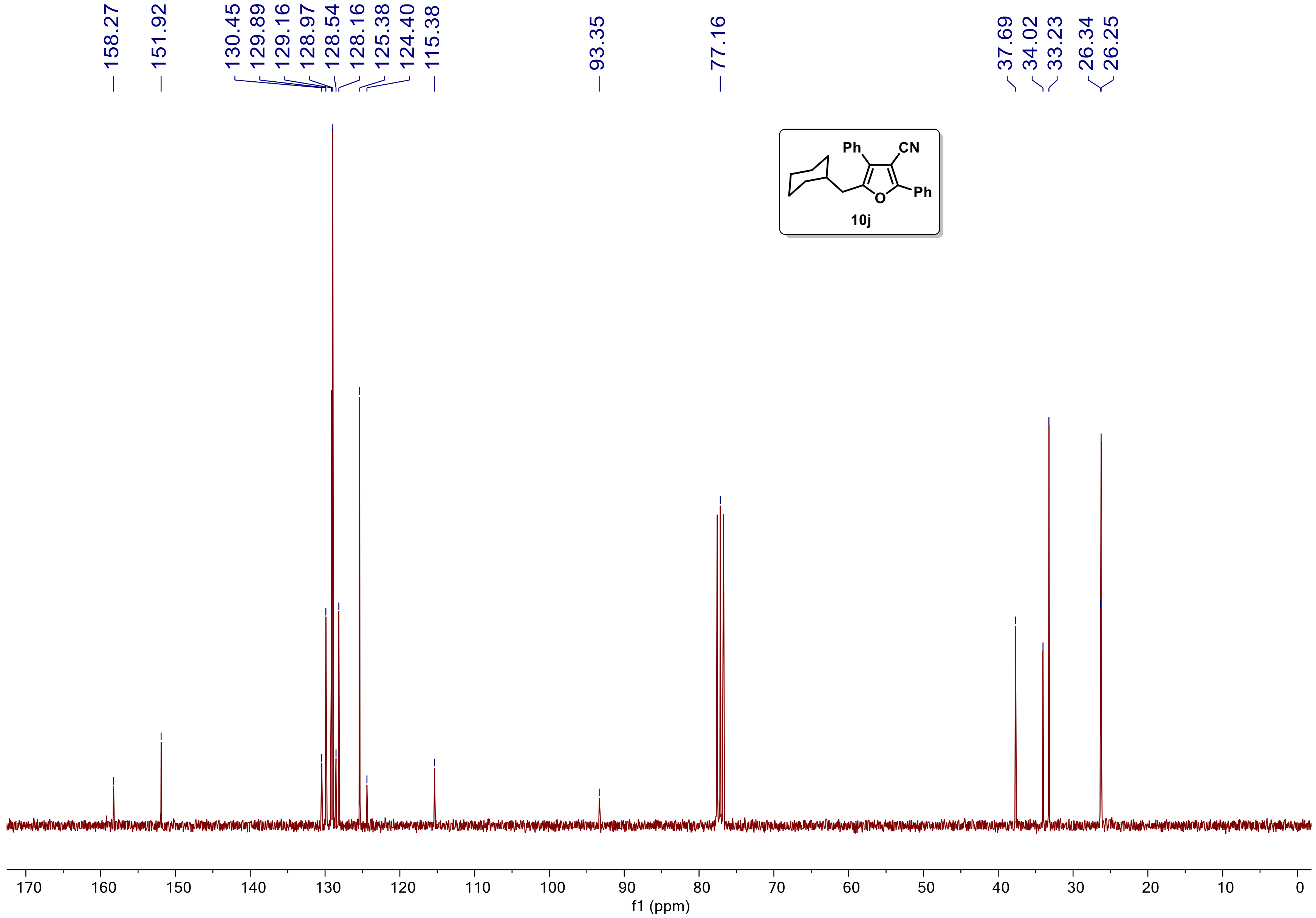
158.27
151.92
130.45
129.89
129.16
128.97
128.54
128.16
125.38
124.40
115.38
93.35
77.16
37.69
34.02
33.23
26.34
26.25
Ph
CN
O
Ph
10j
170 160 150 140 130 120 110 100 90 80 70 60 50 40 30 20 10 0
f1 (ppm)

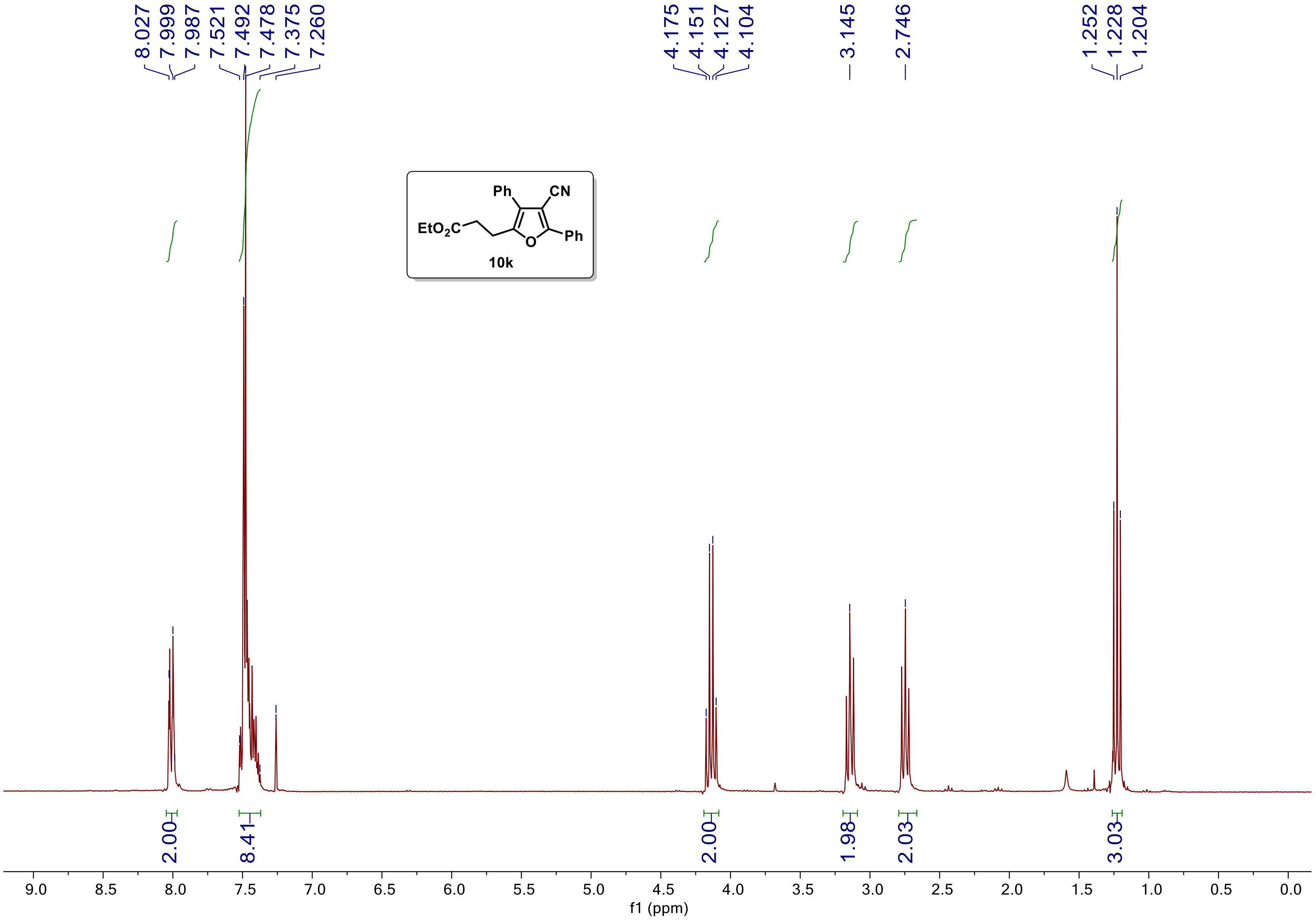
8.027
7.999
7.987
7.521
7.492
7.478
7.375
7.260
4.175
4.151
4.127
4.104
3.145
2.746
1.252
1.228
1.204
Ph
CN
EtO2C
O
Ph
10k
2.00
8.41
2.00
1.98
2.03
3.03
9.0
8.5
8.0
7.5
7.0
6.5
6.0
5.5
5.0
4.5
4.0
3.5
3.0
2.5
2.0
1.5
1.0
0.5
0.0
f1 (ppm)

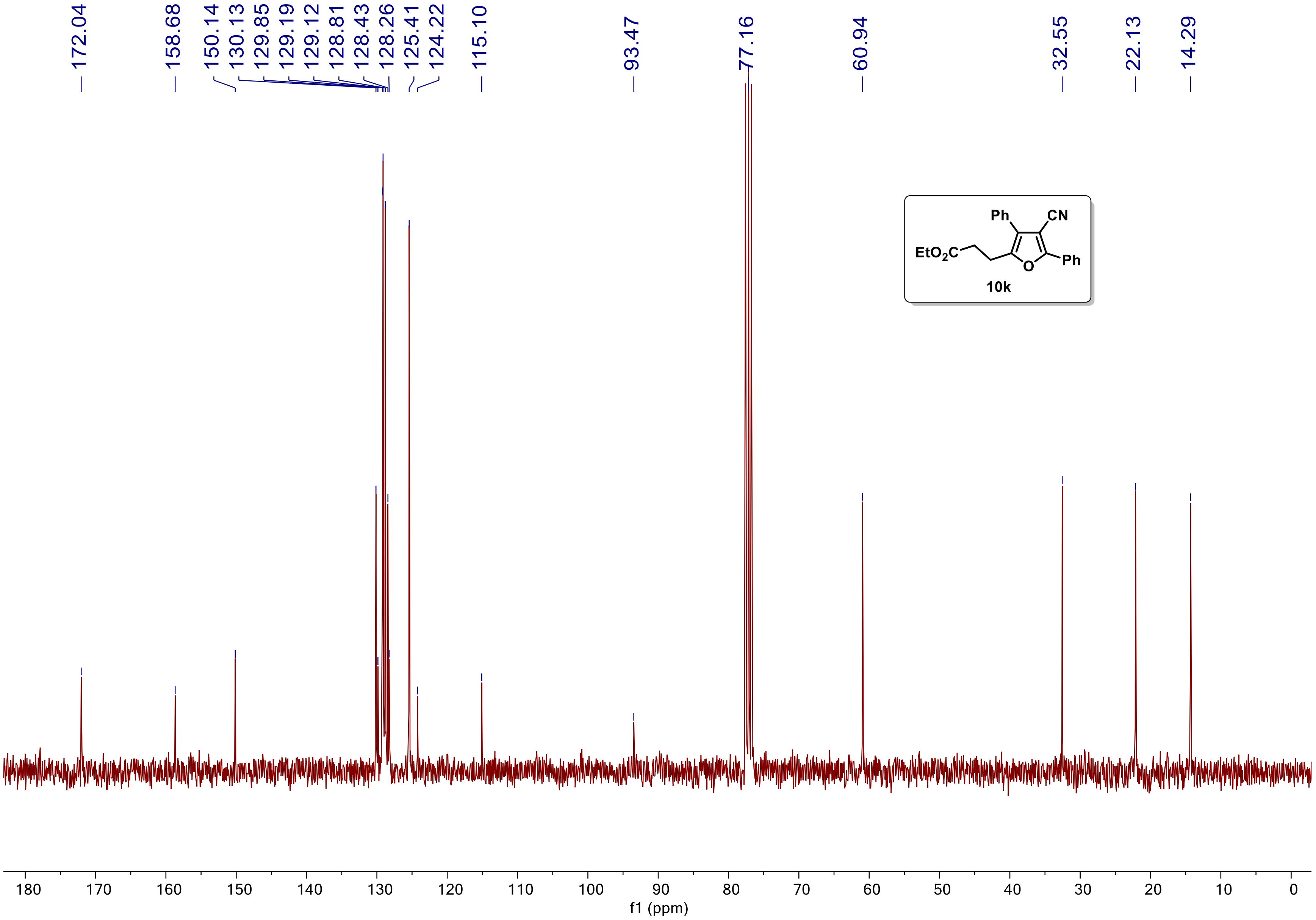
172.04
158.68
150.14
130.13
129.85
129.19
129.12
128.81
128.43
128.26
125.41
124.22
115.10
93.47
77.16
60.94
32.55
22.13
14.29
Ph
CN
EtO2C
O
Ph
10k
180 170 160 150 140 130 120 110 100 90 80 70 60 50 40 30 20 10 0
f1 (ppm)

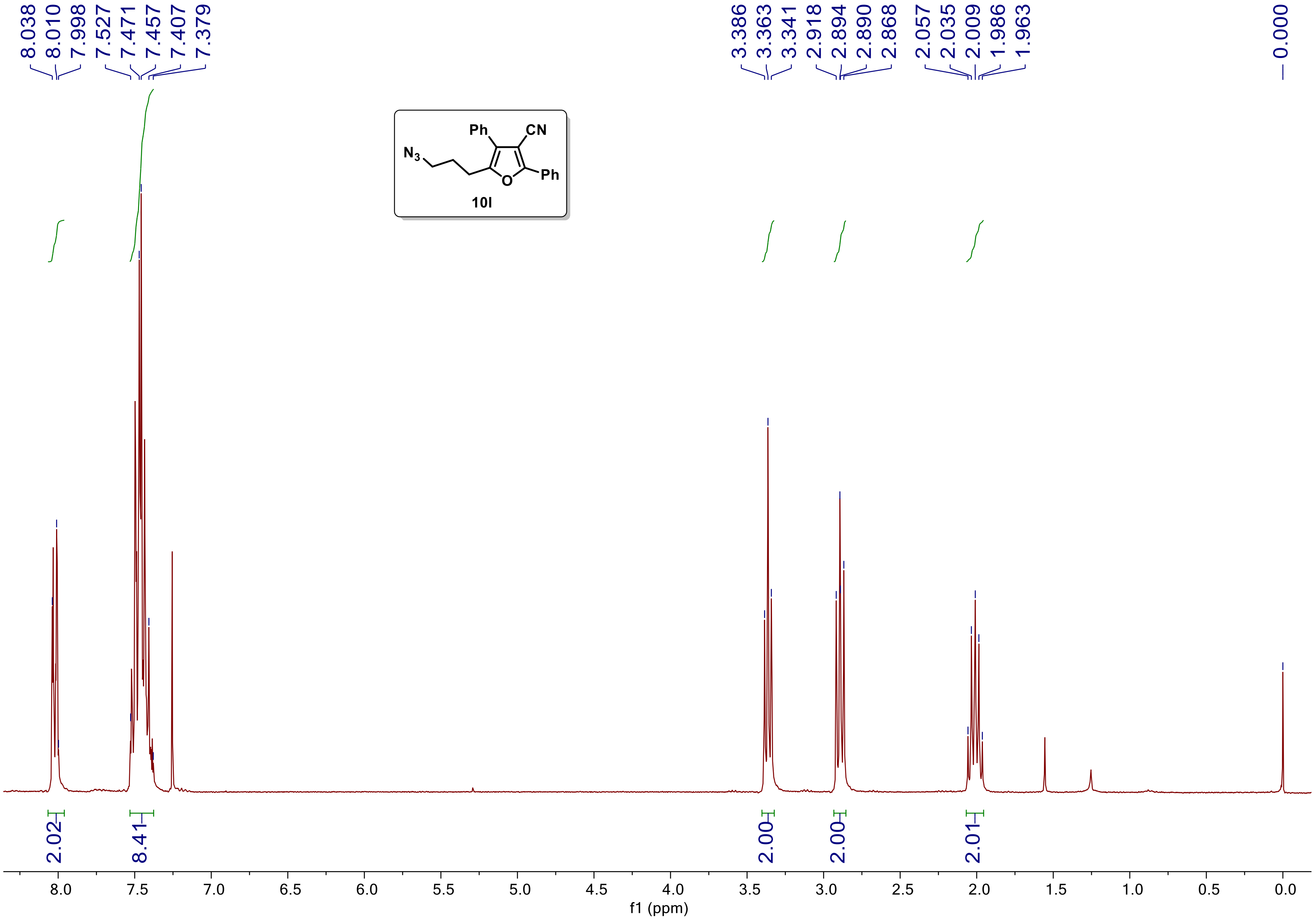
8.038
8.010
7.998
7.527
7.471
7.457
7.407
7.379
3.386
3.363
3.341
2.918
2.894
2.890
2.868
2.057
2.035
2.009
1.986
1.963
0.000
Ph
CN
N3
O
Ph
10l
2.02
8.41
2.00
2.00
2.01
8.0
7.5
7.0
6.5
6.0
5.5
5.0
4.5
4.0
3.5
3.0
2.5
2.0
1.5
1.0
0.5
0.0
f1 (ppm)

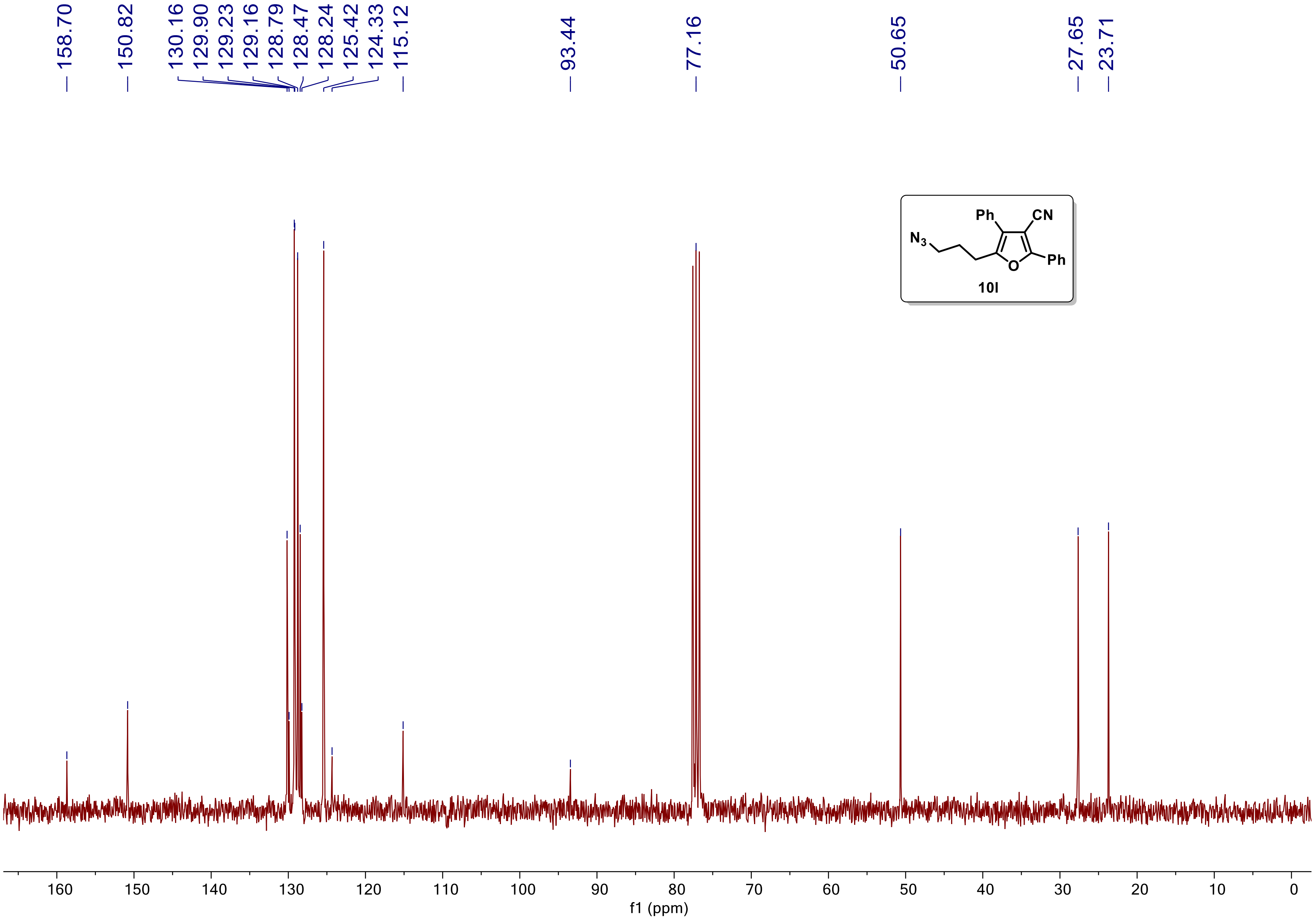
158.70
150.82
130.16
129.90
129.23
129.16
128.79
128.47
128.24
125.42
124.33
115.12
93.44
77.16
50.65
27.65
23.71
Ph
CN
N3
O
Ph
10l
160 150 140 130 120 110 100 90 80 70 60 50 40 30 20 10 0
f1 (ppm)

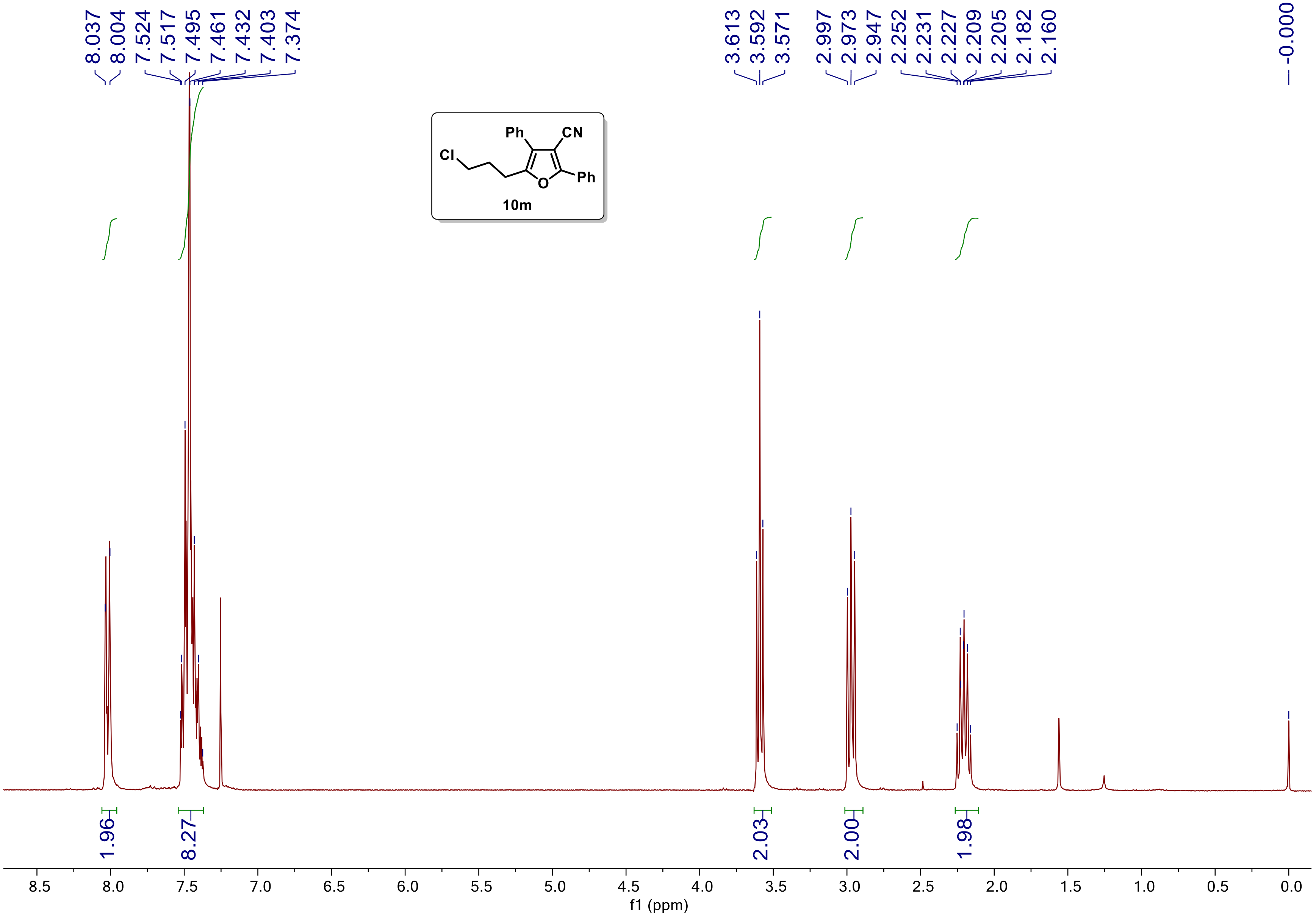
8.037
8.004
7.524
7.517
7.495
7.461
7.432
7.403
7.374
3.613
3.592
3.571
2.997
2.973
2.947
2.252
2.231
2.227
2.209
2.205
2.182
2.160
-0.000
Ph
CN
Cl
Ph
O
10m
1.96
8.27
2.03
2.00
1.98
8.5
8.0
7.5
7.0
6.5
6.0
5.5
5.0
4.5
4.0
3.5
3.0
2.5
2.0
1.5
1.0
0.5
0.0
f1 (ppm)

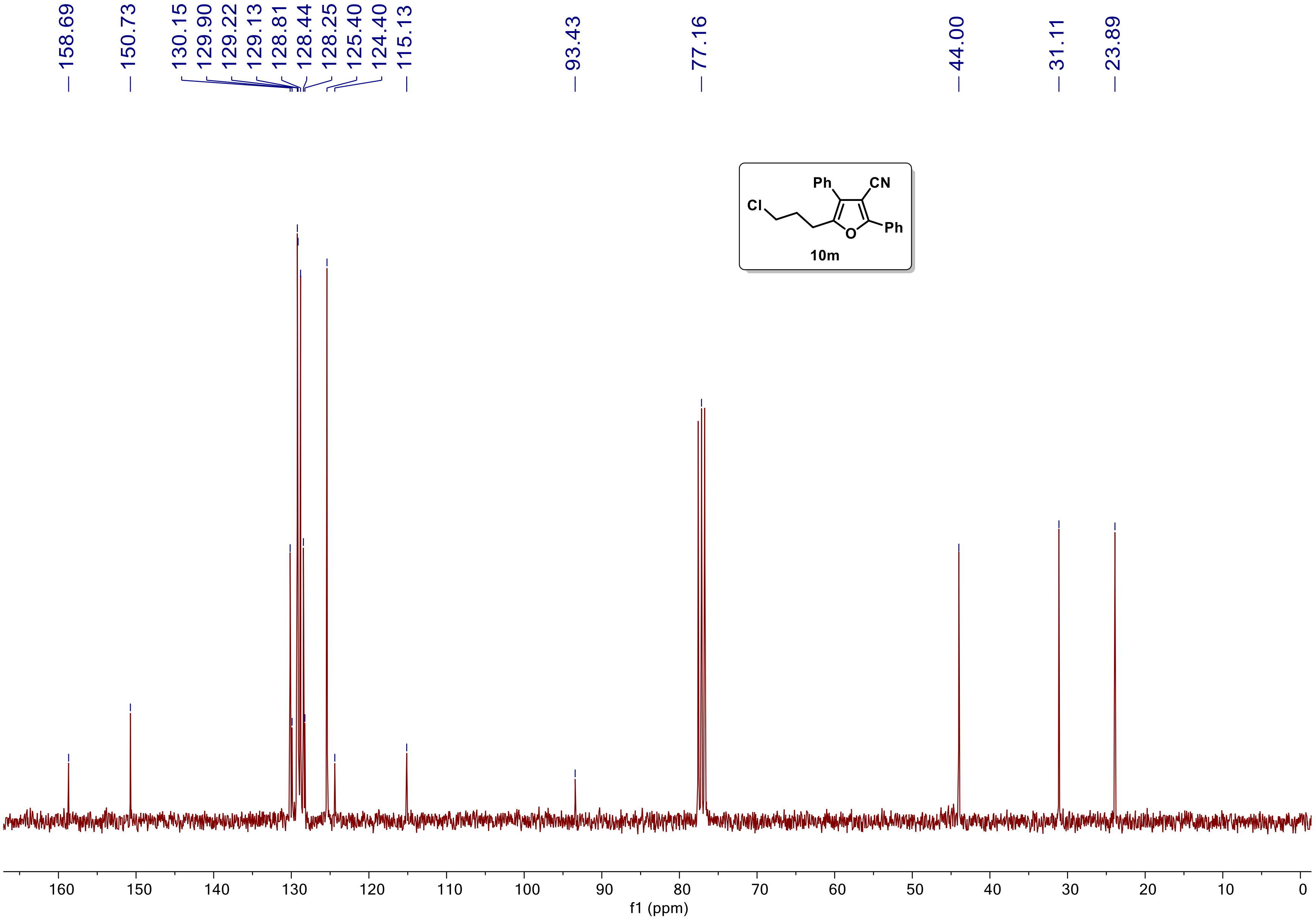

158.69
150.73
130.15
129.90
129.22
129.13
128.81
128.44
128.25
125.40
124.40
115.13
93.43
77.16
44.00
31.11
23.89
Ph
CN
Cl
O
Ph
10m
160
150
140
130
120
110
100
90
80
70
60
50
40
30
20
10
0
f1 (ppm)

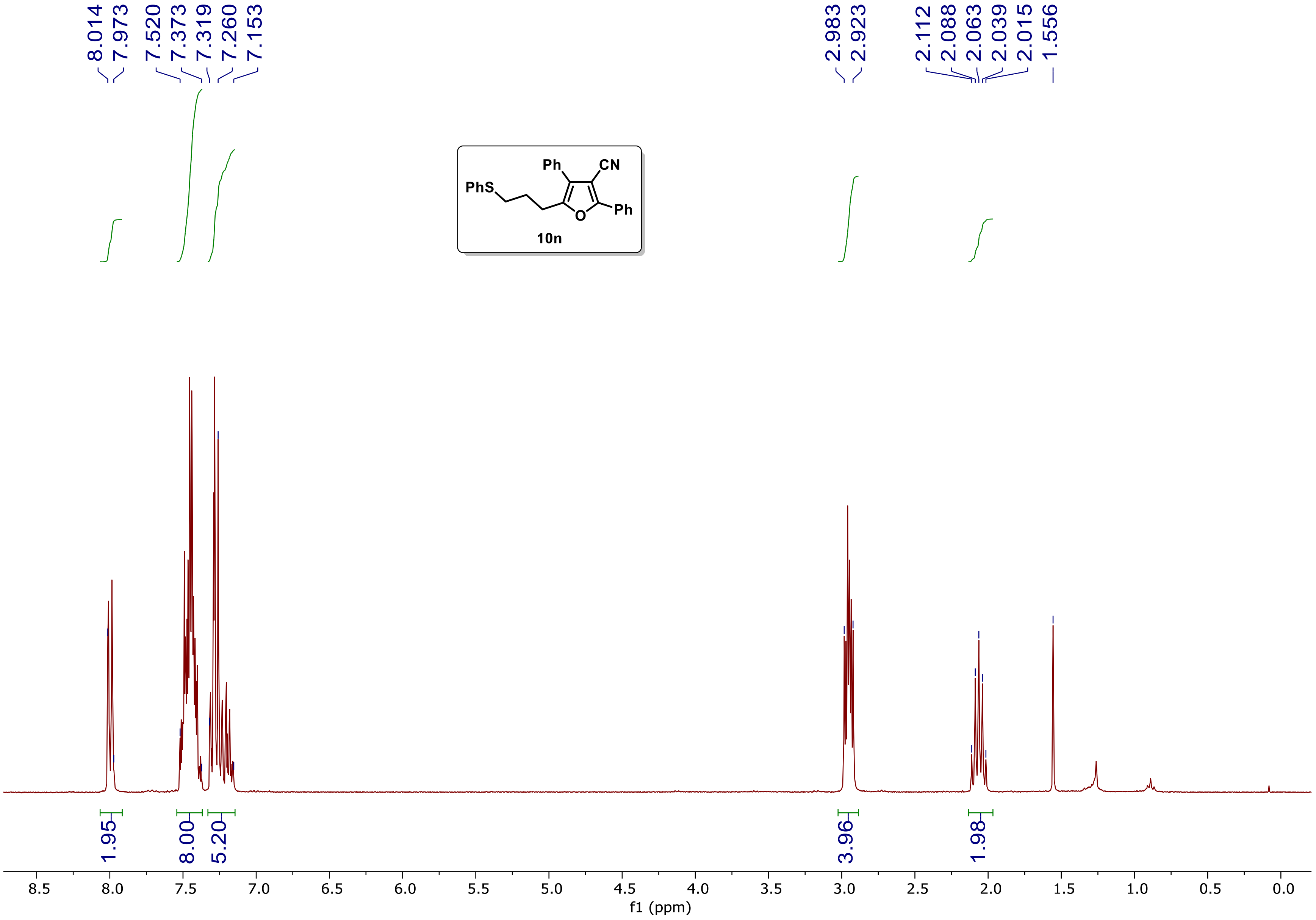
8.014
7.973
7.520
7.373
7.319
7.260
7.153
2.983
2.923
2.112
2.088
2.063
2.039
2.015
1.556
Ph
CN
PhS
Ph
O
10n
1.95
8.00
5.20
3.96
1.98
8.5 8.0 7.5 7.0 6.5 6.0 5.5 5.0 4.5 4.0 3.5 3.0 2.5 2.0 1.5 1.0 0.5 0.0
f1 (ppm)

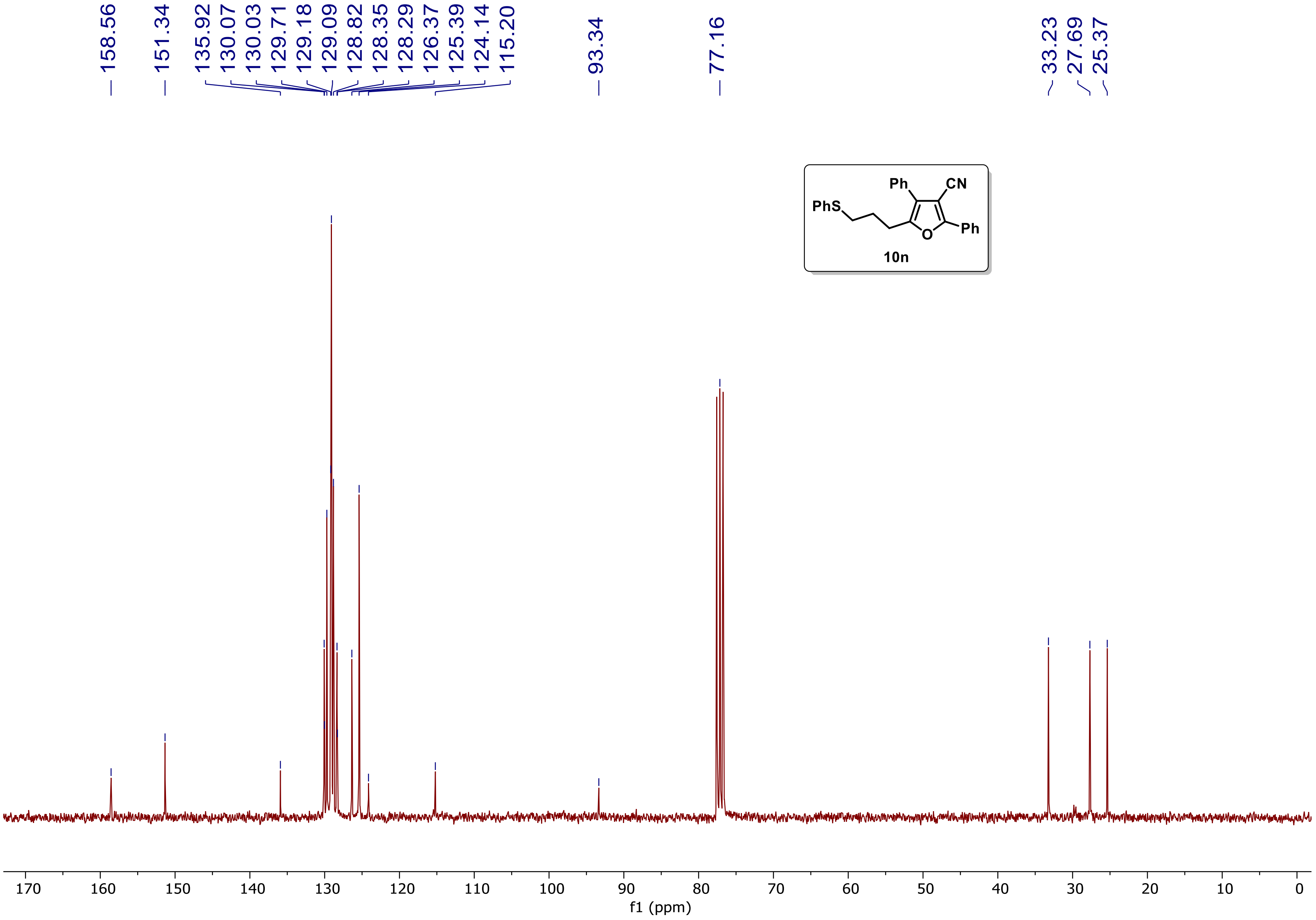

158.56
151.34
135.92
130.07
130.03
129.71
129.18
129.09
128.82
128.35
128.29
126.37
125.39
124.14
115.20
93.34
77.16
33.23
27.69
25.37
PhS
Ph
CN
O
Ph
10n
170
160
150
140
130
120
110
100
90
80
70
60
50
40
30
20
10
0
f1 (ppm)

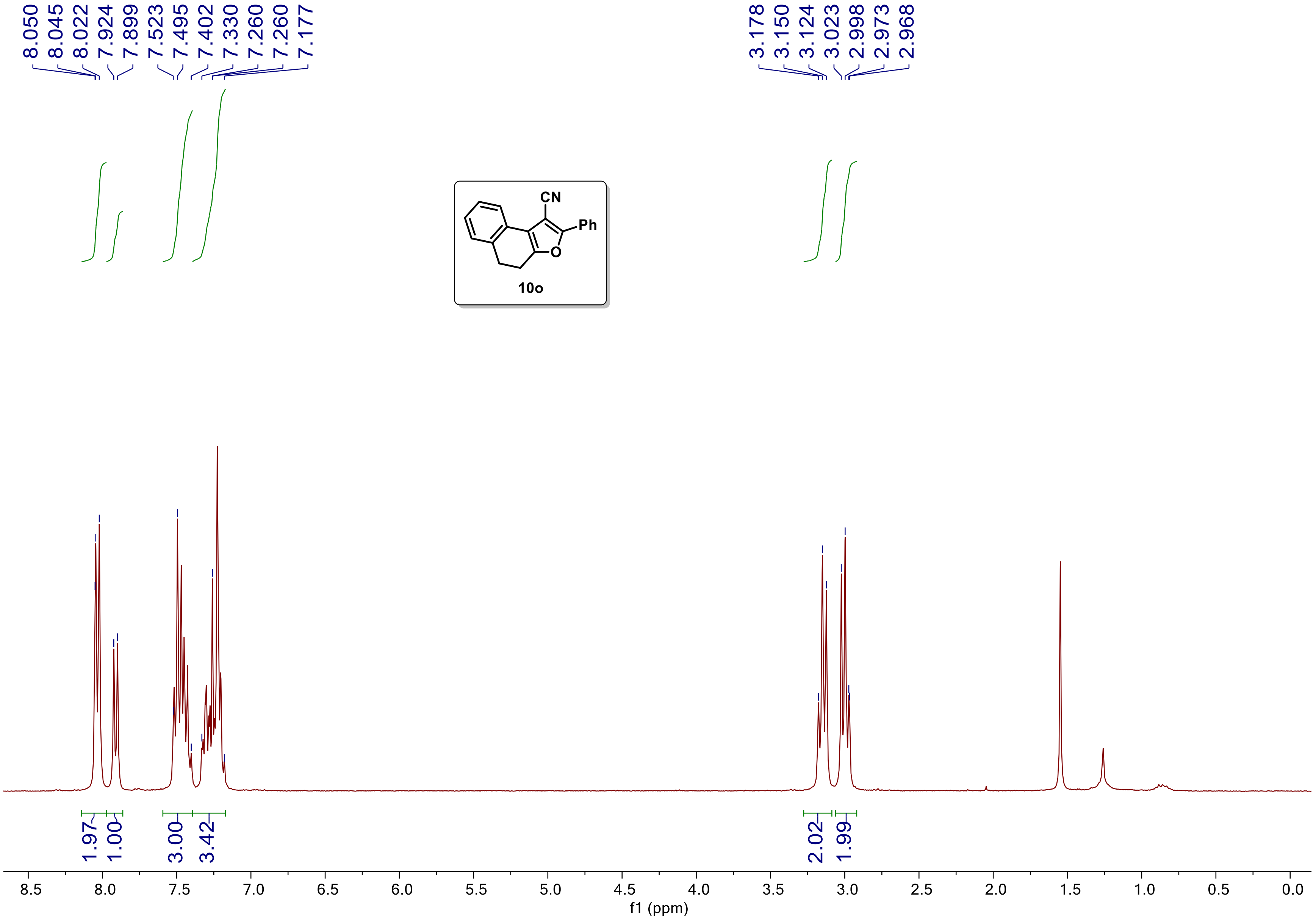
8.050 8.045 8.022 7.924 7.899 7.523 7.495 7.402 7.330 7.260 7.260 7.177
3.178 3.150 3.124 3.023 2.998 2.973 2.968
CN
Ph
O
10o
1.97 1.00 3.00 3.42 2.02 1.99
8.5 8.0 7.5 7.0 6.5 6.0 5.5 5.0 4.5 4.0 3.5 3.0 2.5 2.0 1.5 1.0 0.5 0.0
f1 (ppm)

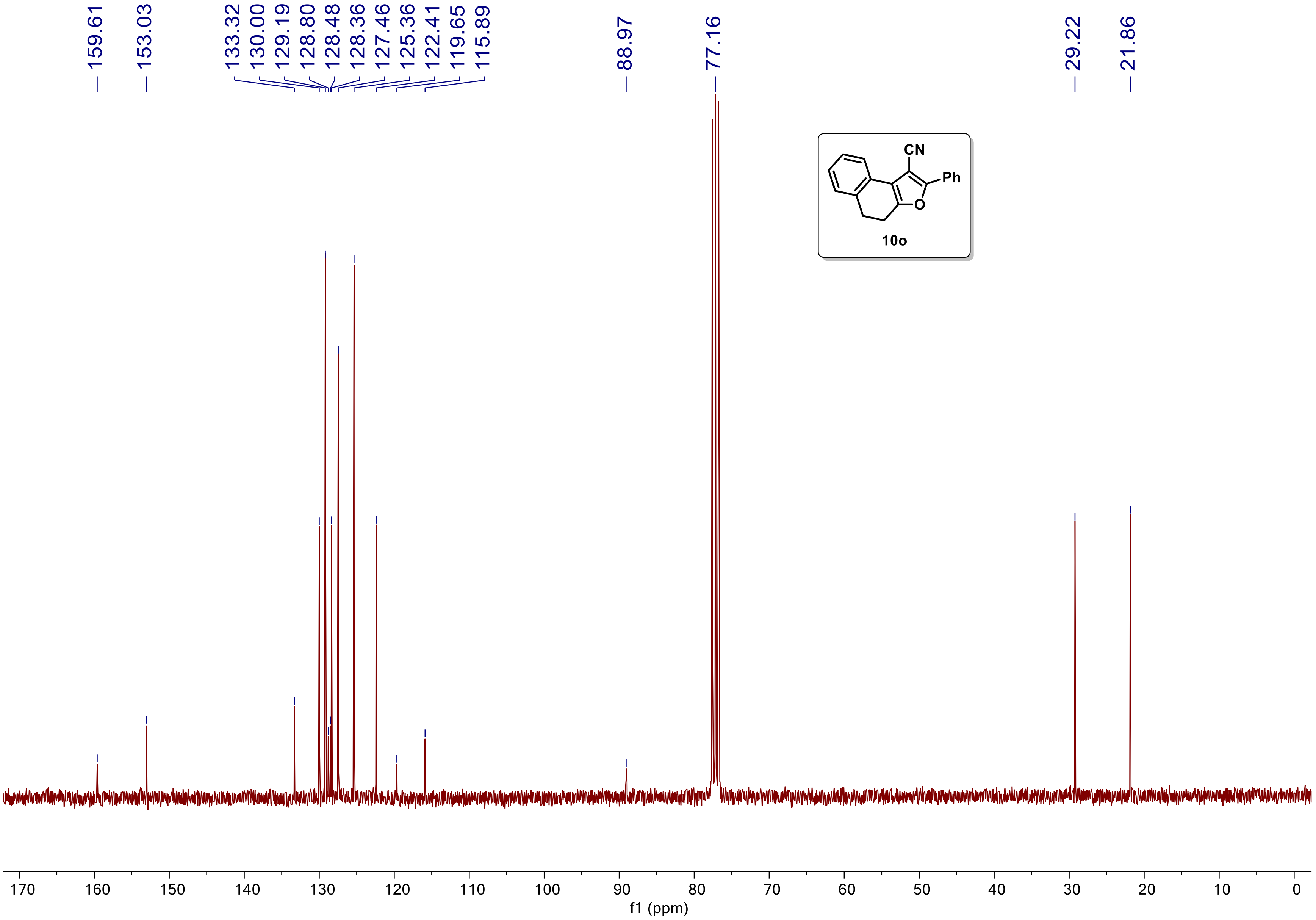
159.61
153.03
133.32
130.00
129.19
128.80
128.48
128.36
127.46
125.36
122.41
119.65
115.89
88.97
77.16
29.22
21.86
CN
Ph
O
10o
170
160
150
140
130
120
110
100
90
80
70
60
50
40
30
20
10
0
f1 (ppm)

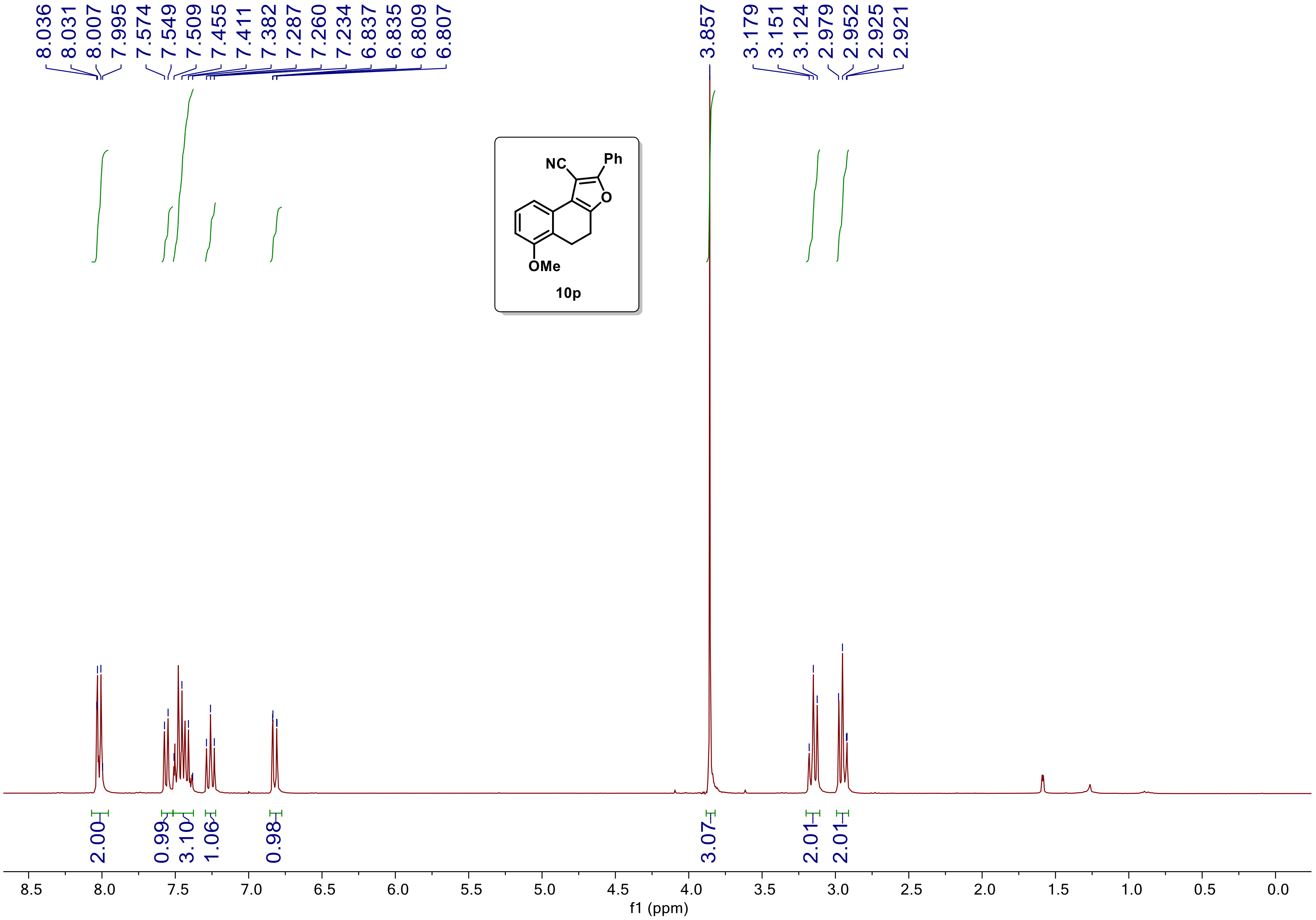
8.036 8.031 8.007 7.995 7.574 7.549 7.509 7.455 7.411 7.382 7.287 7.260 7.234 6.837 6.835 6.809 6.807
3.857
3.179 3.151 3.124 2.979 2.952 2.925 2.921
NC
Ph
O
OMe
10p
2.00
0.99
3.10
1.06
0.98
3.07
2.01
2.01
8.5 8.0 7.5 7.0 6.5 6.0 5.5 5.0 4.5 4.0 3.5 3.0 2.5 2.0 1.5 1.0 0.5 0.0
f1 (ppm)

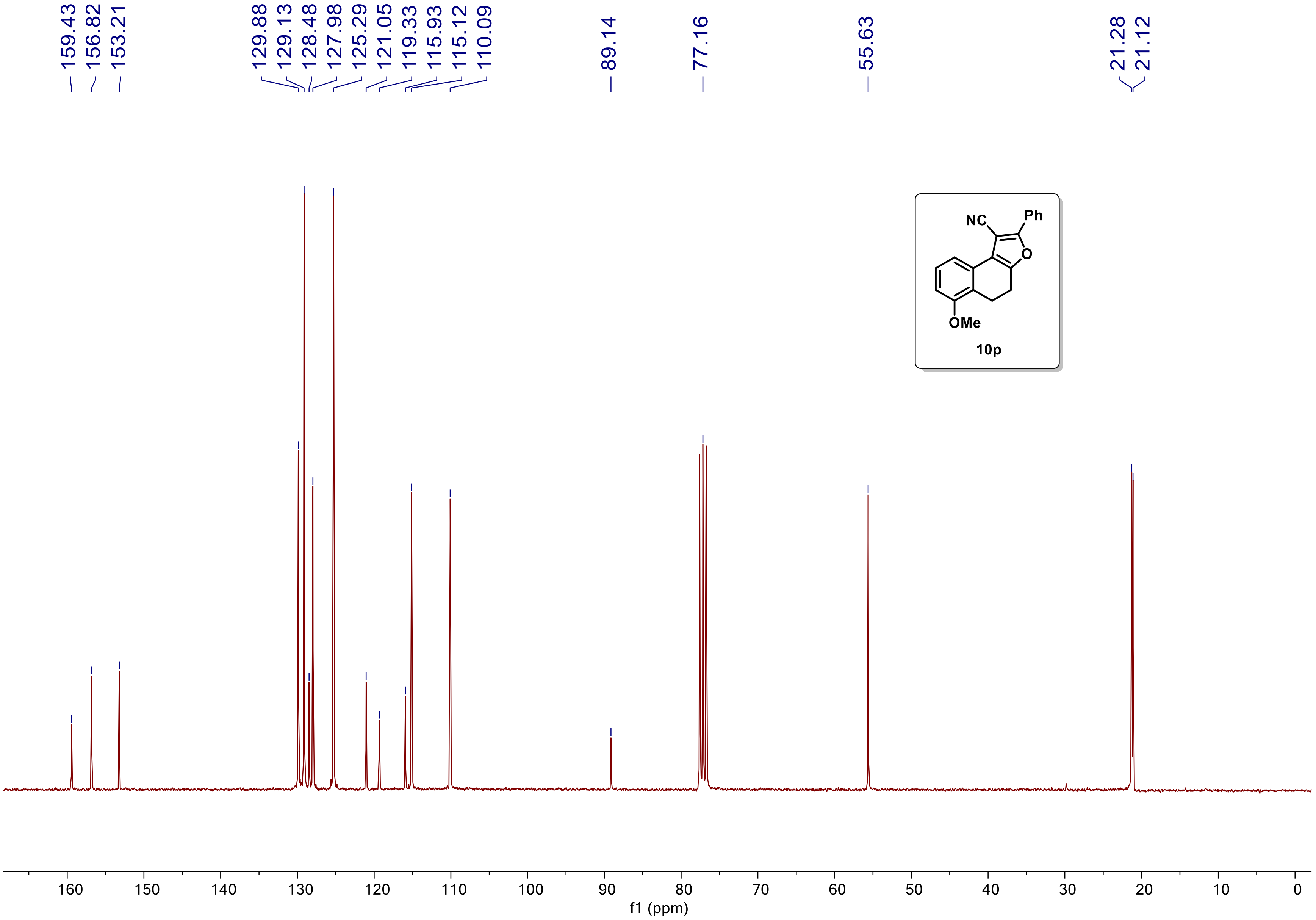
159.43
156.82
153.21
129.88
129.13
128.48
127.98
125.29
121.05
119.33
115.93
115.12
110.09
89.14
77.16
55.63
21.28
21.12
NC
Ph
O
OMe
10p
160
150
140
130
120
110
100
90
80
70
60
50
40
30
20
10
0
f1 (ppm)

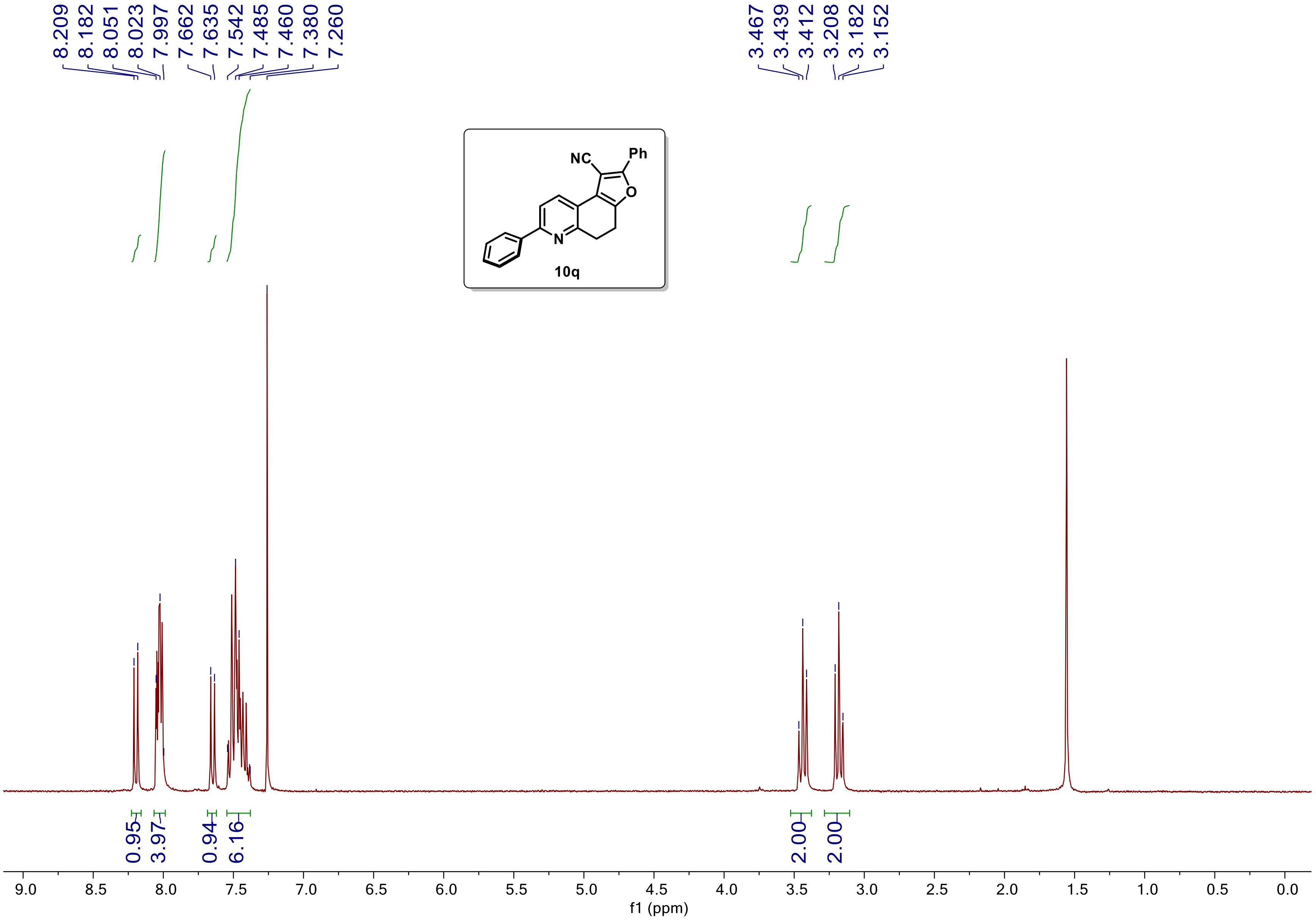
8.209
8.182
8.051
8.023
7.997
7.662
7.635
7.542
7.485
7.460
7.380
7.260
3.467
3.439
3.412
3.208
3.182
3.152
NC
Ph
O
N
10q
0.95
3.97
0.94
6.16
2.00
2.00
9.0 8.5 8.0 7.5 7.0 6.5 6.0 5.5 5.0 4.5 4.0 3.5 3.0 2.5 2.0 1.5 1.0 0.5 0.0
f1 (ppm)

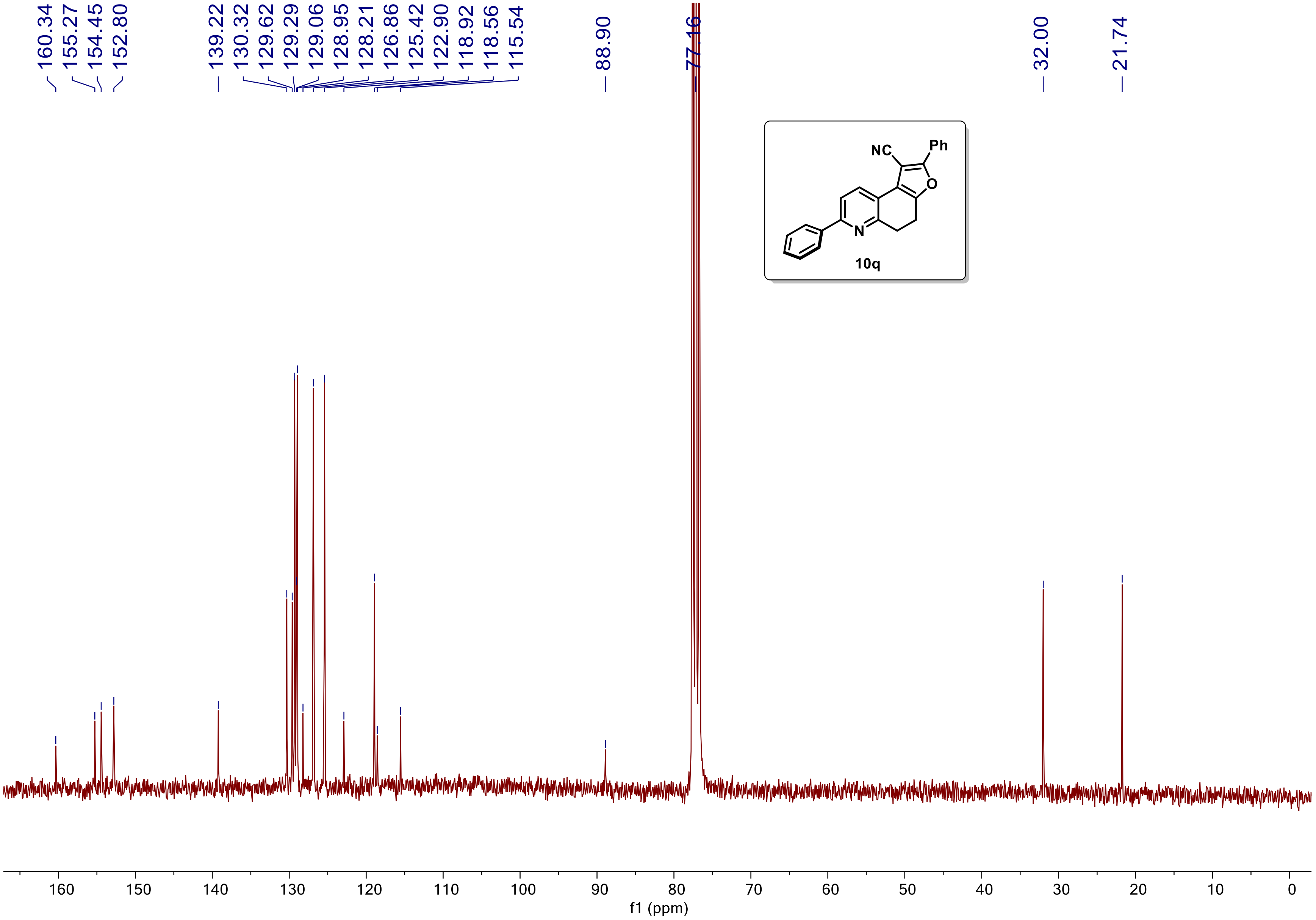
160.34
155.27
154.45
152.80
139.22
130.32
129.62
129.29
129.06
128.95
128.21
126.86
125.42
122.90
118.92
118.56
115.54
88.90
77.16
32.00
21.74
NC
Ph
O
N
10q
160
150
140
130
120
110
100
90
80
70
60
50
40
30
20
10
0
f1 (ppm)

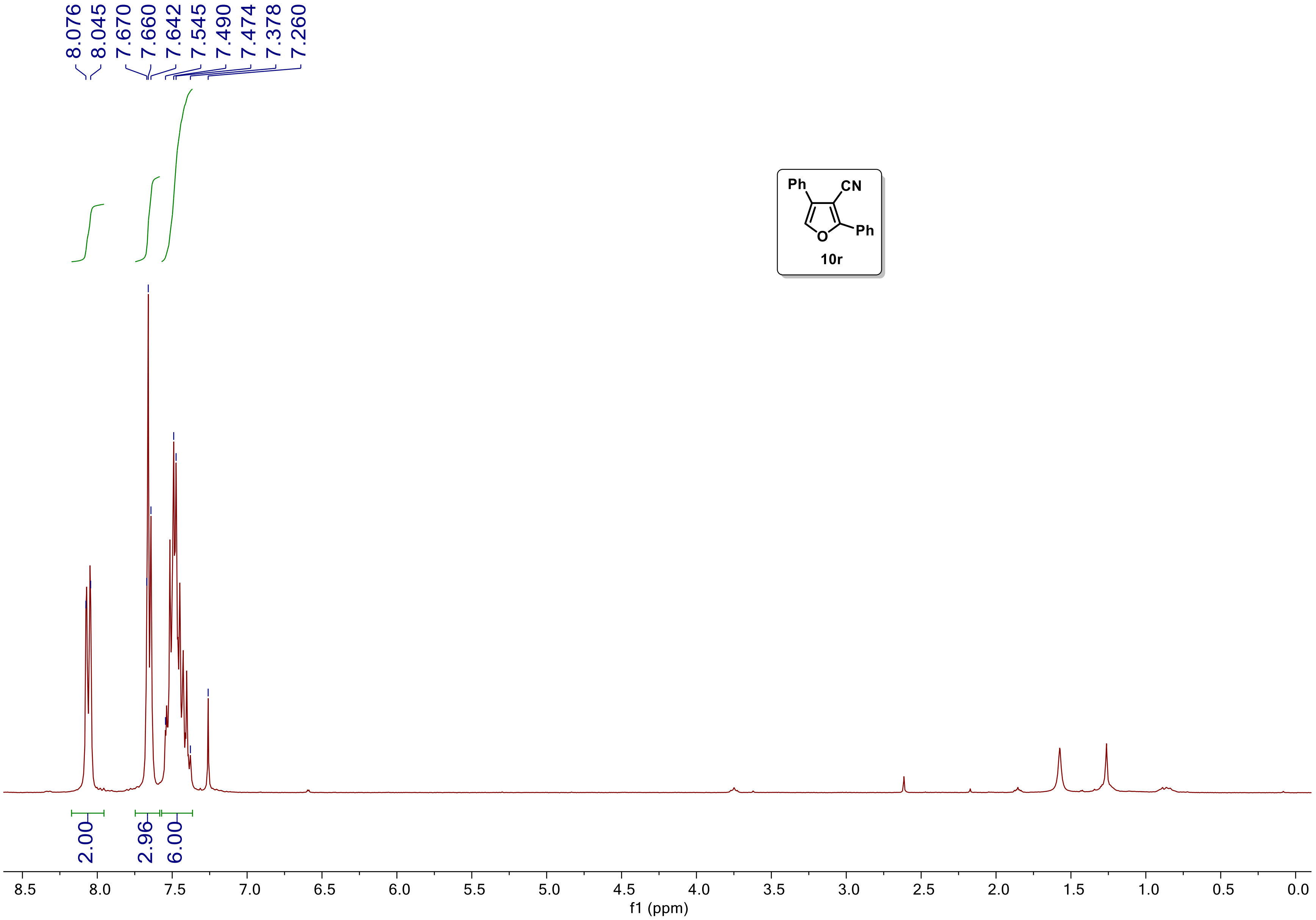
8.076
8.045
7.670
7.660
7.642
7.545
7.490
7.474
7.378
7.260
Ph
CN
O
Ph
10r
2.00
2.96
6.00
8.5 8.0 7.5 7.0 6.5 6.0 5.5 5.0 4.5 4.0 3.5 3.0 2.5 2.0 1.5 1.0 0.5 0.0
f1 (ppm)

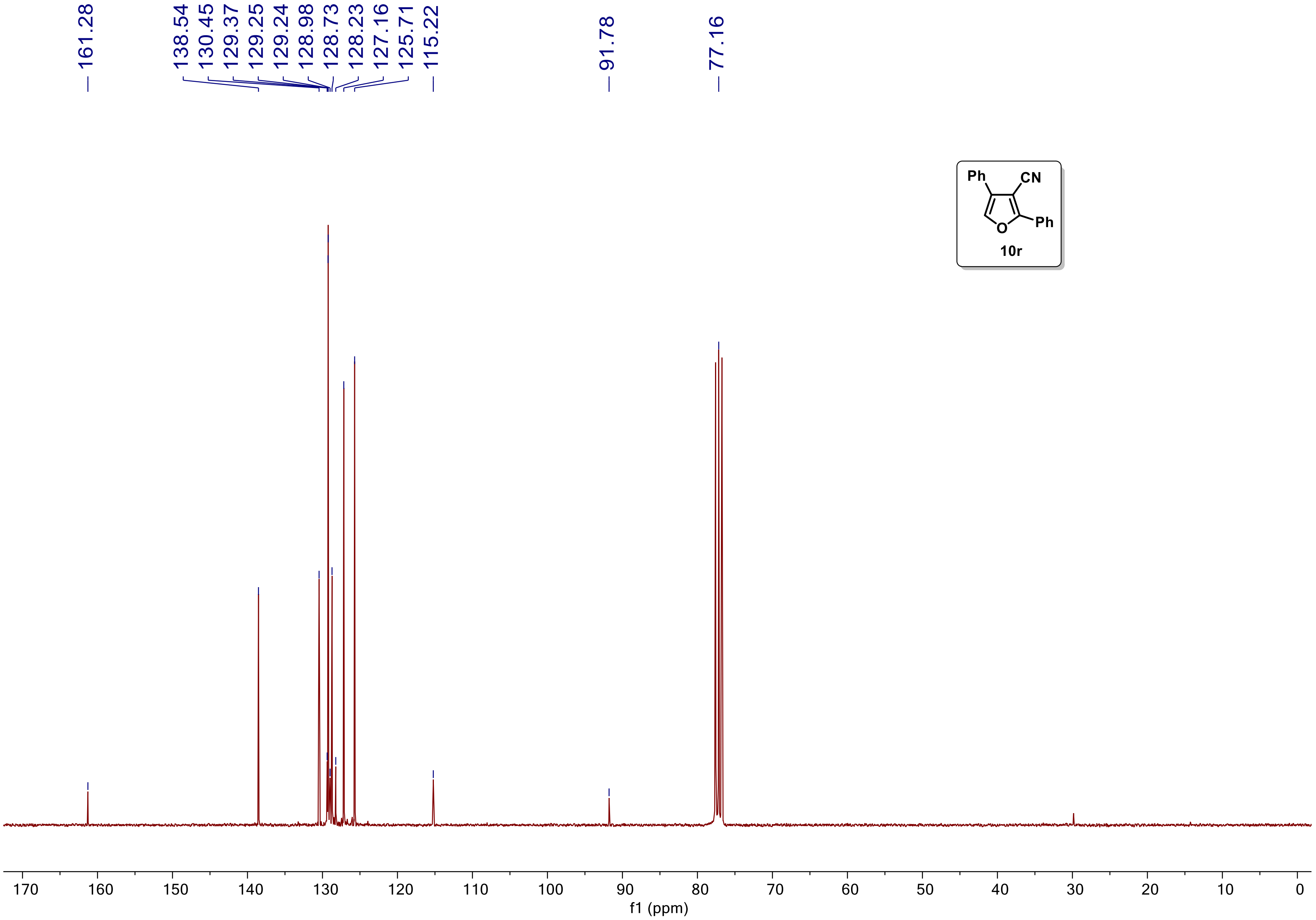

161.28
138.54
130.45
129.37
129.25
129.24
128.98
128.73
128.23
127.16
125.71
115.22
91.78
77.16
Ph
CN
O
Ph
10r
170
160
150
140
130
120
110
100
90
80
70
60
50
40
30
20
10
0
f1 (ppm)

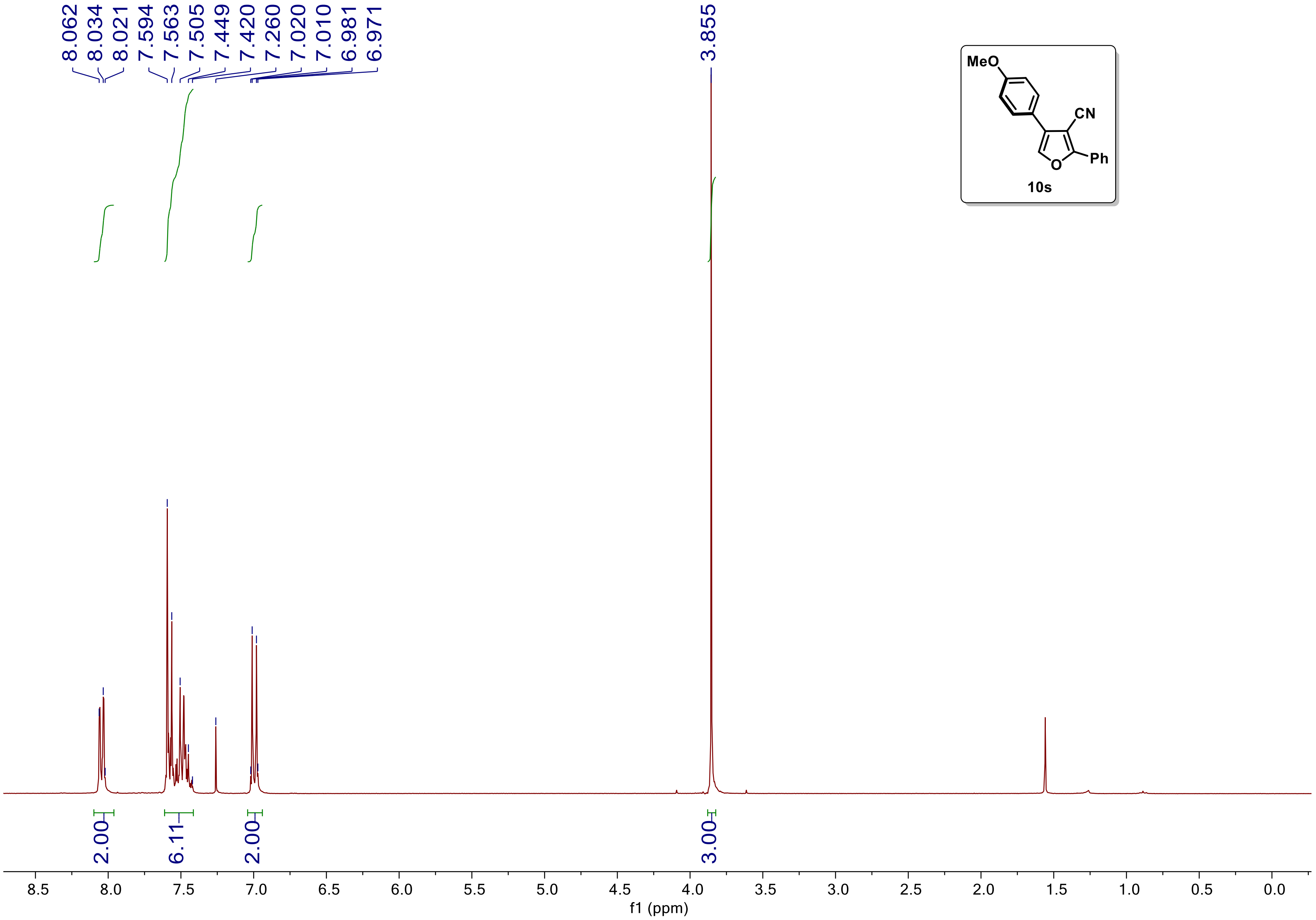
8.062
8.034
8.021
7.594
7.563
7.505
7.449
7.420
7.260
7.020
7.010
6.981
6.971
3.855
MeO
CN
Ph
O
10s
2.00
6.11
2.00
3.00
8.5 8.0 7.5 7.0 6.5 6.0 5.5 5.0 4.5 4.0 3.5 3.0 2.5 2.0 1.5 1.0 0.5 0.0
f1 (ppm)

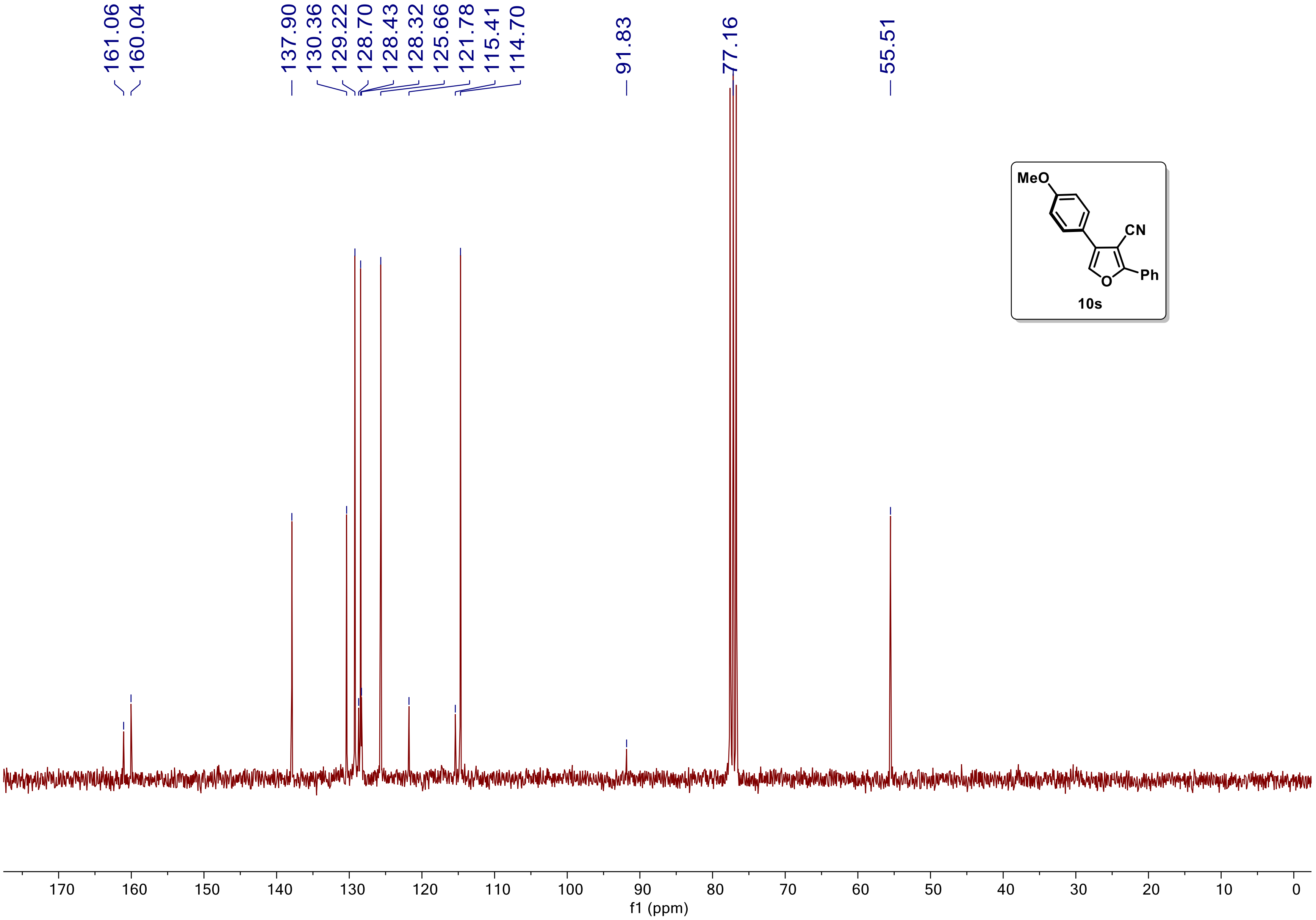
161.06
160.04
137.90
130.36
129.22
128.70
128.43
128.32
125.66
121.78
115.41
114.70
91.83
77.16
55.51
MeO
CN
O
Ph
10s
170
160
150
140
130
120
110
100
90
80
70
60
50
40
30
20
10
0
f1 (ppm)

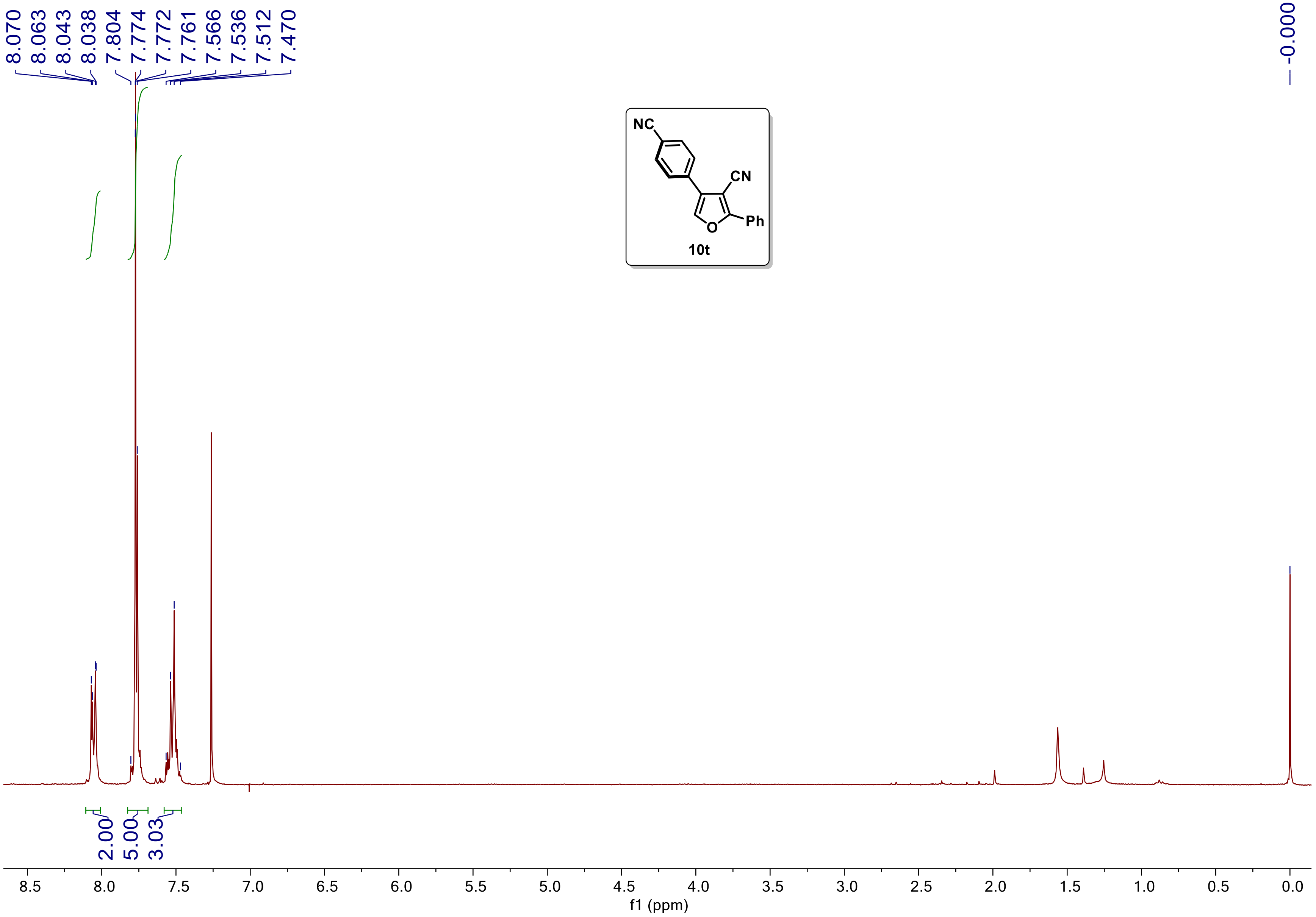
8.070
8.063
8.043
8.038
7.804
7.774
7.772
7.761
7.566
7.536
7.512
7.470
-0.000
NC
CN
O
Ph
10t
2.00
5.00
3.03
8.5
8.0
7.5
7.0
6.5
6.0
5.5
5.0
4.5
4.0
3.5
3.0
2.5
2.0
1.5
1.0
0.5
0.0
f1 (ppm)

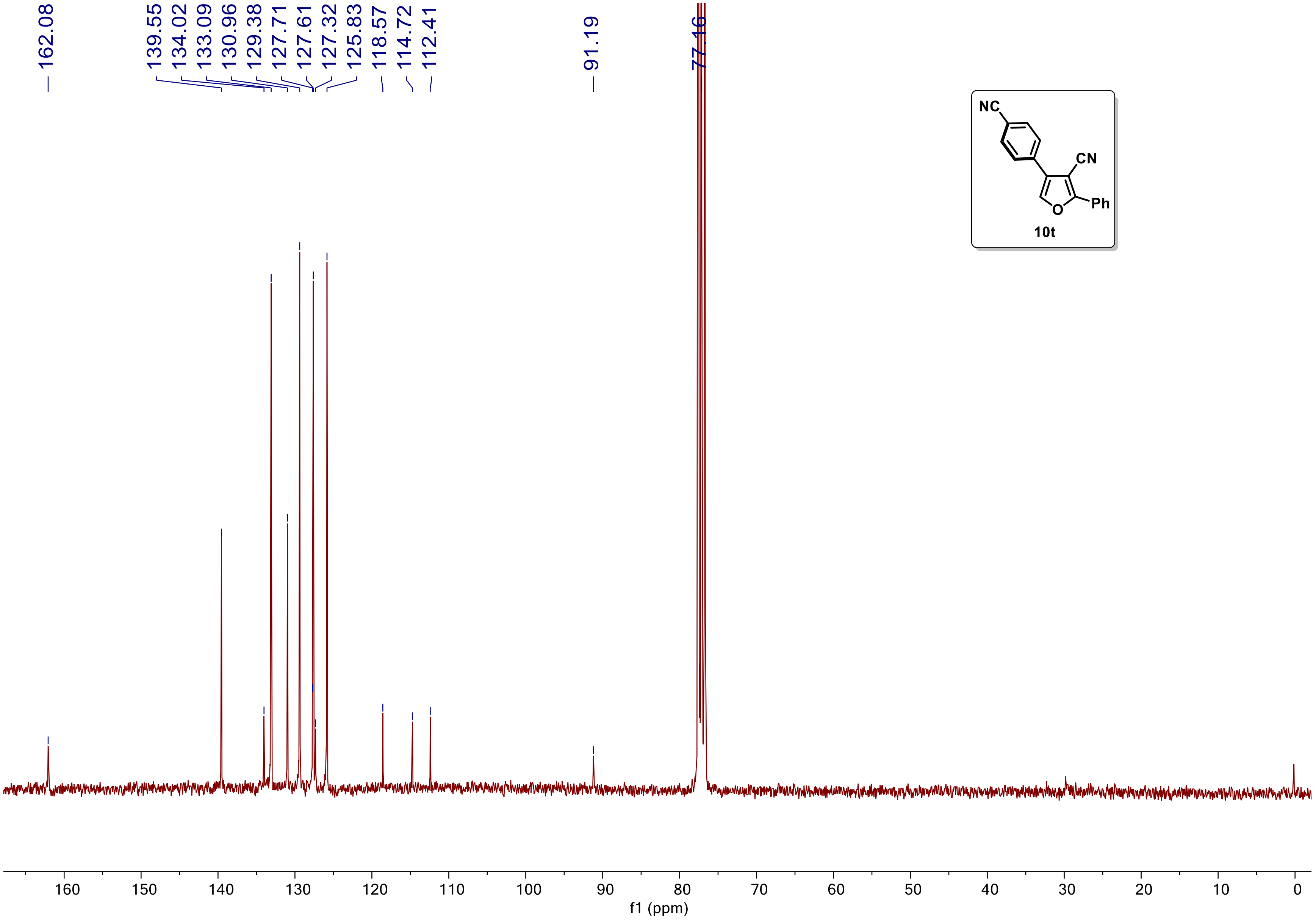
162.08
139.55
134.02
133.09
130.96
129.38
127.71
127.61
127.32
125.83
118.57
114.72
112.41
91.19
77.16
NC
CN
Ph
O
10t
160 150 140 130 120 110 100 90 80 70 60 50 40 30 20 10 0
f1 (ppm)

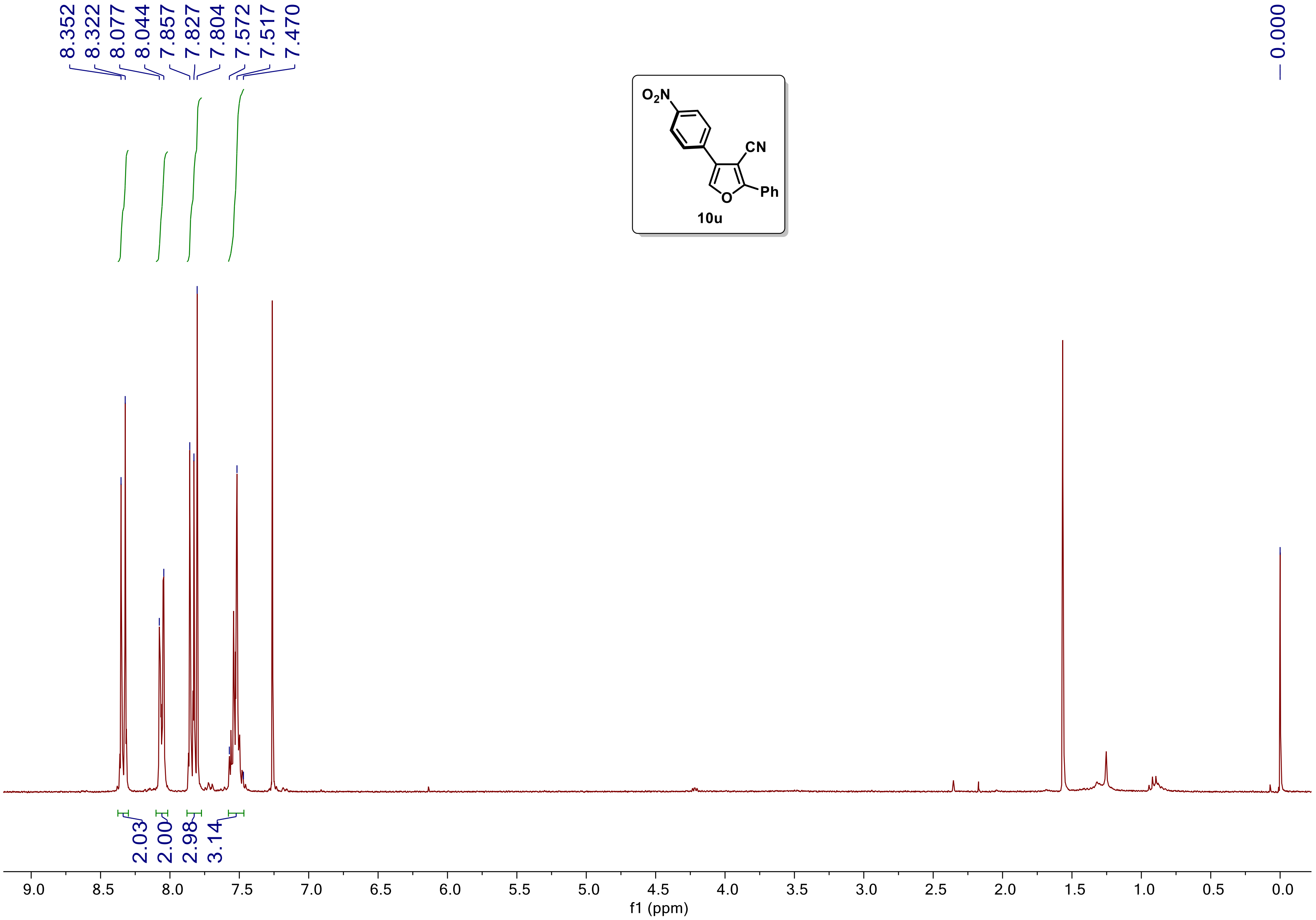
8.352
8.322
8.077
8.044
7.857
7.827
7.804
7.572
7.517
7.470
0.000
O2N
CN
O
Ph
10u
2.03
2.00
2.98
3.14
9.0 8.5 8.0 7.5 7.0 6.5 6.0 5.5 5.0 4.5 4.0 3.5 3.0 2.5 2.0 1.5 1.0 0.5 0.0
f1 (ppm)

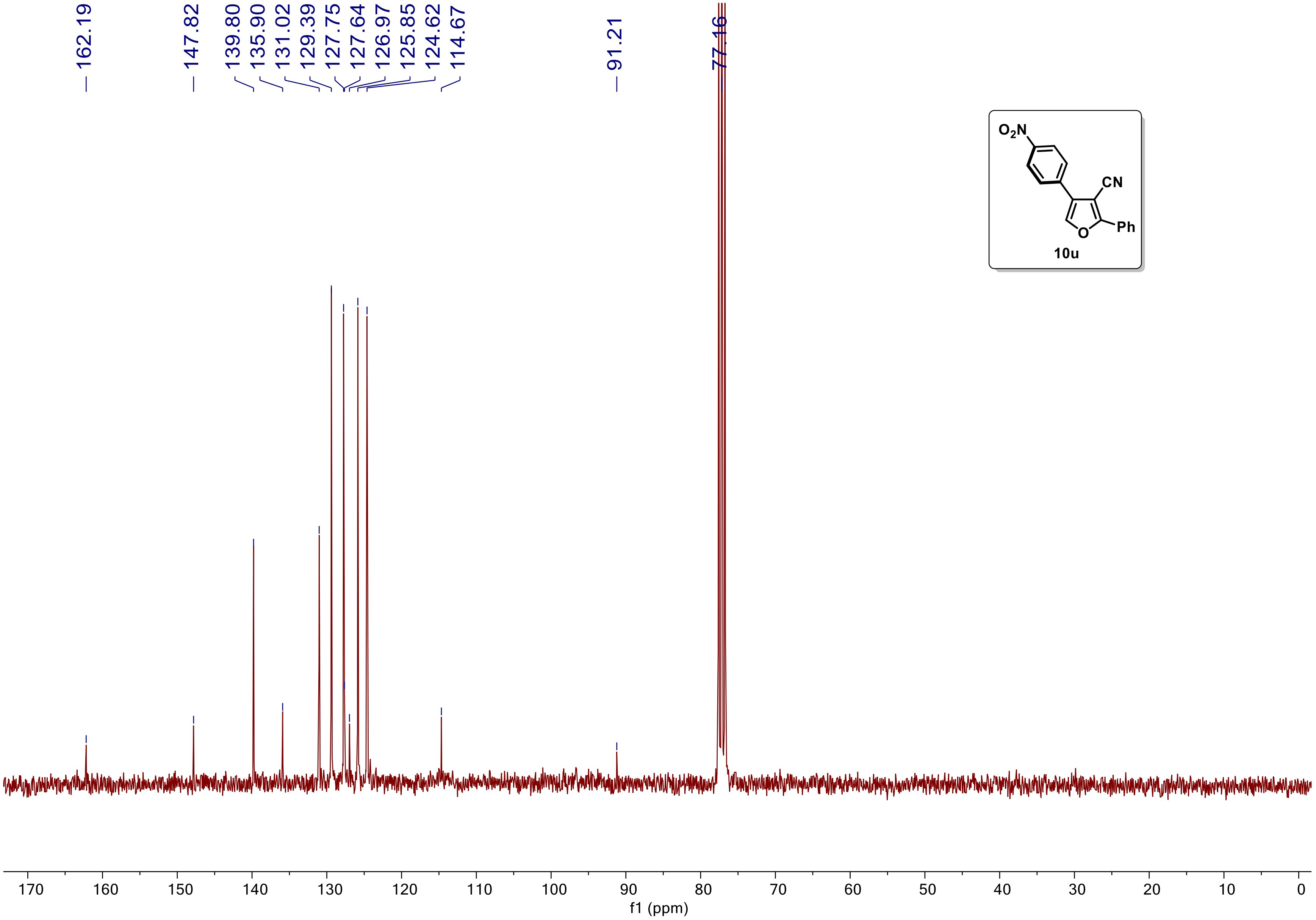
162.19
147.82
139.80
135.90
131.02
129.39
127.75
127.64
126.97
125.85
124.62
114.67
91.21
77.16
170 160 150 140 130 120 110 100 90 80 70 60 50 40 30 20 10 0
f1 (ppm)
O_2N
CN
Ph
O
10u

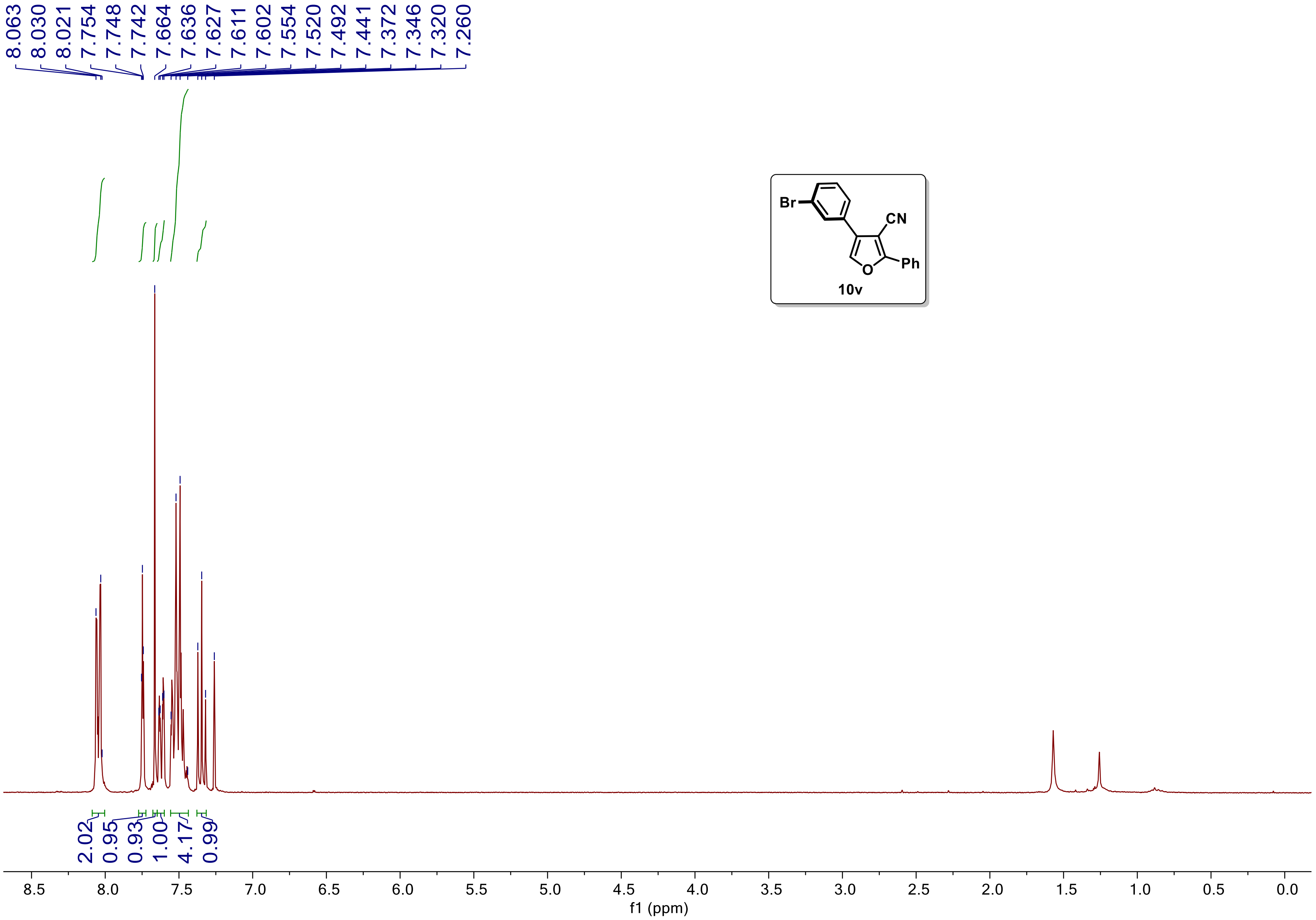

8.063
8.030
8.021
7.754
7.748
7.742
7.664
7.636
7.627
7.611
7.602
7.554
7.520
7.492
7.441
7.372
7.346
7.320
7.260
2.02
0.95
0.93
1.00
4.17
0.99
8.5
8.0
7.5
7.0
6.5
6.0
5.5
5.0
4.5
4.0
3.5
3.0
2.5
2.0
1.5
1.0
0.5
0.0
f1 (ppm)
Br
CN
Ph
O
10v

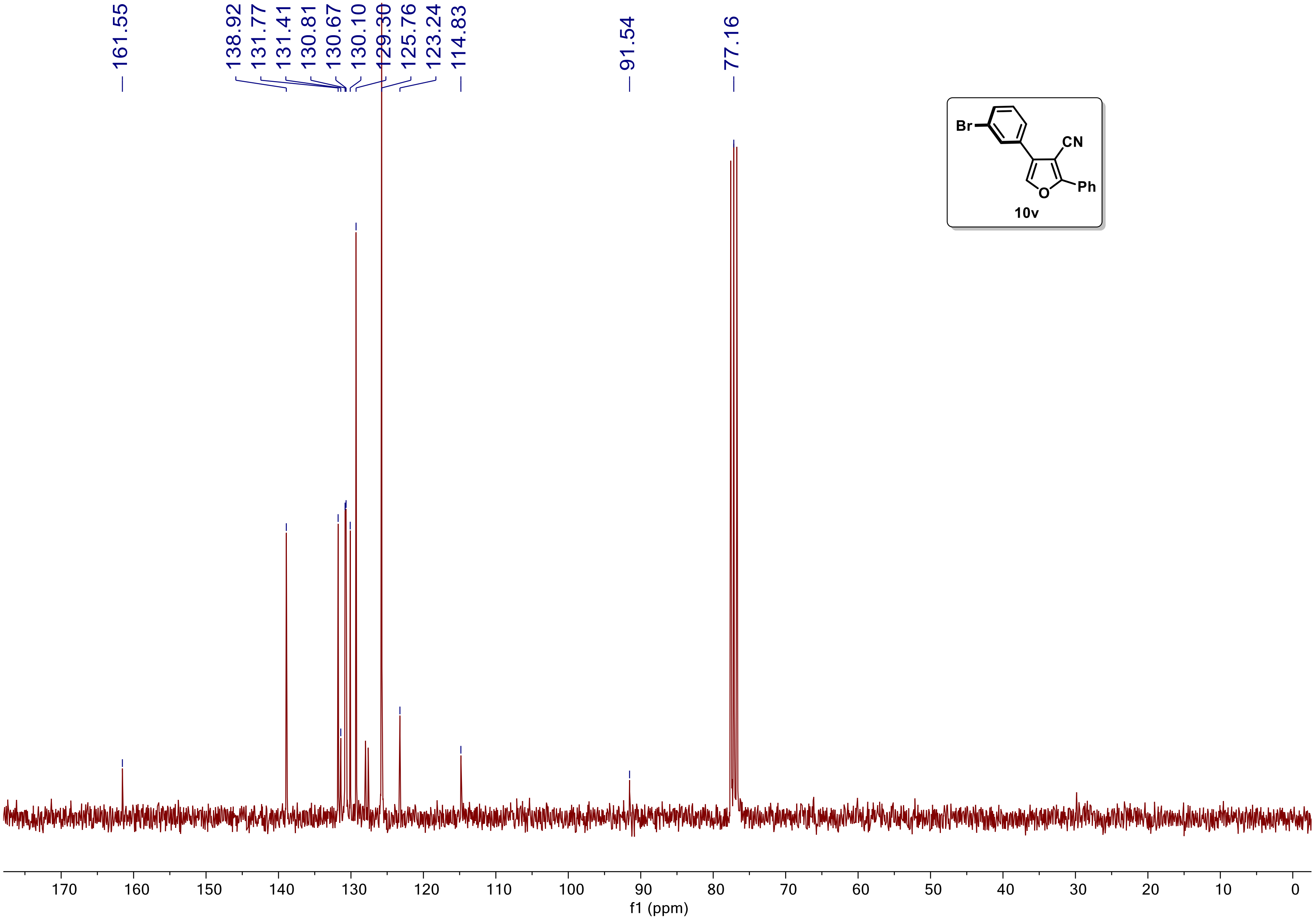
161.55
138.92
131.77
131.41
130.81
130.67
130.10
129.30
125.76
123.24
114.83
91.54
77.16
Br
CN
Ph
O
10v
170
160
150
140
130
120
110
100
90
80
70
60
50
40
30
20
10
0
f1 (ppm)

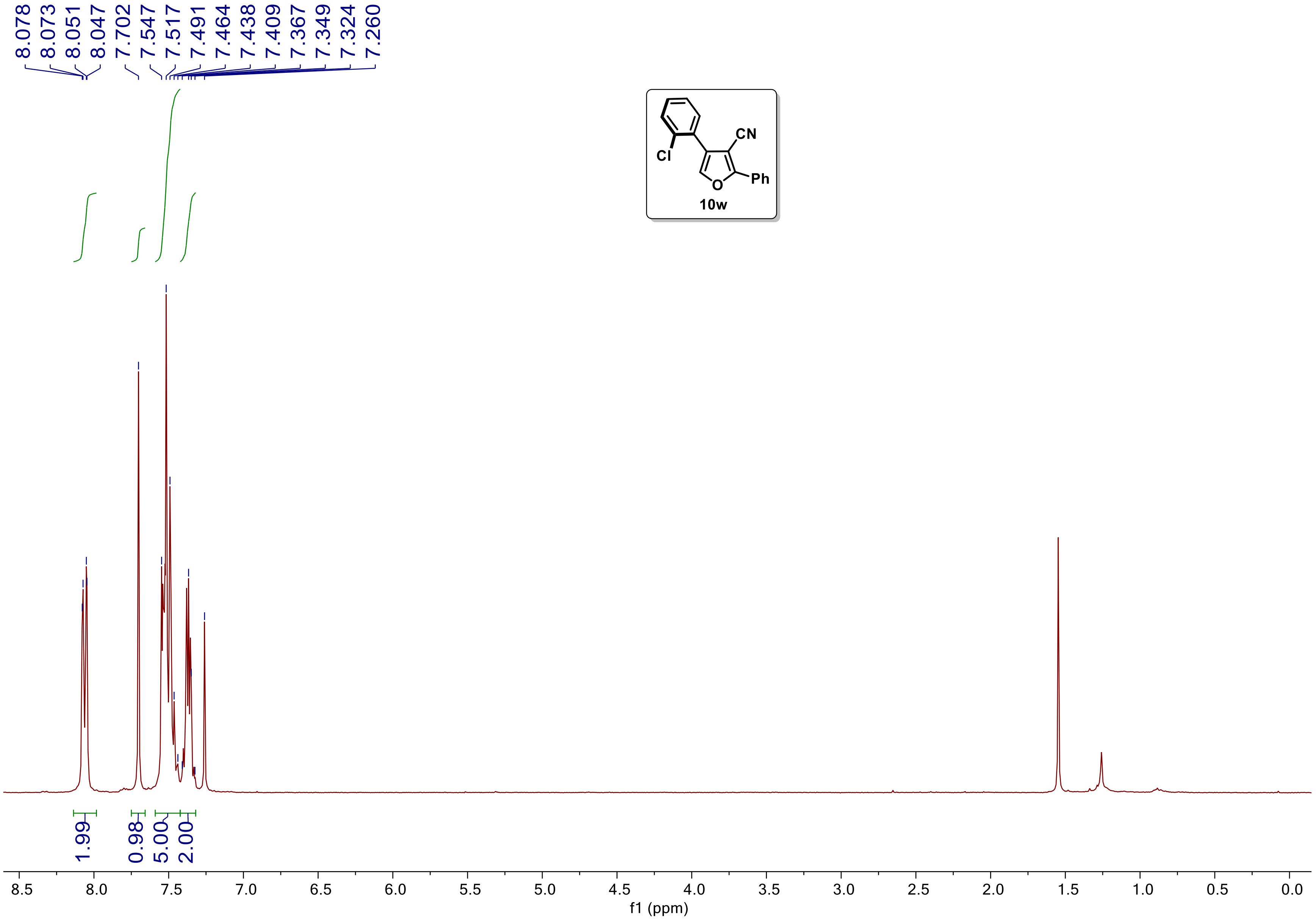
8.078
8.073
8.051
8.047
7.702
7.547
7.517
7.491
7.464
7.438
7.409
7.367
7.349
7.324
7.260
CN
Cl
Ph
O
10w
1.99
0.98
5.00
2.00
8.5 8.0 7.5 7.0 6.5 6.0 5.5 5.0 4.5 4.0 3.5 3.0 2.5 2.0 1.5 1.0 0.5 0.0
f1 (ppm)

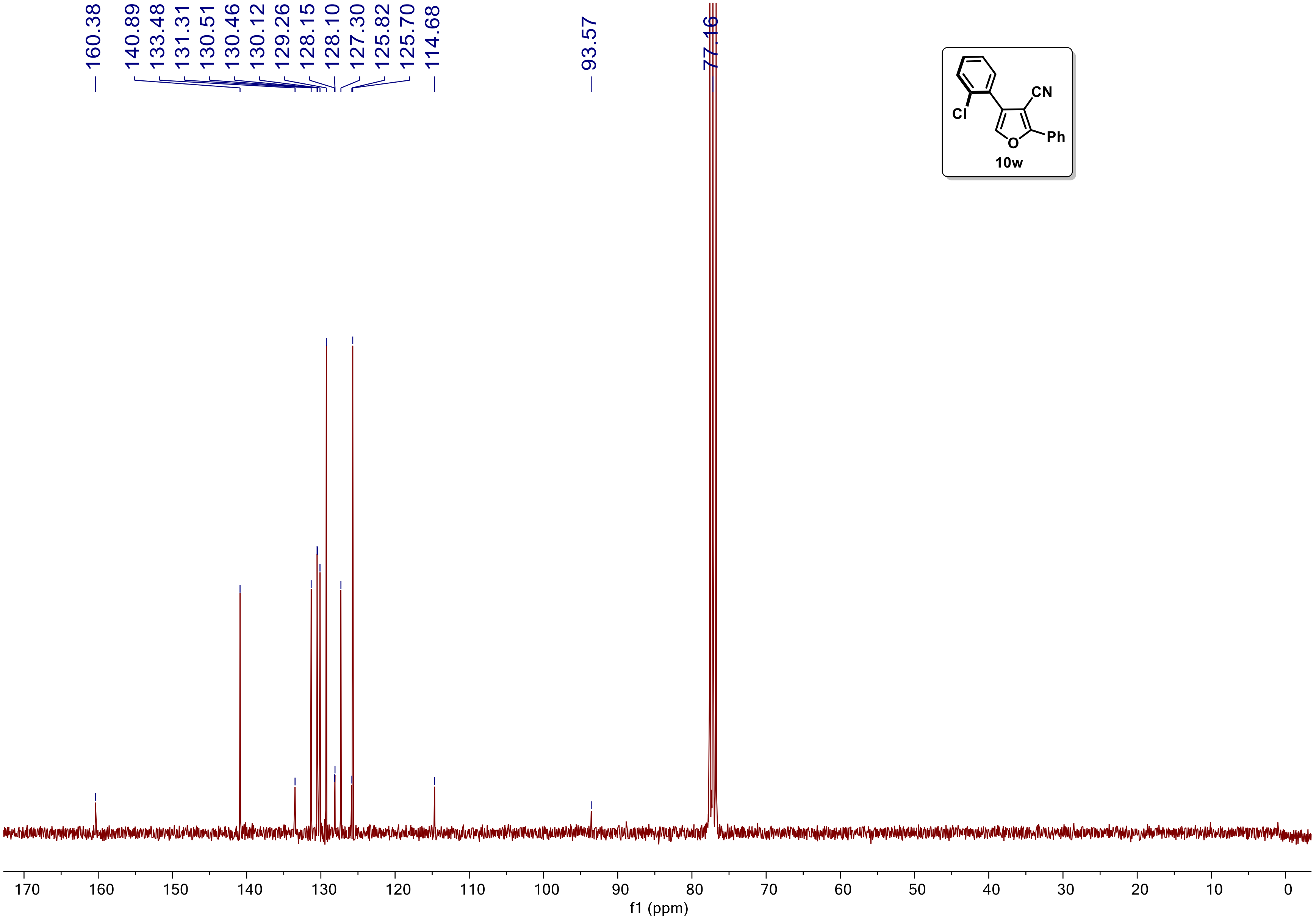
160.38
140.89
133.48
131.31
130.51
130.46
130.12
129.26
128.15
128.10
127.30
125.82
125.70
114.68
93.57
77.16
CN
Cl
Ph
O
10w
170 160 150 140 130 120 110 100 90 80 70 60 50 40 30 20 10 0
f1 (ppm)

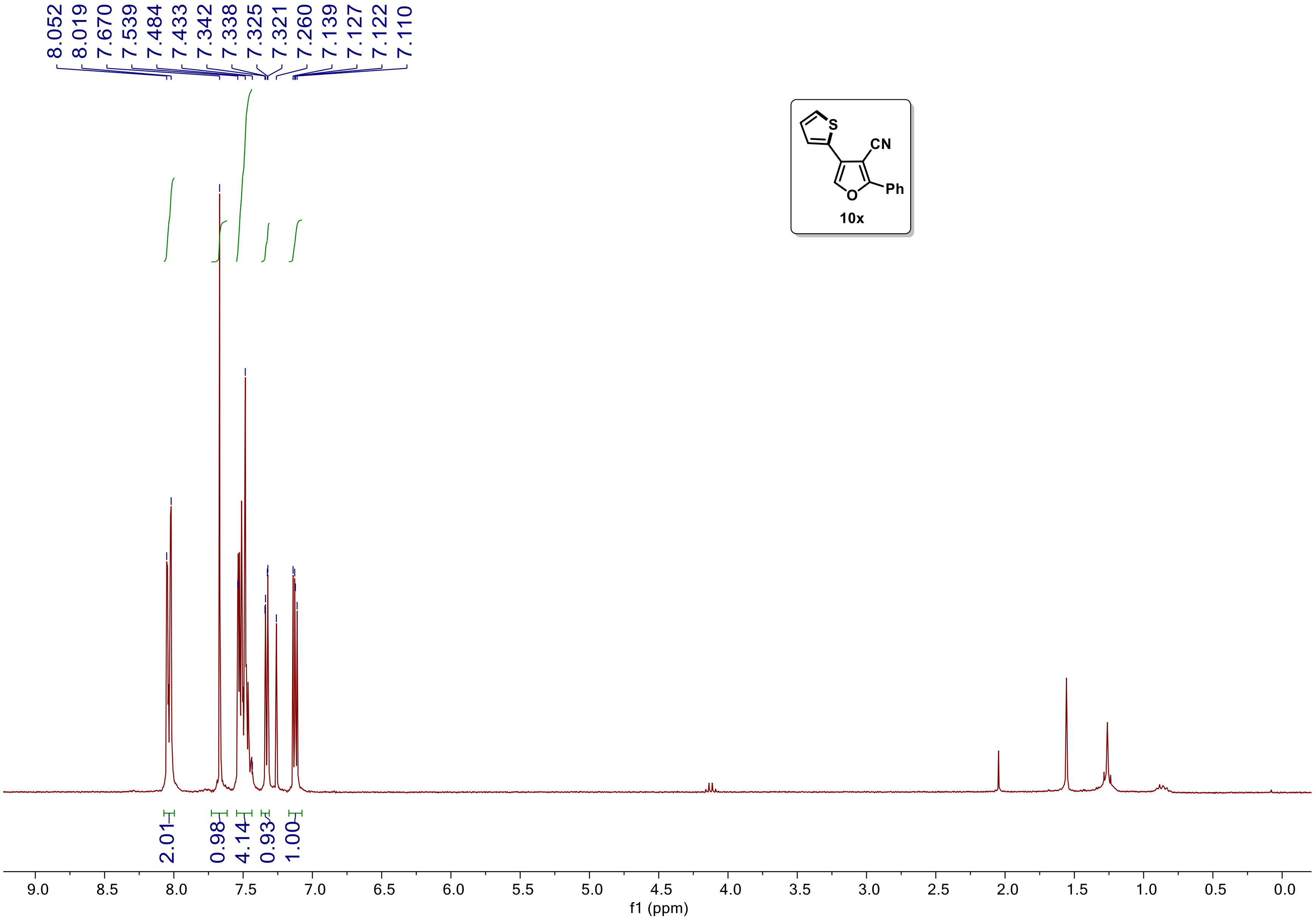
8.052 8.019 7.670 7.539 7.484 7.433 7.342 7.338 7.325 7.321 7.260 7.139 7.127 7.122 7.110
S
CN
O
Ph
10x
2.01
0.98
4.14
0.93
1.00
9.0 8.5 8.0 7.5 7.0 6.5 6.0 5.5 5.0 4.5 4.0 3.5 3.0 2.5 2.0 1.5 1.0 0.5 0.0
f1 (ppm)

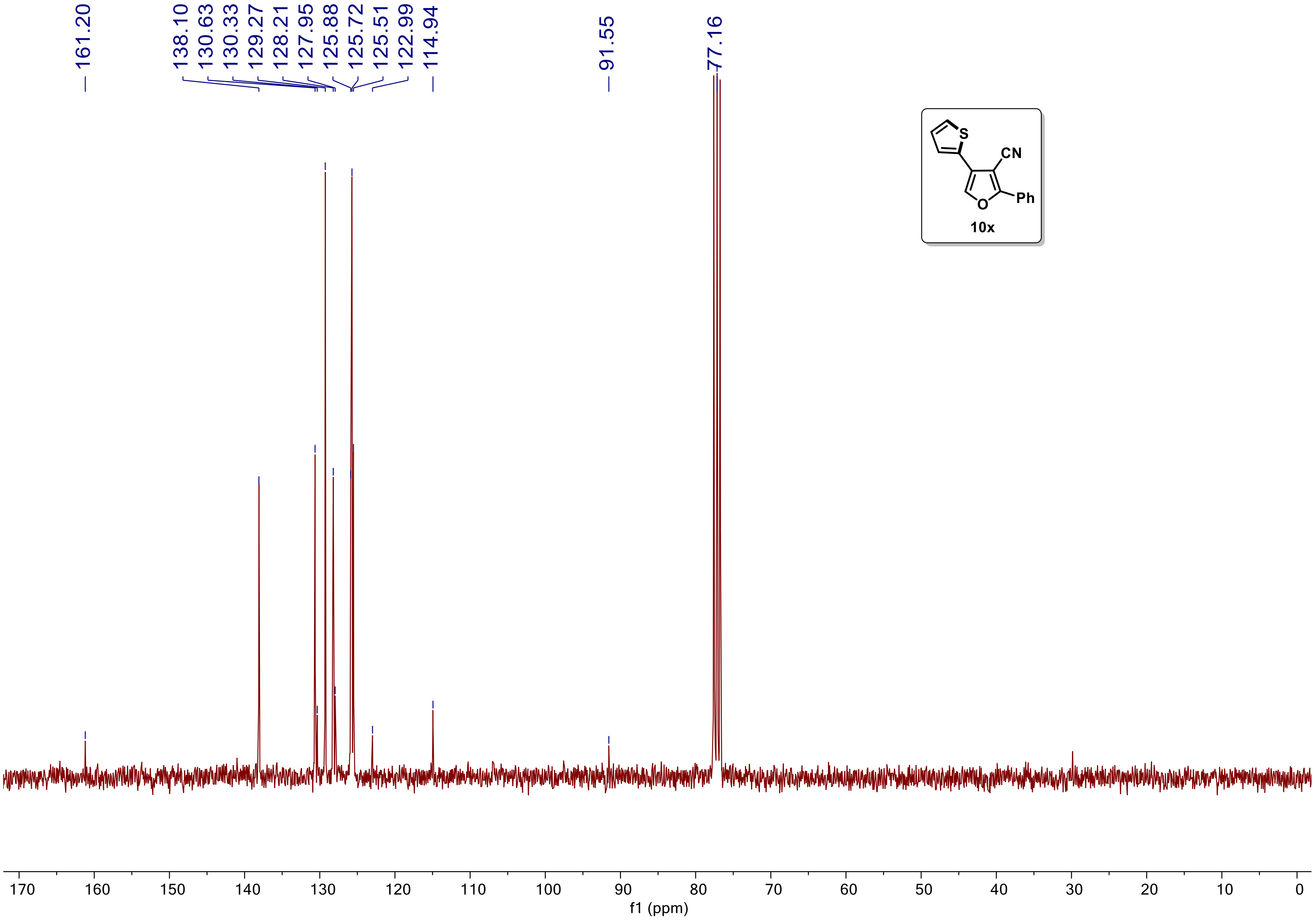
161.20
138.10
130.63
130.33
129.27
128.21
127.95
125.88
125.72
125.51
122.99
114.94
91.55
77.16
S
CN
O
Ph
10x
170 160 150 140 130 120 110 100 90 80 70 60 50 40 30 20 10 0
f1 (ppm)

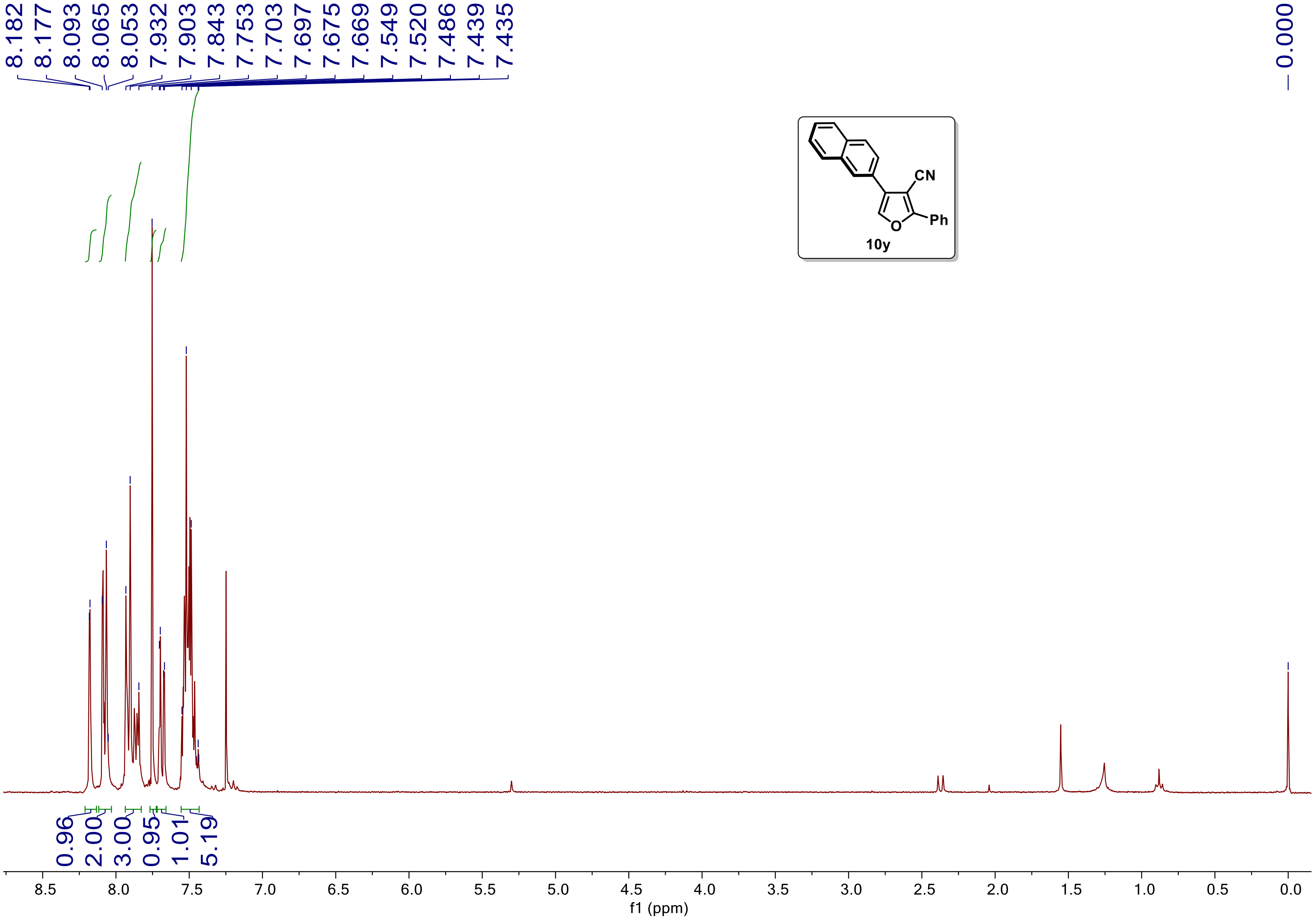
8.182
8.177
8.093
8.065
8.053
7.932
7.903
7.843
7.753
7.703
7.697
7.675
7.669
7.549
7.520
7.486
7.439
7.435
0.000
CN
Ph
O
10y
0.96
2.00
3.00
0.95
1.01
5.19
8.5
8.0
7.5
7.0
6.5
6.0
5.5
5.0
4.5
4.0
3.5
3.0
2.5
2.0
1.5
1.0
0.5
0.0
f1 (ppm)

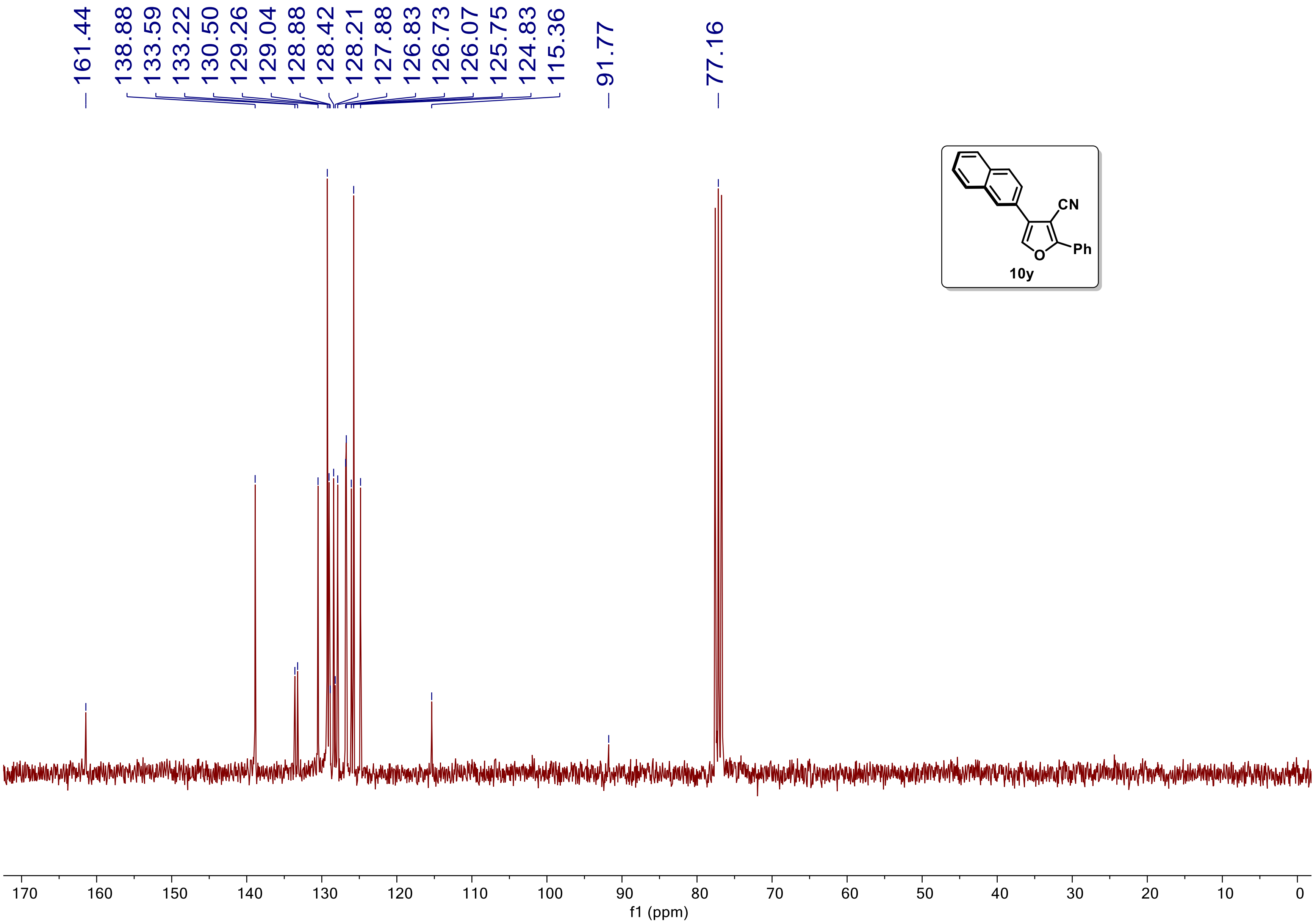

161.44
138.88
133.59
133.22
130.50
129.26
129.04
128.88
128.42
128.21
127.88
126.83
126.73
126.07
125.75
124.83
115.36
91.77
77.16
CN
Ph
O
10y
170
160
150
140
130
120
110
100
90
80
70
60
50
40
30
20
10
0
f1 (ppm)

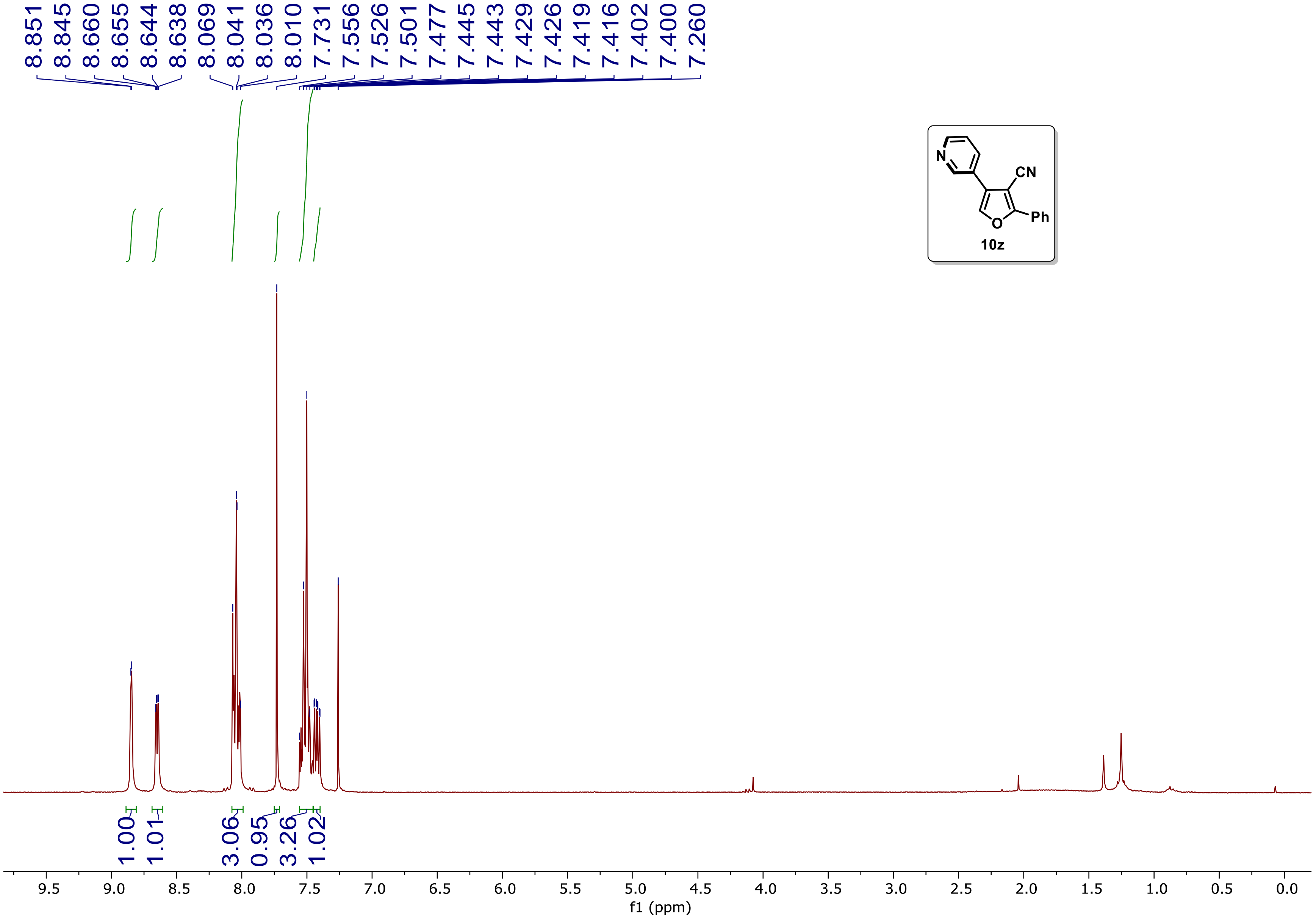
8.851
8.845
8.660
8.655
8.644
8.638
8.069
8.041
8.036
8.010
7.731
7.556
7.526
7.501
7.477
7.445
7.443
7.429
7.426
7.419
7.416
7.402
7.400
7.260
N
CN
Ph
O
10z
1.00
1.01
3.06
0.95
3.26
1.02
9.5 9.0 8.5 8.0 7.5 7.0 6.5 6.0 5.5 5.0 4.5 4.0 3.5 3.0 2.5 2.0 1.5 1.0 0.5 0.0
f1 (ppm)

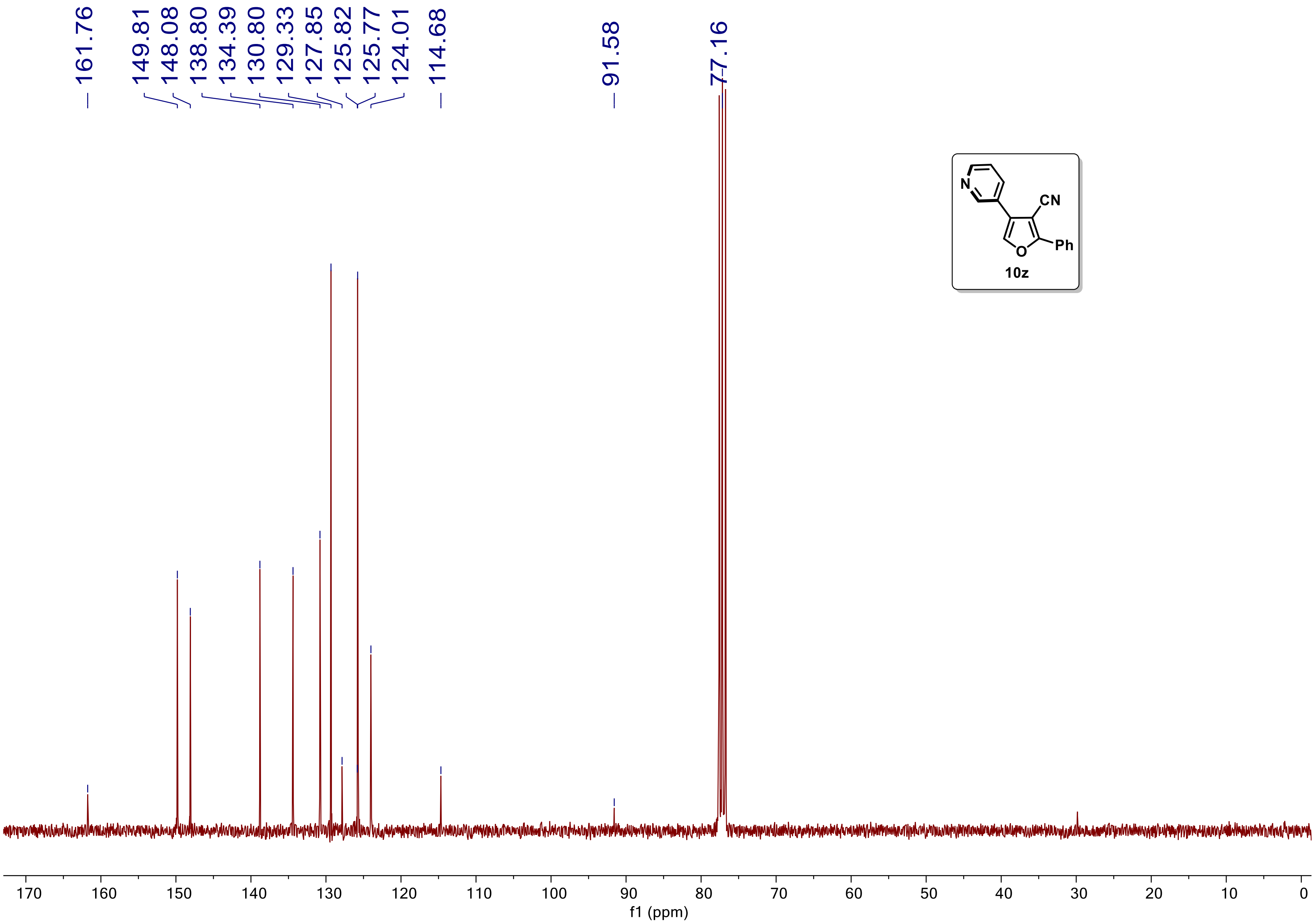
161.76
149.81
148.08
138.80
134.39
130.80
129.33
127.85
125.82
125.77
124.01
114.68
91.58
77.16
N
CN
Ph
O
10z
170 160 150 140 130 120 110 100 90 80 70 60 50 40 30 20 10 0
f1 (ppm)

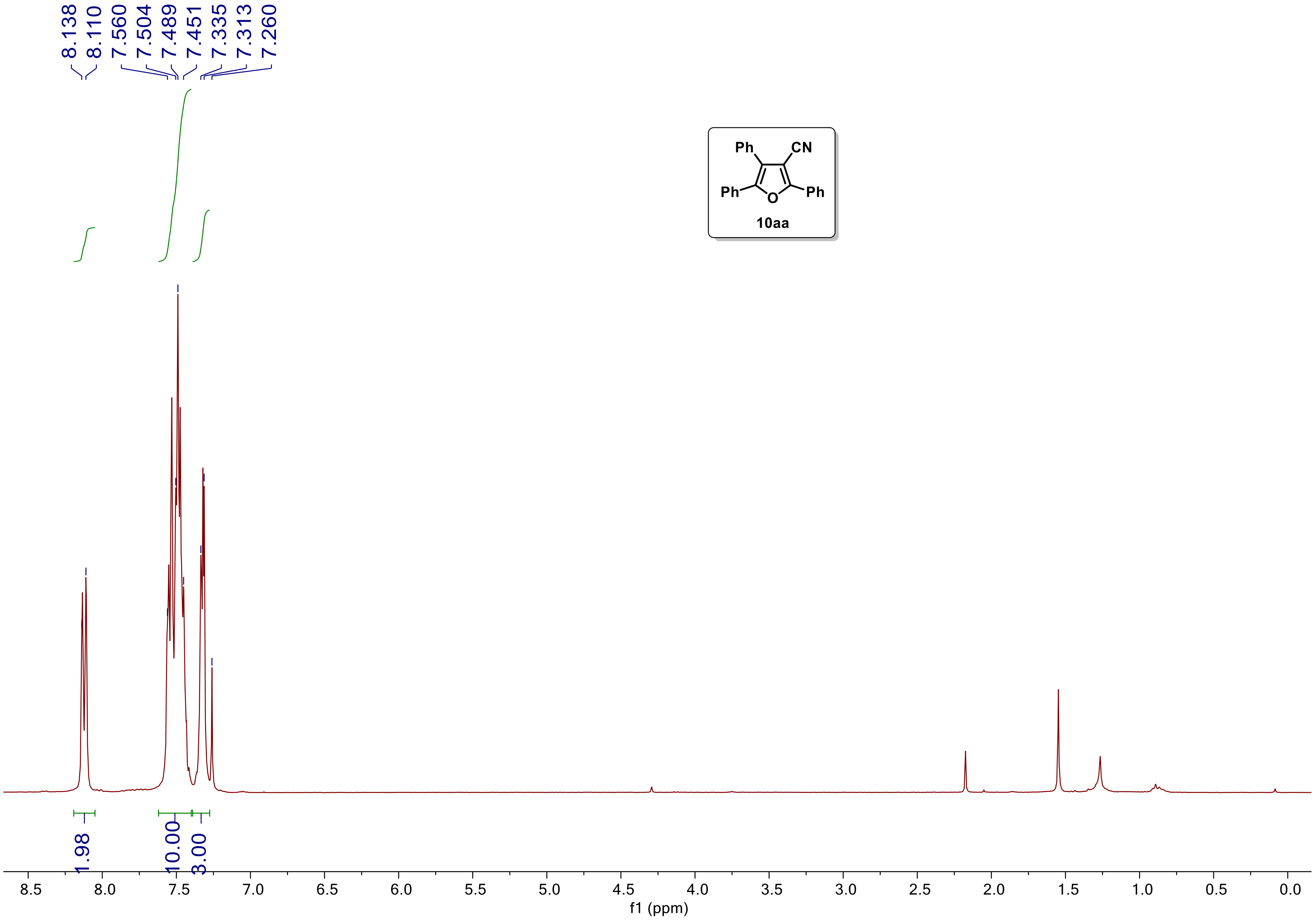

8.138
8.110
7.560
7.504
7.489
7.451
7.335
7.313
7.260
Ph
CN
Ph
O
Ph
10aa
1.98
10.00
3.00
8.5
8.0
7.5
7.0
6.5
6.0
5.5
5.0
4.5
4.0
3.5
3.0
2.5
2.0
1.5
1.0
0.5
0.0
f1 (ppm)

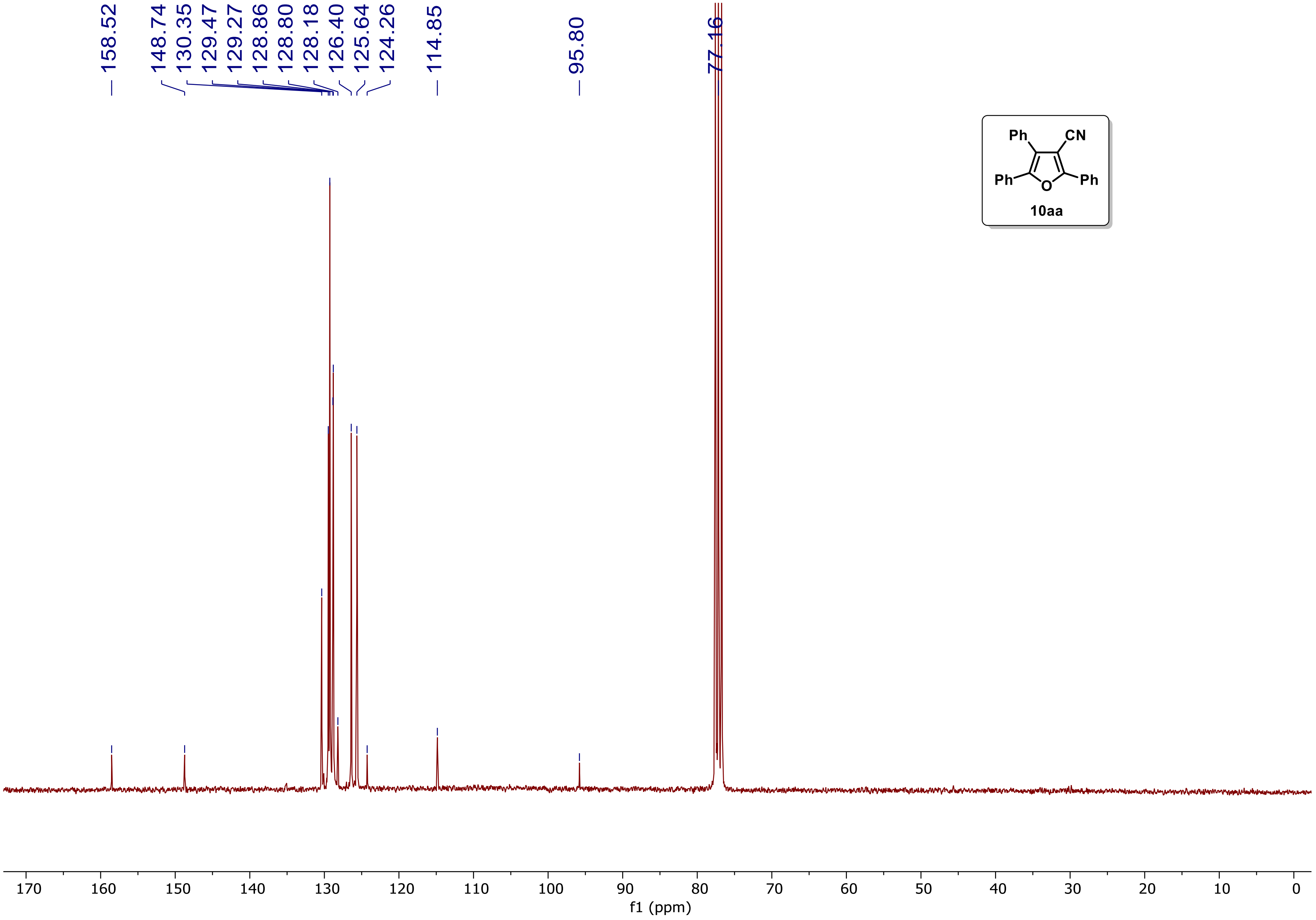
158.52
148.74
130.35
129.47
129.27
128.86
128.80
128.18
126.40
125.64
124.26
114.85
95.80
77.16
Ph
CN
Ph
O
Ph
10aa
170 160 150 140 130 120 110 100 90 80 70 60 50 40 30 20 10 0
f1 (ppm)

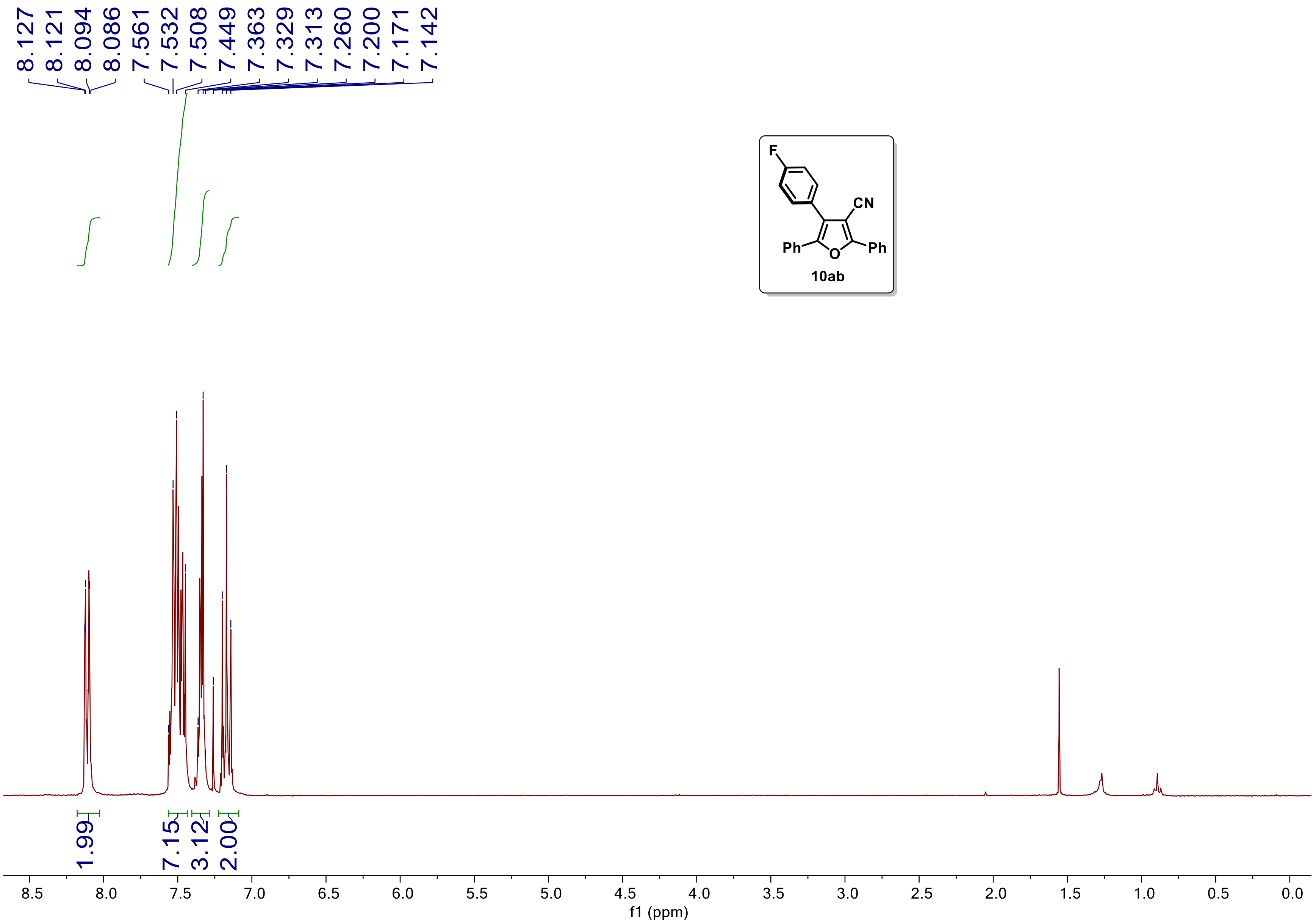
8.127
8.121
8.094
8.086
7.561
7.532
7.508
7.449
7.363
7.329
7.313
7.260
7.200
7.171
7.142
1.99
7.15
3.12
2.00
8.5
8.0
7.5
7.0
6.5
6.0
5.5
5.0
4.5
4.0
3.5
3.0
2.5
2.0
1.5
1.0
0.5
0.0
f1 (ppm)
F
CN
Ph
O
Ph

10ab

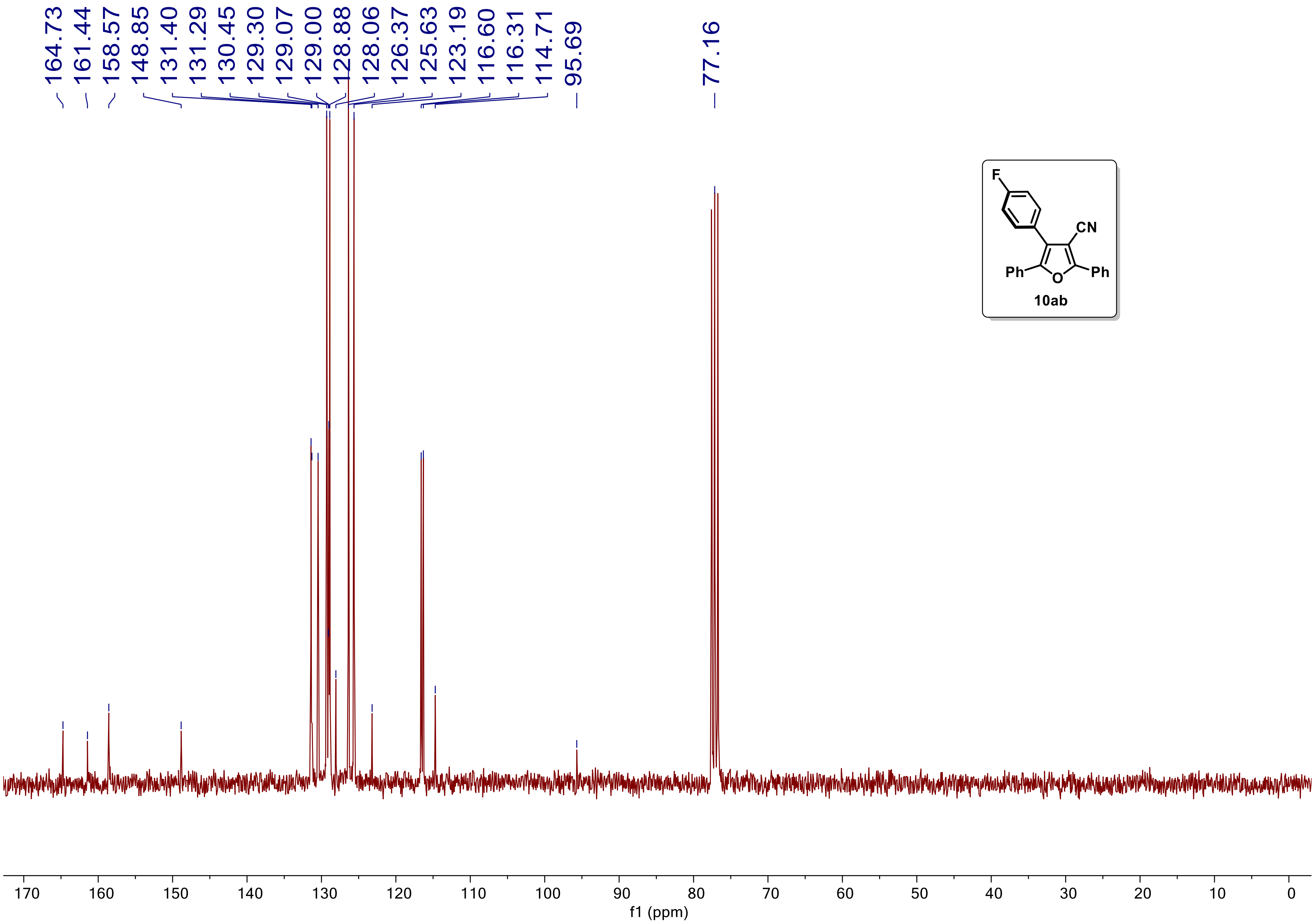
164.73
161.44
158.57
148.85
131.40
131.29
130.45
129.30
129.07
129.00
128.88
128.06
126.37
125.63
123.19
116.60
116.31
114.71
95.69
77.16
170
160
150
140
130
120
110
100
90
80
70
60
50
40
30
20
10
0
f1 (ppm)
F
CN
Ph
O
Ph

10ab

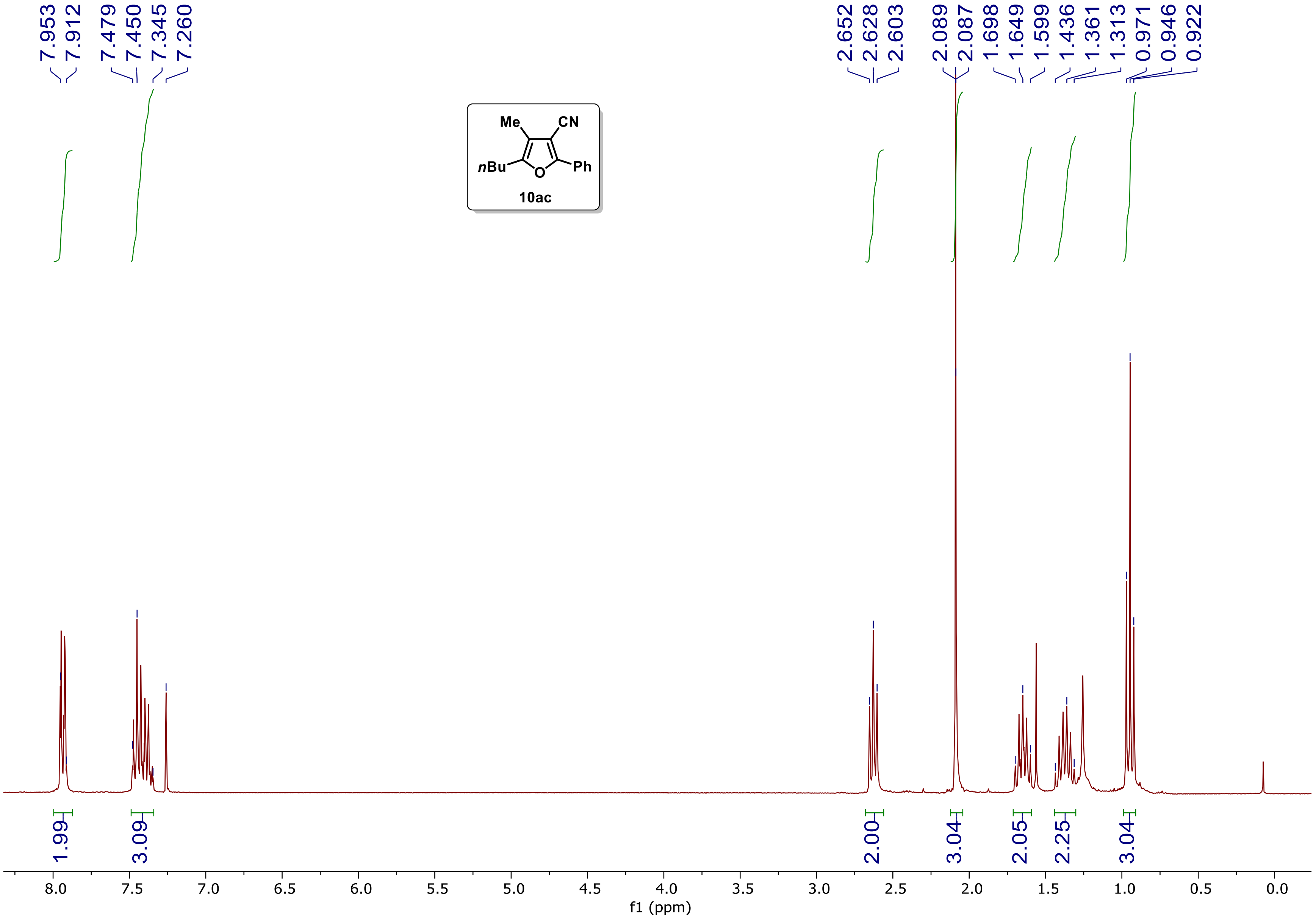

7.953
7.912
7.479
7.450
7.345
7.260
2.652
2.628
2.603
2.089
2.087
1.698
1.649
1.599
1.436
1.361
1.313
0.971
0.946
0.922
Me
CN
nBu
O
Ph
10ac
1.99
3.09
2.00
3.04
2.05
2.25
3.04
8.0
7.5
7.0
6.5
6.0
5.5
5.0
4.5
4.0
3.5
3.0
2.5
2.0
1.5
1.0
0.5
0.0
f1 (ppm)

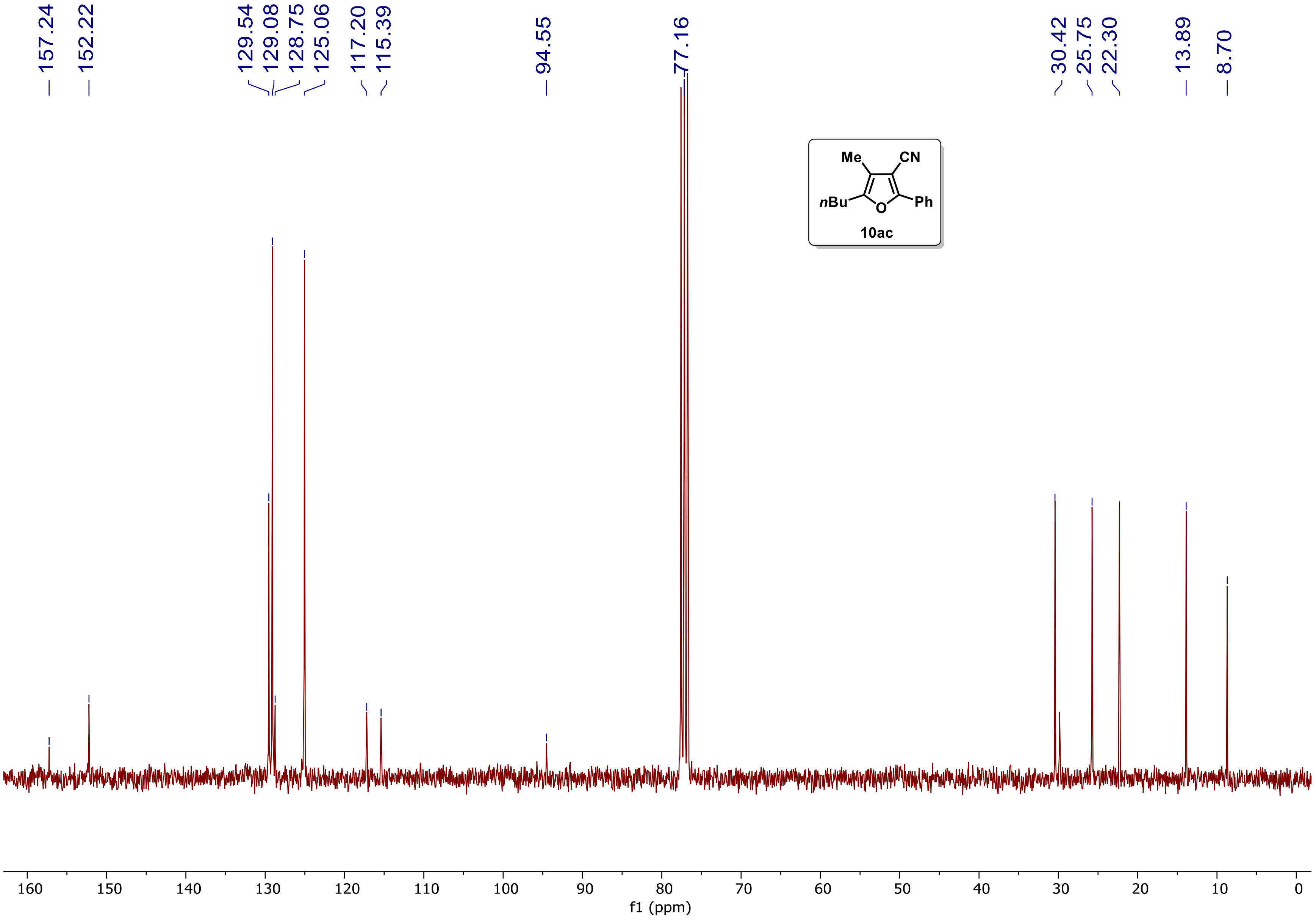
157.24
152.22
129.54
129.08
128.75
125.06
117.20
115.39
94.55
77.16
30.42
25.75
22.30
13.89
8.70
Me
CN
nBu
O
Ph
10ac
160
150
140
130
120
110
100
90
80
70
60
50
40
30
20
10
0
f1 (ppm)

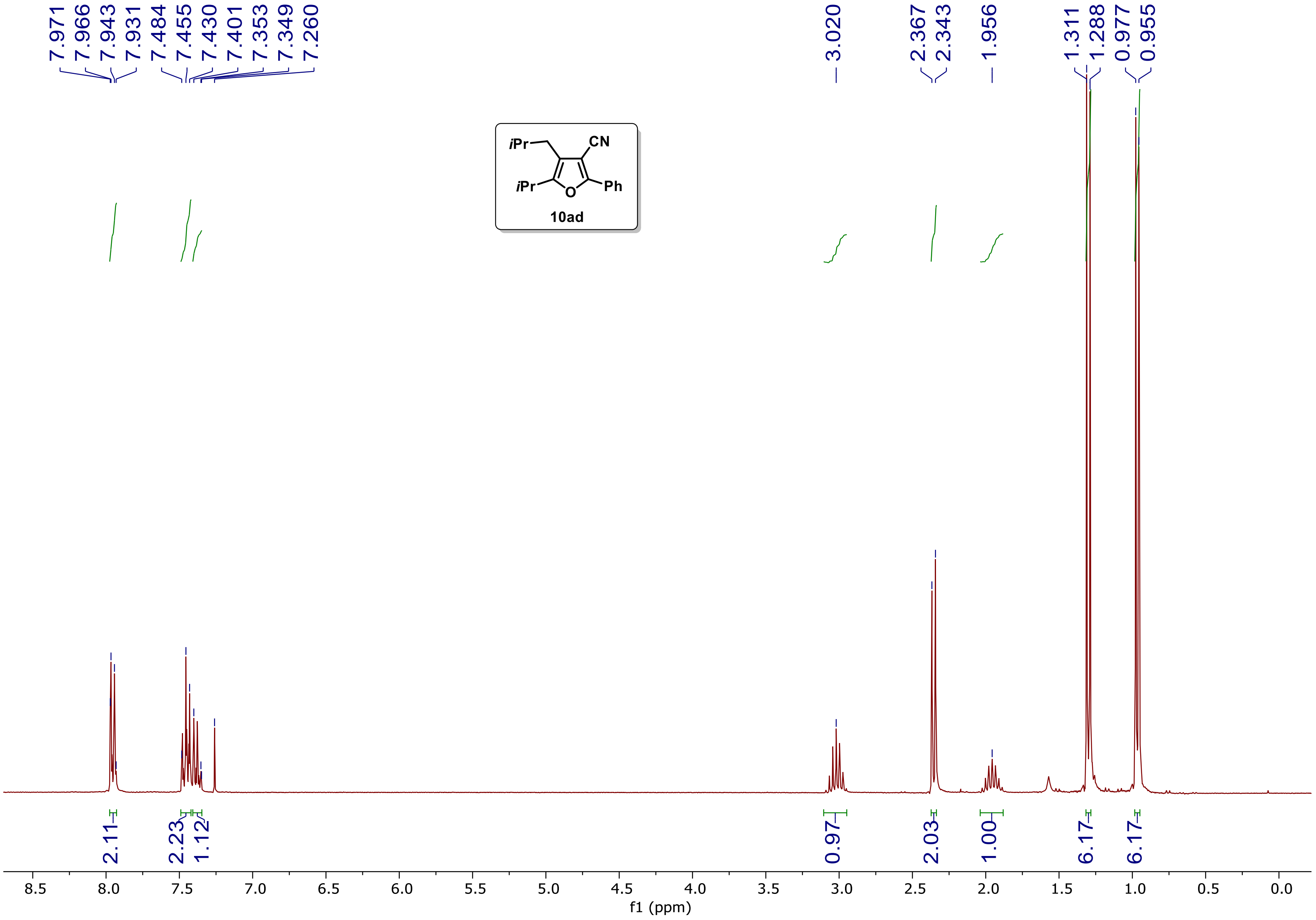

7.971
7.966
7.943
7.931
7.484
7.455
7.430
7.401
7.353
7.349
7.260
3.020
2.367
2.343
1.956
1.311
1.288
0.977
0.955
iPr
CN
iPr
Ph
O
10ad
2.11
2.23
1.12
0.97
2.03
1.00
6.17
6.17
8.5
8.0
7.5
7.0
6.5
6.0
5.5
5.0
4.5
4.0
3.5
3.0
2.5
2.0
1.5
1.0
0.5
0.0
f1 (ppm)

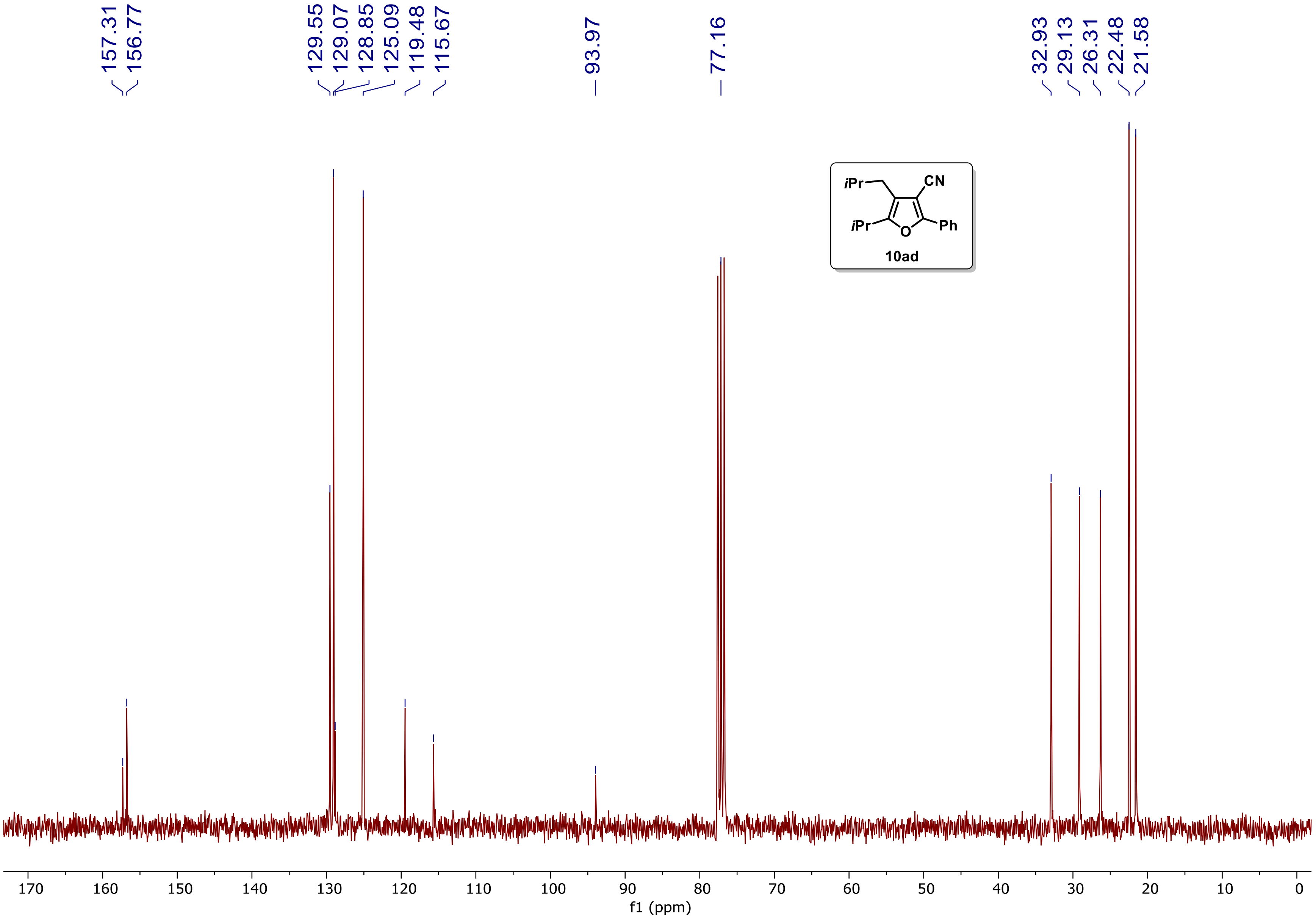
157.31
156.77
129.55
129.07
128.85
125.09
119.48
115.67
93.97
77.16
32.93
29.13
26.31
22.48
21.58
iPr
CN
iPr
O
Ph
10ad
170
160
150
140
130
120
110
100
90
80
70
60
50
40
30
20
10
0
f1 (ppm)

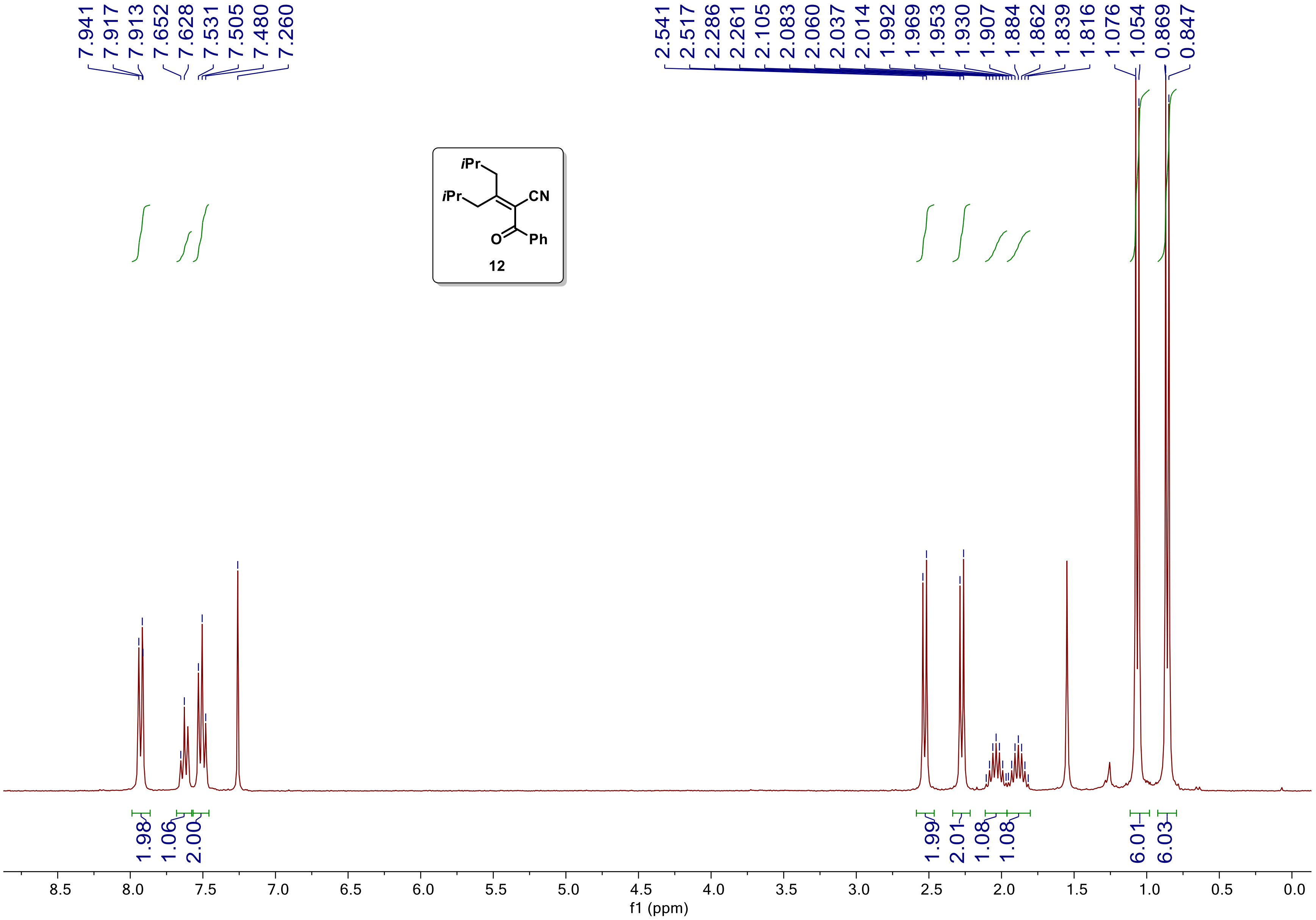

7.941
7.917
7.913
7.652
7.628
7.531
7.505
7.480
7.260
2.541
2.517
2.286
2.261
2.105
2.083
2.060
2.037
2.014
1.992
1.969
1.953
1.930
1.907
1.884
1.862
1.839
1.816
1.076
1.054
0.869
0.847
iPr
iPr
CN
O
Ph
12
1.98
1.06
2.00
1.99
2.01
1.08
1.08
6.01
6.03
8.5
8.0
7.5
7.0
6.5
6.0
5.5
5.0
4.5
4.0
3.5
3.0
2.5
2.0
1.5
1.0
0.5
0.0
f1 (ppm)

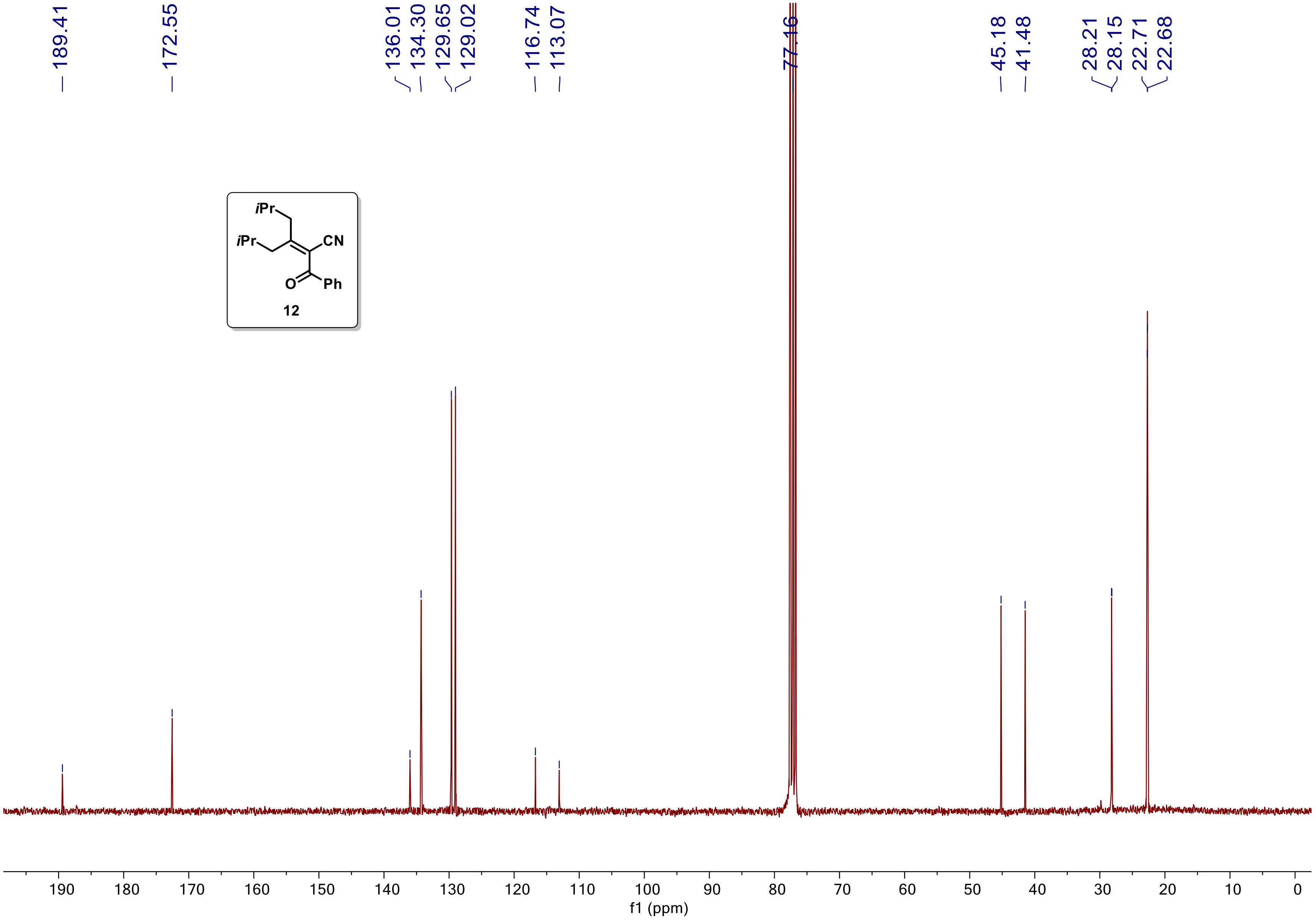
189.41
172.55
136.01
134.30
129.65
129.02
116.74
113.07
77.16
45.18
41.48
28.21
28.15
22.71
22.68
iPr
iPr
CN
O
Ph
12
190 180 170 160 150 140 130 120 110 100 90 80 70 60 50 40 30 20 10 0
f1 (ppm)

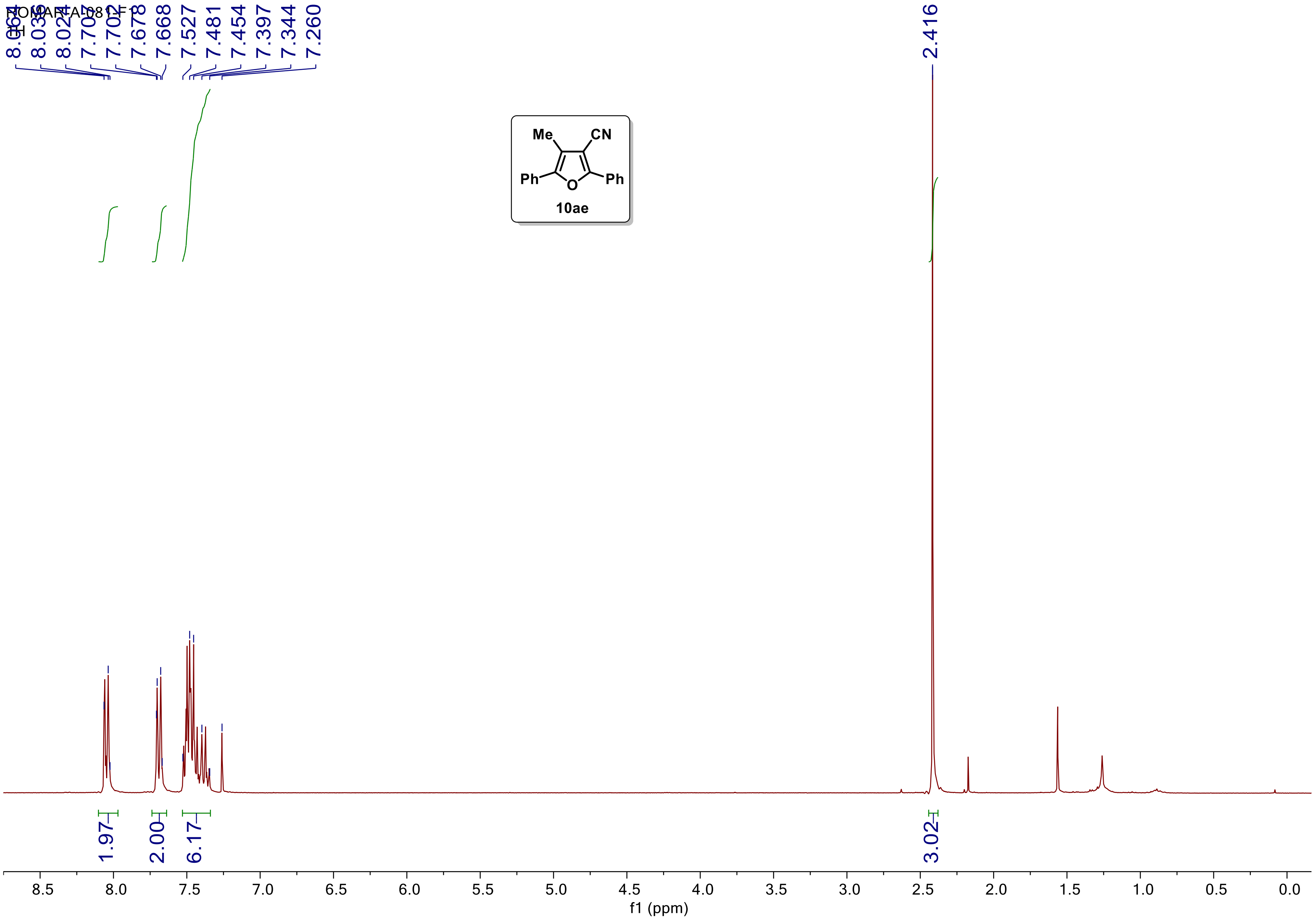
7.678
7.668
7.527
7.481
7.454
7.397
7.344
7.260
2.416
Me
CN
Ph
O
Ph
10ae
1.97
2.00
6.17
3.02
8.5
8.0
7.5
7.0
6.5
6.0
5.5
5.0
4.5
4.0
3.5
3.0
2.5
2.0
1.5
1.0
0.5
0.0
f1 (ppm)

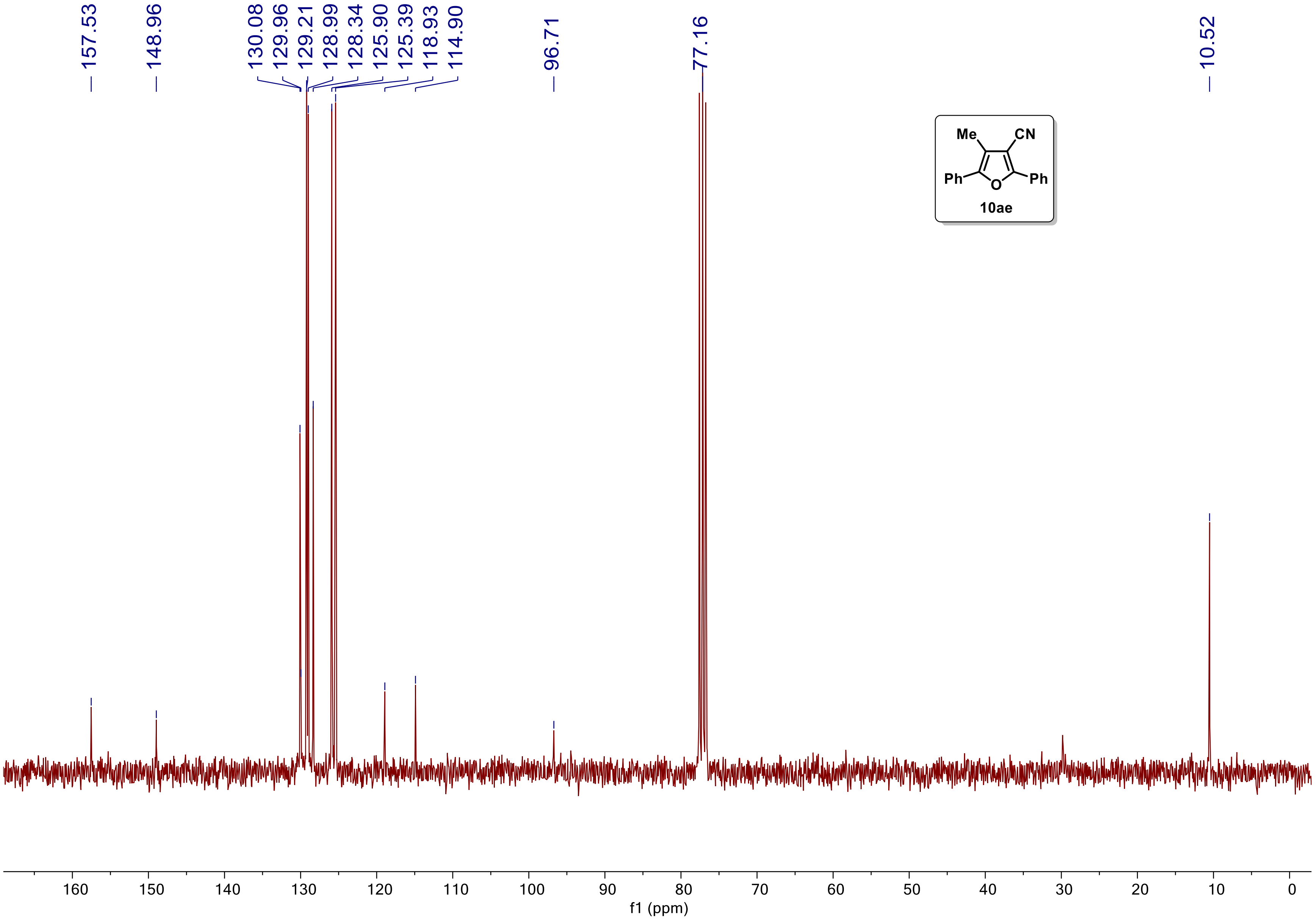
157.53
148.96
130.08
129.96
129.21
128.99
128.34
125.90
125.39
118.93
114.90
96.71
77.16
10.52
Me
CN
Ph
O
Ph
10ae
160
150
140
130
120
110
100
90
80
70
60
50
40
30
20
10
0
f1 (ppm)

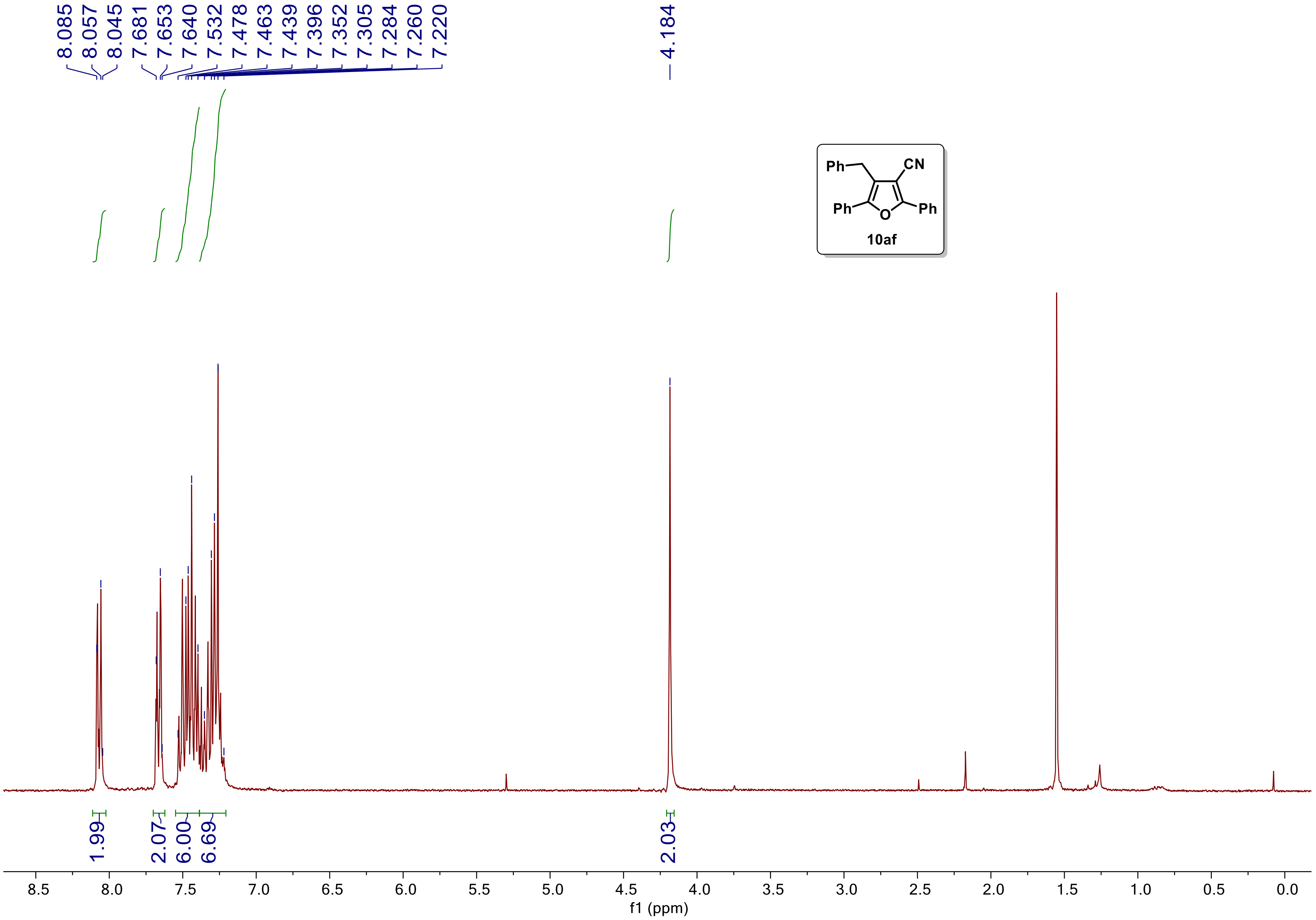

8.085
8.057
8.045
7.681
7.653
7.640
7.532
7.478
7.463
7.439
7.396
7.352
7.305
7.284
7.260
7.220
4.184
1.99
2.07
6.00
6.69
2.03
8.5
8.0
7.5
7.0
6.5
6.0
5.5
5.0
4.5
4.0
3.5
3.0
2.5
2.0
1.5
1.0
0.5
0.0
f1 (ppm)
Ph
CN
Ph
O
Ph

10af

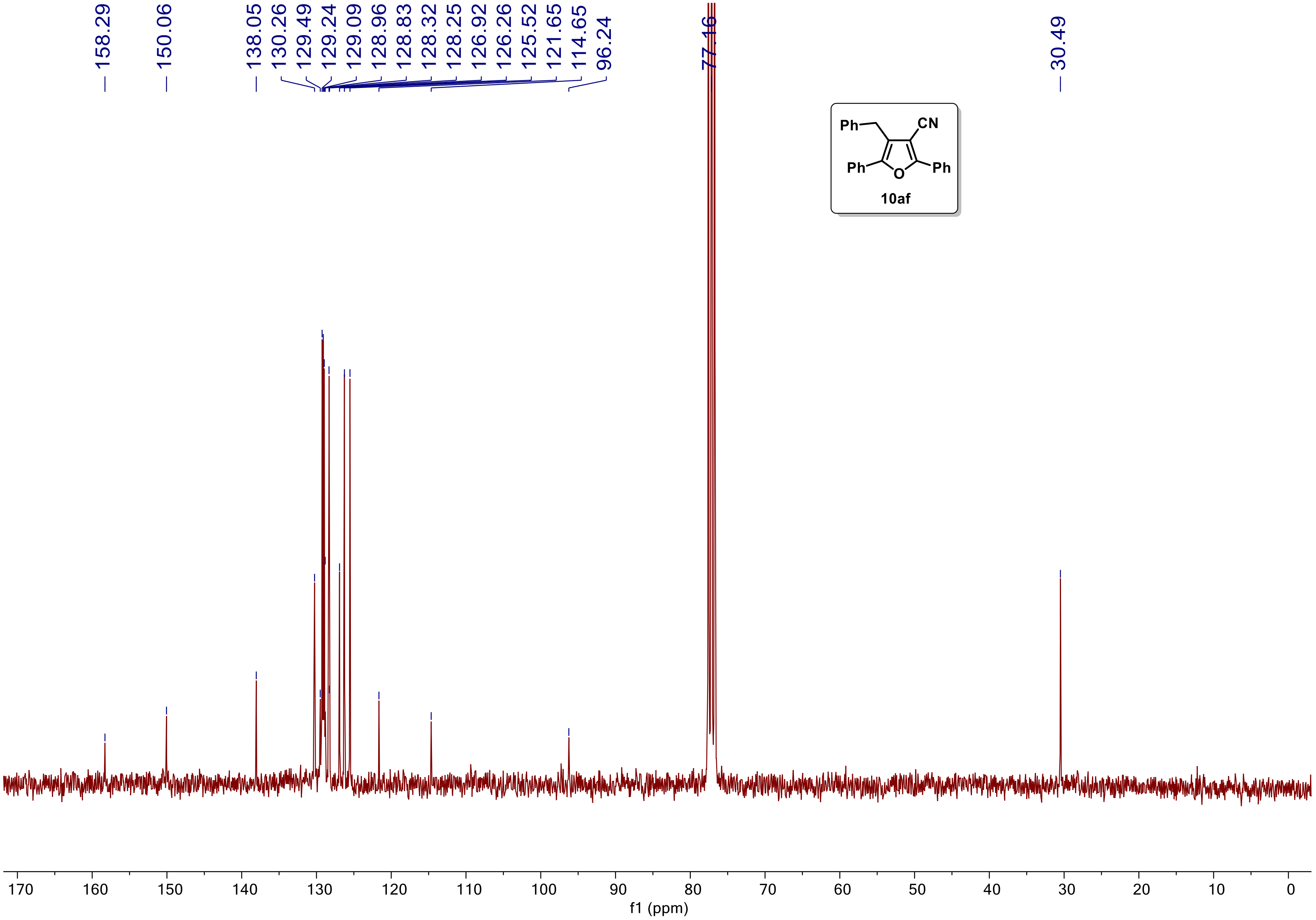
158.29
150.06
138.05
130.26
129.49
129.24
129.09
128.96
128.83
128.32
128.25
126.92
126.26
125.52
121.65
114.65
96.24
77.16
30.49
Ph
CN
Ph
O
Ph
Ph
10af
170
160
150
140
130
120
110
100
90
80
70
60
50
40
30
20
10
0
f1 (ppm)

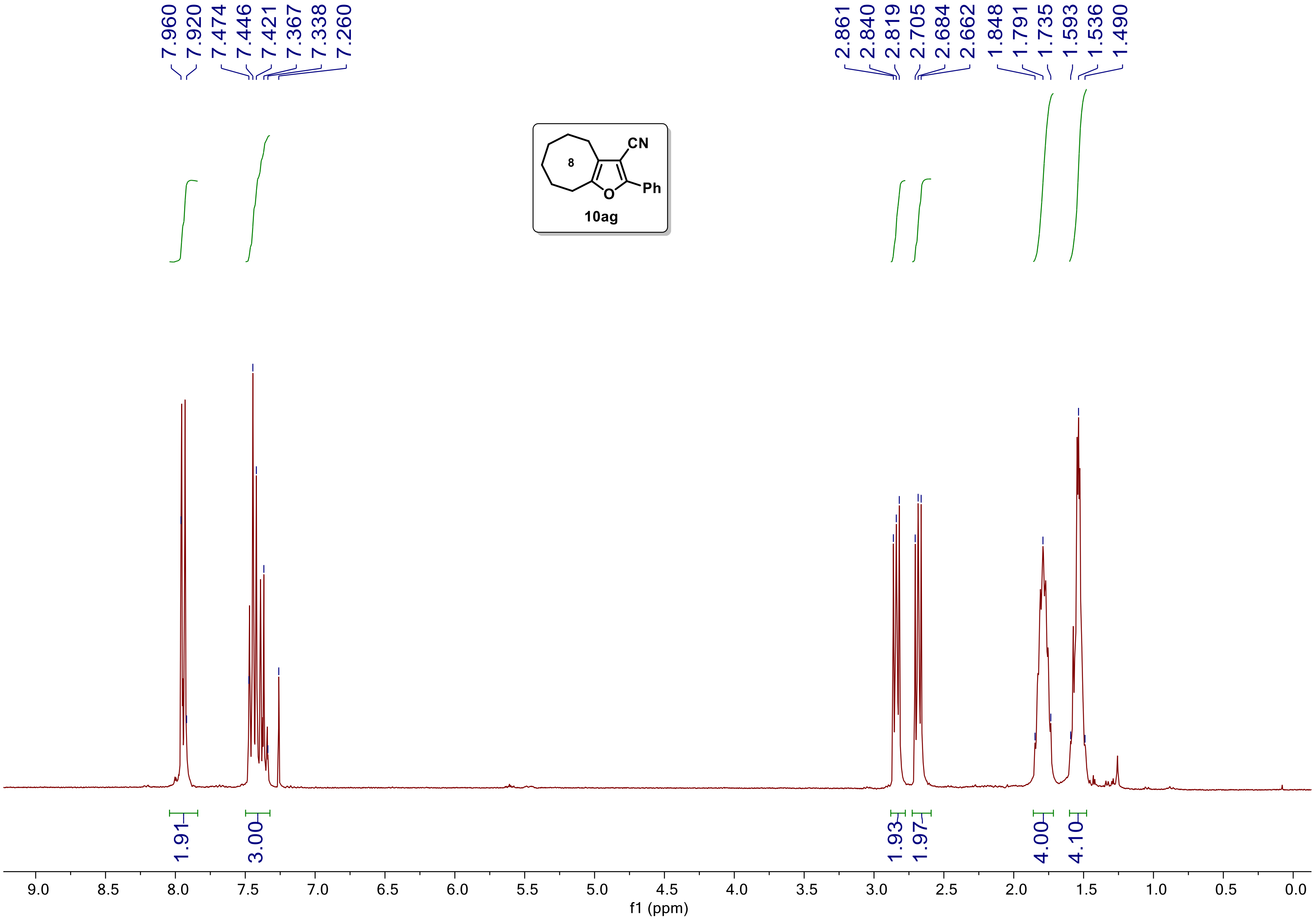
7.960
7.920
7.474
7.446
7.421
7.367
7.338
7.260
2.861
2.840
2.819
2.705
2.684
2.662
1.848
1.791
1.735
1.593
1.536
1.490
CN
8
O
Ph
10ag
1.91
3.00
1.93
1.97
4.00
4.10
9.0
8.5
8.0
7.5
7.0
6.5
6.0
5.5
5.0
4.5
4.0
3.5
3.0
2.5
2.0
1.5
1.0
0.5
0.0
f1 (ppm)

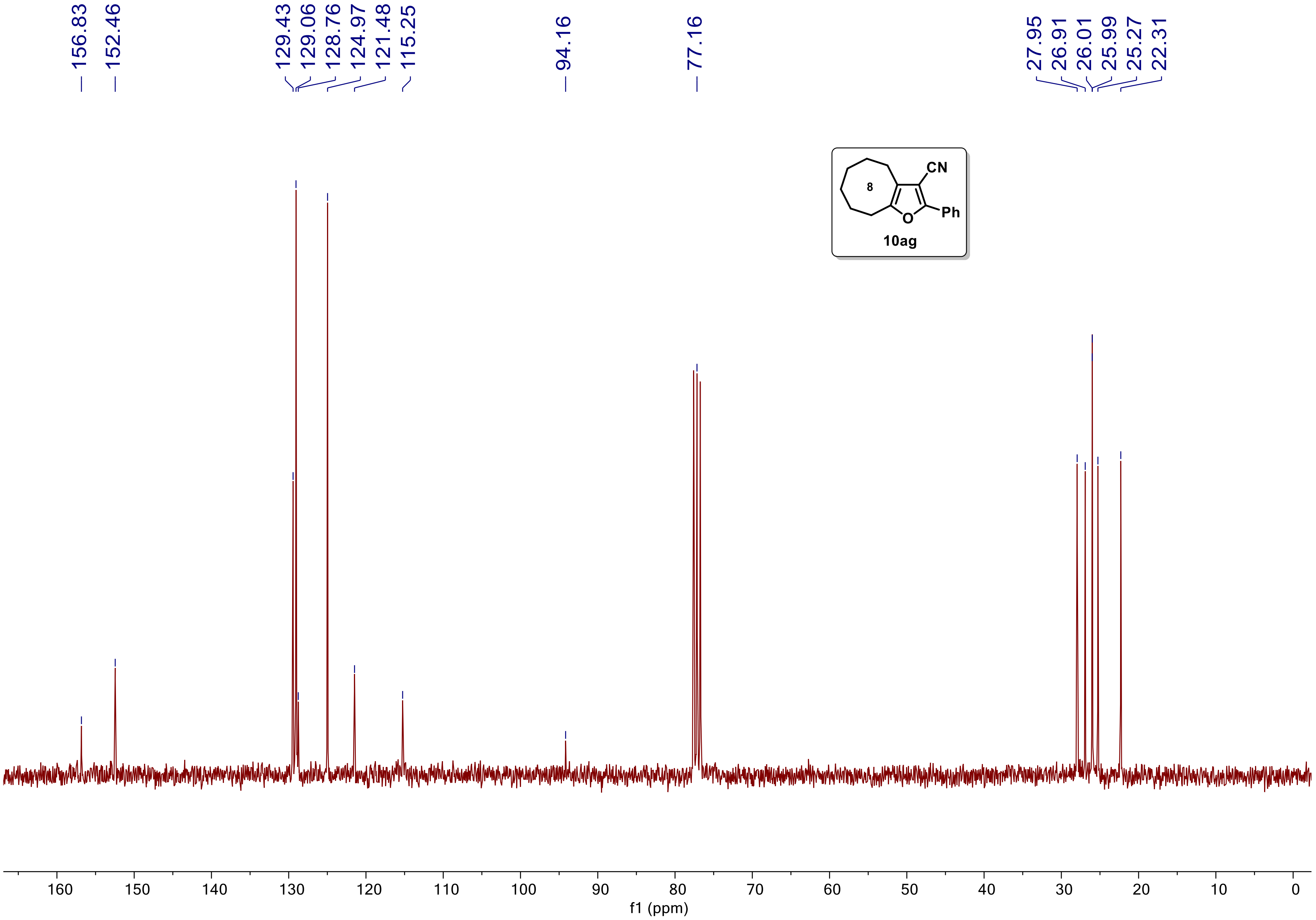
156.83
152.46
129.43
129.06
128.76
124.97
121.48
115.25
94.16
77.16
27.95
26.91
26.01
25.99
25.27
22.31
CN
8
O
Ph
10ag
160 150 140 130 120 110 100 90 80 70 60 50 40 30 20 10 0
f1 (ppm)

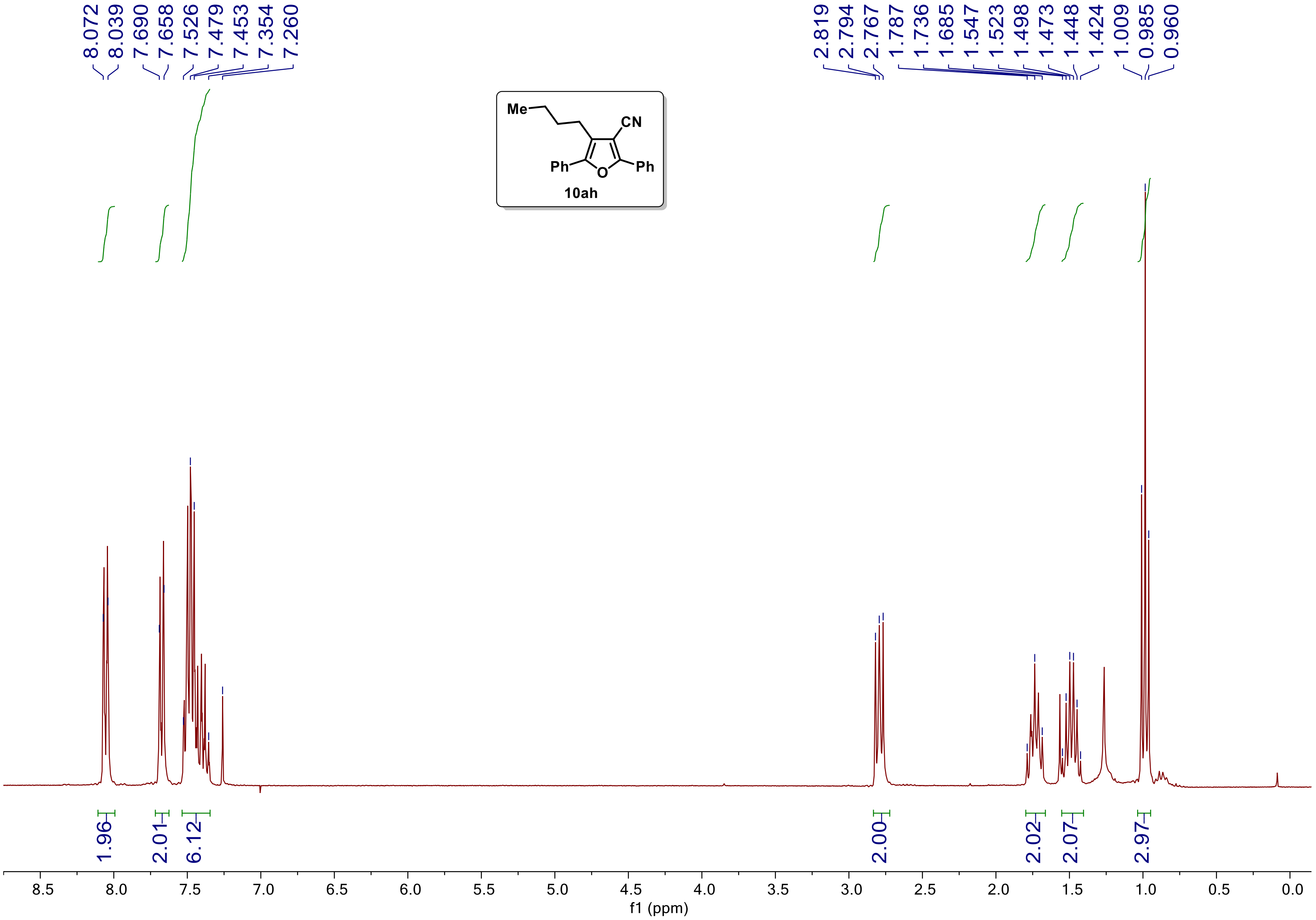
8.072
8.039
7.690
7.658
7.526
7.479
7.453
7.354
7.260
2.819
2.794
2.767
1.787
1.736
1.685
1.547
1.523
1.498
1.473
1.448
1.424
1.009
0.985
0.960
Me
CN
Ph
O
Ph
10ah
1.96
2.01
6.12
2.00
2.02
2.07
2.97
8.5
8.0
7.5
7.0
6.5
6.0
5.5
5.0
4.5
4.0
3.5
3.0
2.5
2.0
1.5
1.0
0.5
0.0
f1 (ppm)

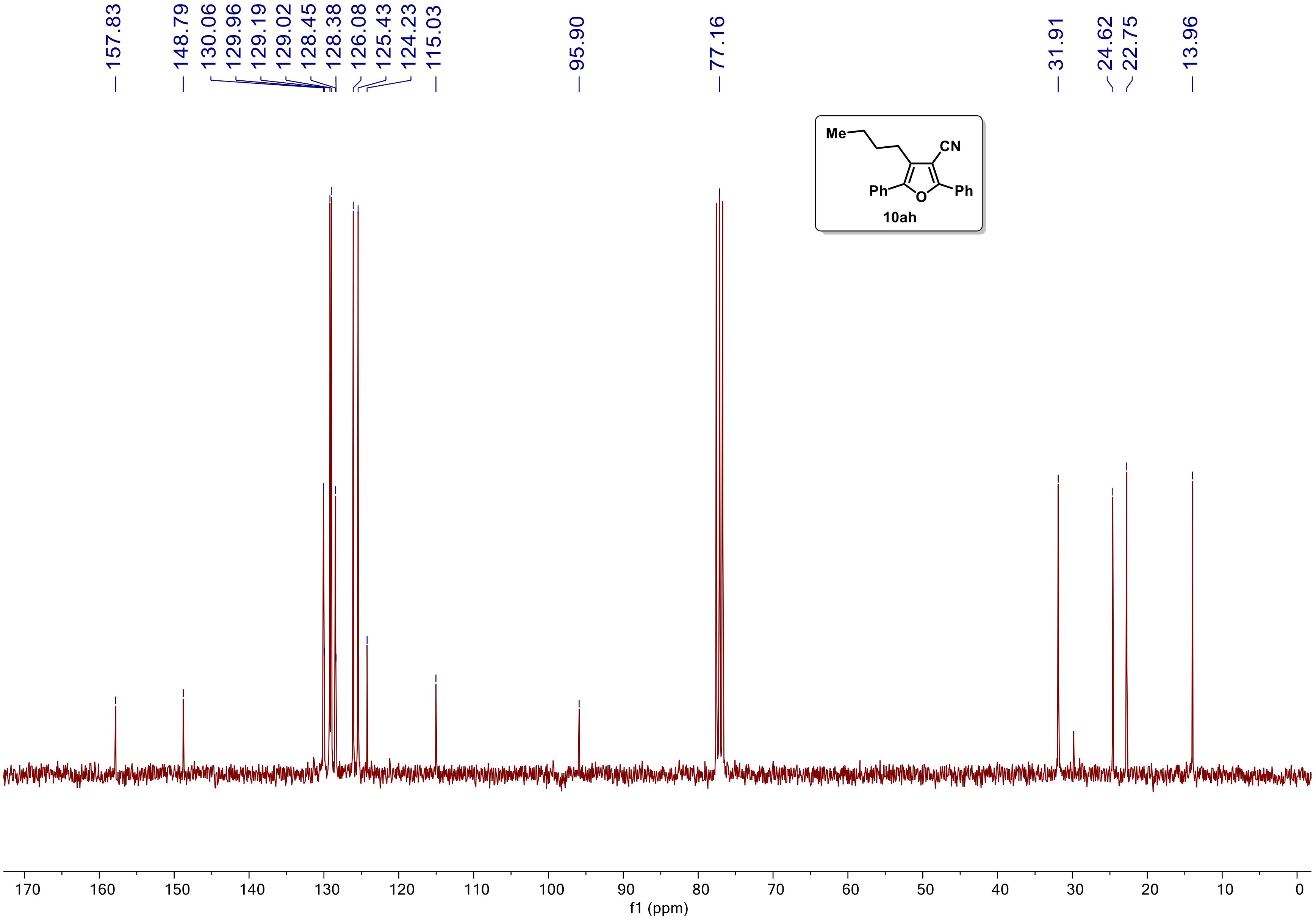
157.83
148.79
130.06
129.96
129.19
129.02
128.45
128.38
126.08
125.43
124.23
115.03
95.90
77.16
31.91
24.62
22.75
13.96
Me
CN
Ph
O
Ph
10ah
170
160
150
140
130
120
110
100
90
80
70
60
50
40
30
20
10
0
f1 (ppm)

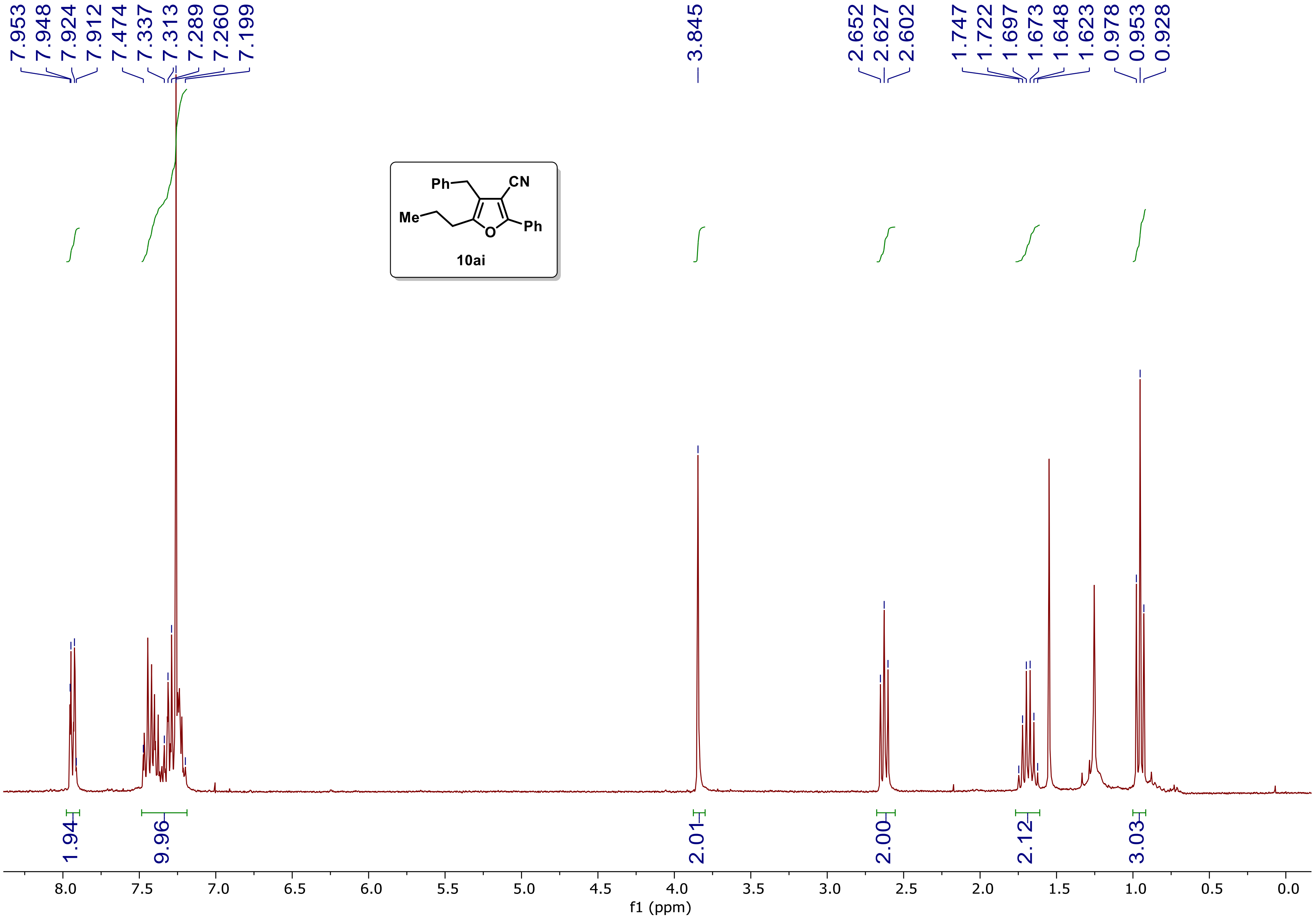
7.953
7.948
7.924
7.912
7.474
7.337
7.313
7.289
7.260
7.199
3.845
2.652
2.627
2.602
1.747
1.722
1.697
1.673
1.648
1.623
0.978
0.953
0.928
Ph
CN
Me
O
Ph
10ai
1.94
9.96
2.01
2.00
2.12
3.03
8.0
7.5
7.0
6.5
6.0
5.5
5.0
4.5
4.0
3.5
3.0
2.5
2.0
1.5
1.0
0.5
0.0
f1 (ppm)

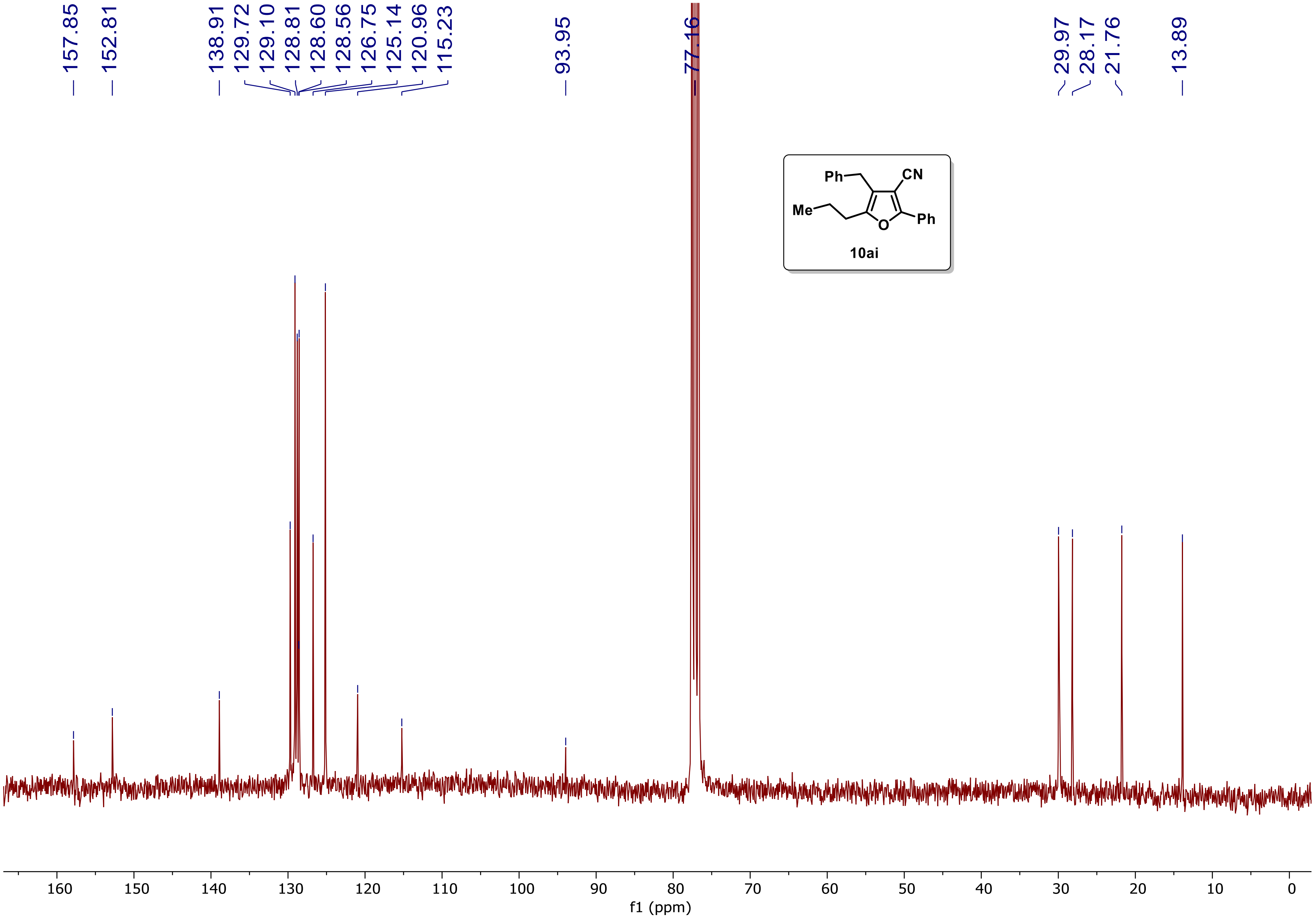
157.85
152.81
138.91
129.72
129.10
128.81
128.60
128.56
126.75
125.14
120.96
115.23
93.95
77.16
29.97
28.17
21.76
13.89
Ph
CN
Me
O
Ph
10ai
160
150
140
130
120
110
100
90
80
70
60
50
40
30
20
10
0
f1 (ppm)

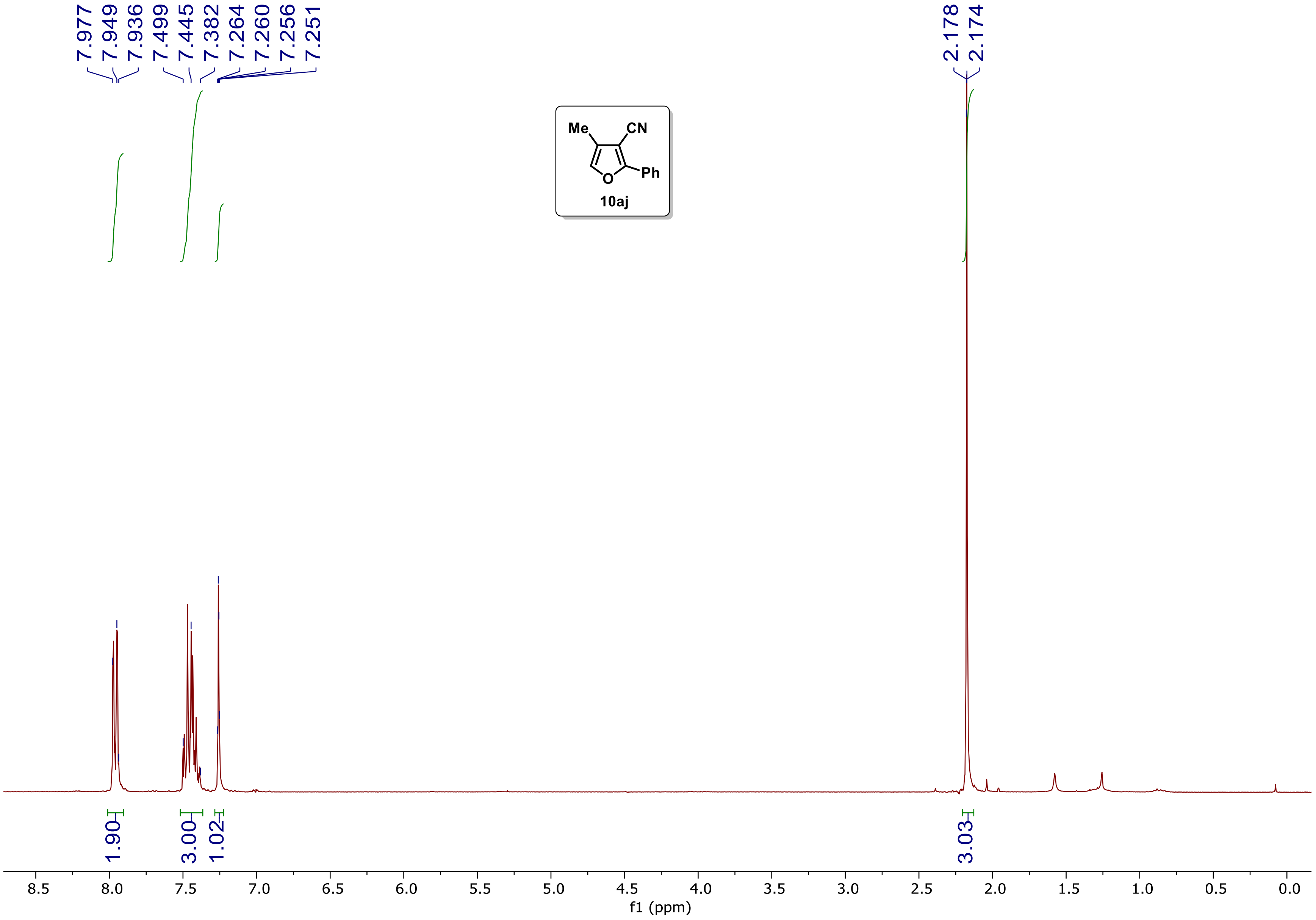

7.977
7.949
7.936
7.499
7.445
7.382
7.264
7.260
7.256
7.251
2.178
2.174
Me
CN
O
Ph
10aj
1.90
3.00
1.02
3.03
8.5 8.0 7.5 7.0 6.5 6.0 5.5 5.0 4.5 4.0 3.5 3.0 2.5 2.0 1.5 1.0 0.5 0.0
f1 (ppm)

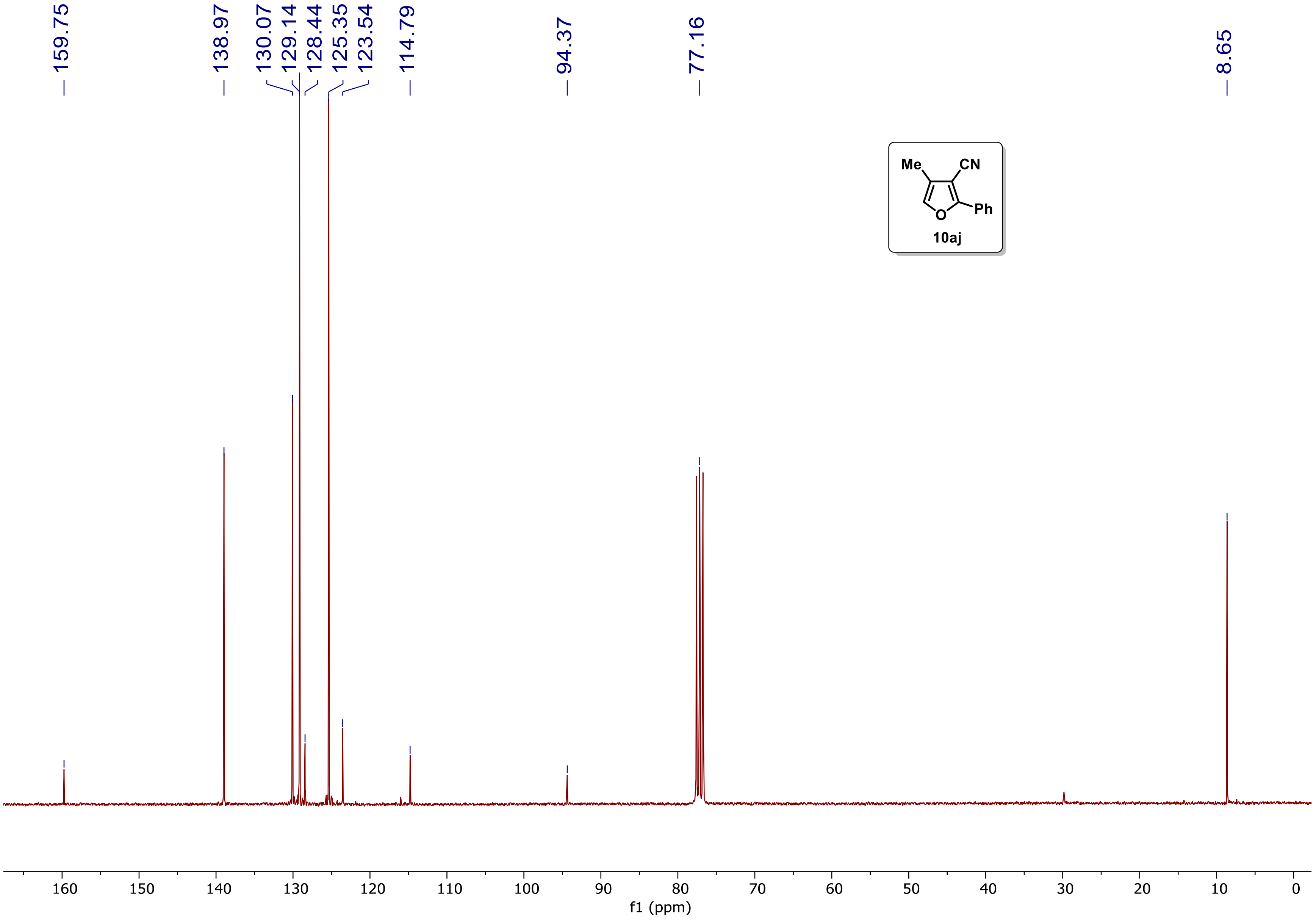
159.75
138.97
130.07
129.14
128.44
125.35
123.54
114.79
94.37
77.16
8.65
Me
CN
O
Ph
10aj
160
150
140
130
120
110
100
90
80
70
60
50
40
30
20
10
0
f1 (ppm)

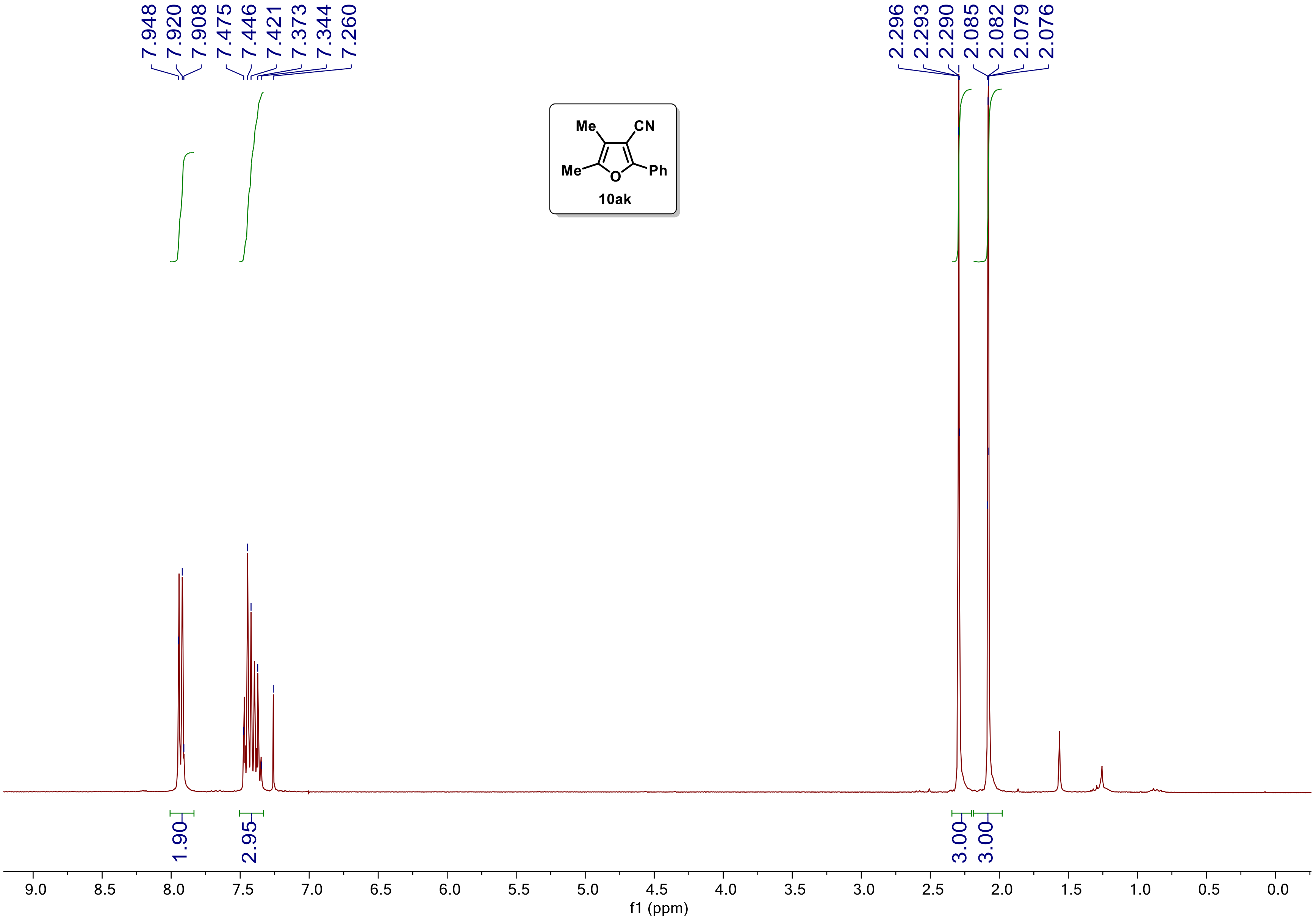
7.948
7.920
7.908
7.475
7.446
7.421
7.373
7.344
7.260
2.296
2.293
2.290
2.085
2.082
2.079
2.076
Me
CN
Me
O
Ph
10ak
1.90
2.95
3.00
3.00
9.0 8.5 8.0 7.5 7.0 6.5 6.0 5.5 5.0 4.5 4.0 3.5 3.0 2.5 2.0 1.5 1.0 0.5 0.0
f1 (ppm)

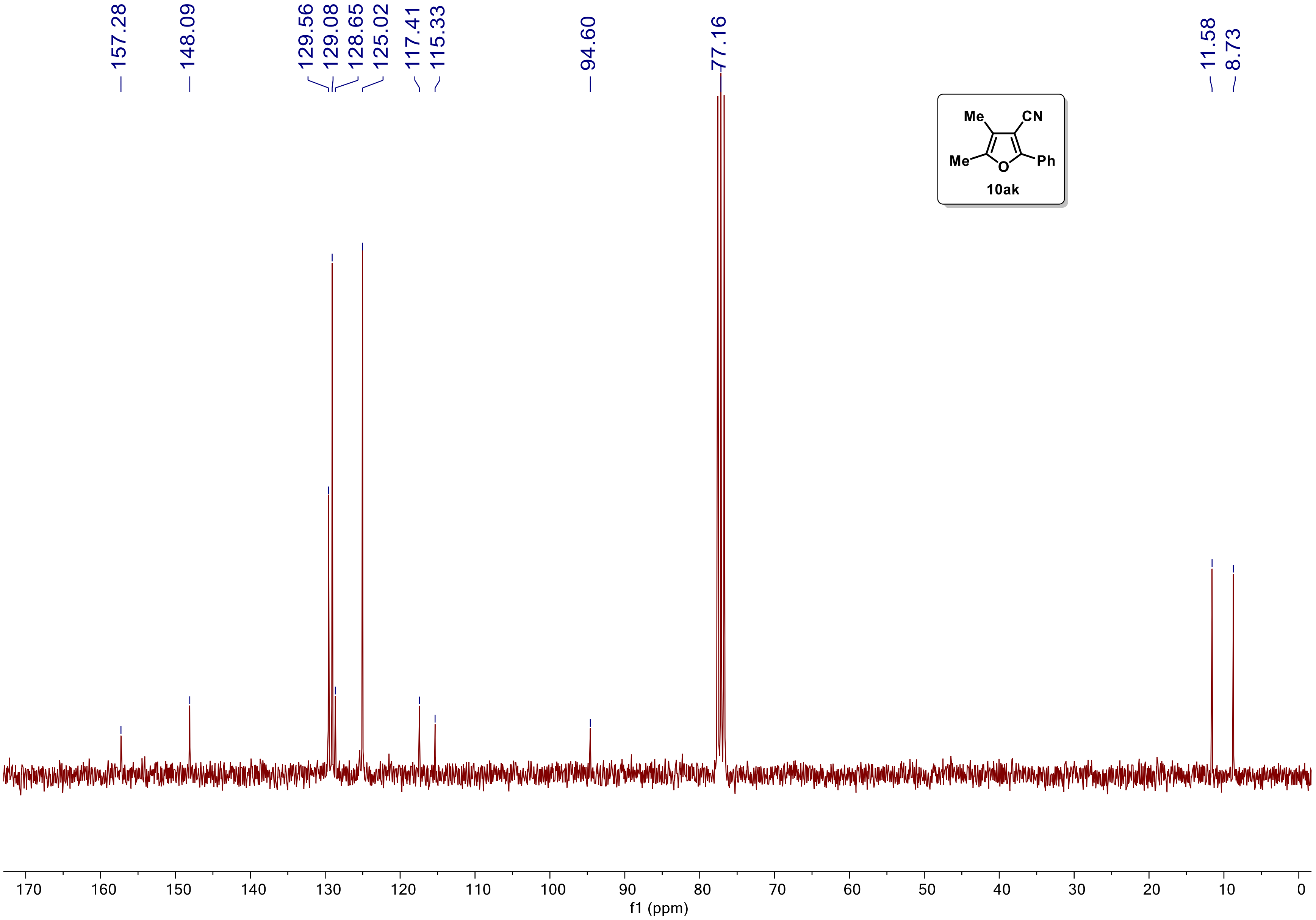
157.28
148.09
129.56
129.08
128.65
125.02
117.41
115.33
94.60
77.16
11.58
8.73
Me
CN
Me
O
Ph
10ak
170 160 150 140 130 120 110 100 90 80 70 60 50 40 30 20 10 0
f1 (ppm)

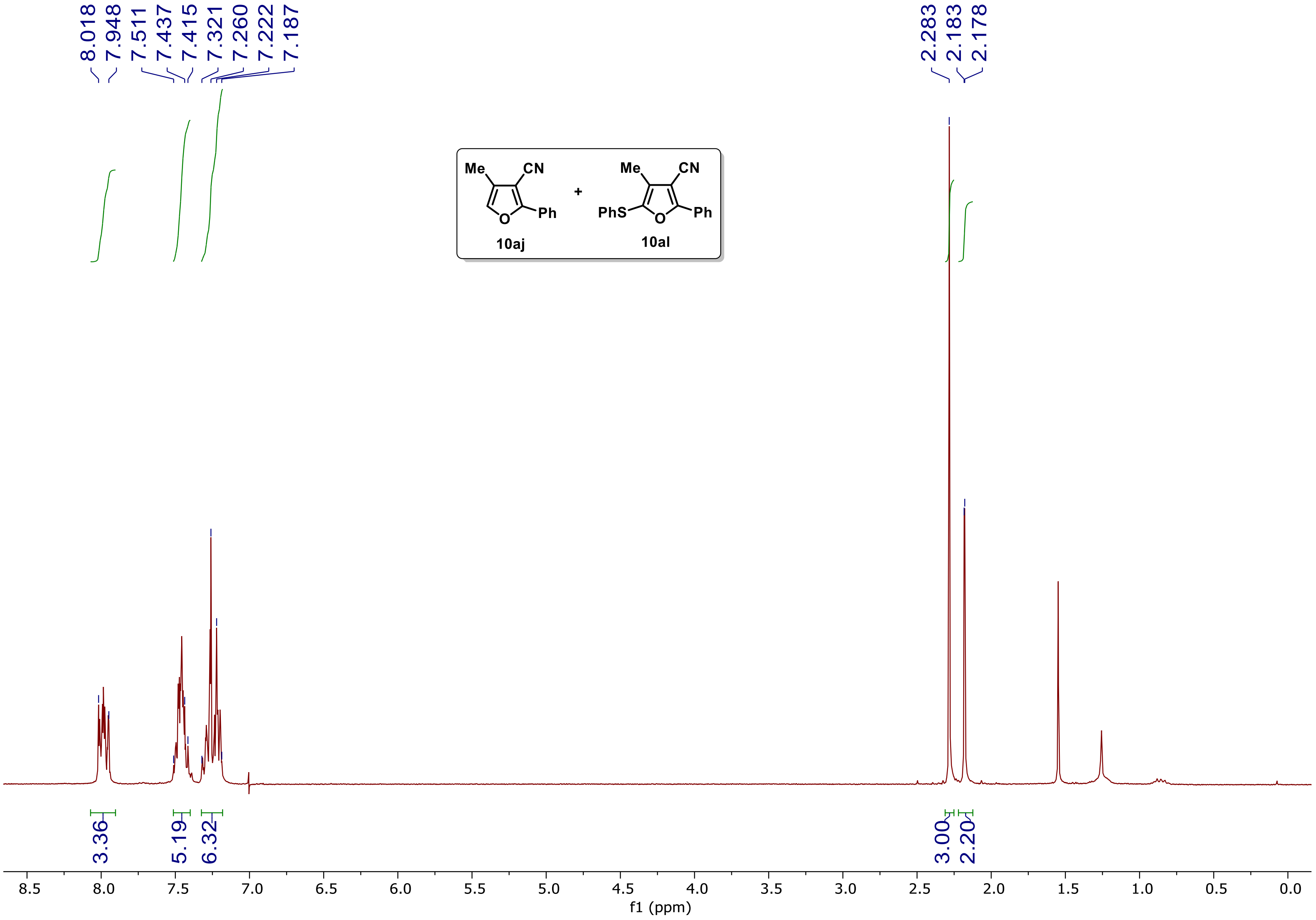

8.018
7.948
7.511
7.437
7.415
7.321
7.260
7.222
7.187
2.283
2.183
2.178
Me
CN
Ph
O
10aj
+
Me
CN
PhS
O
Ph
10al
3.36
5.19
6.32
3.00
2.20
8.5
8.0
7.5
7.0
6.5
6.0
5.5
5.0
4.5
4.0
3.5
3.0
2.5
2.0
1.5
1.0
0.5
0.0
f1 (ppm)

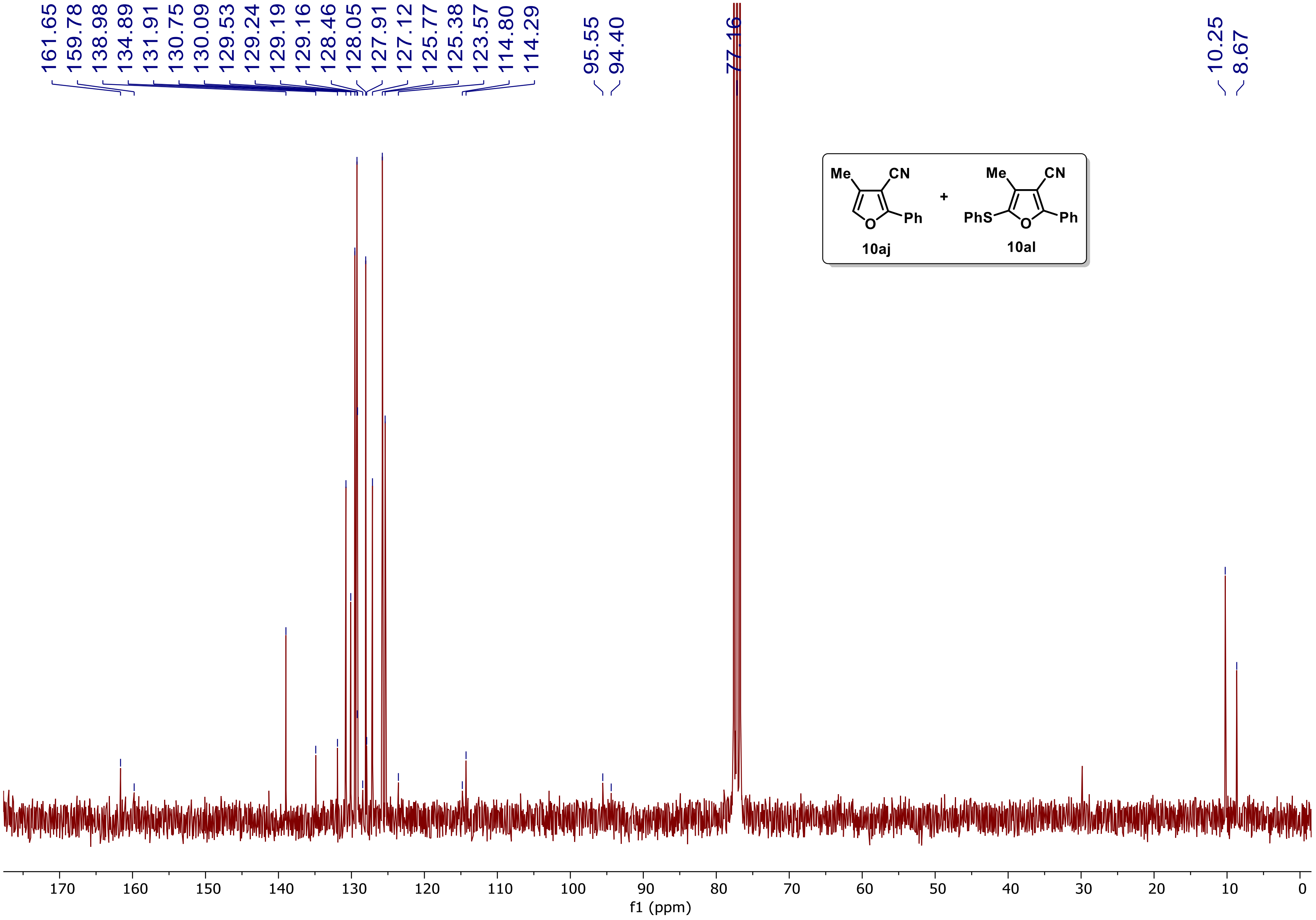
161.65
159.78
138.98
134.89
131.91
130.75
130.09
129.53
129.24
129.19
129.16
128.46
128.05
127.91
127.12
125.77
125.38
123.57
114.80
114.29
95.55
94.40
77.16
10.25
8.67
Me
CN
Ph
O
10aj
+
PhS
10al
f1 (ppm)

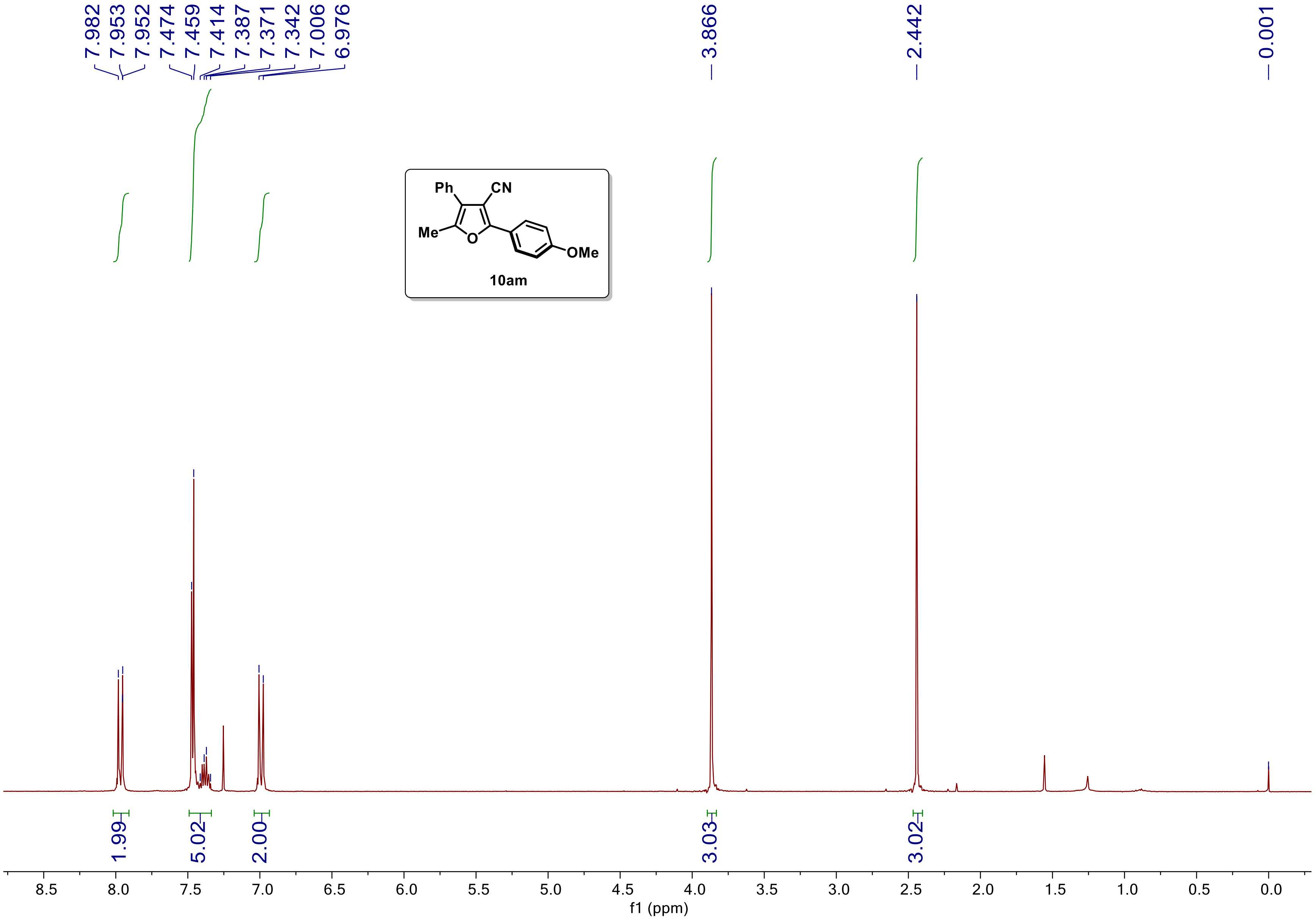
7.982
7.953
7.952
7.474
7.459
7.414
7.387
7.371
7.342
7.006
6.976
3.866
2.442
0.001
Ph
CN
Me
O
OMe
10am
1.99
5.02
2.00
3.03
3.02
8.5
8.0
7.5
7.0
6.5
6.0
5.5
5.0
4.5
4.0
3.5
3.0
2.5
2.0
1.5
1.0
0.5
0.0
f1 (ppm)

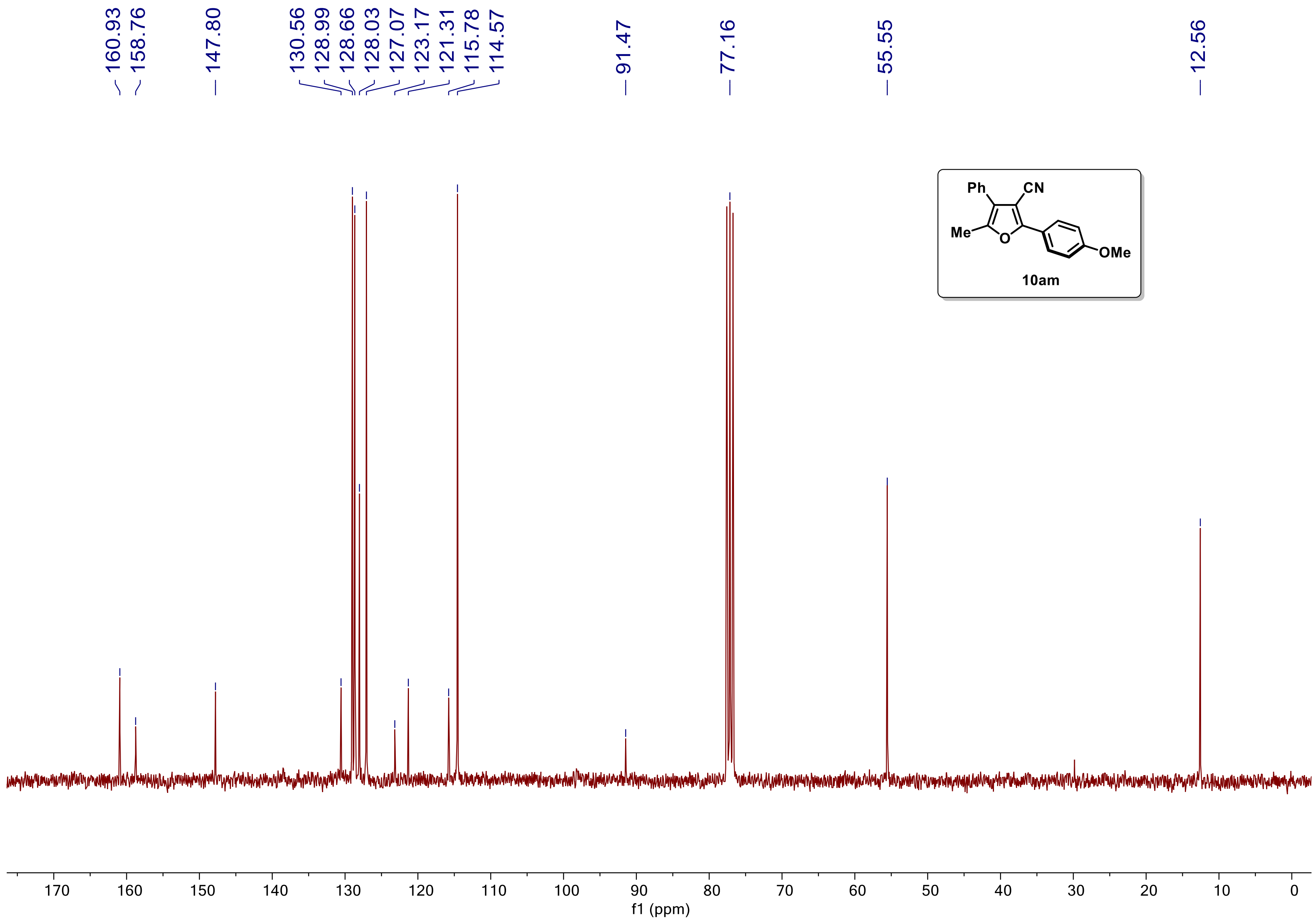
160.93
158.76
147.80
130.56
128.99
128.66
128.03
127.07
123.17
121.31
115.78
114.57
91.47
77.16
55.55
12.56
Ph
CN
Me
O
OMe
10am
170 160 150 140 130 120 110 100 90 80 70 60 50 40 30 20 10 0
f1 (ppm)

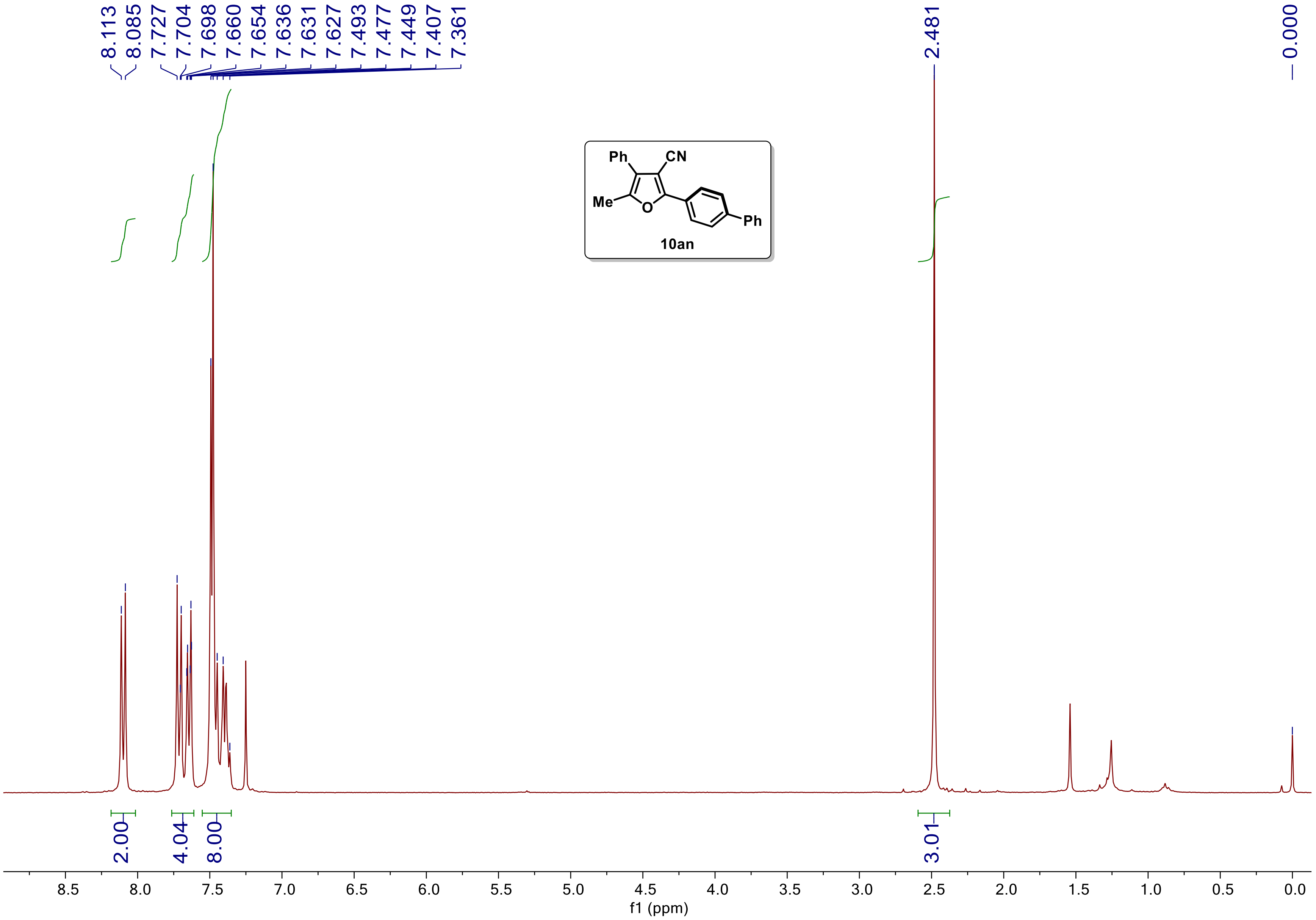
8.113
8.085
7.727
7.704
7.698
7.660
7.654
7.636
7.631
7.627
7.493
7.477
7.449
7.407
7.361
2.481
0.000
Ph
CN
Me
O
Ph
10an
2.00
4.04
8.00
3.01
8.5 8.0 7.5 7.0 6.5 6.0 5.5 5.0 4.5 4.0 3.5 3.0 2.5 2.0 1.5 1.0 0.5 0.0
f1 (ppm)

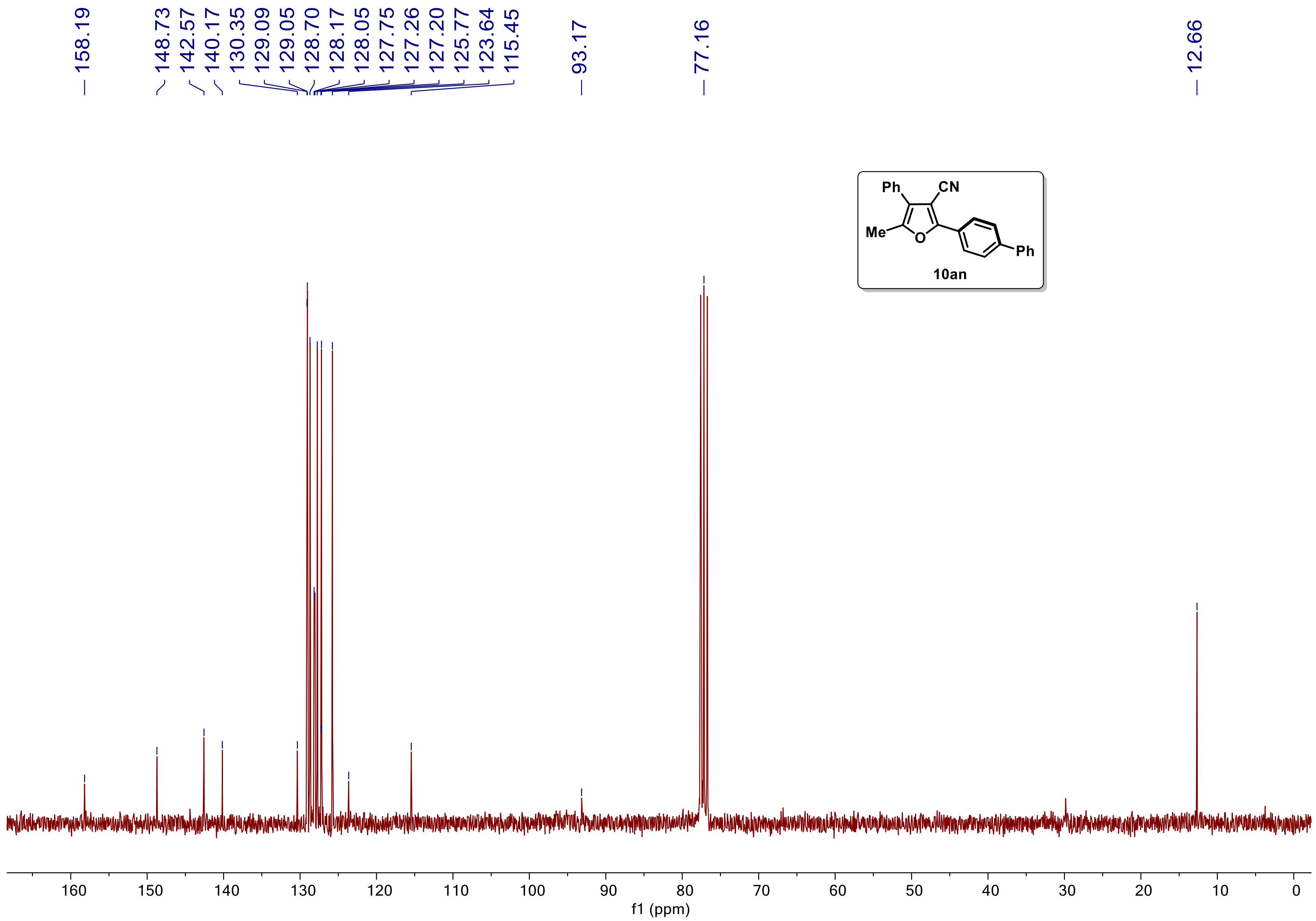

158.19
148.73
142.57
140.17
130.35
129.09
129.05
128.70
128.17
128.05
127.75
127.26
127.20
125.77
123.64
115.45
93.17
77.16
12.66
Ph
CN
Me
O
Ph
10an
160
150
140
130
120
110
100
90
80
70
60
50
40
30
20
10
0
f1 (ppm)

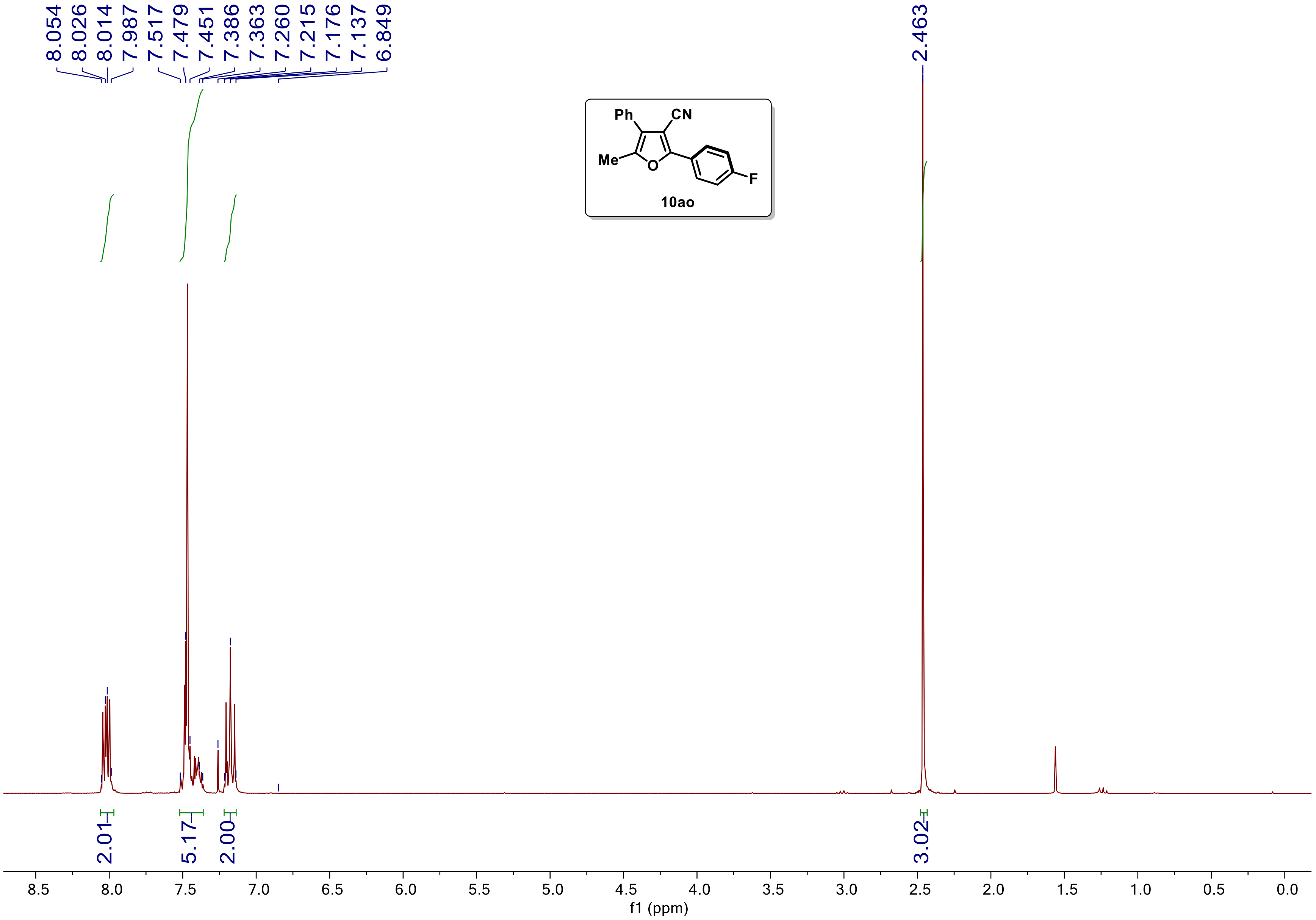

8.054
8.026
8.014
7.987
7.517
7.479
7.451
7.386
7.363
7.260
7.215
7.176
7.137
6.849
2.463
Ph
CN
Me
O
F
10ao
2.01
5.17
2.00
3.02
8.5
8.0
7.5
7.0
6.5
6.0
5.5
5.0
4.5
4.0
3.5
3.0
2.5
2.0
1.5
1.0
0.5
0.0
f1 (ppm)

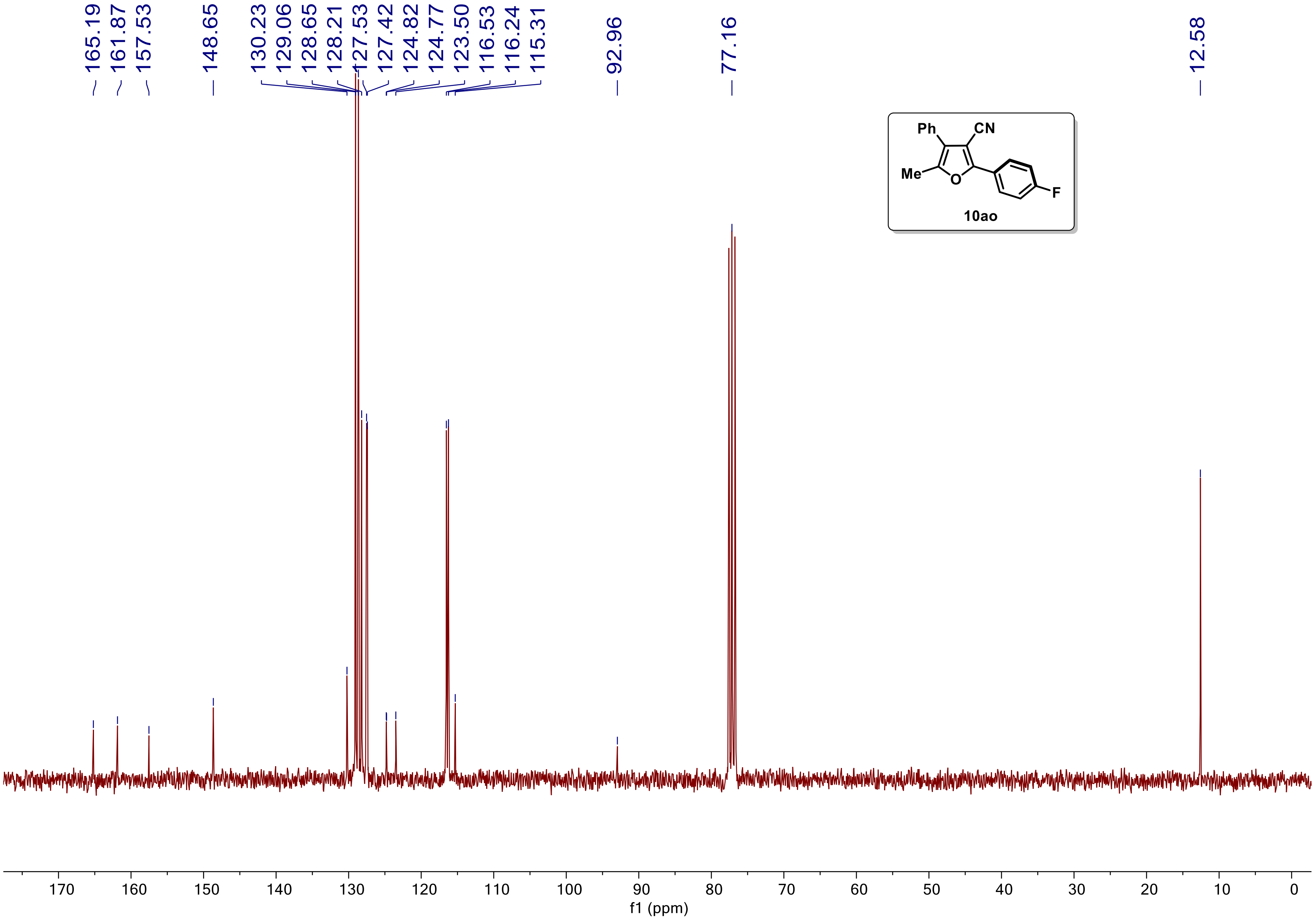
165.19
161.87
157.53
148.65
130.23
129.06
128.65
128.21
127.53
127.42
124.82
124.77
123.50
116.53
116.24
115.31
92.96
77.16
12.58
Ph
CN
Me
O
F
10ao
170 160 150 140 130 120 110 100 90 80 70 60 50 40 30 20 10 0
f1 (ppm)

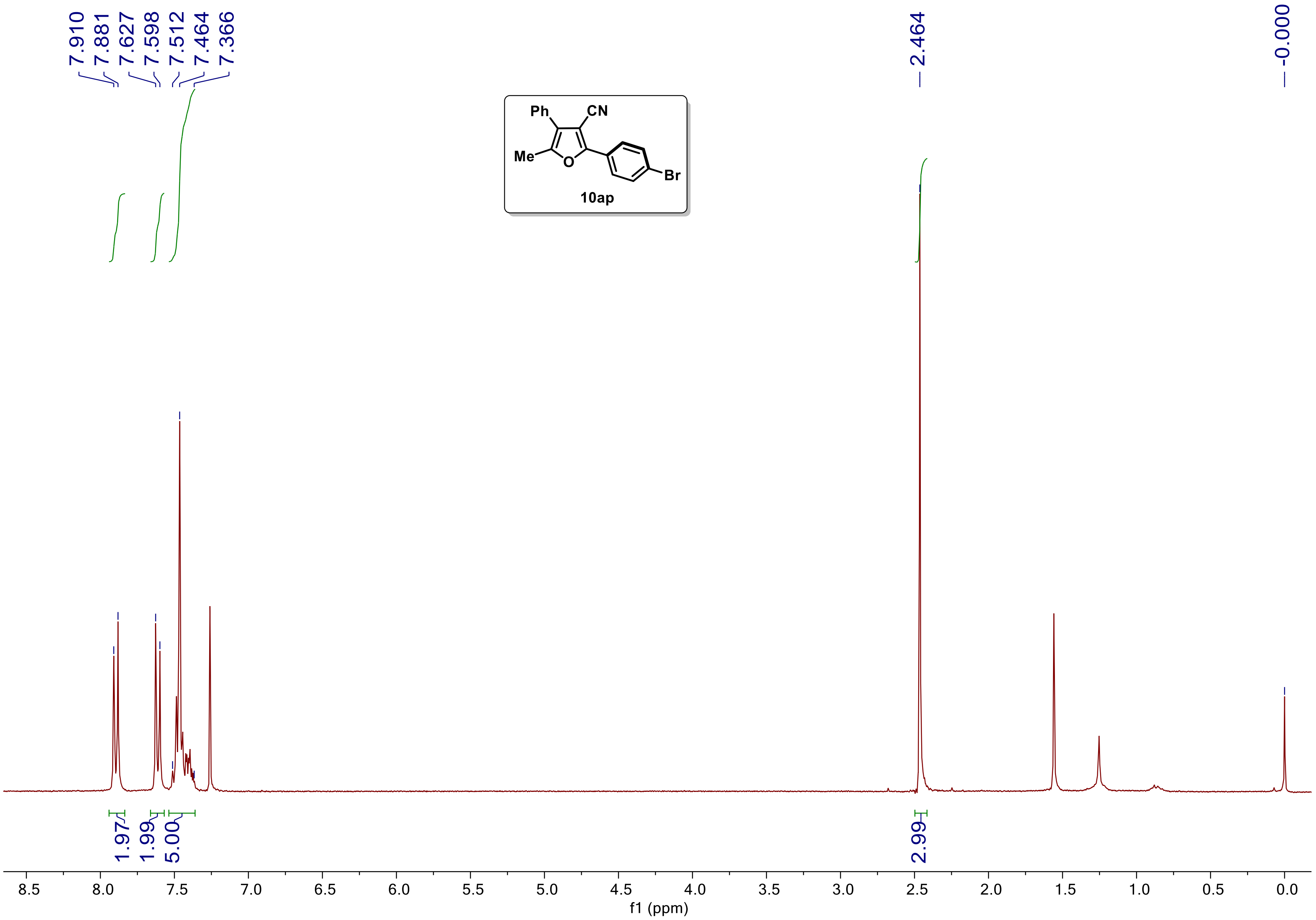

7.910
7.881
7.627
7.598
7.512
7.464
7.366
2.464
-0.000
Ph
CN
Me
O
Br
10ap
1.97
1.99
5.00
2.99
8.5
8.0
7.5
7.0
6.5
6.0
5.5
5.0
4.5
4.0
3.5
3.0
2.5
2.0
1.5
1.0
0.5
0.0
f1 (ppm)

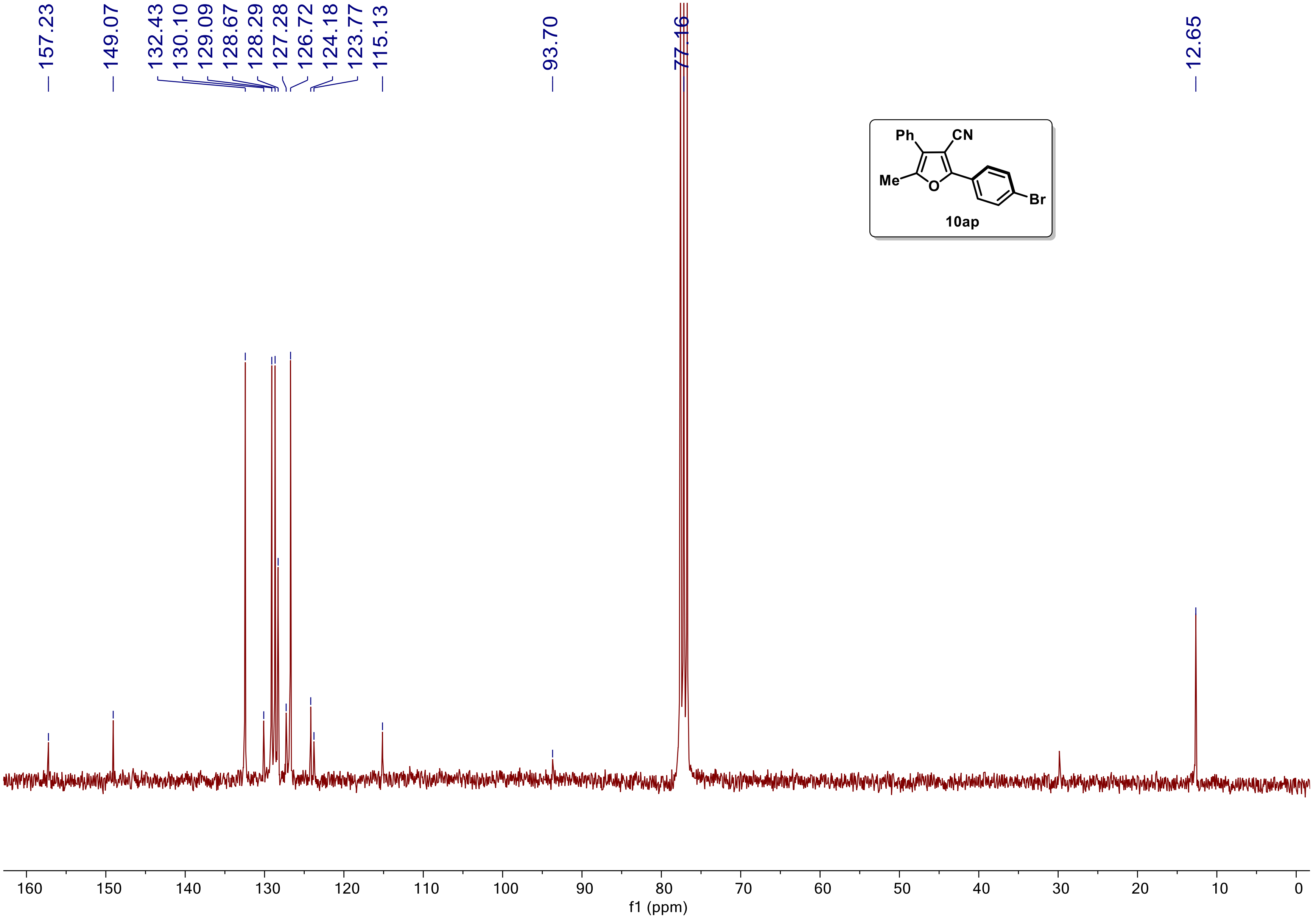
157.23
149.07
132.43
130.10
129.09
128.67
128.29
127.28
126.72
124.18
123.77
115.13
93.70
77.16
12.65
Ph
CN
Me
O
Br
10ap
160 150 140 130 120 110 100 90 80 70 60 50 40 30 20 10 0
f1 (ppm)

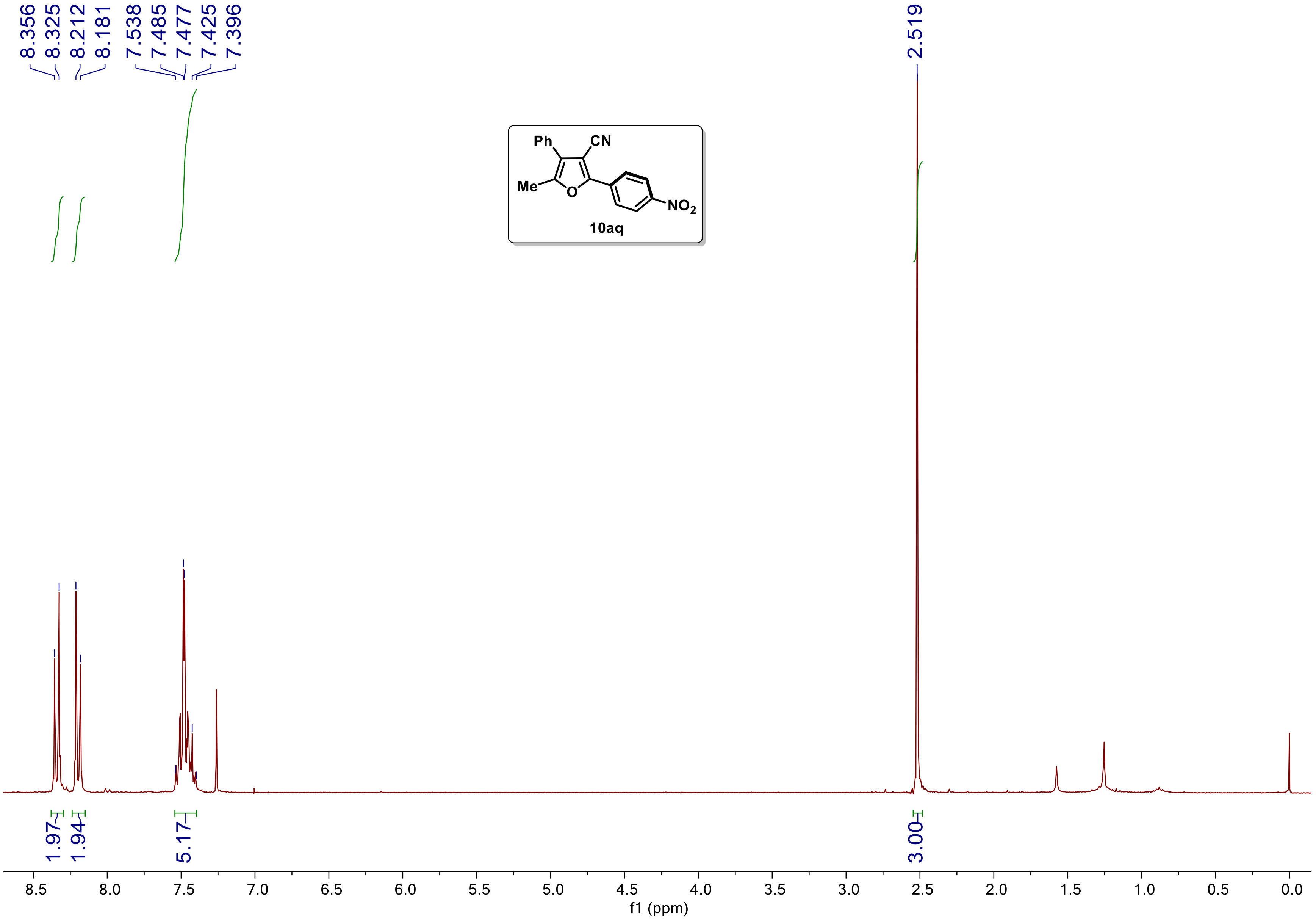
8.356
8.325
8.212
8.181
7.538
7.485
7.477
7.425
7.396
2.519
Ph
CN
Me
O
NO2
10aq
1.97
1.94
5.17
3.00
8.5
8.0
7.5
7.0
6.5
6.0
5.5
5.0
4.5
4.0
3.5
3.0
2.5
2.0
1.5
1.0
0.5
0.0
f1 (ppm)

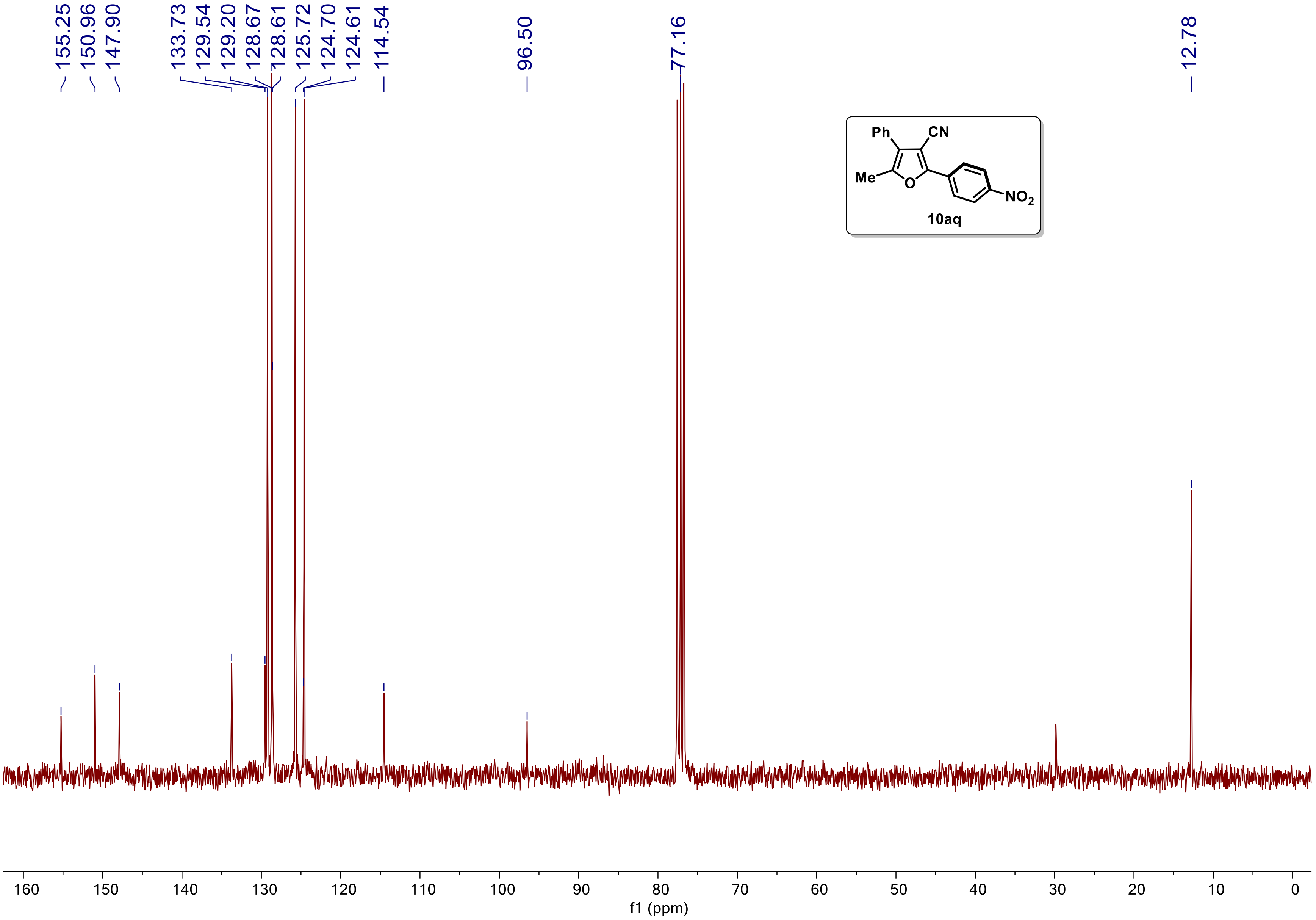
155.25
150.96
147.90
133.73
129.54
129.20
128.67
128.61
125.72
124.70
124.61
114.54
96.50
77.16
12.78
Ph
CN
Me
O
NO2
10aq
160
150
140
130
120
110
100
90
80
70
60
50
40
30
20
10
0
f1 (ppm)

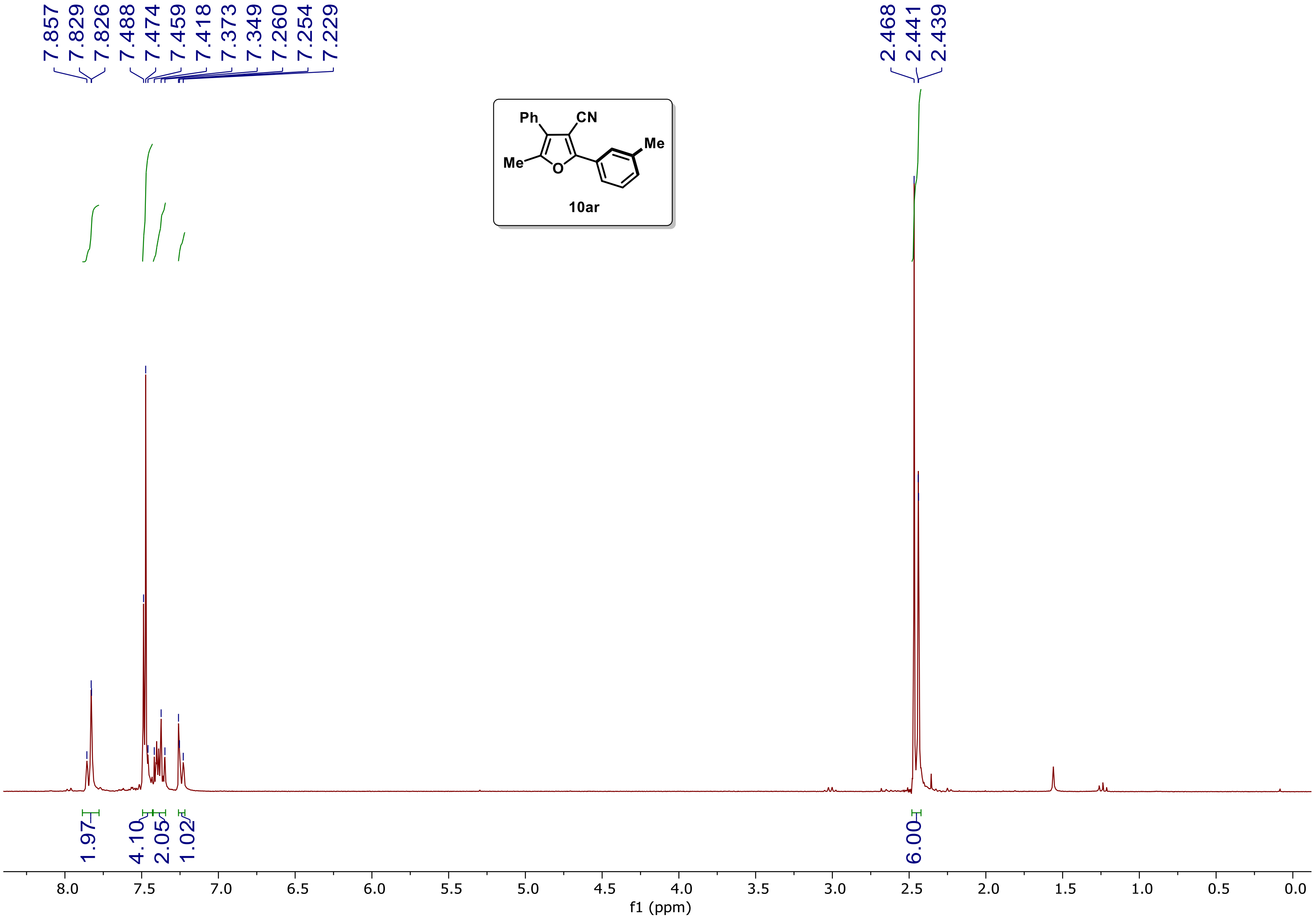
7.857 7.829 7.826 7.488 7.474 7.459 7.418 7.373 7.349 7.260 7.254 7.229
2.468 2.441 2.439
Ph
CN
Me
Me
O
10ar
1.97
4.10
2.05
1.02
6.00
8.0 7.5 7.0 6.5 6.0 5.5 5.0 4.5 4.0 3.5 3.0 2.5 2.0 1.5 1.0 0.5 0.0
f1 (ppm)

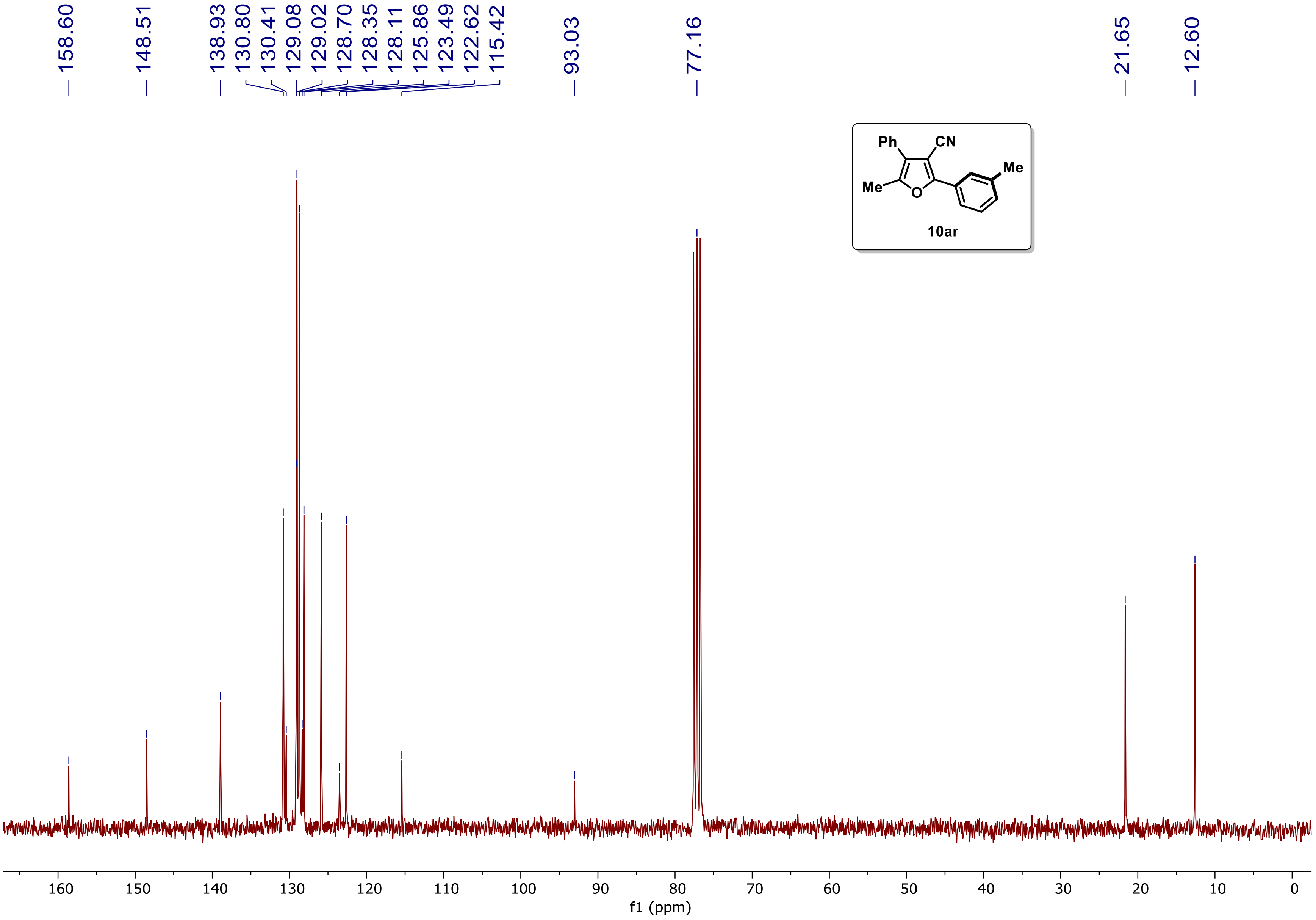
158.60
148.51
138.93
130.80
130.41
129.08
129.02
128.70
128.35
128.11
125.86
123.49
122.62
115.42
93.03
77.16
21.65
12.60
Ph
CN
Me
Me
O
10ar
160
150
140
130
120
110
100
90
80
70
60
50
40
30
20
10
0
f1 (ppm)

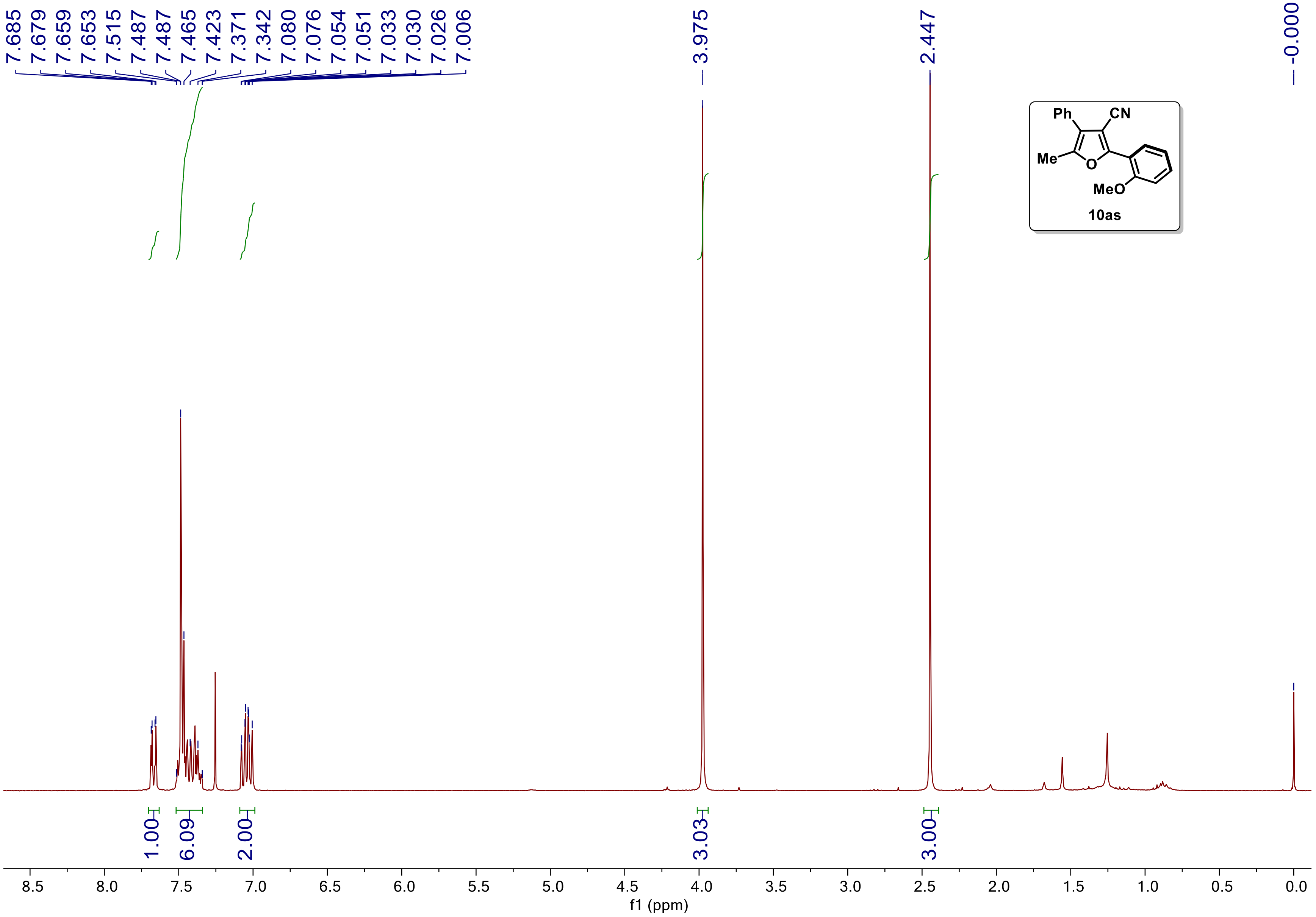
7.685
7.679
7.659
7.653
7.515
7.487
7.487
7.465
7.423
7.371
7.342
7.080
7.076
7.054
7.051
7.033
7.030
7.026
7.006
3.975
2.447
-0.000
Ph
CN
Me
O
MeO
10as
1.00
6.09
2.00
3.03
3.00
8.5
8.0
7.5
7.0
6.5
6.0
5.5
5.0
4.5
4.0
3.5
3.0
2.5
2.0
1.5
1.0
0.5
0.0
f1 (ppm)

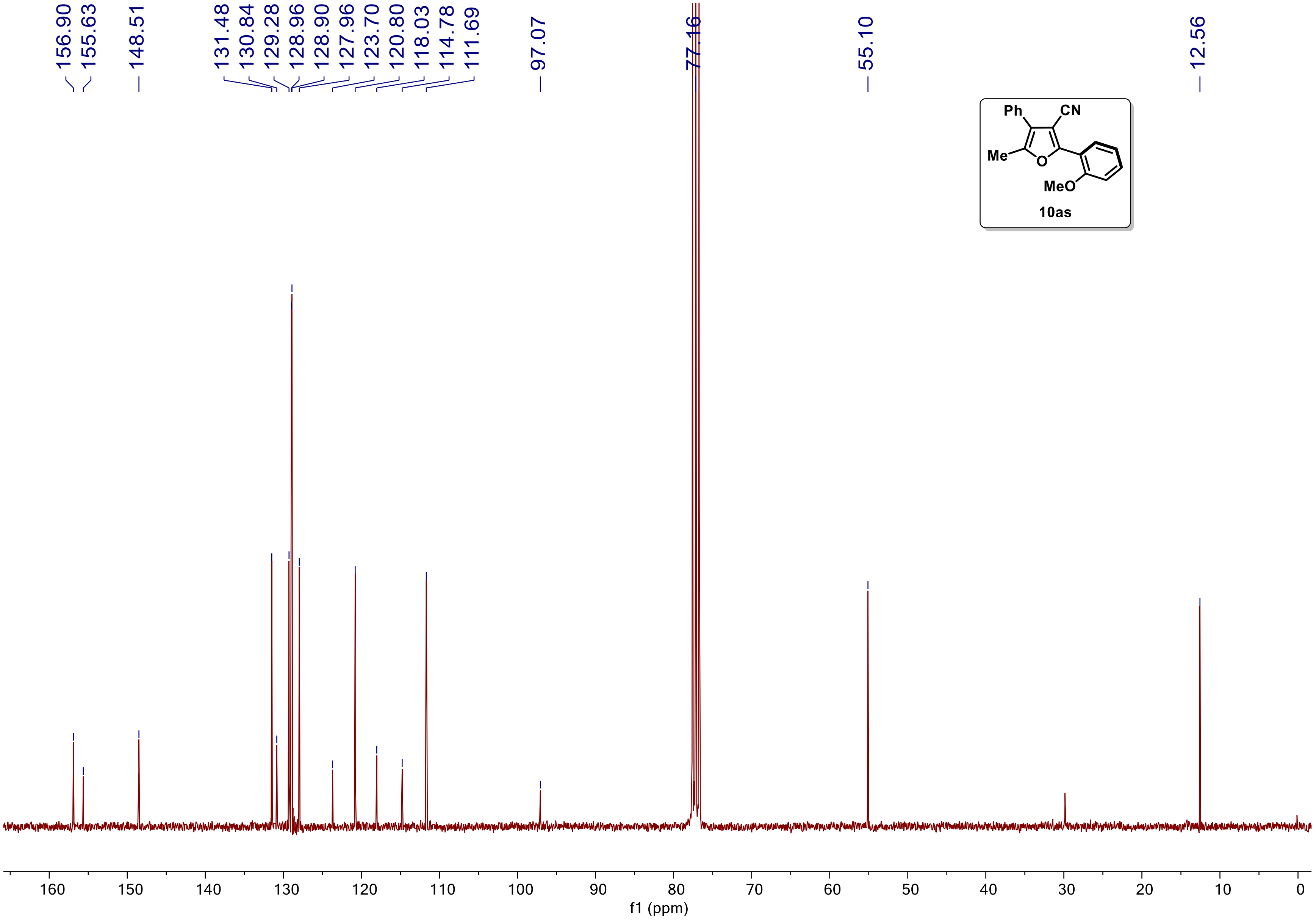

156.90
155.63
148.51
131.48
130.84
129.28
128.96
128.90
127.96
123.70
120.80
118.03
114.78
111.69
97.07
77.16
55.10
12.56
Ph
CN
Me
O
MeO
10as
160
150
140
130
120
110
100
90
80
70
60
50
40
30
20
10
0
f1 (ppm)

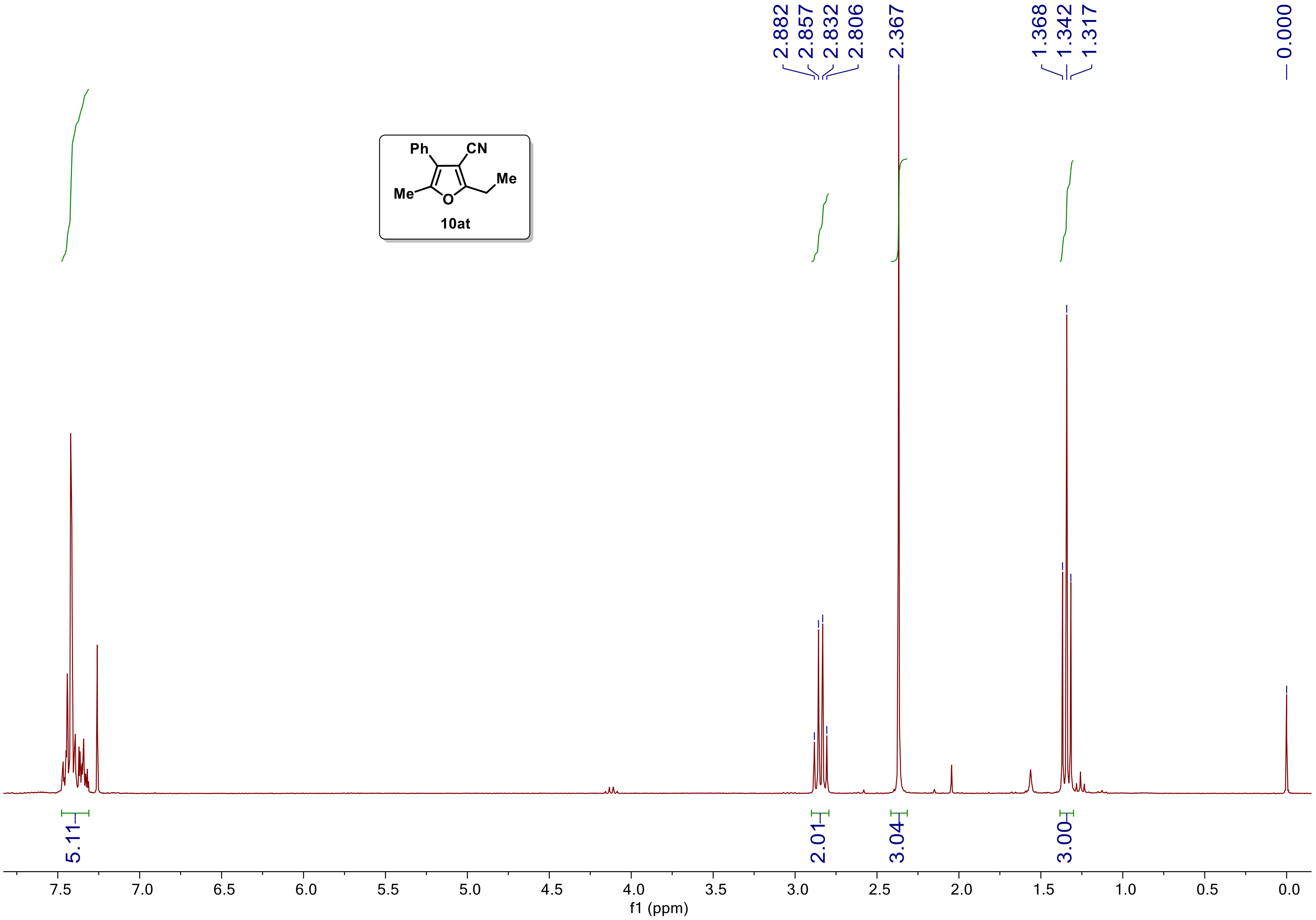

2.882
2.857
2.832
2.806
2.367
1.368
1.342
1.317
0.000
Ph
CN
Me
Me
O
10at
5.11
2.01
3.04
3.00
7.5
7.0
6.5
6.0
5.5
5.0
4.5
4.0
3.5
3.0
2.5
2.0
1.5
1.0
0.5
0.0
f1 (ppm)

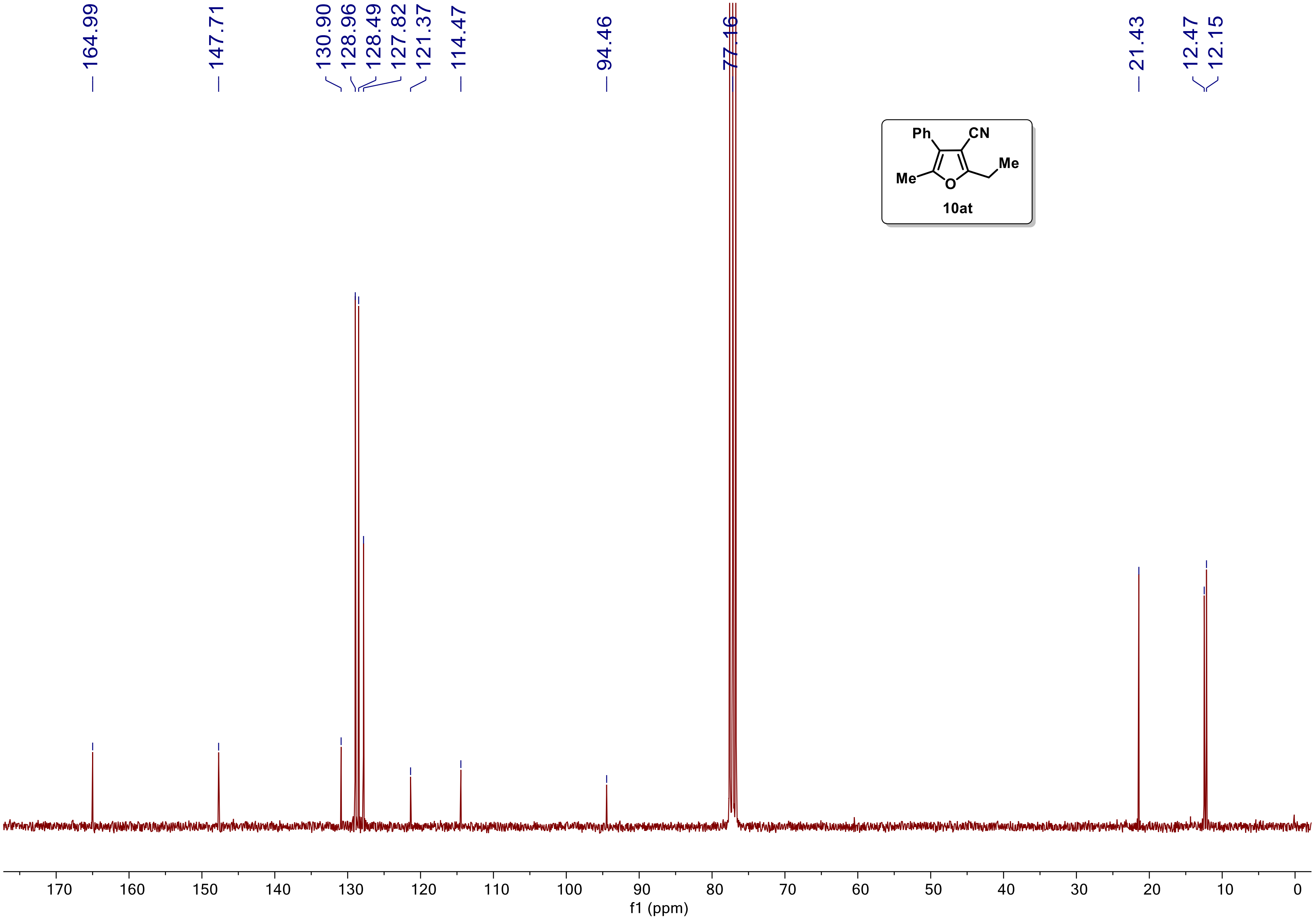
164.99
147.71
130.90
128.96
128.49
127.82
121.37
114.47
94.46
77.16
21.43
12.47
12.15
Ph
CN
Me
Me
O
10at
170
160
150
140
130
120
110
100
90
80
70
60
50
40
30
20
10
0
f1 (ppm)

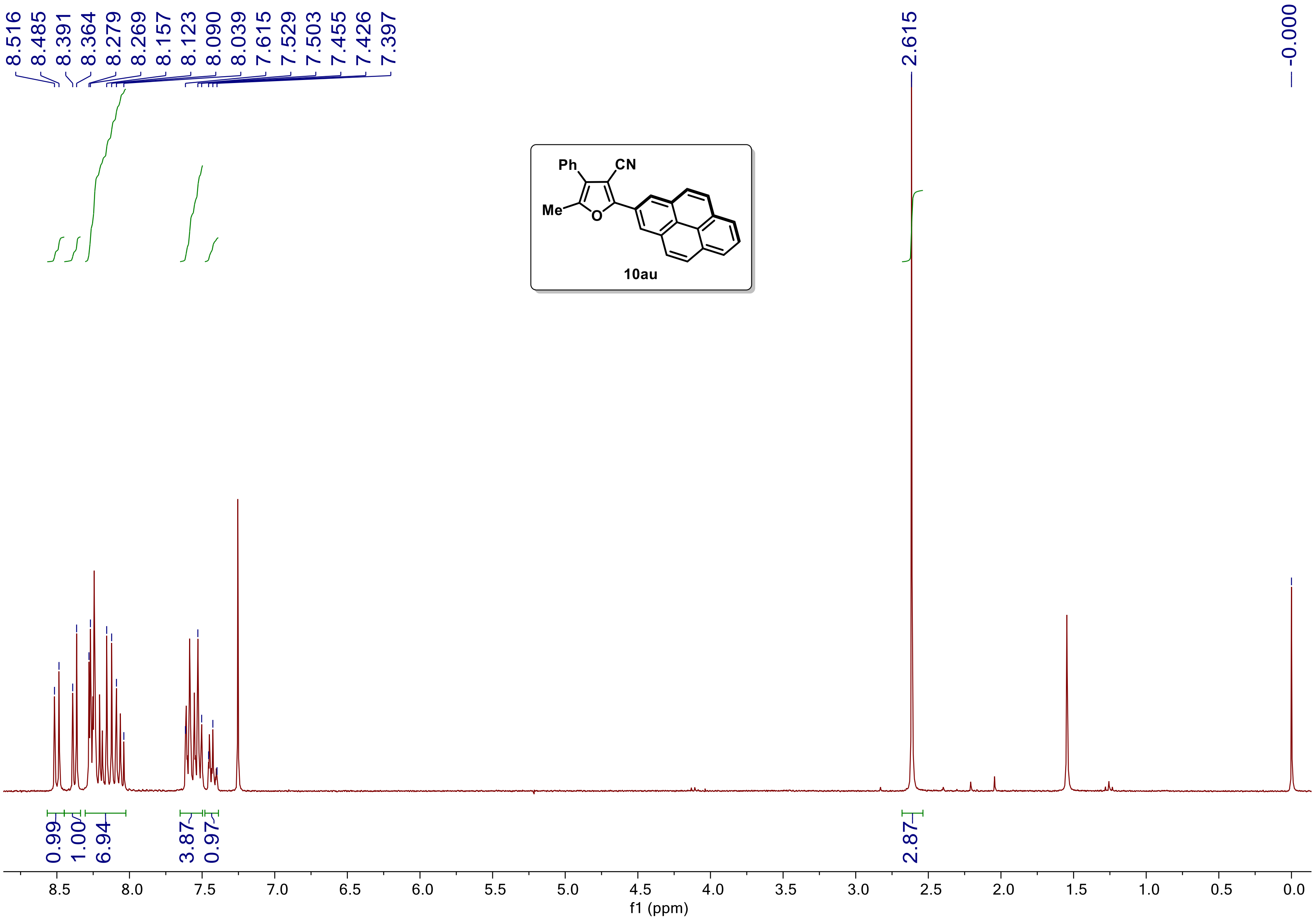
8.516
8.485
8.391
8.364
8.279
8.269
8.157
8.123
8.090
8.039
7.615
7.529
7.503
7.455
7.426
7.397
2.615
-0.000
Ph
CN
Me
O
10au
0.99
1.00
6.94
3.87
0.97
2.87
f1 (ppm)

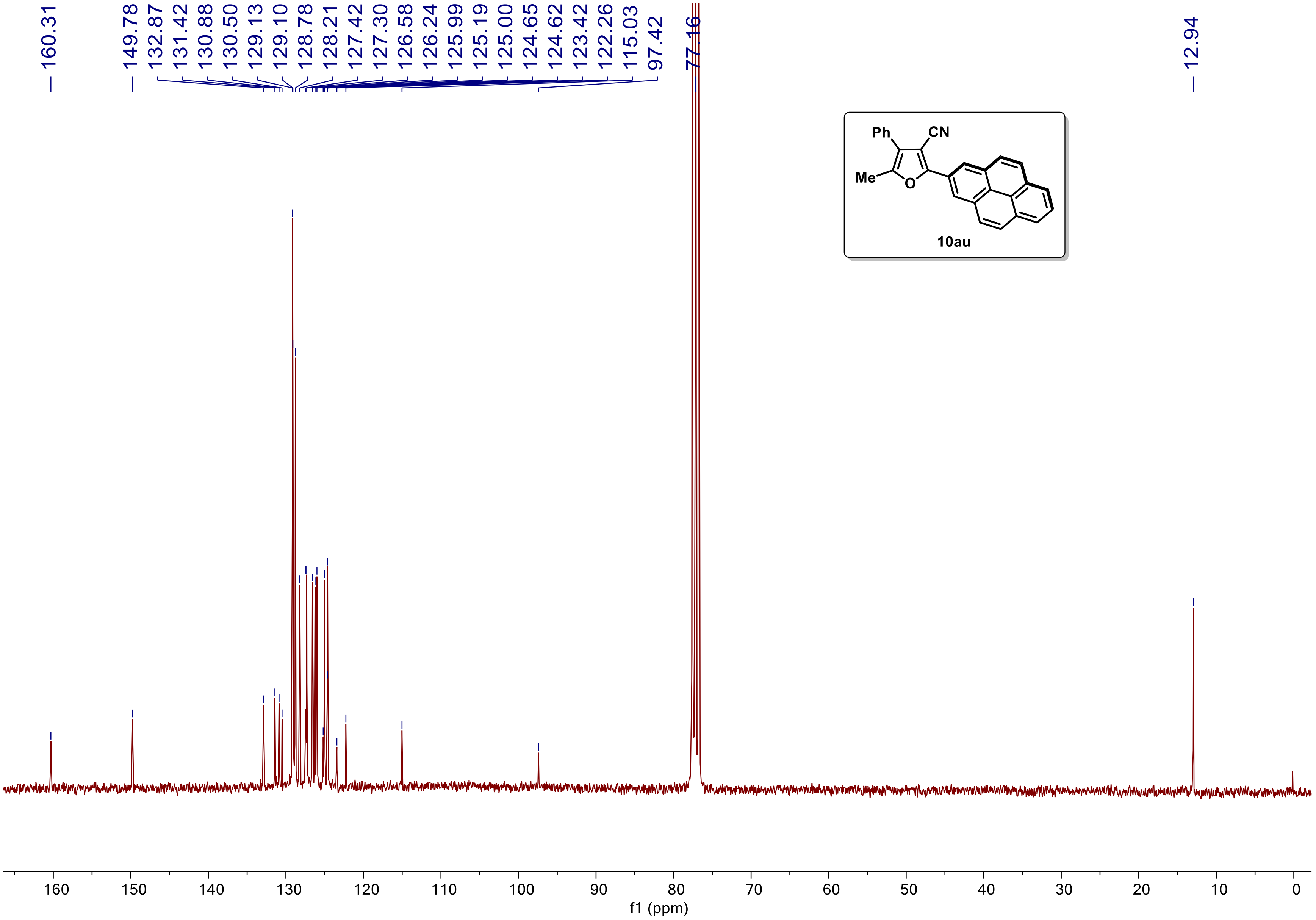
160.31
149.78
132.87
131.42
130.88
130.50
129.13
129.10
128.78
128.21
127.42
127.30
126.58
126.24
125.99
125.19
125.00
124.65
124.62
123.42
122.26
115.03
97.42
77.16
12.94
Ph
CN
Me
O
10au
160
150
140
130
120
110
100
90
80
70
60
50
40
30
20
10
0
f1 (ppm)

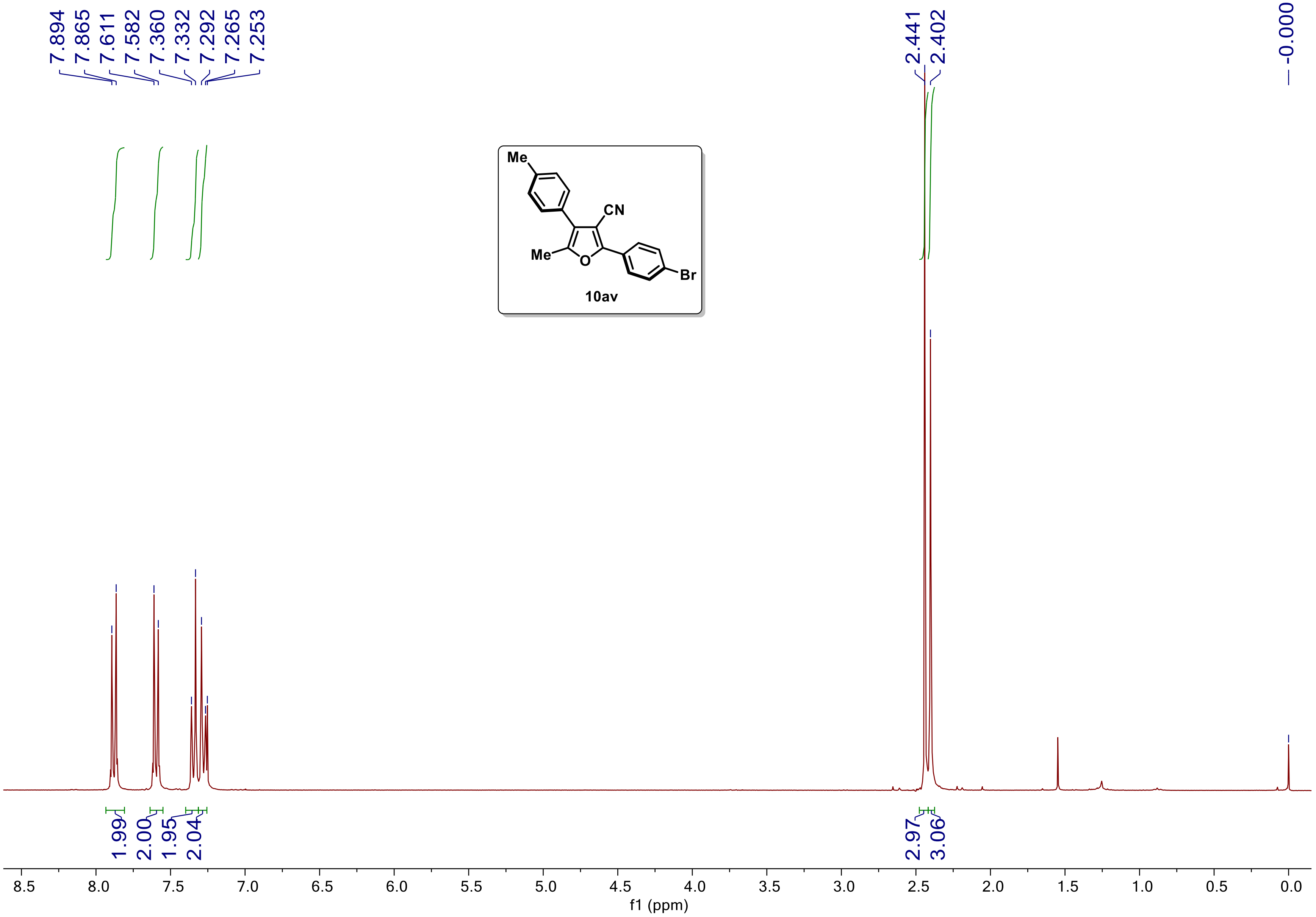
7.894
7.865
7.611
7.582
7.360
7.332
7.292
7.265
7.253
2.441
2.402
-0.000
Me
CN
Me
O
Br
10av
1.99
2.00
1.95
2.04
2.97
3.06
8.5
8.0
7.5
7.0
6.5
6.0
5.5
5.0
4.5
4.0
3.5
3.0
2.5
2.0
1.5
1.0
0.5
0.0
f1 (ppm)

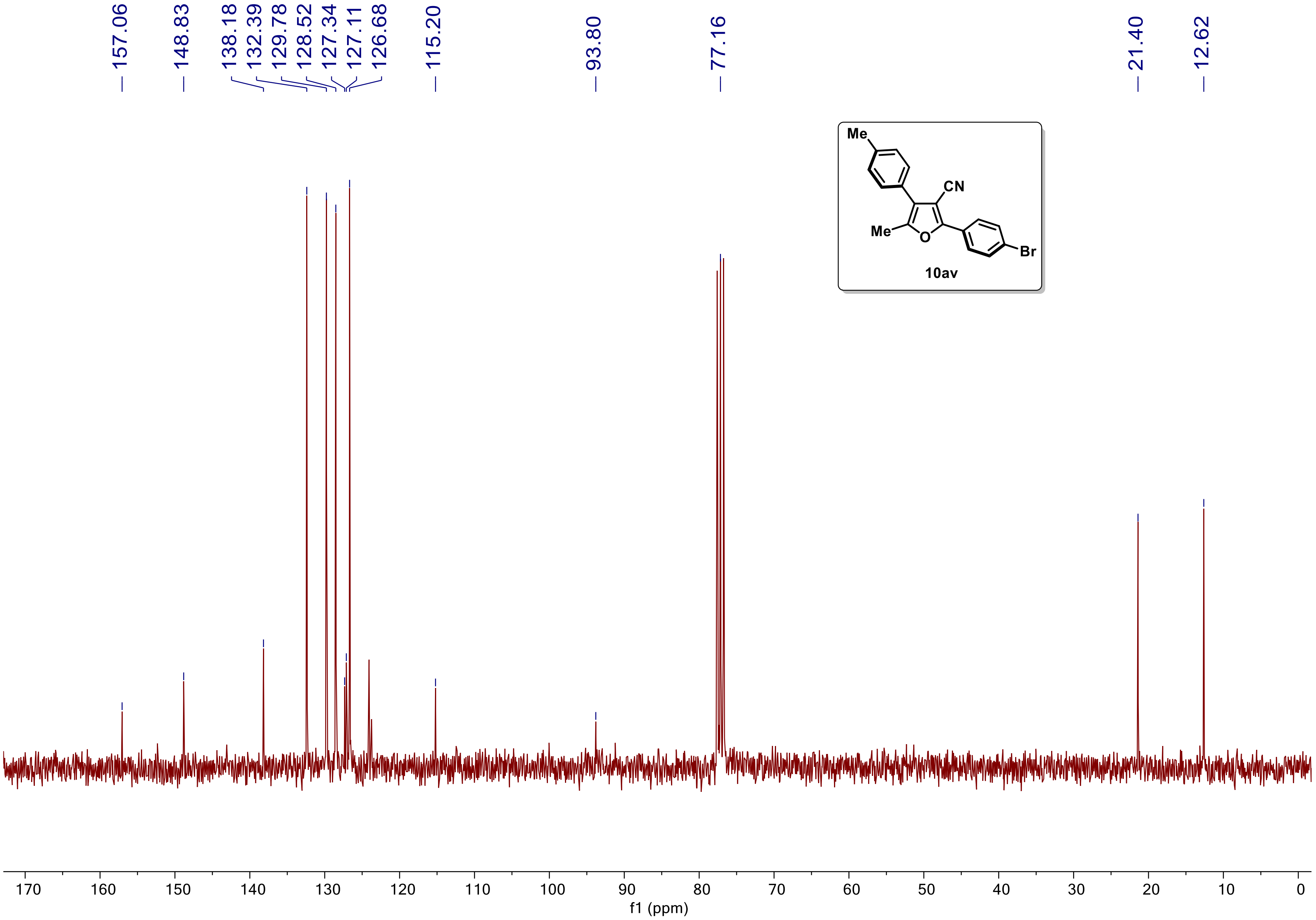
157.06
148.83
138.18
132.39
129.78
128.52
127.34
127.11
126.68
115.20
93.80
77.16
21.40
12.62
Me
CN
Me
O
Br
10av
170 160 150 140 130 120 110 100 90 80 70 60 50 40 30 20 10 0
f1 (ppm)

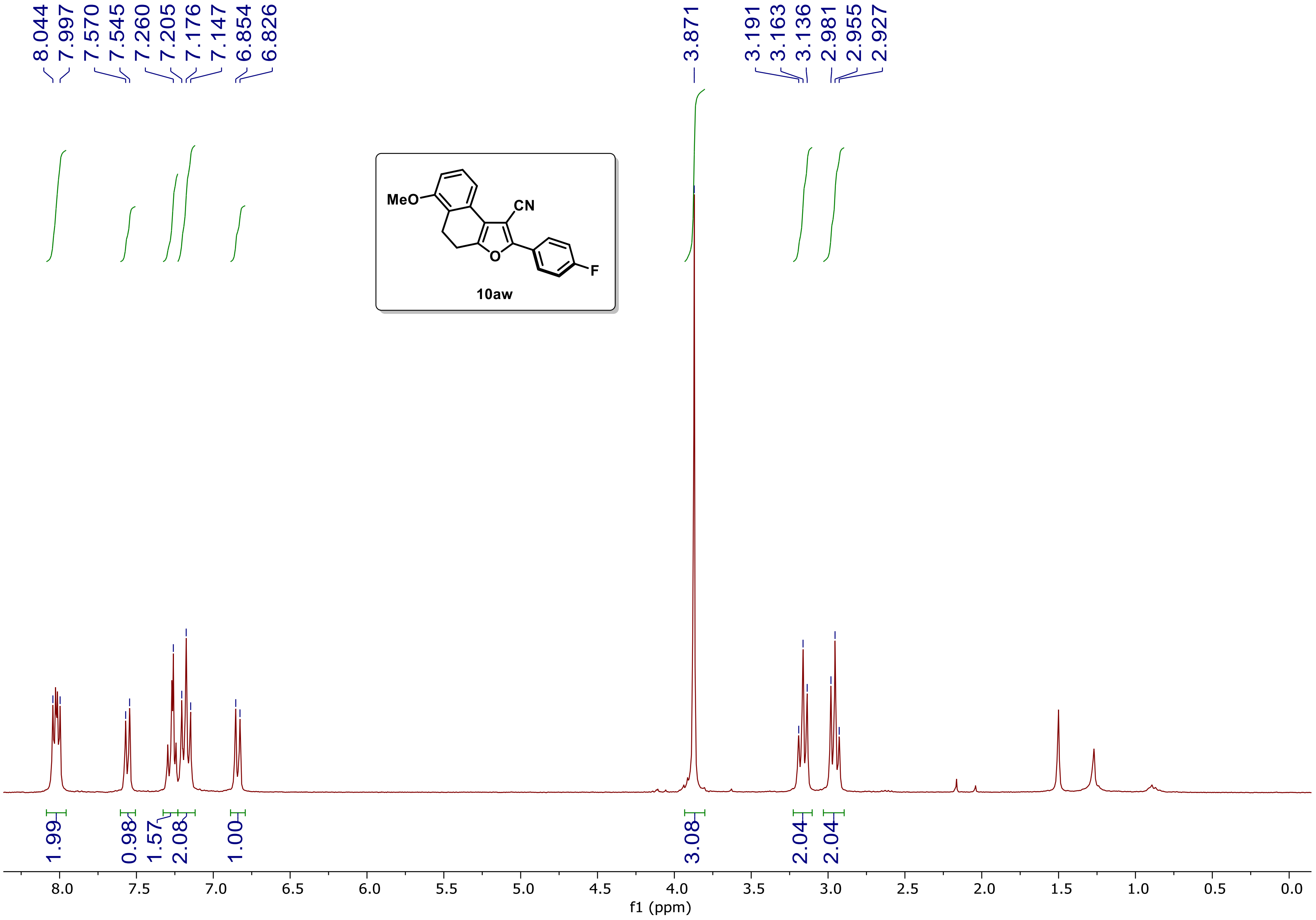
8.044
7.997
7.570
7.545
7.260
7.205
7.176
7.147
6.854
6.826
3.871
3.191
3.163
3.136
2.981
2.955
2.927
MeO
CN
O
F
10aw
1.99
0.98
1.57
2.08
1.00
3.08
2.04
2.04
8.0
7.5
7.0
6.5
6.0
5.5
5.0
4.5
4.0
3.5
3.0
2.5
2.0
1.5
1.0
0.5
0.0
f1 (ppm)

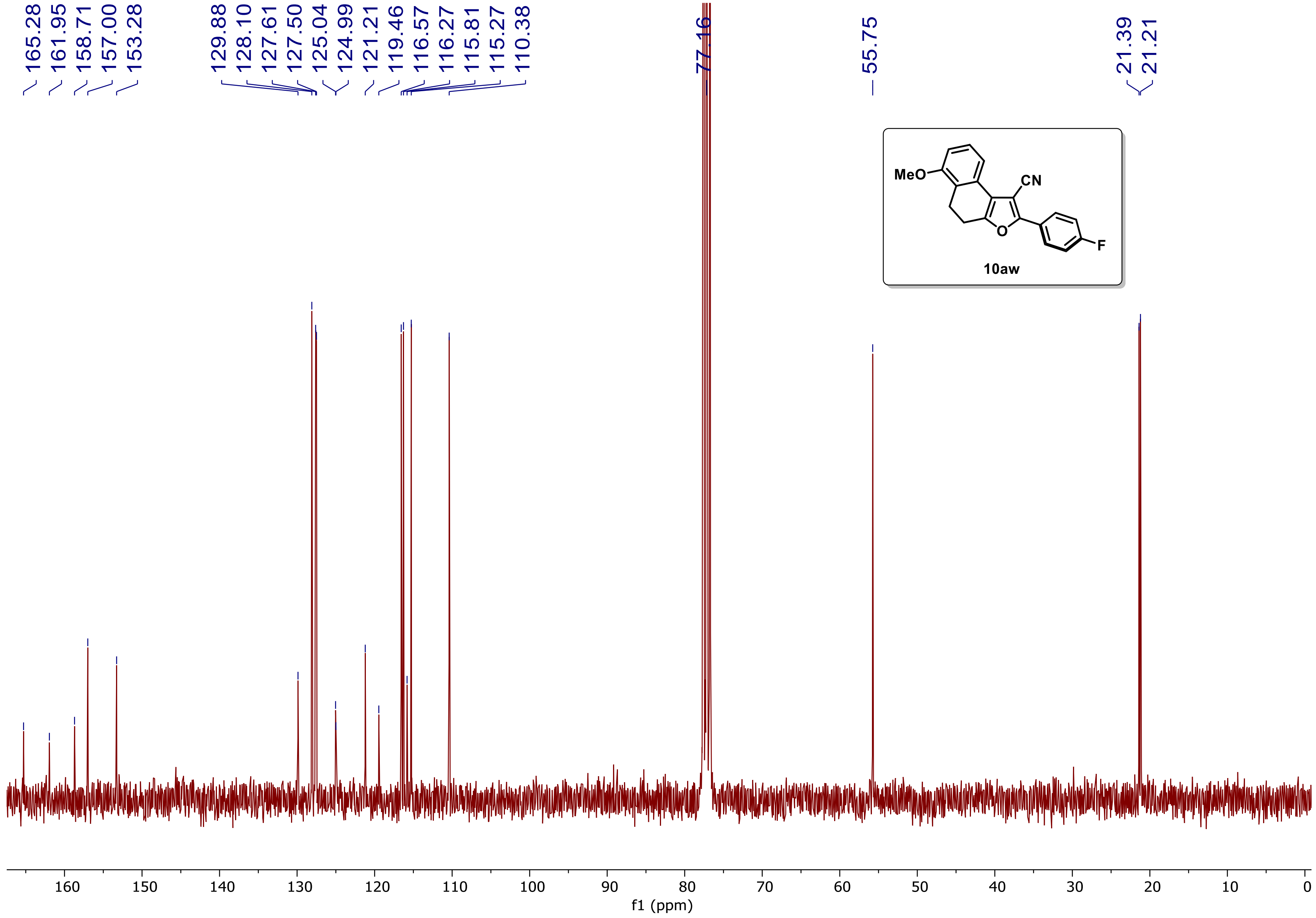
165.28
161.95
158.71
157.00
153.28
129.88
128.10
127.61
127.50
125.04
124.99
121.21
119.46
116.57
116.27
115.81
115.27
110.38
77.16
55.75
21.39
21.21
MeO
CN
O
F
10aw
160
150
140
130
120
110
100
90
80
70
60
50
40
30
20
10
0
f1 (ppm)

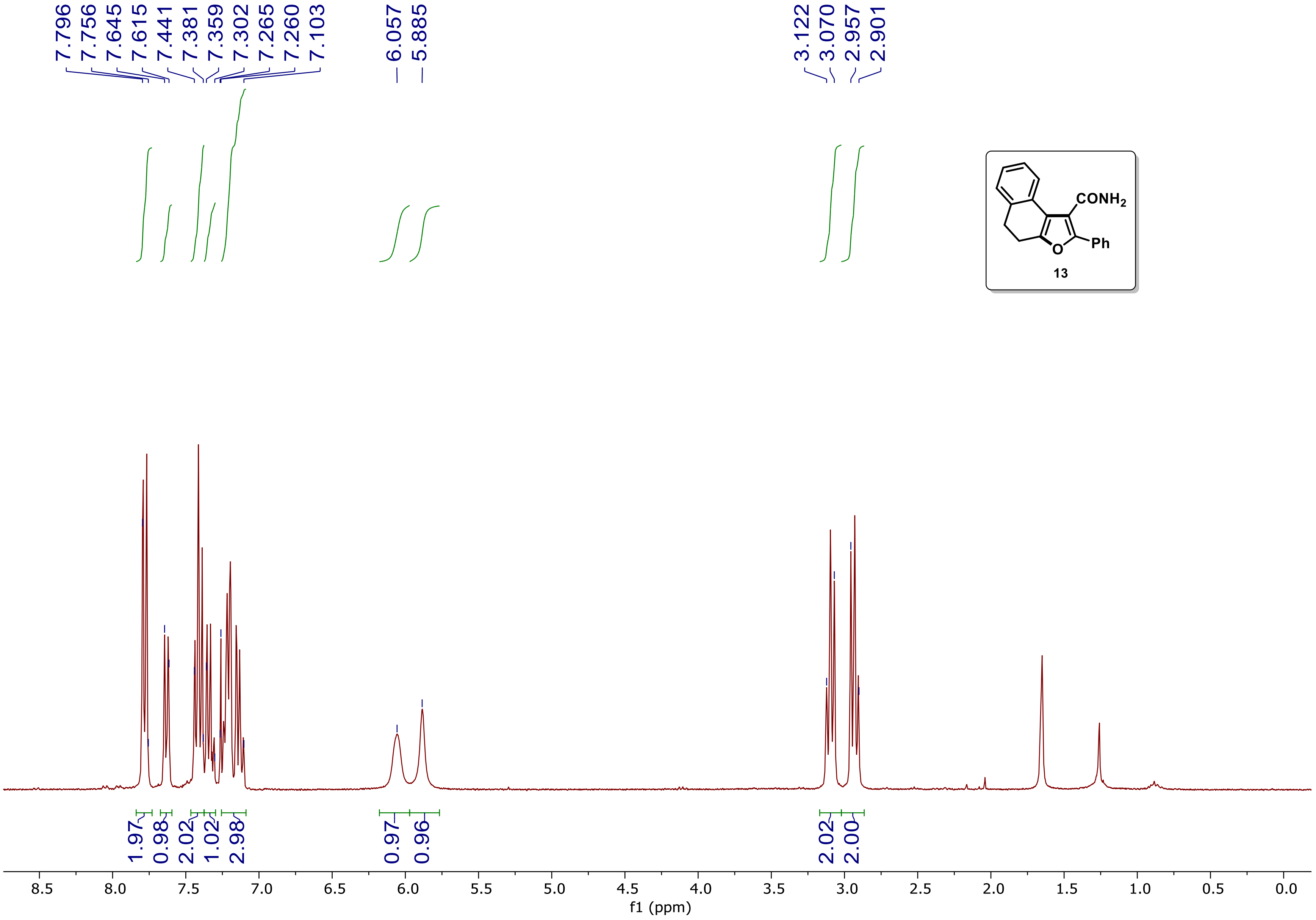
7.796
7.756
7.645
7.615
7.441
7.381
7.359
7.302
7.265
7.260
7.103
6.057
5.885
3.122
3.070
2.957
2.901
CONH2
O
Ph
13
1.97
0.98
2.02
1.02
2.98
0.97
0.96
2.02
2.00
8.5
8.0
7.5
7.0
6.5
6.0
5.5
5.0
4.5
4.0
3.5
3.0
2.5
2.0
1.5
1.0
0.5
0.0
f1 (ppm)

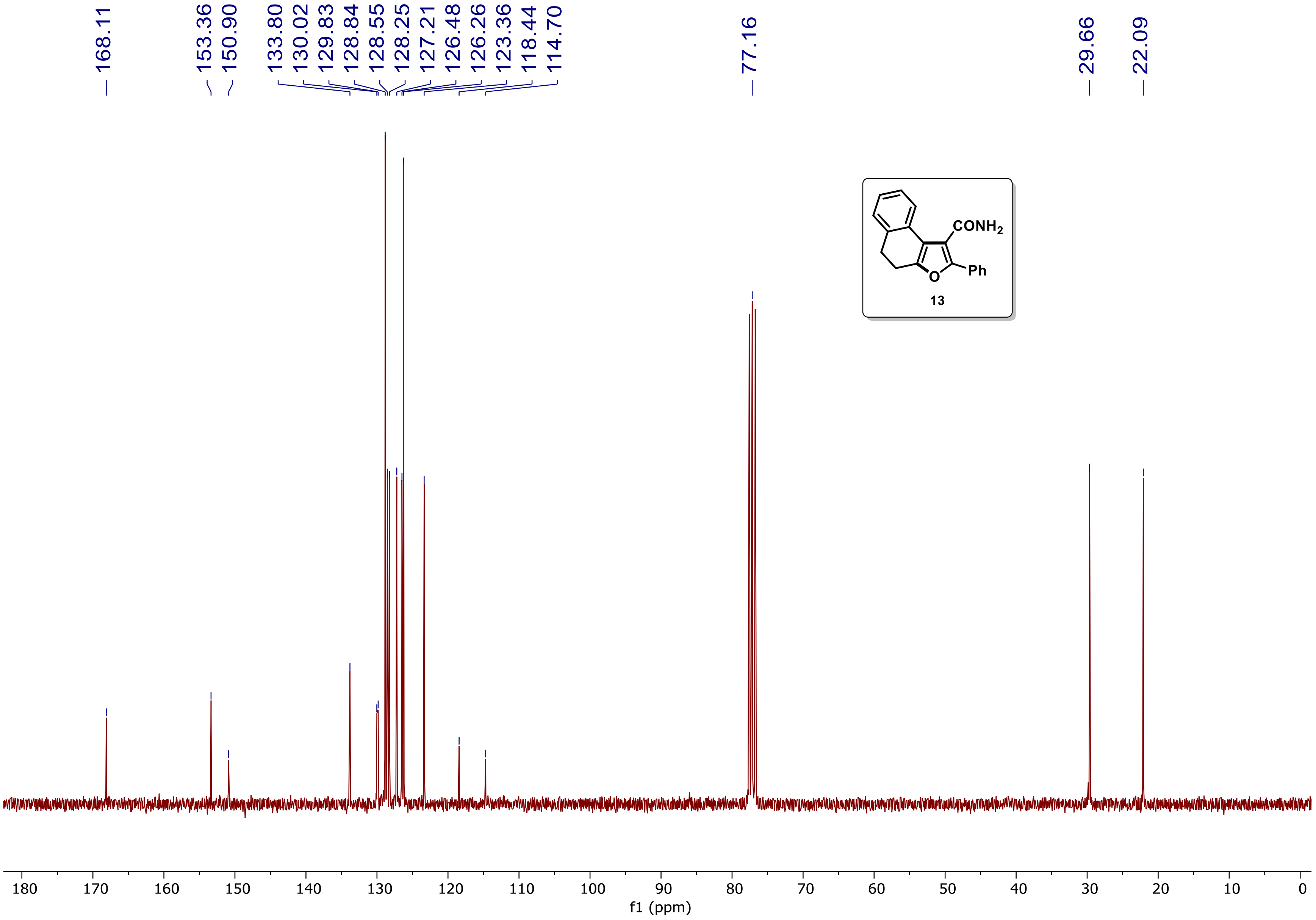
168.11
153.36
150.90
133.80
130.02
129.83
128.84
128.55
128.25
127.21
126.48
126.26
123.36
118.44
114.70
77.16
29.66
22.09
CONH2
Ph
O
13
180 170 160 150 140 130 120 110 100 90 80 70 60 50 40 30 20 10 0
f1 (ppm)

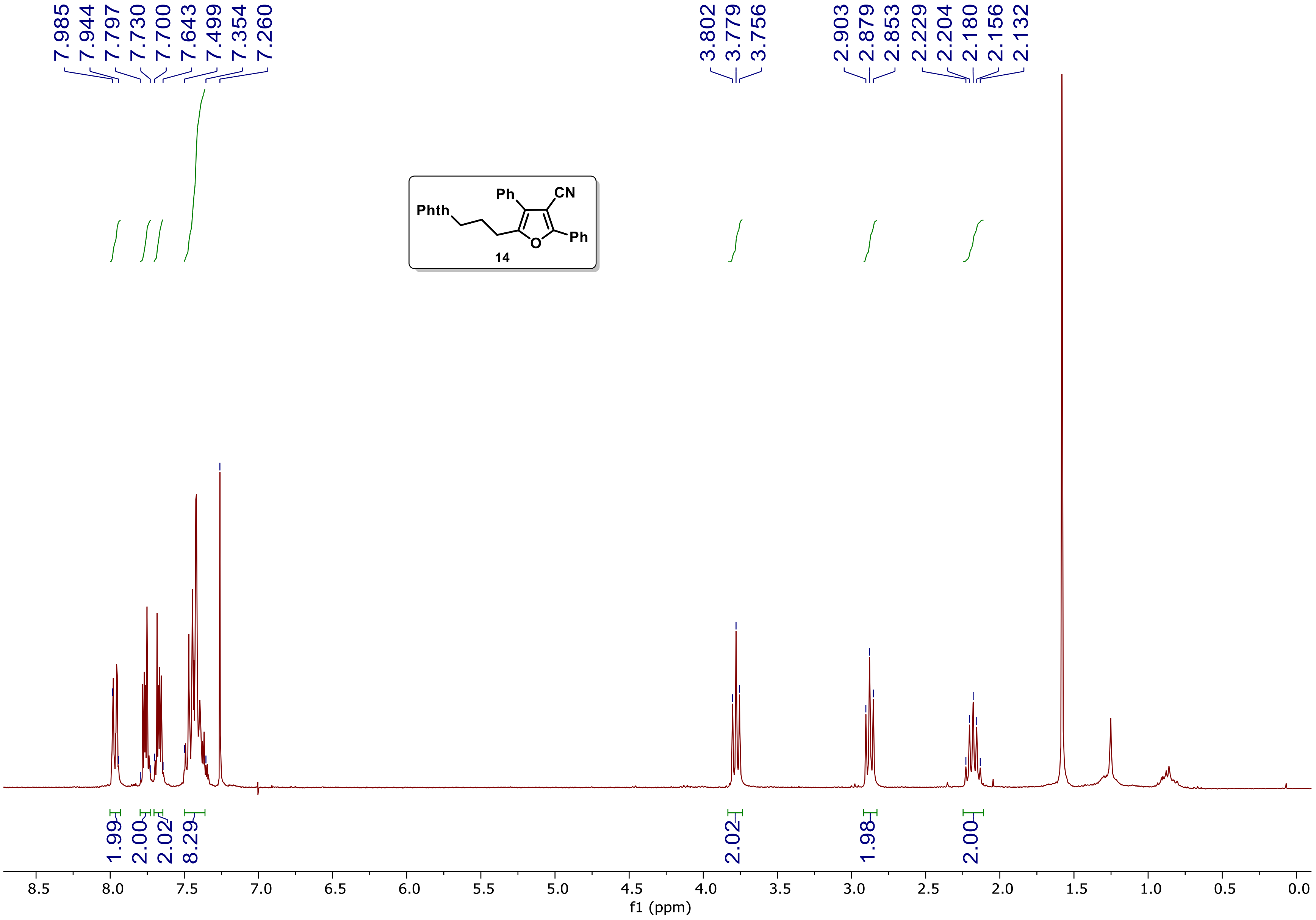
7.985
7.944
7.797
7.730
7.700
7.643
7.499
7.354
7.260
3.802
3.779
3.756
2.903
2.879
2.853
2.229
2.204
2.180
2.156
2.132
Phth
Ph
CN
O
Ph
14
1.99
2.00
2.02
8.29
2.02
1.98
2.00
8.5
8.0
7.5
7.0
6.5
6.0
5.5
5.0
4.5
4.0
3.5
3.0
2.5
2.0
1.5
1.0
0.5
0.0
f1 (ppm)

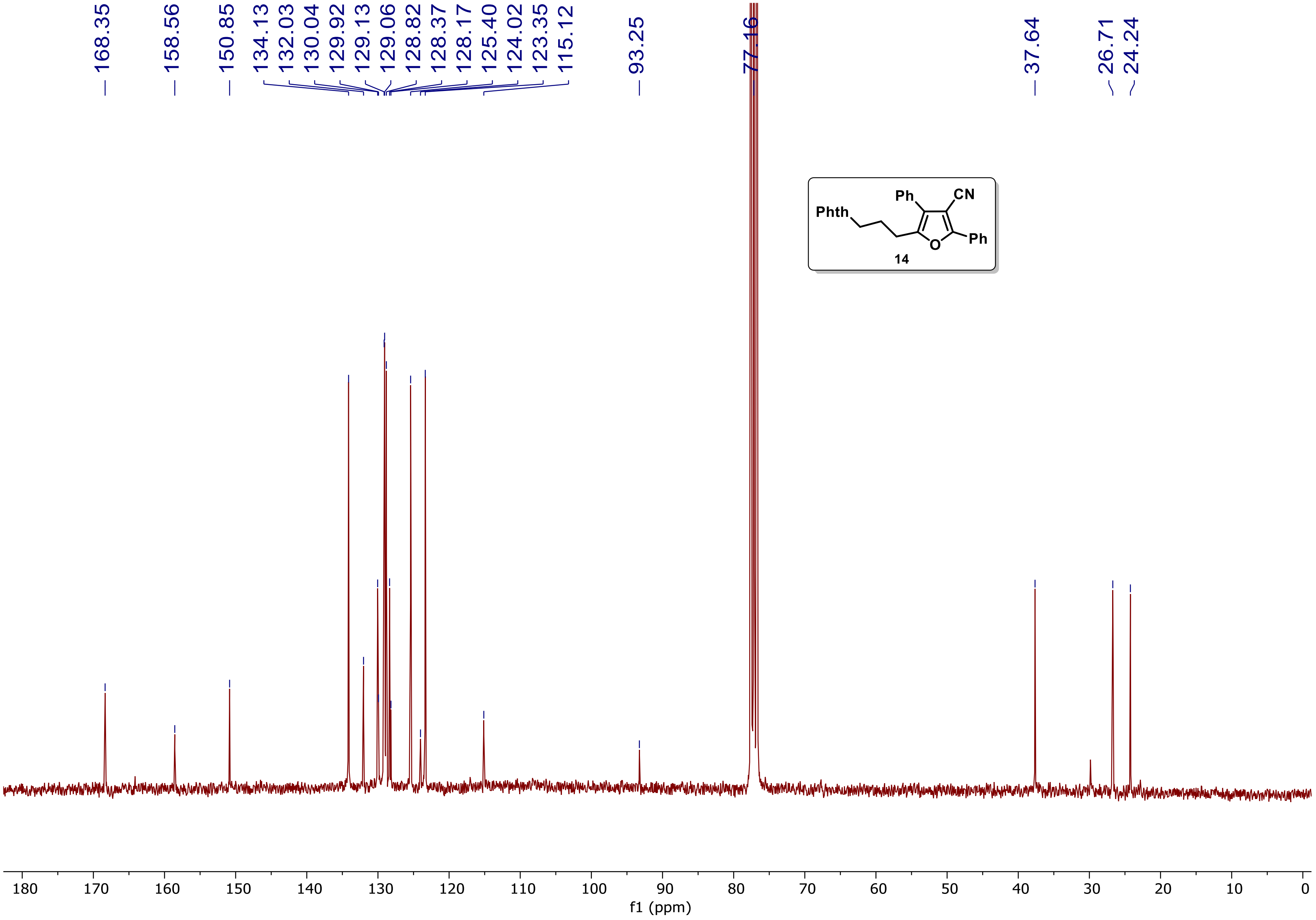
168.35
158.56
150.85
134.13
132.03
130.04
129.92
129.13
129.06
128.82
128.37
128.17
125.40
124.02
123.35
115.12
93.25
77.16
37.64
26.71
24.24
Phth
Ph
CN
Ph
O
14
180 170 160 150 140 130 120 110 100 90 80 70 60 50 40 30 20 10 0
f1 (ppm)

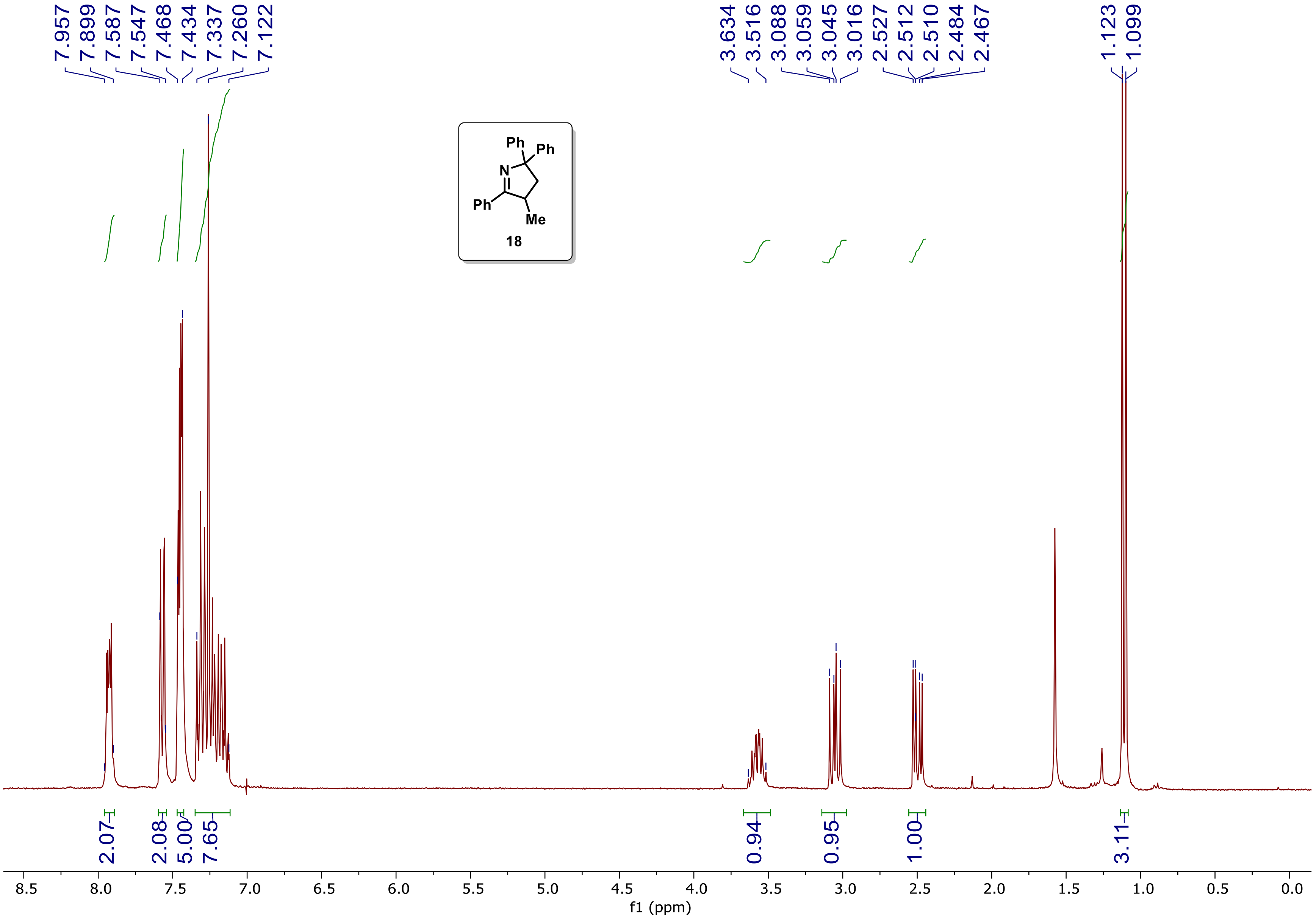

7.957
7.899
7.587
7.547
7.468
7.434
7.337
7.260
7.122
3.634
3.516
3.088
3.059
3.045
3.016
2.527
2.512
2.510
2.484
2.467
1.123
1.099
Ph
Ph
N
Ph
Me
18
2.07
2.08
5.00
7.65
0.94
0.95
1.00
3.11
8.5
8.0
7.5
7.0
6.5
6.0
5.5
5.0
4.5
4.0
3.5
3.0
2.5
2.0
1.5
1.0
0.5
0.0
f1 (ppm)

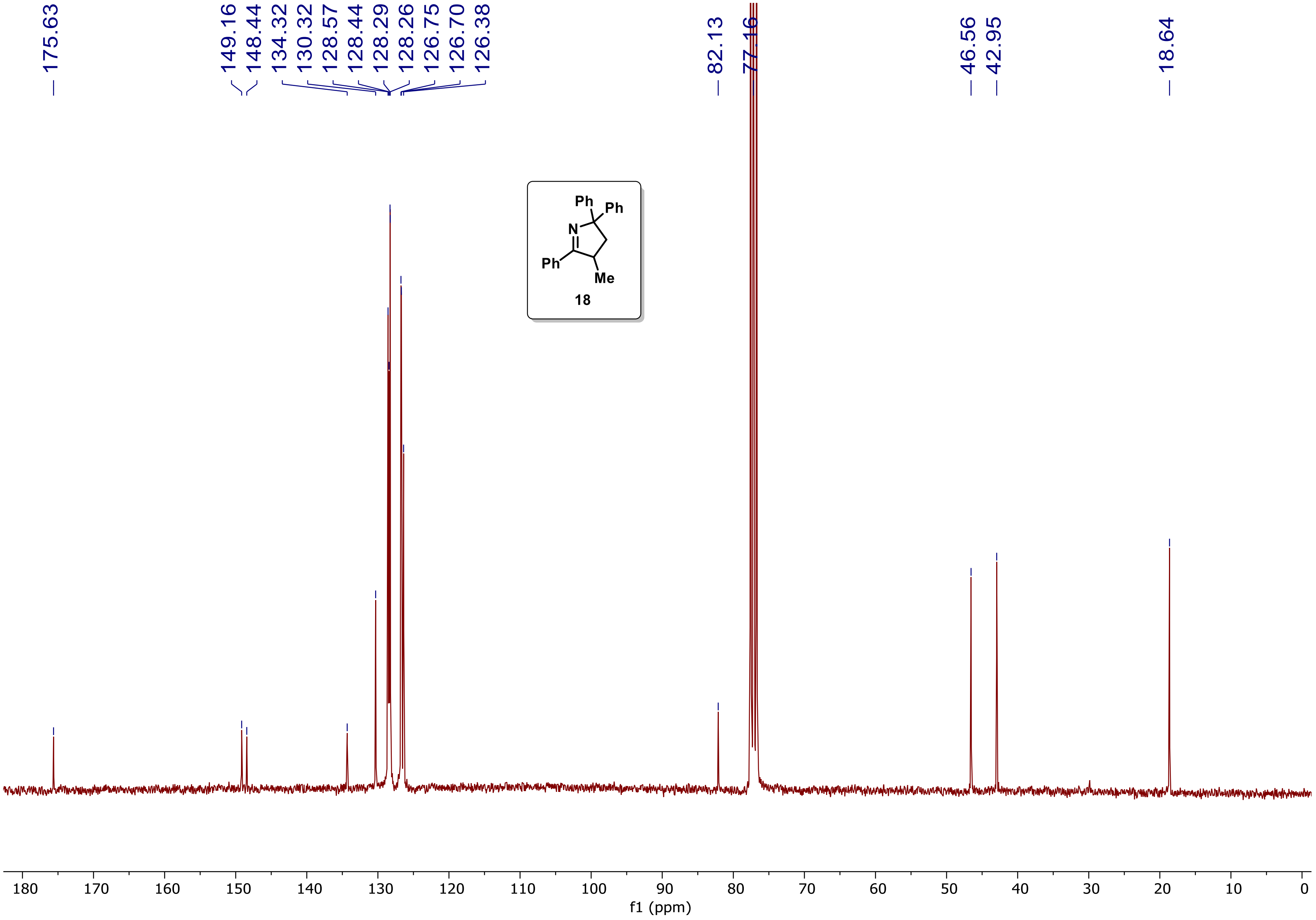
175.63
149.16
148.44
134.32
130.32
128.57
128.44
128.29
128.26
126.75
126.70
126.38
82.13
77.16
46.56
42.95
18.64
Ph
Ph
N
Ph
Me
18
180 170 160 150 140 130 120 110 100 90 80 70 60 50 40 30 20 10 0
f1 (ppm)
